# Are fatty nuts a weighty concern? A systematic review and meta‐analysis and dose–response meta‐regression of prospective cohorts and randomized controlled trials

**DOI:** 10.1111/obr.13330

**Published:** 2021-09-08

**Authors:** Stephanie K. Nishi, Effie Viguiliouk, Sonia Blanco Mejia, Cyril W. C. Kendall, Richard P. Bazinet, Anthony J. Hanley, Elena M. Comelli, Jordi Salas Salvadó, David J. A. Jenkins, John L. Sievenpiper

**Affiliations:** ^1^ Department of Nutritional Sciences, Temerty Faculty of Medicine University of Toronto Toronto Ontario Canada; ^2^ Toronto 3D (Diet, Digestive Tract and Disease) Knowledge Synthesis and Clinical Trials Unit Toronto Ontario Canada; ^3^ Clinical Nutrition and Risk Factor Modification Center St. Michael's Hospital Toronto Ontario Canada; ^4^ Biomedical Research Centre for Obesity Physiopathology and Nutrition Network (CIBEROBN) Instituto de Salud Carlos III (ISCIII) Madrid Spain; ^5^ Departament de Bioquimica i Biotecnologia, Unitat de Nutrició Humana Universitat Rovira i Virgili Reus Spain; ^6^ Institut d'Investigació Sanitària Pere Virgili (IISPV) Reus Spain; ^7^ College of Pharmacy and Nutrition University of Saskatchewan Saskatoon Saskatchewan Canada; ^8^ Department of Medicine University of Toronto Toronto Ontario Canada; ^9^ Dalla Lana School of Public Health University of Toronto Toronto Ontario Canada; ^10^ Joannah and Brian Lawson Centre for Child Nutrition University of Toronto Toronto Ontario Canada; ^11^ Division of Endocrinology & Metabolism St. Michael's Hospital Toronto Ontario Canada; ^12^ Li Ka Shing Knowledge Institute St. Michael's Hospital Toronto Ontario Canada

**Keywords:** body weight, meta‐analysis, nuts, systematic review

## Abstract

Nuts are recommended for cardiovascular health, yet concerns remain that nuts may contribute to weight gain due to their high energy density. A systematic review and meta‐analysis of prospective cohorts and randomized controlled trials (RCTs) was conducted to update the evidence, provide a dose–response analysis, and assess differences in nut type, comparator and more in subgroup analyses. MEDLINE, EMBASE, and Cochrane were searched, along with manual searches. Data from eligible studies were pooled using meta‐analysis methods. Interstudy heterogeneity was assessed (Cochran Q statistic) and quantified (*I* ^2^ statistic). Certainty of the evidence was assessed by Grading of Recommendations Assessment, Development, and Evaluation (GRADE). Six prospective cohort studies (7 unique cohorts, *n* = 569,910) and 86 RCTs (114 comparisons, *n =* 5873) met eligibility criteria. Nuts were associated with lower incidence of overweight/obesity (RR 0.93 [95% CI 0.88 to 0.98] *P* < 0.001, “moderate” certainty of evidence) in prospective cohorts. RCTs presented no adverse effect of nuts on body weight (MD 0.09 kg, [95% CI −0.09 to 0.27 kg] *P* < 0.001, “high” certainty of evidence). Meta‐regression showed that higher nut intake was associated with reductions in body weight and body fat. Current evidence demonstrates the concern that nut consumption contributes to increased adiposity appears unwarranted.

**Abbreviations**
AHS‐2: Adventist Health Study‐2
BMI: Body mass index
CI: Confidence intervals
DASH: Dietary Approaches to stop hypertension
EPIC –PANACEA: European prospective investigation into cancer and nutrition – physical activity, nutrition, alcohol, cessation of smoking, eating out of home in relation to anthropometry
FDA: Food and drug administration
GRADE: Grading of Recommendations Assessment, Development, and Evaluation
HPFS: Health professionals follow‐up study
MD: Mean differences
MIDs: Minimally important differences
MOOSE: Meta‐analyses of observational studies in epidemiology
MUFAs: Monounsaturated fatty acids
NHS II: Nurses' health study II
NHS: Nurses' health study
NOS: Newcastle‐Ottawa scale
PICOTS: Participants, interventions/exposures, comparators, outcomes, time, setting
PREDIMED: PREvención con DIeta MEDiterránea
PRISMA: Preferred reporting items for systematic reviews and meta‐analyses
PUFAs: Polyunsaturated fatty acids
RCTs: Randomized controlled trials
RR: Relative risks
SD: Standard deviation
SMD: Standardized mean differences
SUN: Seguimiento Universidad de Navarra study

## 1 INTRODUCTION

Obesity remains a serious unmet public health concern, especially as it has been identified during our current coronavirus pandemic circumstances as one of the strongest risk factors for COVID‐19 morbidity and mortality.^1^ Having increased adiposity is also a major driver of diabetes and cardiovascular disease. Over the past three decades, the body of evidence from epidemiologic studies and controlled trials has grown supporting the consumption of nuts for cardiometabolic health benefits, such as diabetes,^2^ metabolic syndrome,^3^ and cardiovascular disease.^4^, ^5^ Accordingly, major dietary guidelines,^6^, ^7^, ^8^, ^9^, ^10^ as well as clinical practice guidelines for diabetes and heart disease, have recommended nuts alone or as part of dietary patterns such as the Mediterranean, Portfolio, vegetarian/plant‐based, and Dietary Approaches to Stop Hypertension (DASH) dietary patterns for diabetes and cardiovascular health.^11^, ^12^, ^13^, ^14^, ^15^ Despite these recommendations, concerns persist that nuts may contribute to weight gain due to their high energy density.^6^ With the rise in overweight and obesity and its downstream cardiometabolic complications, cardiovascular and diabetes associations and recommendations have cautioned against the over consumption of nuts at the same time that they recommend them, at doses ranging from approximately 1 to 1.5 ounces per day (~28 to 42.5 g/day), for cardiovascular disease prevention.^14^, ^16^, ^17^, ^18^, ^19^ Even though the prevalence of nut intake has gradually increased over the past decade, predominately in middle to high‐income economies, the intake levels have remained well below guideline recommendations.^20^ One of the barriers to increasing the consumption of nuts is the perception that they may contribute to weight gain more than other “healthy foods” owing to their high energy density.^21^, ^22^, ^23^, ^24^ Based on their macronutrient composition and Atwater factor kilocalorie determinations, tree nuts and peanuts are high in fat providing more than 40% of their total energy content, ranging from ~44% in pistachios and cashews to ~76% in macadamia,^25^, ^26^, ^27^, ^28^, ^29^, ^30^, ^31^, ^32^, ^33^, ^34^ and hence, there is concern that this leads to high caloric intake.

Whether nut intake at or above recommended levels contributes to weight gain and leads to obesity, and if this is affected by subsets of populations, nut intervention characteristics or study traits are unclear. Previous syntheses of the evidence have assessed the best evidence from prospective cohort studies^35^ and randomized controlled trials (RCTs).^36^, ^37^ While these two lines of evidence failed to show an adverse signal of nuts, the data in prospective cohort studies and several important adiposity outcomes in RCTs were not meta‐analyzed and dose–response relationships and the certainty of evidence were not assessed.

To address these knowledge gaps, a series of systematic reviews and meta‐analyses were conducted assessing global and abdominal measures of adiposity in prospective cohorts and RCTs with an assessment of the certainty of the evidence using the Grading of Recommendations Assessment, Development, and Evaluation (GRADE) approach.

## 2 METHODS

These systematic reviews and meta‐analyses followed the Cochrane Handbook for Systematic Reviews of Interventions.^38^ Results are reported in accordance with the Meta‐analyses of Observational Studies in Epidemiology (MOOSE) guidelines for the analysis of prospective cohorts^39^ (Table S1) and the Preferred Reporting Items for Systematic Reviews and Meta‐Analyses (PRISMA) guidelines for controlled trials^40^ (Table S2). The protocol is registered at ClinicalTrials.gov (identifier, NCT02654535).

### 2.1 Study selection

MEDLINE, EMBASE, and the Cochrane Central Register of Controlled Trials were searched from inception through August 12, 2019. The full set of terms used for the search strategy is available in Table S3. Manual searches of the reference lists of included studies supplemented electronic searches. Table S4 provides the PICOTS framework of the search strategy.

Briefly, search terms encompassed those specifying the exposure and outcomes. The exposure included tree nuts (one‐seeded fruit in a hard shell, including almonds, Brazil nuts, cashews, hazelnuts, macadamia nuts, pecans, pine nuts, pistachios, and walnuts) and peanuts (technically a member of the legume family but sharing a similar nutritional profile with tree nuts), herein referred to collectively as “nuts.” Outcomes were measures of adiposity, including, but not limited to overweight, obesity, body weight, body mass index (BMI), and waist circumference. Reports were included if they were a prospective cohort or RCT investigating nut consumption on adiposity related outcomes in adults (men and nonpregnant, nonbreastfeeding women ≥18 years). For prospective cohort studies the duration had to be at least 1 year, and for the trials the intervention had to be given in a randomized manner for at least 3 weeks in comparison with a control. No language restrictions were applied. Reports were excluded if they did not include consumption of the whole nut or nut butters (i.e., nut oils or extracts), were not done in humans, or did not provide suitable endpoint data. When multiple publications existed for the same study, the article with the most applicable information and longest duration was included.

### 2.2 Data extraction

Two reviewers (SKN and EV or SBM) independently reviewed and extracted relevant data from each report, including study design, blinding, sample size, participant characteristics, follow‐up duration, intervention (nut type and dose), comparator diet, macronutrient profile, funding source, and outcome data using standardized proformas. Where data were presented in a language other than English, the assistance of a translator was utilized. Where data were included in figures and not provided numerically, data were extracted using the software program Plot Digitizer V.2.6.8.^41^ Missing information for any endpoint or study details were requested from the authors of all included studies and published abstracts where applicable. Disagreements in data extracted were resolved by consensus.

### 2.3 Outcomes

The primary outcomes were incidence of overweight or obesity in prospective cohort studies and body weight in RCTs. Secondary outcomes included markers of global adiposity (body weight [prospective cohort studies only], BMI, and body fat percentage) and abdominal adiposity (waist circumference, waist‐to‐hip ratio, and visceral adipose tissue). Change from baseline differences was preferred over end differences and expressed as mean ± standard deviation (SD). When not provided, between treatment differences in change‐from‐baseline or end differences were calculated by subtracting means (Mean differences [MDs]), and SDs were calculated from the available data using published formulas.^38^

### 2.4 Risk of bias assessment

Risk of bias for each included cohort and trial was assessed by two independent reviewers with differences resolved by consensus. The Newcastle–Ottawa Scale (NOS) was used to assess the risk of bias in prospective cohorts and the Cochrane Risk of Bias tool was used to assess RCTs.^42^, ^43^

The NOS for prospective cohorts is a rating scale which awards points based on cohort selection, adequacy of outcome measures, and comparability of cohorts regarding design or analysis.^42^ A maximum of 9 points may be awarded, with a score of 6 or more being considered higher quality.

For the Cochrane Risk of Bias tool for RCTs, the assessment was done across five domains of bias (sequence generation, allocation concealment, blinding, incomplete outcome data, and selective reporting). The risk of bias was assessed as low (proper methods taken to reduce bias), high (improper methods creating bias), or unclear (insufficient information provided to determine the bias level).^43^

### 2.5 Data synthesis and analysis

Data analyses were conducted using Review Manager (RevMan) V.5.3 (Copenhagen, Denmark: The Nordic Cochrane Centre, The Cochrane Collaboration, 2014) and Stata V.16 (College Station, TX, StataCorp LP).

Risk ratios (RRs) and MDs were pooled, as applicable, for the prospective cohort studies comparing highest versus lowest dose categories from the most adjusted models for nut consumption and MDs for the RCTs using the generic inverse variance method. Standardized mean differences (SMD) were utilized to standardized results to a uniform scale, such as for studies where the outcome was measured using different methods and nonconvertible units and for summary forestplots of the pooled effect estimates. Random‐effects DerSimonian–Laird models^44^ were used even in the absence of statistically significant between‐study heterogeneity, as they yield more conservative summary effect estimates in the presence of residual heterogeneity. Fixed‐effects models were only used where there were <5 included studies as there is too little information to estimate *τ* ^2^ reliably.^45^ Paired analyses were applied for crossover trials.

If nut intake was reported as servings per period of time, it was converted into grams per day using 28 g as equivalent to one serving in the prospective cohort studies.^6^ The assigned dose was considered as the mean consumption in each quantile of nut consumption. If the assigned doses were not reported, the mean dose was approximated for each quantile using the midpoint of the lower and upper bounds, with “never/almost never” being considered as 0 g/day. If the lowest and highest dose categories of a study were ≤ and ≥, respectively, the equivalent value was considered the dose of the category. When cohort person‐year per category was not available, categories were regarded as equal in size and follow‐up, and the case number per category was obtained by Bekkering's method.^46^ Data were expressed as RRs or MDs with 95% confidence intervals (CI) for the prospective cohort studies and MDs with 95% CIs for the RCTs.

Dose–response meta‐regression analyses were conducted to explore the relationship between nut dose (g/day) and all outcomes. Continuous linear dose–response gradients were assessed using meta‐regression and nonlinear dose–response thresholds using spline curve modeling by MKSPLINE or a fractional polynomial procedure. Categorical dose–response analyses were assessed at the median intake level of ≥45.5 g/day, which is comparable to the qualified health claim dose ≥42.5 g/day (based on the Food and Drug Administration [FDA] amount noted in the cardiovascular disease risk reduction claim^17^).

Interstudy heterogeneity was assessed by the Cochran Q statistic and quantified by the *I* ^2^ statistic, where *I* ^2^ ≥ 50% and *P* < 0.10 was considered evidence of substantial heterogeneity. Sources of heterogeneity were explored using sensitivity and subgroup analyses. Sensitivity analyses were performed in which each individual study (prospective cohort study or RCT) was removed from the meta‐analyses and the effect size recalculated with the remaining studies to determine whether a single study exerted an undue influence on the overall results. Sensitivity analyses were also undertaken using correlation coefficients of 0.25, 0.50, and 0.75 for paired analyses of crossover trials and fixed‐effects meta‐analyses to determine whether the overall results were robust to the use of different models. A priori subgroup analyses (categorical and continuous) were conducted, using meta‐regression if ≥10 studies per outcome were available, for nut type, nut dose, feeding control, comparator, energy balance, study design, duration of follow‐up, health status, and risk of bias. Post hoc subgroup analyses were conducted for trials by weight goal (weight loss and weight maintenance), energy intake, and funding source. Categorical subgroup analyses were carried out using median values where applicable. Post hoc analyses for the prospective cohorts were conducted using the unadjusted models, specifically models not adjusting for energy intake, where applicable.

Publication bias was assessed, if ≥10 studies were available, by visual inspection of funnel plots and formal testing with the Egger^47^ and Begg^48^ tests. If publication bias was suspected, adjustment for funnel plot asymmetry was done by imputing missing study data using the Duval and Tweedle trim‐and‐fill method.^49^

### 2.6 Grading of the evidence

The GRADE approach was used to assess the certainty of the evidence.^50^ The evidence was graded as “high,” “moderate,” “low,” or “very low” certainty. Prospective cohort studies start as “low” certainty and RCTs as “high” certainty of evidence by default and can be downgraded or upgraded further based on prespecified criteria. Criteria to downgrade evidence include risk of bias (weight of reports show risk of bias as assessed by NOS < 6 for prospective cohort studies or Cochrane Risk of Bias tool for trials), inconsistency (substantial unexplained inter‐study heterogeneity, *I* ^2^ > 50%; *P* < 0.10), indirectness (limited generalizability of the findings), imprecision (the 95% CI for estimates are wide, crossing prespecified minimally important differences [MIDs]), and publication bias (evidence of small‐study effects). Criteria to upgrade evidence include a large magnitude of association, dose–response gradient, and attenuation of the pooled‐risk estimate by plausible confounders.^50^

## 3 RESULTS

### 3.1 Search results

**FIGURE 1:**
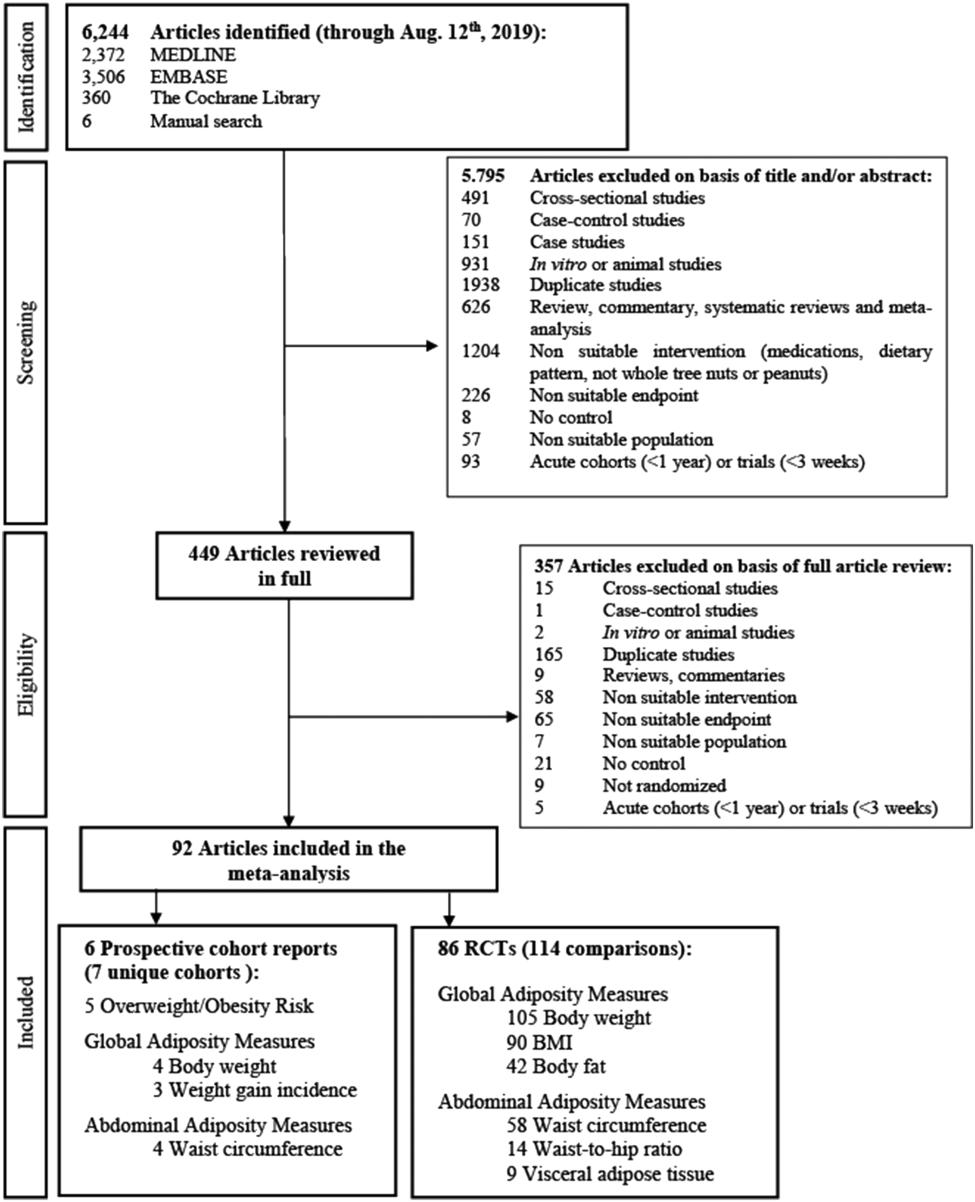
Summary of evidence search and selection

Figure 1 shows the flow of the literature. The search identified 6244 reports, of which 5795 were excluded based on review of title and abstract. The remaining 449 reports were retrieved and reviewed in full, of which 357 were excluded. A total of 92 reports containing data from 7 unique prospective cohorts (6 reports) involving 569,910 participants^51^, ^52^, ^53^, ^54^, ^55^, ^56^ and 86 RCTs involving 114 trial comparisons involving 5873 participants^57^, ^58^, ^59^, ^60^, ^61^, ^62^, ^63^, ^64^, ^65^, ^66^, ^67^, ^68^, ^69^, ^70^, ^71^, ^72^, ^73^, ^74^, ^75^, ^76^, ^77^, ^78^, ^79^, ^80^, ^81^, ^82^, ^83^, ^84^, ^85^, ^86^, ^87^, ^88^, ^89^, ^90^, ^91^, ^92^, ^93^, ^94^, ^95^, ^96^, ^97^, ^98^, ^99^, ^100^, ^101^, ^102^, ^103^, ^104^, ^105^, ^106^, ^107^, ^108^, ^109^, ^110^, ^111^, ^112^, ^113^, ^114^, ^115^, ^116^, ^117^, ^118^, ^119^, ^120^, ^121^, ^122^, ^123^, ^124^, ^125^, ^126^, ^127^, ^128^, ^129^, ^130^, ^131^, ^132^, ^133^, ^134^, ^135^, ^136^, ^137^, ^138^, ^139^, ^140^, ^141^, ^142^ met eligibility criteria and were included in the analyses. Authors from seven trials provided additional data for inclusion in the syntheses.^68^, ^71^, ^75^, ^78^, ^102^, ^142^, ^143^

### 3.2 Study characteristics

Tables 1 and S5 show the characteristics of the included prospective cohort studies. The studies came from the United States^52^, ^55^, ^56^ and 10 countries in Europe.^51^, ^53^, ^54^ The median age was 48.1 years (range 37.3 to 55.0 years). Median follow‐up was 18 years (range 2.3 to 24 years). Dietary intake assessments were performed with validated semiquantitative food frequency questionnaires in all studies, while one cohort used a combination of semiquantitative food frequency questionnaire and/or validated 7‐day food record depending on the country.^54^ Median nut intake at baseline in the highest quantiles of consumers was estimated at 7 g/day ranging from 3 to 28 or more g/day. Ascertainment of adiposity measures was self‐reported in all but one cohort^54^ where body weight and height were measured by the study center. All studies were funded by agency alone, except one report which was funded by agency and agency‐industry.

**TABLE 1:** Summary of characteristics of prospective cohort studies assessing the association between tree nuts and peanut intake and overweight/obesity risk and measures of adiposity

Cohort characteristic^a^	
Number of cohorts	7
Participants^b^	569,910
Males:Females (%)	26:74
Age (years) (range)	48.1 (37.3 to 55.0)
Baseline body weight (kg)^c^	68.0 (62.7 to 83.0)
Baseline BMI (kg/m^2^)^d^	24.7 (18.1 to 26.0)
Follow‐up duration (years) (range)	18.0 (2.3 to 24.0)
Setting (frequency)	
Europe	3
United States	4
Nut types (frequency)	
Tree nuts and peanuts	6
Walnuts, almonds, hazelnuts, peanuts	1
Nut dose (g/day)	7 (3 to 28)

^a^ Median, unless otherwise indicated.

^b^ When multiple reports of the same cohort were present, the total number of participants was calculated using the number from the cohort report with the largest number of participants as an effort to avoid double counting.

^c^ 4/7 cohorts reported baseline body weight (kg).

^d^ 6/7 cohorts reported baseline BMI (kg/m^b^).

Table S6 shows the confounding variables included in the most adjusted models for each of the included prospective cohort studies. The median number of variables in the most adjusted models was 11 (range 7–20) based on the 5 reports where information was available. Fifty percent of the cohort reports adjusted for the confounding variable of energy intake.

Tables 2 and S7 show the characteristics of the included RCTs. All RCTs were conducted in outpatient settings where noted, with the majority (43.9%) conducted in the United States. Trials had a median follow‐up duration of 8 weeks (range: 3 to 104 weeks), a slightly higher distribution of women (58%) compared to men (42%), and more than half used a parallel design (54/86 trials). Most of the trials recruited participants with overweight or obesity (34 trial comparisons); there was also representation from participants free from chronic disease (26 trial comparisons) and other cardiometabolic health conditions. Median baseline (range) values for body weight and BMI were 81.0 kg (49.7 to 111.2 kg) and 29.2 kg/m^2^ (19.9 to 38.4 kg/m^2^), respectively.

**TABLE 2:** Summary of characteristics of randomized controlled trials assessing the association between nut intake and measures of adiposity (continued on next page)

Trial characteristic^a^		
Number trials (unique reports: comparisons)	86:114	
Trial size (range)	49 (9 to 317)	
Study design (C:P) (%)^b^	37:63	
Setting (IP:OP:NR) (%)	0:99:1	
Follow‐up duration (weeks) (range)	8 (3 to 104)	
Male:Female (%)^c^	41:59	
Age (years)^d^	50 (18 to 69.3)	
Baseline body weight (kg)^e^	81.0 (49.7 to 111.2)	
Baseline BMI (kg/m^2^)^f^	29.2 (19.9 to 38.4)	
Health status (frequency)		
Dyslipidemia	13	
Healthy	26	
Overweight/obese	34	
Diabetes	23	
Metabolic syndrome	13	
Coronary heart disease	2	
Multiple	4	
Country (frequency)		
Australia	8	
Brazil	5	
Canada	4	
China	4	
France	1	
Germany	1	
India	3	
Iran	4	
Israel	1	
Italy	5	
Japan	2	
Korea	4	
Multiple countries	1	
New Zealand	6	
Pakistan	2	
South Africa	2	
Spain	7	
Sweden	1	
Taiwan	1	
Turkey	1	
United States	50	
Not reported	1	
**Trial characteristic** ^a^	
Nut type (frequency)		
Almonds	33	
Brazil nut	1	
Cashew nut	4	
Hazelnut	6	
Macadamia	3	
Mixed nuts	10	
Nuts, undefined	3	
Peanuts	6	
Pecans	3	
Pistachios	13	
Walnuts	32	
Nut dose (g/day) (range)	45.5 (5 to 100)	
Intervention type (frequency)		
Metabolically controlled	16	
Controlled feeding	2	
Supplemented	88	
Dietary advice	5	
Not reported	3	
Energy balance (frequency)		
Negative	15	
Neutral	80	
Positive	8	
Not reported	11	
Comparator (frequency)		
Carbohydrate	28	
Fat	20	
Protein	4	
Mixed macronutrient	43	
No nuts	19	
Designed for weight maintenance (frequency)		
Yes	34	
No	73	
Not reported	7	
Funding source (%)		
Agency	18.4	
Agency‐industry	29.0	
Industry	39.5	
None reported	13.2	

Abbreviations: BMI, body mass index; C, crossover; IP, inpatient; N, number; NR, not reported; OP, outpatient; P, parallel.

^a^ Median, based on the 114 trial comparisons, unless otherwise indicated.

^b^ Based on the 86 trial reports. This value did not significantly differ from trial comparisons (34:66).

^c^ 111/114 trial comparisons provided data on sex.

^d^ 108/114 trial comparisons provided data on baseline age.

^e^ 98/114 trial comparisons provided data on baseline body weight.

^f^ 101/114 trial comparisons provided data on baseline BMI.

#### 3.2.1 Risk of bias

Table S8 shows the NOS risk of bias assessments for the included prospective cohort studies. Three of the 6 prospective cohort reports were scored as ≥6 on the NOS scale, denoting high‐quality studies. Overall, there was evidence of serious risk of bias across the studies.

Figure S1 shows the Cochrane Risk of Bias assessments for the included RCTs, with the overall risk of bias proportions presented in Figure S2. The majority of trial comparisons were judged as having unclear or low risk of bias across domains. Overall, there was no evidence of serious risk of bias across the studies.

#### 3.2.2 Association of nut intake with incident overweight/obesity and measures of global adiposity

**FIGURE 2:**
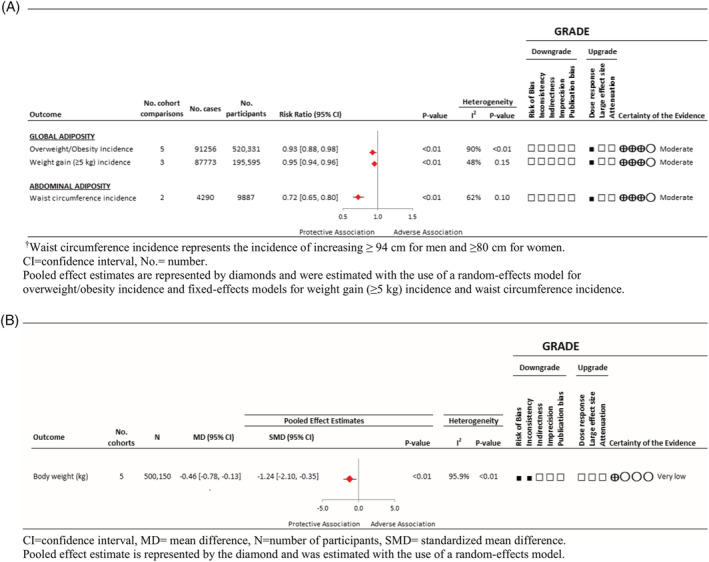
(A) Summary of the pooled effect estimates of prospective cohort studies assessing the association between tree nut and peanut intake and adiposity incidence outcomes, with GRADE assessment. (B) Summary of the pooled effect estimates of prospective cohort studies assessing the association between tree nut and peanut intake and body weight change outcome, with Grading of Recommendations Assessment, Development, and Evaluation (GRADE) assessment

Figures 2A,B and S3–S5 show the association of nut consumption with incident overweight/obesity and measures of global adiposity in five prospective cohort studies involving 520,331 participants. Higher nut intake was associated with a decrease in the primary outcome of overweight/obesity incidence (RR 0.93 [95% CI 0.88 to 0.98], *P* < 0.01; substantial heterogeneity, *I* ^2^ = 90.0%, P‐heterogeneity < 0.01). Similarly, higher nut consumption was associated with weight loss (MD −0.46 kg [95% CI −0.78 to −0.13 kg], *P* < 0.01; substantial heterogeneity, *I* ^2^ = 95.9%, P‐heterogeneity < 0.01) and reduced risk of weight gain ≥ 5 kg (RR 0.95 [95% CI, 0.94 to 0.96], *P* < 0.01; no substantial heterogeneity, *I* ^2^ = 46.7%, P‐heterogeneity = 0.15). Pooled analyses from the least adjusted models (i.e., models not adjusting for energy intake) assessing body weight change indicate that higher nut consumption was associated with weight loss (MD −0.64 kg [95% CI −1.12 to −0.15 kg]). Only one cohort provided data from unadjusted models for overweight/obesity incidence and risk of weight gain ≥ 5 kg, indicating no association with higher nut consumption.^51^

#### 3.2.3 Association of nut intake with measures of abdominal adiposity

Figures 2A and S6 show the association of nut intake with measures of abdominal adiposity in 2 prospective cohorts involving 1297 participants. Nut intake was associated with a lower risk of an elevated waist circumference ≥94 cm for men and ≥80 cm for women. (RR 0.72 [95% CI 0.65 to 0.80]; *P* < 0.01; no substantial heterogeneity, *I* ^2^ = 62.3%, P‐heterogeneity = 0.10).

#### 3.2.4 Effect of nut intake on body weight and measures of global adiposity

**FIGURE 3:**
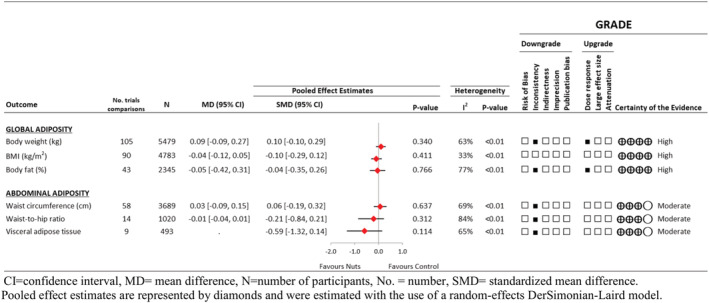
Summary of the pooled effect estimates of randomized controlled trials assessing the association between tree nut and peanut intake and adiposity outcomes, with Grading of Recommendations Assessment, Development, and Evaluation (GRADE) assessment

Figures 3 and S7–S9 show the effect of nuts on markers of global adiposity in the RCTs. There was no effect of nuts compared with control on the primary outcome body weight (105 trial comparisons involving 9655 participants, MD 0.09 kg, [95% CI −0.09 to 0.27 kg], *P* = 0.340; substantial heterogeneity, *I* ^2^ = 63.2%, P‐heterogeneity < 0.01). No effect was also seen on BMI (90 trial comparisons involving 4783 participants, MD −0.04 kg [95% CI −0.12 to 0.05 kg], *P* = 0.411; substantial heterogeneity, *I* ^2^ = 32.7%, P‐heterogeneity < 0.01) or body fat (14 trial comparisons involving 2345 participants, MD −0.05% [95% CI −0.42 to 0.31%], *P* = 0.77; substantial heterogeneity, *I* ^2^ = 77.04%, P‐heterogeneity < 0.01).

#### 3.2.5 Effect of nut intake on measures of abdominal adiposity

Figures 3 and S10–S12 show the pooled effect estimates for the markers of abdominal adiposity in the RCTs. There was no effect of nuts compared with control on waist circumference (58 trial comparisons involving 3689 participants, MD 0.03 cm [95% CI −0.09 to 0.15 cm], *P* = 0.637; substantial heterogeneity, *I* ^2^ = 69.7%, P‐heterogeneity < 0.01), waist‐to‐hip ratio (14 trial comparisons involving 1,020 participants, MD −0.01 [95% CI −0.04 to 0.01], *P* = 0.312 substantial heterogeneity, *I* ^2^ = 84.1%, P‐heterogeneity < 0.01), or visceral adipose tissue (9 trial comparisons involving 493 participants, SMD −0.59 [95% CI −1.32 to 0.14], *P* = 0.114; substantial heterogeneity, *I* ^2^ = 64.7%, P‐heterogeneity < 0.01).

### 3.3 Dose response analyses

Figure S13 shows the continuous linear and nonlinear dose response analyses in the prospective cohort studies. There was evidence of an inverse linear dose–response gradient for overweight/obesity, weight gain (≥5 kg), and waist circumference incidence (*P* < 0.05). With evidence of a nonlinear dose–response threshold for overweight/obesity incidence with a decrease until 4 g/day followed by a plateau and for waist circumference incidence, there was no significant effect up to 17 g/day with a reduction at 18 g/day onward (*P* > 0.05). Categorical dose response analyses were not undertaken for the prospective cohorts, as <10 studies were available for analyses.

Table S9 and Figure S14 show the continuous linear and nonlinear dose response analyses and Figures S15–S19 show the categorical dose response analyses in the RCTs. Continuous analyses showed that higher nut doses were associated with reductions in body weight (*ß* −0.012 [95% CI −0.024 to −0.001], *P* = 0.04) and body fat (*ß* −0.035 [95%CI −0.058 to −0.013], *P* < 0.01).

### 3.4 Sensitivity analyses

Table S10 and Figures S20–S23 show the sensitivity analyses for the prospective cohort studies. For overweight/obesity incidence, the removal of the Adventist Health Study‐2 (AHS‐2)^52^ involving >50% vegetarian participants, reduced the heterogeneity from substantial to nonsubstantial (*I* ^2^ = 14%, P‐heterogeneity = 0.32) without altering the direction, significance, or magnitude of the pooled‐risk estimate (RR 0.96 [95% CI 0.95 to 0.98], *P* < 0.001). No other sensitivity analyses altered the direction, significance, or magnitude of the pooled‐risk estimates or the evidence of heterogeneity. In the sensitivity analyses where fixed effects models were applied to the outcomes of overweight/obesity incidence and body weight change and where random‐effects models were applied to the outcomes of weight gain (≥5 kg) incidence and waist circumference incidence, the direction, magnitude, and significance of the pooled estimates remained comparable to those produced by the original models applied.

Tables S11 and S12 and Figures S24–S29 show the sensitivity analyses for the RCTs. In the sensitivity analysis of visceral adipose tissue, one trial^79^ was influential in that its removal nonsignificantly altered the magnitude of the pooled effect in the remaining trials by >10% and reduced the heterogeneity to no longer be significant (*I* ^2^ = 0%, P‐heterogeneity = 0.473). Sensitivity analyses using correlation coefficients of 0.25 and 0.75 for paired analyses of crossover trials in the meta‐analyses of RCTs also did not significantly modify the results. In the sensitivity analyses where fixed effects models were applied, the direction, magnitude, and significance of the pooled estimates remained comparable to those produced by the random‐effects models, with the exception of a significant reduction observed in body weight (MD −0.19 kg [95% CI −0.24 to −013 kg] *P* < 0.01; *I* ^2^ = 63.2%, P‐heterogeneity<0.01) and significant reductions with nut consumption observed in body fat (MD −0.13% [95% CI −0.24 to −0.01%], *P* = 0.03; *I* ^2^ = 77%, P‐heterogeneity<0.01) and waist‐to‐hip ratio (MD −0.02 [95% CI −0.03 to −0.01], *P* = <0.01; *I* ^2^ = 84%, P‐heterogeneity<0.01) in the RCTs.

### 3.5 Subgroup analyses

We did not conduct a priori subgroup analyses for any outcomes in the prospective cohort studies, as <10 studies were available for analyses.

Figures S15–S19 and S30–S34 show the categorical a priori and post hoc subgroup analyses in the RCTs. Subgroup analyses were not conducted for visceral adipose tissue, as <10 studies were available for analyses. The categorical analyses indicated a significant effect modification by nut type for body weight (higher for walnuts and peanuts), for body fat (higher for macadamia), for waist circumference (lower for almonds, Brazil nuts; higher for pistachios), feeding control for body fat (higher for dietary advice), comparator for body fat (higher for no nuts), energy balance for body weight (higher for positive or not reported), BMI (lower for not reported and higher for neutral), waist‐to‐hip ratio (lower for negative energy balance), design for waist circumference (higher for cross‐sectional); health status for BMI (higher in participants with prediabetes), waist circumference (lower in participants who have overweight/obesity; higher for participants who are healthy or have prediabetes), intended weight maintenance for waist circumference (lower for reports where weight maintenance was not reported), and funding source for BMI and waist circumference (higher for those with industry funding) (*P* < 0.05). None explained the evidence of heterogeneity. Exploratory analyses comparing location where the trial was conducted by continent, as well as characteristics of the nut interventions, specifically whether they were salted, unsalted or mixed and roasted, raw or mixed showed no differences (data not shown).

### 3.6 Publication bias

Publication bias was not assessed for any outcomes in the prospective cohort studies, as <10 studies were available for analyses.

Figure S35 shows the funnel plots for body weight, BMI, body fat, waist circumference, and waist‐to‐hip ratio. No evidence of publication bias was seen for BMI, body fat, and waist‐to‐hip ratio. There was evidence of small‐study effects for body weight by the Egger's test and waist circumference for the Begg's test (*P* < 0.05). Figure S36 shows the investigation of these effects by Trim‐and‐Fill analysis indicated no meaningful change to effect estimates.

### 3.7 GRADE assessment

Table S13 shows that for the prospective cohort meta‐analyses, the overall certainty of the evidence for the association of nut consumption was graded as “moderate” for overweight/obesity incidence, weight gain (≥5 kg) incidence, waist circumference incidence of increasing ≥94 cm for men and ≥80 cm for women owing to an upgrade for dose–response and “very low” for body weight change owing to downgrades for inconsistency and risk of bias.

Table S14 shows the certainty of evidence for meta‐analyses of RCTs was graded as “high” for BMI, as well as “high” for body weight, body fat owing to a downgrade for inconsistency and an upgrade for a dose–response gradient, and “moderate” for waist circumference, waist‐to‐hip ratio, and visceral adipose tissue owing to a downgrade for inconsistency (*I* ^2^ > 50%, P‐heterogeneity <0.01).

## 4 DISCUSSION

The present systematic review and meta‐analysis of nut consumption and adiposity involving six prospective cohort studies and 86 RCTs (114 trial comparisons) did not illustrate an increased risk of overweight/obesity or raise other measures of adiposity studied in adults.

Based on the long‐term findings from the prospective cohort studies, a significant inverse association was observed across outcomes assessed. These findings align with those proposed by the systematic review of prospective studies by Eslami and colleagues.^35^ Suggesting that nut consumption may have a protective effect on risk of adiposity accumulation. This is further supported by the results of the present aggregate analyses from the RCTs, which showed a lack of a causal effect of nut consumption on the reported measures of adiposity. Previous systematic reviews and meta‐analyses of trials involved differing inclusion and exclusion criteria yet showed similar findings in regard to a lack of effect of nut consumption on body weight, BMI, or waist circumference.^36^, ^37^ The lack of effect of nut consumption on waist circumference is further supported by Blanco Mejia and colleagues in their systematic review and meta‐analysis assessing nuts and metabolic syndrome.^3^

Significant heterogeneity in the current analysis did exist. While this heterogeneity could not be adequately assessed categorically for the cohorts as there were too few cohort studies, subgroup analyses and meta‐regression of the trials identified potential sources of heterogeneity. For the trials, similar to the previous publications,^36^, ^37^ energy balance was identified as a potential source of heterogeneity. However, in the current analysis, incorporating nuts into a dietary pattern involving an overall negative energy balance compared to a negative energy balance without nuts was observed to favour nuts in regard to not increasing body weight, BMI, or waist‐to‐hip ratio. Inclusion of nuts as a part of a dietary pattern without concern for increased body weight or adiposity measures is further supported by findings from the PREDIMED trial, where inclusion of nuts as part of a Mediterranean dietary pattern saw slightly reduced body weight and adiposity measures with no significant differences when compared with the Mediterranean dietary pattern with olive oil or the low fat dietary pattern.^144^ A sensitivity analysis involving the inclusion of the PREDIMED trial did not significantly affect the magnitude or direction of the current findings. In addition to energy balance, nut dose was detected as a potential effect modifier of body weight and body fat, where greater reductions were observed with increasing nut dose. In categorical analyses, nut doses ≥45.5 g/day indicated lower adiposity measures compared to lower doses. As nut doses of 1 to 1.5 ounces (~28 to 42.5 g) per day are often noted in dietary guidelines, as well as the FDA qualified health claim for coronary heart disease risk reduction, this suggests the provision often seen following nut recommendations, as well as stated at the end of the applicable qualified health claims asserting “see nutrition information for fat [and calorie] content” with the implied message that foods high in fat and calories lead to increased adiposity may be unwarranted.^17^, ^18^, ^19^ Likewise, continuous linear meta‐regression identified dose‐dependent relationships between nut consumption with both body weight and body fat, where nut dose was inversely correlated with body weight and fat. However, significant departures from linearity were observed in BMI, waist circumference, and waist‐to‐hip ratio, where the maximum protective dose appeared to be around 50 g/day based on waist‐to‐hip ratio. Although the waist‐to‐hip ratio may have been confounded by the nonsignificant positive correlation observed between waist circumference and nut consumption. This positive association between nut consumption and waist circumference differs from findings in the literature, where nut and seed consumption has been associated with significantly decreased pericardial fat, and trends toward decreased visceral fat,^145^ and monounsaturated fat intake, which is prevalent in nuts, compared to carbohydrate intake has been shown to prevent central fat redistribution.^146^

### 4.1 Strengths and limitations

Strengths of the present systematic review and meta‐analysis include its comprehensive design, comprising both prospective cohort studies and RCTs, using the GRADE approach to evaluate the certainty of evidence. The prospective cohort studies provide assessment of nut consumption over the long term in a large sample of participants in free‐living conditions in relation to adiposity. The design of RCTs provides the best protection against bias; there were also a substantial number of trials identified (106 trial comparisons) for the primary outcome of body weight; the median follow‐up period was 8 weeks, which allows for the assessment of a moderate duration of intervention. In addition, the meta‐regression and subgroup analyses provide further insight as to various factors that have previously been hypothesized to influence the impact of nut consumption on adiposity.

These analyses are not without limitations. For the prospective cohort studies, we downgraded the certainty of the evidence for serious inconsistency in the estimates across the studies for body weight change as there was evidence of unexplained heterogeneity (92%). Although the inconsistency may have related to measurement error as there was a lack of repeated measurement of intake of nuts, use of a food frequency questionnaire measure that was not specifically validated for nut intake, and adiposity measures were mainly self‐reported by participants. Risk of bias was also observed for body weight change as participants were primarily comprised of well‐educated individuals, many of whom were health professionals, including university graduates from SUN and health professionals recruited from NHS, NHS II, and HPFS, and thus may not be generalizable to other populations.

For the RCTs, we downgraded the certainty of evidence for serious inconsistency in the estimates due to unexplained heterogeneity in all the outcomes assessed, except BMI. Subgroup analyses indicated potential sources of heterogeneity; however, this was often observed when the covariate was unevenly distributed, as well as the differences in treatment effects between subgroups are unlikely to otherwise alter clinical decisions.

Weighing these strengths and limitations using GRADE, the certainty of evidence ranged from “very low” to “high.” A reason for the “very low” certainty of evidence observed is due to the GRADE approach starting observational studies at “low” certainty. Overall, the prospective cohort studies showed mostly “moderate” and the RCTs showed equally “high” and “moderate” certainty of evidence.

### 4.2 Potential mechanisms of action

There are several biological mechanisms which may explain the association, more specifically, the lack of association observed between nut consumption with overweight/obesity risk and other measures of adiposity, including: (1) unsaturated fatty acid content, (2) satiating effect, and (3) physical structure, each in a way associated with the bioavailability of nuts when consumed. Nuts are rich in unsaturated fatty acids (monounsaturated fatty acids [MUFAs] and polyunsaturated fatty acids [PUFAs]), which are suggested to be more readily oxidized^147^ and have a greater thermogenic effect^148^ compared to saturated fatty acids, leading to less fat accumulation. Nuts are also rich in protein and fiber and dietary components associated with increased satiety.^149^, ^150^, ^151^ In addition to the protein and dietary fiber content of nuts, the physical structure may also contribute to their satiating effect since the mastication process involved in mechanically reducing nuts to a particle size small enough to swallow activates signaling systems that may modify appetite sensations.^152^ The physical structure of nuts may also contribute to fat malabsorption due to the fat content in nuts being contained within walled cellular structures that are incompletely masticated and/or digested.^153^, ^154^, ^155^, ^156^ Thus, due to these biological mechanisms which may be associated with decreased bioavailability, the Atwater Factor, a system for determining the energy value of foods which was founded over a century ago, associated with nuts, may overestimate the calories obtained by the body from nut consumption by approximately 16% to 25% depending on the nut type and form.^157^, ^158^, ^159^ This may potentially explain the present findings of a protective effect of nut consumption on measures of adiposity.

### 4.3 Practical implications

Current clinical practice guidelines already suggest the incorporation of nuts for the improvement of glycemic control and cardiovascular risk factors; however, there are often qualifiers regarding their fat content and energy density.^14^, ^15^, ^16^ With overweight and obesity respectively affecting 39% and 13% of adults globally and increased adiposity being a modifiable risk factor for diabetes and cardiovascular diseases, body weight management is an important consideration in dietary and lifestyle recommendations.^160^ Evidence from this systematic review and meta‐analysis suggests that nuts may continue to be highlighted as a nutrient dense component of dietary patterns for their cardiometabolic benefits without concerns of an adverse effect on weight control. Nuts are currently recommended as part of the Mediterranean, Portfolio, and DASH dietary patterns, yet despite tree nut and peanut intake increasing over the past 10 years, intake worldwide remains low at an estimated 16.7 g/day with about 15.2 g being contributed by peanuts.^20^ This is far below current recommendations of 1 to 1.5 ounces per day (approximately 28.3 to 42.5 g/day).^6^, ^17^, ^18^, ^19^ Based on the median nut intake in the trials of the current analyses and FDA qualified health claims, a dose of 42.5 g/day of nuts could easily be integrated into a daily dietary pattern by incorporating them into meals and/or consuming them as snacks. Except for individuals with nut allergies, no increase in side effects compared with control groups was reported in any of the cohort studies or trials, suggesting that dietary patterns which incorporate nuts as a regularly consumed component are safe. Future research may further assess the impact of different varieties of nuts and formats in which they may be consumed and how they are incorporated into the diet.

## 5 CONCLUSION

Current evidence suggests that nut consumption does not lead to increased adiposity. Health professionals and dietary guidelines may recommend nuts, for those without allergies, for their cardiometabolic benefits without stipulations or concern of an adverse effect on weight control.

## SOURCE OF RESEARCH SUPPORT

**SKN** was funded by Ontario Graduate Scholarships, Peterborough K. M. Hunter Charitable Foundation Graduate Awards, Banting & Best Diabetes Centre‐Novo Nordisk Studentship, Nora Martin Fellowship in Nutritional Sciences, and a Dietitians of Canada Graduate Student Award. **EMC** was the recipient of the Lawson Family Chair in Microbiome Nutrition Research at the Faculty of Medicine, University of Toronto. **DJAJ** was funded by the Government of Canada through the Canada Research Chair Endowment. **JLS** was funded by a PSI Graham Farquharson Knowledge Translation Fellowship, Diabetes Canada Clinician Scientist award, CIHR INMD/CNS New Investigator Partnership Prize, and Banting and Best Diabetes Centre Sun Life Financial New Investigator Award.

## ROLE OF THE FUNDING SOURCE

This work was funded by operating funds provided through a PSI Graham Farquharson Knowledge Translation Fellowship and Banting & Best Diabetes Centre Sun Life Financial New Investigator Award. None of the sponsors had a role in any aspect of the present study, including design and conduct of the study; collection, management, analysis, and interpretation of the data and preparation, review, approval of the manuscript, or decision to publish.

## POTENTIAL CONFLICTS OF INTEREST

SKN serves as a volunteer member of the not‐for‐profit group Plant‐Based Canada as of 2019. EV served as a scientific advisor for New Era Nutrition from 2019 to 2020. SBM reports no competing interests. CWCK has received research support from the Advanced Foods and Materials Network, Agricultural Bioproducts Innovation Program through the Pulse Research Network, Agriculture and Agri‐Food Canada, Almond Board of California, Barilla, Calorie Control Council, CIHR, Canola Council of Canada, The International Tree Nut Council Nutrition Research & Education Foundation, Kellogg, Loblaw Companies Ltd., Pulse Canada, Saskatchewan Pulse Growers, and Unilever. He has received consultant fees from American Pistachio Growers, speaker fees from the Peanut Institute, Tate & Lyle and The WhiteWave Foods Company, and travel funding from Sabra Dipping Company, Tate & Lyle, International Tree Nut Council Research & Education Foundation, California Walnut Commission, Sun‐Maid, The Peanut Institute, General Mills, Oldways Foundation, and International Nut and Dried Fruit Council Foundation. He is on the Clinical Practice Guidelines Expert Committee for Nutrition Therapy of the European Association for the Study of Diabetes (EASD). He is a member of the International Carbohydrate Quality Consortium (ICQC), Secretary of the Diabetes and Nutrition Study Group (DNSG) of the EASD, and a Director of the Toronto 3D Knowledge Synthesis and Clinical Trials foundation. RPB has received research grants from Bunge Ltd., Arctic Nutrition, the Dairy Farmers of Canada, and Nestle Inc., as well as travel support from Mead Johnson and mass spectrometry equipment and support from Sciex. RPB is on the executive of the International Society for the Study of Fatty acids and Lipids and held a meeting on behalf of Fatty Acids and Cell Signaling, both of which rely on corporate sponsorship. AJH has received research support from the Dairy Farmers of Canada. JSS received financial support by ICREA under the ICREA Academia program; the SEMERGEN grant; Department of Health of the Government of Navarra (61/2015); the Fundació La Marató de TV (Ref. 201630.10); the AstraZeneca Young Investigators Award in Category of Obesity and T2D 2017 (D.R.); grants from the Consejería de Salud de la Junta de Andalucía (PI0458/2013; PS0358/2016; PI0137/2018); the PROMETEO/2017/017 grant from the Generalitat Valenciana, and the SEMERGEN grant; and grant of support to research groups 35/2011 (Balearic Islands Government; FEDER funds) (J.A.T.). EMC has received grants and/or research support from Dairy Farmers of Canada, Lallemand Health Solutions, and Ocean Spray and has received consultant fees or speaker or travel support from Danone, Nestlé, and Lallemand Health Solutions. DJAJ has received research grants from Saskatchewan & Alberta Pulse Growers Associations; the Agricultural Bioproducts Innovation Program through the Pulse Research Network; the Advanced Foods and Material Network; Loblaw Companies Ltd.; Unilever Canada and Netherlands; Barilla; the Almond Board of California; Agriculture and Agri‐food Canada; Pulse Canada; Kellogg's Company, Canada; Quaker Oats, Canada; Procter & Gamble Technical Centre Ltd.; Bayer Consumer Care, Springfield, NJ; Pepsi/Quaker; International Nut & Dried Fruit Council (INC); Soy Foods Association of North America; the Coca‐Cola Company (investigator initiated, unrestricted grant); Solae; Haine Celestial; the Sanitarium Company; Orafti; the International Tree Nut Council Nutrition Research and Education Foundation; the Peanut Institute; Soy Nutrition Institute (SNI); the Canola and Flax Councils of Canada; the Calorie Control Council; the Canadian Institutes of Health Research (CIHR); the Canada Foundation for Innovation (CFI); and the Ontario Research Fund (ORF). He has received in‐kind supplies for trials as a research support from the Almond board of California, Walnut Council of California, the Peanut Institute, Barilla, Unilever, Unico, Primo, Loblaw Companies, Quaker (Pepsico), Pristine Gourmet, Bunge Limited, Kellogg Canada, and WhiteWave Foods. He has been on the speaker's panel, served on the scientific advisory board, and/or received travel support and/or honoraria from 2020 China Glycemic Index (GI) International Conference, Atlantic Pain Conference, Academy of Life Long Learning, the Almond Board of California, Canadian Agriculture Policy Institute, Loblaw Companies Ltd, the Griffin Hospital (for the development of the NuVal scoring system), the Coca‐Cola Company, Epicure, Danone, Diet Quality Photo Navigation (DQPN), Better Therapeutics (FareWell), Verywell, True Health Initiative (THI), Heali AI Corp, Institute of Food Technologists (IFT), Soy Nutrition Institute (SNI), Herbalife Nutrition Institute (HNI), Saskatchewan & Alberta Pulse Growers Associations, Sanitarium Company, Orafti, the International Tree Nut Council Nutrition Research and Education Foundation, the Peanut Institute, Herbalife International, Pacific Health Laboratories, Nutritional Fundamentals for Health (NFH), Barilla, Metagenics, Bayer Consumer Care, Unilever Canada and Netherlands, Solae, Kellogg, Quaker Oats, Procter & Gamble, Abbott Laboratories, Dean Foods, the California Strawberry Commission, Haine Celestial, PepsiCo, the Alpro Foundation, Pioneer Hi‐Bred International, DuPont Nutrition and Health, Spherix Consulting and WhiteWave Foods, the Advanced Foods and Material Network, the Canola and Flax Councils of Canada, Agri‐Culture and Agri‐Food Canada, the Canadian Agri‐Food Policy Institute, Pulse Canada, the Soy Foods Association of North America, the Nutrition Foundation of Italy (NFI), Nutra‐Source Diagnostics, the McDougall Program, the Toronto Knowledge Translation Group (St. Michael's Hospital), the Canadian College of Naturopathic Medicine, The Hospital for Sick Children, the Canadian Nutrition Society (CNS), the American Society of Nutrition (ASN), Arizona State University, Paolo Sorbini Foundation and the Institute of Nutrition, and Metabolism and Diabetes. He received an honorarium from the United States Department of Agriculture to present the 2013 W.O. Atwater Memorial Lecture. He received the 2013 Award for Excellence in Research from the International Nut and Dried Fruit Council. He received funding and travel support from the Canadian Society of Endocrinology and Metabolism to produce mini cases for the Canadian Diabetes Association (CDA). He is a member of the International Carbohydrate Quality Consortium (ICQC). His wife, Alexandra L Jenkins, is a director and partner of INQUIS Clinical Research for the Food Industry, his two daughters, Wendy Jenkins and Amy Jenkins, have published a vegetarian book that promotes the use of the foods described here, The Portfolio Diet for Cardiovascular Risk Reduction (Academic Press/Elsevier 2020 ISBN:978‐0‐12‐810510‐8) and his sister, Caroline Brydson, received funding through a grant from the St. Michael's Hospital Foundation to develop a cookbook for one of his studies. JLS has received research support from the Canadian Foundation for Innovation, Ontario Research Fund, Province of Ontario Ministry of Research and Innovation and Science, Canadian Institutes of health Research (CIHR), Diabetes Canada, PSI Foundation, Banting and Best Diabetes Centre (BBDC), American Society for Nutrition (ASN), INC International Nut and Dried Fruit Council Foundation, National Dried Fruit Trade Association, National Honey Board (the U.S. Department of Agriculture [USDA] honey “Checkoff” program), International Life Sciences Institute (ILSI), Pulse Canada, Quaker Oats Center of Excellence, The United Soybean Board (the USDA soy “Checkoff” program), The Tate and Lyle Nutritional Research Fund at the University of Toronto, The Glycemic Control and Cardiovascular Disease in Type 2 Diabetes Fund at the University of Toronto (a fund established by the Alberta Pulse Growers), and The Nutrition Trialists Fund at the University of Toronto (a fund established by an inaugural donation from the Calorie Control Council). He has received in‐kind food donations to support a randomized controlled trial from the Almond Board of California, California Walnut Commission, Peanut Institute, Barilla, Unilever/Upfield, Unico/Primo, Loblaw Companies, Quaker, Kellogg Canada, WhiteWave Foods/Danone, and Nutrartis. He has received travel support, speaker fees, and/or honoraria from Diabetes Canada, Dairy Farmers of Canada, FoodMinds LLC, International Sweeteners Association, Nestlé, Pulse Canada, Canadian Society for Endocrinology and Metabolism (CSEM), GI Foundation, Abbott, General Mills, Biofortis, ASN, Northern Ontario School of Medicine, INC Nutrition Research & Education Foundation, European Food Safety Authority (EFSA), Comité Européen des Fabricants de Sucre (CEFS), Nutrition Communications, International Food Information Council (IFIC), Calorie Control Council, International Glutamate Technical Committee, and Physicians Committee for Responsible Medicine. He has or has had ad hoc consulting arrangements with Perkins Coie LLP, Tate & Lyle, Wirtschaftliche Vereinigung Zucker e.V., Danone, and Inquis Clinical Research. He is a member of the European Fruit Juice Association Scientific Expert Panel and former member of the Soy Nutrition Institute (SNI) Scientific Advisory Committee. He is on the Clinical Practice Guidelines Expert Committees of Diabetes Canada, European Association for the study of Diabetes (EASD), Canadian Cardiovascular Society (CCS), and Obesity Canada/Canadian Association of Bariatric Physicians and Surgeons. He serves or has served as an unpaid scientific advisor for the Food, Nutrition, and Safety Program (FNSP) and the Technical Committee on Carbohydrates of ILSI North America. He is a member of the International Carbohydrate Quality Consortium (ICQC), Executive Board Member of the Diabetes and Nutrition Study Group (DNSG) of the EASD and Director of the Toronto 3D Knowledge Synthesis and Clinical Trials foundation. His wife is an employee of AB InBev. There are no patents, products in development, or marketed products to declare.

## Supporting information


**Table S1.** MOOSE (Meta‐analyses Of Observational Studies in Epidemiology) Checklist
**Table S2.** PRISMA Checklist (continued on next page)^a^.
**Table S3.** Search strategy
**Table S4.** PICOTS framework of the search strategy and inclusion/exclusion criteria.
**Table S5a.** Characteristics of prospective cohort studies assessing dietary tree nut and peanut intake and overweight or obesity incidence (5 cohorts, N = 520,331).
**Table S5b.** Characteristics of prospective cohort studies assessing dietary tree nut and peanut intake and body weight change (5 cohorts, N = 500,150).
**Table S5c.** Characteristics of prospective cohort studies assessing dietary tree nut and peanut intake and incidence of ≥5 kg weight gain (3 cohorts, N = 195,595).
**Table S5d.** Characteristics of prospective cohort studies assessing dietary tree nut and peanut intake and incidence of waist circumference increasing above recommendation (2 cohorts, N = 9,887).
**Table S6a.** Analysis of confounding variables among prospective cohort studies assessing dietary tree nut and peanut intake and overweight or obesity incidence.
**Table S6b.** Analysis of confounding variables among prospective cohort studies assessing dietary tree nut and peanut intake and body weight change.
**Table S6c.** Analysis of confounding variables among prospective cohort studies assessing dietary tree nut and peanut intake and incidence of ≥5 kg weight gain.
**Table S6d.** Analysis of confounding variables among prospective cohort studies assessing dietary tree nut and peanut intake and incidence of waist circumference increasing above recommendation.
**Table S7.** Characteristics of randomized controlled trials assessing dietary tree nut and peanut intake and adiposity outcomes (114 trial comparisons, N = 5,873).
**Table S8.** Newcastle Ottawa Scale (NOS) for assessing the quality of prospective cohort studies.
**Table S9.** Continuous *A priori* subgroup analysis for the effect of nut consumption on measures of adiposity in randomized controlled trials.
**Table S10.** Sensitivity analyses assessing the effect of the systematic removal of an individual study on altering the significance of the pooled effect estimate or the evidence for heterogeneity for the prospective cohort studies pooled analyses. ^a^
**Table S11.** Sensitivity analysis of the systematic removal of each trial. ^a^
**Table S12.** Sensitivity analysis of the use of correlation coefficient of 0.25 and 0.75 for crossover trials.
**Table S13.** GRADE assessments for the prospective cohort studies.
**Table S14.** GRADE assessment of certainty of evidence for the outcomes of interest of randomized controlled trials.
**Figure S1.** Cochrane risk of bias summary for all included randomized controlled trials
**Figure S2.** Risk of bias proportion graph for all included randomized controlled trials
**Figure S3.** Forest plot of prospective cohorts investigating the association of nut consumption on overweight/obesity risk.
**Figure S4a.** Forest plot of prospective cohorts investigating the association of nut consumption on body weight change (kg).
**Figure S5.** Forest plot of prospective cohorts investigating the association of nut consumption on weight gain (≥5 kg) incidence.
**Figure S6.** Forest plot of prospective cohorts investigating the association of nut consumption on the incidence of waist circumference increasing ≥94 cm in men and ≥80 cm in women.
**Figure S7.** Forest plot of randomized controlled trials investigating the effects of nut consumption on body weight (kg).
**Figure S8.** Forest plot of randomized controlled trials investigating the effects of nut consumption on BMI (kg/m2).
**Figure S9.** Forest plot of randomized controlled trials investigating the effects of nut consumption on body fat (%).
**Figure S10.** Forest plot of randomized controlled trials investigating the effects of nut consumption on waist circumference (cm).
**Figure S11.** Forest plot of randomized controlled trials investigating the effects of nut consumption on waist‐to‐hip ratio.
**Figure S12.** Forest plot of randomized controlled trials investigating the effects of nut consumption on visceral adipose tissue.
**Figure S13.** Linear and non‐linear meta‐regression analyses for the effect of nut consumption on measures of adiposity from prospective cohorts.
**Figure S14.** Linear and non‐linear meta‐regression analyses for the effect of nut consumption on measures of adiposity from randomized controlled trials.
**Figure S15.** *A priori* subgroup analysis for mean differences (95% CIs) of the effects of nut consumption in on body weight (kg).
**Figure S16.** *A priori* subgroup analysis for mean differences (95% CIs) of the effects of nut consumption in on BMI (kg/m2).
**Figure S17.** *A priori* subgroup analysis for mean differences (95% CIs) of the effects of nut consumption on body fat (%).
**Figure S18.** *A priori* subgroup analysis for mean differences (95% CIs) of the effects of nut consumption on waist circumference (cm).
**Figure S19.** *A priori* subgroup analysis for mean differences (95% CIs) of the effects of nut consumption on waist‐to‐hip ratio.
**Figure S20.** Forest plot of prospective cohorts investigating the association of nut consumption on overweight/obesity risk using a fixed‐effects model.
**Figure S21a.** Forest plot of prospective cohorts investigating the association of nut consumption on body weight change (kg) with the use of a fixed‐effects model.
**Figure S22.** Forest plot of prospective cohorts investigating the association of nut consumption on weight gain (≥5 kg) incidence with the use of a random‐effects model.
**Figure S23.** Forest plot of prospective cohorts investigating the association of nut consumption on the incidence of waist circumference increasing ≥94 cm in men and ≥80 cm in women with the use of a random‐effects model.
**Figure S24.** Forest plot of randomized controlled trials investigating the effects of nut consumption on body weight (kg) with the use of a fixed‐effects model.
**Figure S25.** Forest plot of randomized controlled trials investigating the effects of nut consumption on body mass index (BMI) (kg/m2) with the use of a fixed‐effects model.
**Figure S26.** Forest plot of randomized controlled trials investigating the effects of nut consumption on body fat (%) with the use of a fixed‐effects model.
**Figure S27.** Forest plot of randomized controlled trials investigating the effects of nut consumption on waist circumference (cm) with the use of a fixed‐effects model.
**Figure S28.** Forest plot of randomized controlled trials investigating the effects of nut consumption on waist‐to‐up ratio with the use of a fixed‐effects model.
**Figure S29.** Forest plot of randomized controlled trials investigating the effects of nut consumption on visceral adipose tissue with the use of a fixed‐effects model.
**Figure S30.** Risk of bias (using The Cochrane Collaboration Tool) subgroup analysis for the effect of nut consumption on body weight (kg).
**Figure S31.** Risk of bias (using The Cochrane Collaboration Tool) subgroup analysis for the effect of nut consumption on BMI (kg/m^2^).
**Figure S32.** Risk of bias (using The Cochrane Collaboration Tool) subgroup analysis for the effect of nut consumption on body fat (%).
**Figure S33.** Risk of bias (using The Cochrane Collaboration Tool) subgroup analysis for the effect of nut consumption on waist circumference (cm).
**Figure S34.** Risk of bias (using The Cochrane Collaboration Tool) subgroup analysis for the effect of nut consumption on waist‐to‐hip ratio.
**Figure S35.** Funnel plot for the effect of nut consumption on adiposity measures.
**Figure S36.** Trim‐and‐Fill analysis for the effect of nut consumption on adiposity measures.
