# Supplementary material for: Are fatty nuts a weighty concern? A systematic review and meta‐analysis and dose–response meta‐regression of prospective cohorts and randomized controlled trials
Source: Obes Rev. 2021 Sep 8;22(11):e13330. doi: 10.1111/obr.13330 (PMC9285885; doi:10.1111/obr.13330)
Supplement: Supplementary file 1 — Table S1. MOOSE (Meta‐analyses Of Observational Studies in Epidemiology) Checklist Table S2. PRISMA Checklist (continued on next page)a. Table S3. Search strategy Table S4. PICOTS framework of the search strategy and inclusion/exclusion criteria. Table S5a. Characteristics of prospective cohort studies assessing dietary tree nut and peanut intake and overweight or obesity incidence (5 cohorts, N = 520,331). Table S5b. Characteristics of prospective cohort studies assessing dietary tree nut and peanut intake and body weight change (5 cohorts, N = 500,150). Table S5c. Characteristics of prospective cohort studies assessing dietary tree nut and peanut intake and incidence of ≥5 kg weight gain (3 cohorts, N = 195,595). Table S5d. Characteristics of prospective cohort studies assessing dietary tree nut and peanut intake and incidence of waist circumference increasing above recommendation (2 cohorts, N = 9,887). Table S6a. Analysis of confounding variables among prospective cohort studies assessing dietary tree nut and peanut intake and overweight or obesity incidence. Table S6b. Analysis of confounding variables among prospective cohort studies assessing dietary tree nut and peanut intake and body weight change. Table S6c. Analysis of confounding variables among prospective cohort studies assessing dietary tree nut and peanut intake and incidence of ≥5 kg weight gain. Table S6d. Analysis of confounding variables among prospective cohort studies assessing dietary tree nut and peanut intake and incidence of waist circumference increasing above recommendation. Table S7. Characteristics of randomized controlled trials assessing dietary tree nut and peanut intake and adiposity outcomes (114 trial comparisons, N = 5,873). Table S8. Newcastle Ottawa Scale (NOS) for assessing the quality of prospective cohort studies. Table S9. Continuous A priori subgroup analysis for the effect of nut consumption on measures of adiposity in randomized controlled trials. Table S10. Sensitivity [file OBR-22-0-s001.pdf]

ONLINE SUPPLEMENTARY MATERIAL

Are Fatty Nuts a Weighty Concern? A Systematic Review and Meta-Analysis and Dose-response Meta-regression of Prospective Cohorts and Randomized Controlled Trials

Stephanie K Nishi PhD, RD[1-6], Effie Viguiliouk MSc[1-3], Sonia Blanco Mejia MD[1-3], Cyril WC Kendall PhD[1-3,7], Richard P Bazinet PhD[1], Anthony J Hanley PhD [1,8,10], Elena M Comelli PhD [1,10], Jordi Salas Salvadó PhD, MD[4-6], David JA Jenkins MD, DSc [1-3,8,11,12], John L Sievenpiper MD, PhD[1-3,8,11,12]

[1]Department of Nutritional Sciences, Faculty of Medicine, University of Toronto, Toronto, ON, Canada
[2]Toronto 3D (Diet, Digestive Tract and Disease) Knowledge Synthesis and Clinical Trials Unit, Toronto, ON, Canada
[3]Clinical Nutrition and Risk Factor Modification Center, St. Michael's Hospital, Toronto, ON, Canada
[4]Biomedical Research Centre for Obesity Physiopathology and Nutrition Network (CIBEROBN), Instituto de Salud Carlos III (ISCIII), Madrid, Spain.
[5]Universitat Rovira i Virgili, Department of Biochemistry and Biotechnology, Human Nutrition Unit, Reus, Spain
[6]Institut d'Investigació Sanitària Pere Virgili (IISPV), Reus, Spain
[7]College of Pharmacy and Nutrition, University of Saskatchewan, Saskatoon, SK, Canada
[8]Department of Medicine, University of Toronto, Toronto, ON, Canada
[9]Dalla Lana School of Public Health, University of Toronto, Toronto, ON, Canada
[10]Joannah and Brian Lawson Centre for Child Nutrition, University of Toronto, Toronto, ON, Canada
[11]Division of Endocrinology & Metabolism, St. Michael's Hospital, Toronto, ON, Canada
[12]Li Ka Shing Knowledge Institute, St. Michael's Hospital, Toronto, ON, Canada

Corresponding author:
Dr. John L Sievenpiper, MD, PhD, FRCPC
St. Michael's Hospital
#6137-61 Queen Street East,
Toronto, ON, M5C 2T2, CANADA
T: +1 416-867-7475
F: +1 416-867-7495
E: john.sievenpiper@utoronto.ca

SUPPLEMENTARY TABLES....6
Supplementary Table 1. MOOSE (Meta-analyses Of Observational Studies in Epidemiology) Checklist 6
Supplementary Table 2. PRISMA Checklist....8
Supplementary Table 3. Search strategy....10
Supplementary Table 4. PICOTS framework 12
Supplementary Table 5a. Characteristics of prospective cohort studies assessing dietary tree nut and peanut intake and overweight or obesity incidence....13
Supplementary Table 5b. Characteristics of prospective cohort studies assessing dietary tree nut and peanut intake and body weight change 14
Supplementary Table 5c. Characteristics of prospective cohort studies assessing dietary tree nut and peanut intake and incidence of ≥5 kg weight gain....15
Supplementary Table 5d. Characteristics of prospective cohort studies assessing dietary tree nut and peanut intake and incidence of waist circumference increasing above recommendation 16
Supplementary Table 6a. Analysis of confounding variables among prospective cohort studies assessing dietary tree nut and peanut intake and overweight or obesity incidence....17
Supplementary Table 6b. Analysis of confounding variables among prospective cohort studies assessing dietary tree nut and peanut intake and body weight change....18
Supplementary Table 6c. Analysis of confounding variables among prospective cohort studies assessing dietary tree nut and peanut intake and incidence of ≥5 kg weight gain....19
Supplementary Table 6d. Analysis of confounding variables among prospective cohort studies assessing dietary tree nut and peanut intake and incidence of waist circumference increasing above recommendation....20
Supplementary Table 7. Characteristics of randomized controlled trials assessing dietary tree nut and peanut intake and adiposity outcomes....21
Supplementary Table 8. Newcastle Ottawa Scale (NOS) for assessing the quality of prospective cohort studies. 35
Supplementary Table 9. Continuous *A priori* subgroup analysis for the effect of nut consumption on measures of adiposity in randomized controlled trials....36
Supplementary Table 10. Sensitivity analyses assessing the effect of the systematic removal of an individual study on altering the significance of the pooled effect estimate or the evidence for heterogeneity for the prospective cohort studies pooled analyses....38
Supplementary Table 12. Sensitivity analysis of the use of correlation coefficient of 0.25 and 0.75 for crossover trials....46
Supplementary Table 13. GRADE assessments for the prospective cohort studies. 47

Supplementary Table 14. GRADE assessment of certainty of evidence for the outcomes of interest of randomized controlled trials. 48

SUPPLEMENTARY FIGURES....49

Supplementary Figure 1. Cochrane risk of bias summary for all included randomized controlled trials 49

Supplementary Figure 2. Risk of bias proportion graph for all included randomized controlled trials....54

Supplementary Figure 3. Forest plot of prospective cohorts investigating the association of nut consumption on overweight/obesity risk....55

Supplementary Figure 4a. Forest plot of prospective cohorts investigating the association of nut consumption on body weight change (kg) 56

Supplementary Figure 4b. Forest plot of prospective cohorts investigating the association of nut consumption on body weight change (kg), using data from the least adjusted model....56

Supplementary Figure 5. Forest plot of prospective cohorts investigating the association of nut consumption on weight gain (≥5 kg) incidence....57

Supplementary Figure 6. Forest plot of prospective cohorts investigating the association of nut consumption on the incidence of waist circumference increasing ≥94 cm in men and ≥80 cm in women.58

Supplementary Figure 7. Forest plot of randomized controlled trials investigating the effects of nut consumption on body weight (kg)....59

Supplementary Figure 8. Forest plot of randomized controlled trials investigating the effects of nut consumption on BMI (kg/m^2) 63

Supplementary Figure 9. Forest plot of randomized controlled trials investigating the effects of nut consumption on body fat (%). 66

Supplementary Figure 10. Forest plot of randomized controlled trials investigating the effects of nut consumption on waist circumference (cm)....68

Supplementary Figure 11. Forest plot of randomized controlled trials investigating the effects of nut consumption on waist-to-hip ratio....70

Supplementary Figure 12. Forest plot of randomized controlled trials investigating the effects of nut consumption on visceral adipose tissue....71

Supplementary Figure 13. Linear and non-linear meta-regression analyses for the effect of nut consumption on measures of adiposity from prospective cohorts. 72

Supplementary Figure 14. Linear and non-linear meta-regression analyses for the effect of nut consumption on measures of adiposity from randomized controlled trials....73

Supplementary Figure 15. *A priori* subgroup analysis for mean differences (95% CIs) of the effects of nut consumption in on body weight (kg)....74

Supplementary Figure 16. *A priori* subgroup analysis for mean differences (95% CIs) of the effects of nut consumption in on BMI (kg/m^2) 78

Supplementary Figure 17. *A priori* subgroup analysis for mean differences (95% CIs) of the effects of nut consumption on body fat (%). 82

Supplementary Figure 18. *A priori* subgroup analysis for mean differences (95% CIs) of the effects of nut consumption on waist circumference (cm) 85

Supplementary Figure 19. *A priori* subgroup analysis for mean differences (95% CIs) of the effects of nut consumption on waist-to-hip ratio 89

Supplementary Figure 20. Forest plot of prospective cohorts investigating the association of nut consumption on overweight/obesity risk using a fixed-effects model. 92

Supplementary Figure 21a. Forest plot of prospective cohorts investigating the association of nut consumption on body weight change (kg) with the use of a fixed-effects model. 93

Supplementary Figure 21b. Forest plot of prospective cohorts investigating the association of nut consumption on body weight change (kg) with the use of a fixed-effects model, using the least adjusted data. 93

Supplementary Figure 22. Forest plot of prospective cohorts investigating the association of nut consumption on weight gain (≥5 kg) incidence with the use of a random-effects model. 94

Supplementary Figure 23. Forest plot of prospective cohorts investigating the association of nut consumption on the incidence of waist circumference increasing ≥94 cm in men and ≥80 cm in women with the use of a random-effects model. 95

Supplementary Figure 24. Forest plot of randomized controlled trials investigating the effects of nut consumption on body weight (kg) with the use of a fixed-effects model. 96

Supplementary Figure 25. Forest plot of randomized controlled trials investigating the effects of nut consumption on body mass index (BMI) (kg/m^2) with the use of a fixed-effects model 99

Supplementary Figure 26. Forest plot of randomized controlled trials investigating the effects of nut consumption on body fat (%) with the use of a fixed-effects model 102

Supplementary Figure 27. Forest plot of randomized controlled trials investigating the effects of nut consumption on waist circumference (cm) with the use of a fixed-effects model 104

Supplementary Figure 28. Forest plot of randomized controlled trials investigating the effects of nut consumption on waist-to-up ratio with the use of a fixed-effects model. 106

Supplementary Figure 29. Forest plot of randomized controlled trials investigating the effects of nut consumption on visceral adipose tissue with the use of a fixed-effects model 107

Supplementary Figure 30. Risk of bias (using The Cochrane Collaboration Tool) subgroup analysis for the effect of nut consumption on body weight (kg). 108

Supplementary Figure 31. Risk of bias (using The Cochrane Collaboration Tool) subgroup analysis for the effect of nut consumption on BMI (kg/m^2). 109

Supplementary Figure 32. Risk of bias (using The Cochrane Collaboration Tool) subgroup analysis for the effect of nut consumption on body fat (%) 110

Supplementary Figure 33. Risk of bias (using The Cochrane Collaboration Tool) subgroup analysis for the effect of nut consumption on waist circumference (cm) 112

Supplementary Figure 34. Risk of bias (using The Cochrane Collaboration Tool) subgroup analysis for the effect of nut consumption on waist-to-hip ratio. 114

Supplementary Figure 35. Funnel plot for the effect of nut consumption on adiposity measures. 115

Supplementary Figure 36. Trim-and-Fill analysis for the effect of nut consumption on adiposity measures. 116

REFERENCES 117

SUPPLEMENTARY TABLES

Supplementary Table 1. MOOSE (Meta-analyses Of Observational Studies in Epidemiology) Checklist (continued on next page).

Reporting Criteria	Reported (Yes/No)	Reported on Page Number
**Reporting of Background**		
Problem definition	Yes	10
Hypothesis statement	NA	NA
Description of Study Outcome(s)	Yes	11
Type of exposure or intervention used	Yes	11, Supplementary Table 4
Type of study design used	Yes	10,11
Study population	Yes	11
**Reporting of Search Strategy**		
Qualifications of searchers (eg, librarians and investigators)	Yes	1
Search strategy, including time period included in the synthesis and keywords	Yes	10,11, Supplementary Tables 3, 4
Effort to include all available studies, including contact with authors	Yes	15
Databases and registries searched	Yes	10,11
Search software used, name and version, including special features used (eg, explosion)	Yes	10, Supplementary Table 3
Use of hand searching (eg, reference lists of obtained articles)	Yes	10
List of citations located and those excluded, including justifications	Yes	Available: https://utoronto-my.sharepoint.com/:x:/r/personal/s_nishi_mail_utoronto_ca/Documents/SRMA%20Nuts%20%26%20Adiposity/SRMA%20Nuts%20%26%20Adiposity_Search.xlsx?d=wff3703c625ba443e9a6f36ee33caaefd&csf=1&e=jhYHKz
Method for addressing articles published in languages other than English	Yes	11
Method of handling abstracts and unpublished studies	Yes	15
Description of any contact with authors	Yes	15
**Reporting of Methods**		
Description of relevance or appropriateness of studies assembled for assessing the hypothesis to be tested	Yes	17
Rationale for the selection and coding of data (eg, sound clinical principles or convenience)	Yes	Available: https://utoronto-my.sharepoint.com/:x:/r/personal/s_nishi_mail_utoronto_ca/Documents/SRMA%20Nuts%20%26%20Adiposity/SRMA%20Nuts%20%26%20Adiposity_Search.xlsx?d=wff3703c625ba443e9a6f36ee33caaefd&csf=1&e=jhYHKz
Documentation of how data were classified and coded (eg, multiple raters, blinding, and interrater reliability)	Yes	11
Assessment of confounding (eg, comparability of cases and controls in studies where appropriate)	Yes	12

Supplementary Table 1. MOOSE (Meta-analyses Of Observational Studies in Epidemiology) Checklist.

Reporting Criteria	Reported (Yes/No)	Reported on Page Number
Assessment of study quality, including blinding of quality assessors; stratification or regression on possible predictors of study results	Yes	12
Assessment of heterogeneity	Yes	14
Description of statistical methods (eg, complete description of fixed or random effects models, justification of whether the chosen models account for predictors of study results, dose-response models, or cumulative meta-analysis) in sufficient detail to be replicated)	Yes	12-15
Provision of appropriate tables and graphics	Yes	16-22
**Reporting of Results**		
Table giving descriptive information for each study included	Yes	37, Supplementary Table 5
Results of sensitivity testing (eg, subgroup analysis)	Yes	19
Indication of statistical uncertainty of findings	Yes	21,22
**Reporting of Discussion**		
Quantitative assessment of bias (eg, publication bias)	Yes	17,21
Justification for exclusion (eg, of non-English-language citations)	Yes	11, Figure 1
Assessment of quality of included studies	Yes	21,22
**Reporting of Conclusions**		
Consideration of alternative explanations for observed results	Yes	22-24
Generalization of the conclusions (ie, appropriate for the data presented and within the domain of the literature review)	Yes	22-24
Guidelines for future research	Yes	26,27
Disclosure of funding source	Yes	2

From: Stroup DF, Berlin JA, Morton SC, Olkin I, Williamson GD, Rennie D, et al. Meta-analysis of observational studies in epidemiology: A proposal for reporting. JAMA 2000, 283:2008-2012.

Supplementary Table 2. PRISMA Checklist (continued on next page)[a].

Section/topic	#	Checklist item	Reported on page #
**TITLE**			
Title	1	Identify the report as a systematic review, meta-analysis, or both.	1
**ABSTRACT**			
Structured summary	2	Provide a structured summary including, as applicable: background; objectives; data sources; study eligibility criteria, participants, and interventions; study appraisal and synthesis methods; results; limitations; conclusions and implications of key findings; systematic review registration number.	8
**INTRODUCTION**			
Rationale	3	Describe the rationale for the review in the context of what is already known.	9,10
Objectives	4	Provide an explicit statement of questions being addressed with reference to participants, interventions, comparisons, outcomes, and study design (PICOS).	Suppl. Table 4
**METHODS**			
Protocol and registration	5	Indicate if a review protocol exists, if and where it can be accessed (e.g., Web address), and, if available, provide registration information including registration number.	8
Eligibility criteria	6	Specify study characteristics (e.g., PICOS, length of follow-up) and report characteristics (e.g., years considered, language, publication status) used as criteria for eligibility, giving rationale.	10,11, Suppl. Table 4
Information sources	7	Describe all information sources (e.g., databases with dates of coverage, contact with study authors to identify additional studies) in the search and date last searched.	10
Search	8	Present full electronic search strategy for at least one database, including any limits used, such that it could be repeated.	Suppl. Table 3
Study selection	9	State the process for selecting studies (i.e., screening, eligibility, included in systematic review, and, if applicable, included in the meta-analysis).	10,11
Data collection process	10	Describe method of data extraction from reports (e.g., piloted forms, independently, in duplicate) and any processes for obtaining and confirming data from investigators.	11
Data items	11	List and define all variables for which data were sought (e.g., PICOS, funding sources) and any assumptions and simplifications made.	11, Suppl. Table 4
Risk of bias in individual studies	12	Describe methods used for assessing risk of bias of individual studies (including specification of whether this was done at the study or outcome level), and how this information is to be used in any data synthesis.	12
Summary measures	13	State the principal summary measures (e.g., risk ratio, difference in means).	12
Synthesis of results	14	Describe the methods of handling data and combining results of studies, if done, including measures of consistency (e.g., I2) for each meta-analysis.	12

Supplementary Table 2. PRISMA Checklist (continued)[1].

Section/topic	#	Checklist item	Reported on page #
Synthesis of results	14	Describe the methods of handling data and combining results of studies, if done, including measures of consistency (e.g., I2) for each meta-analysis.	12-15
Risk of bias across studies	15	Specify any assessment of risk of bias that may affect the cumulative evidence (e.g., publication bias, selective reporting within studies).	12-15
Additional analyses	16	Describe methods of additional analyses (e.g., sensitivity or subgroup analyses, meta-regression), if done, indicating which were pre-specified.	12-15
**RESULTS**			
Study selection	17	Give numbers of studies screened, assessed for eligibility, and included in the review, with reasons for exclusions at each stage, ideally with a flow diagram.	15, Figure 1
Study characteristics	18	For each study, present characteristics for which data were extracted (e.g., study size, PICOS, follow-up period) and provide the citations.	16, Table 2, Suppl. Table 7
Risk of bias within studies	19	Present data on risk of bias of each study and, if available, any outcome level assessment (see item 12).	17, Suppl. Figures 2-3
Results of individual studies	20	For all outcomes considered (benefits or harms), present, for each study: (a) simple summary data for each intervention group (b) effect estimates and confidence intervals, ideally with a forest plot.	Suppl. Figures 9-14
Synthesis of results	21	Present results of each meta-analysis done, including confidence intervals and measures of consistency.	17-20, Figure 3
Risk of bias across studies	22	Present results of any assessment of risk of bias across studies (see Item 15).	Suppl. Table 14, Suppl. Figures 11,25,27,29,31,33
Additional analysis	23	Give results of additional analyses, if done (e.g., sensitivity or subgroup analyses, meta-regression [see Item 16]).	20-22, Suppl. Tables 11-12, Suppl. Figures 14-34
**DISCUSSION**			
Summary of evidence	24	Summarize the main findings including the strength of evidence for each main outcome; consider their relevance to key groups (e.g., healthcare providers, users, and policy makers).	22-27
Limitations	25	Discuss limitations at study and outcome level (e.g., risk of bias), and at review-level (e.g., incomplete retrieval of identified research, reporting bias).	24,25
Conclusions	26	Provide a general interpretation of the results in the context of other evidence, and implications for future research.	22-27
**FUNDING**			
Funding	27	Describe sources of funding for the systematic review and other support (e.g., supply of data); role of funders for the systematic review.	2

[a]Moher D, Liberati A, Tetzlaff J, Altman DG, The PRISMA Group (2009). Preferred Reporting Items for Systematic Reviews and Meta-Analyses: The PRISMA Statement. PLoS Med 6(7): e1000097. doi:10.1371/journal.pmed1000097.

Supplementary Table 3. Search strategy.

MEDLINE	
1	exp Nuts/
2	nut.mp
3	nuts.mp
4	exp Bertholletia/
5	Brazil nuts.mp
6	walnut*.mp
7	exp Juglans/
8	almond*.mp
9	exp Prunus/
10	cashew*.mp
11	exp Anacardium/
12	hazelnut*.mp
13	exp Corylus/
14	filbert*.mp
15	macadamia*.mp
16	exp Macadamia/
17	pecan*.mp
18	exp Carya/
19	pine nuts.mp
20	exp Pinus/
21	pistachio*.mp
22	exp Pistacia/
23	peanut*.mp
24	Groundnut*.mp
25	exp Arachis hypogaea/
26	or/1-25
27	body weight*.mp
28	exp Body Weight/
29	obes*.mp
30	exp Obesity/
31	overweight.mp
32	exp Overweight/
33	Body mass index.mp
34	BMI.mp
35	body composition.mp
36	exp Body Composition/
37	waist circumference.mp
38	exp Waist Circumference/
39	waist-hip ratio.mp
40	body fat.mp
41	exp Adipose Tissue/
42	body fat distribution.mp
43	exp Body Fat Distribution/
44	visceral fat.mp
45	exp Intra-Abdominal Fat/
46	visceral adipose tissue.mp
47	quetelet index.mp

EMBASE	
1	exp nut/
2	nut.mp
3	nuts.mp
4	exp Bertholletia/
5	bertholletia.mp
6	exp Brazil nut/
7	Brazil nuts.mp
8	walnut*.mp
9	exp walnut/
10	juglans.mp
11	almond*.mp
12	exp almond/
13	exp Prunus/
14	Prunus.mp
15	cashew*.mp
16	exp Anacardium/
17	hazelnut*.mp
18	exp hazelnut/
19	Corylus.mp
20	filbert*.mp
21	macadamia*.mp
22	exp Macadamia/
23	pecan*.mp
24	Carya.mp
25	exp Carya/
26	pine nuts.mp
27	Pinus.mp
28	pistachio*.mp
29	Pistacia.mp
30	exp Pistacia/
31	peanut*.mp
32	groundnut*.mp
33	Arachis hypogaea.mp
34	or/1-33
35	exp body weight/
36	body weight*.mp
37	obes*.mp
38	exp obesity/
39	overweight.mp
40	body mass index.mp
41	exp body mass/
42	BMI.mp
43	quetelet index.mp
44	body composition.mp
45	exp body composition/
46	waist circumference.mp
47	exp waist circumference/

COCHRANE	
1	Nuts/
2	nuts.mp
3	nut.mp
4	Brazil nut.mp
5	Brazil nuts.mp
6	pine nut.mp
7	walnut*.mp
8	Juglans/
9	almond*.mp
10	Prunus/
11	pecan*.mp
12	pistachio*.mp
13	Pistacia/
14	cashew*.mp
15	hazelnut*.mp
16	Corylus/
17	macadamia.mp
18	Anacardium.mp
19	Pinus.mp
20	peanut*.mp
21	Arachis hypogaea.mp
22	or/1-21
23	body weight*.mp
24	obes*.mp
25	overweight.mp
26	body mass index.mp
27	BMI.mp
28	body composition.mp.
29	waist circumference.mp
30	waist-hip ratio.mp
31	body fat.mp
32	Adipose Tissue/
33	body fat distribution.mp
34	visceral fat.mp
35	Intra-Abdominal Fat/
36	visceral adipose tissue.mp
37	quetelet index.mp
38	anthropometry.mp
39	adiposity.mp
40	or/23-39
41	22 and 40

48	anthropometry.mp
49	exp Anthropometry/
50	adiposity.mp
51	or/27-50
52	26 and 51

48	waist-hip ratio.mp
49	body fat.mp
50	adipose tissue.mp
51	exp adipose tissue/
52	body fat distribution.mp
53	visceral fat.mp
54	exp intraabdominal fat/
55	visceral adipose tissue.mp
56	anthropometry.mp
57	adiposity.mp
58	or/35-57
59	34 and 58
60	limit 59 to animals
61	59 not 60

Search terms encompassed those specifying the exposure and outcomes. The exposure included tree nuts (one-seeded fruit in a hard shell, including almonds, Brazil nuts, cashews, hazelnuts, macadamia nuts, pecans, pine nuts, pistachios, walnuts) and peanuts (technically a member of the legume family, but sharing a similar nutritional profile with tree nuts), herein referred to collectively as ‘nuts’. Outcomes were measures of adiposity, including, but not limited to overweight, obesity, body weight, body mass index (BMI), and waist circumference. The search was limited to human studies and had no language restrictions.

Supplementary Table 4. PICOTS framework of the search strategy and inclusion/exclusion criteria.

**PICOTS framework[a] defined in the present systematic review and meta-analysis**					
**Participants**	**Interventions**	**Comparators**	**Outcomes**	**Time[b]**	**Setting**
Adult men and women, excluding pregnant or breastfeeding women.	Tree nuts (one-seeded fruit in a hard shell, including almonds, Brazil nuts, cashews, hazelnuts, macadamia nuts, pecans, pine nuts, pistachios, walnuts) and/or peanuts consumed whole or as butters. (Without uncontrolled co-intervention.)	Non-tree nut and/or peanut supplements or placebo.	Primary:<br>Overweight/Obesity Incidence (Prospective cohorts)<br>Body weight (RCTs)<br>Secondary:<br>Body weight (Prospective cohorts)<br>Weight gain (≥ 5 kg) incidence (Prospective cohorts)<br>Body mass index (BMI) (Prospective cohorts, RCTs)<br>Body Fat (%)(Prospective cohorts, RCTs)<br>Waist circumference (Prospective cohorts, RCTs)<br>Waist-to-hip ratio (Prospective cohorts, RCTs)<br>Visceral adipose tissue (Prospective cohorts, RCTs)	Prospective cohort: ≥ 1 year<br>RCT: ≥ 3 weeks	No setting or language restrictions.

[a]Moher D, Shamseer L, Clarke M, Ghersi D, Liberati A, Petticrew M, Shekelle P, Stewart LA and PRISMA-P Group. Preferred reporting items for systematic review and meta-analysis protocols (PRISMA-P) 2015 statement. Systematic Reviews 2015; 4:1. https://doi.org/10.1186/2046-4053-4-1.
When multiple publications existed for the same study, the article with the most applicable information and longest duration was included.

Supplementary Table 5a. Characteristics of prospective cohort studies assessing dietary tree nut and peanut intake and overweight or obesity incidence (5 cohorts, N=520,331).

Study, Reference	Cohort	Country	N	Age range, yr[a]	Mean FU duration, yr	No. cases	Outcome assessment method	Diet assessment method	Exposure	Lowest tile, (g/d)	Highest tile (g/d)	Funding source
Bes-Rastrollo et al. 2007	SUN	Spain	8865 (3700 M, 5165 W)	18-101	2.25	434	Self-reported	vSFFQ	Walnuts, almonds, hazelnuts, & peanuts	<3.3	≥21.4	Agency
El-Amari et al. 2016	AHS-2	USA	41845 (14437 M, 27408 W)	30-112	8	23,372	Self-reported	vSFFQ	Tree nuts & peanuts	0.2	3	Agency
Freisling et al. 2018 (BMI <25kg/m² at baseline)	EPIC-PANACEA	Denmark, France, Germany, Greece, Italy, the Netherlands, Norway, Spain, Sweden, UK	197,291 (M+W)	25-70	5	31,215	Centre Measured[b]	vSFFQ[c]	Tree nuts & peanuts[d]	0	12.4	Agency, Agency-Industry
Freisling et al. 2018 (BMI ≥25kg/m² at baseline)	EPIC-PANACEA	Denmark, France, Germany, Greece, Italy, the Netherlands, Norway, Spain, Sweden, UK	127,445 (M+W)	25-70	5	14,913	Centre Measured[b]	vSFFQ[c]	Tree nuts & peanuts[d]	0	12.4	Agency, Agency-Industry
Liu et al. 2019	NHS<br>NHS II<br>HPFS	USA	144885 (27521 M, 117364 W)	35-55<br>24-44<br>40-75	24<br>20<br>24	21,322[e]	Self-reported	vSFFQ	Tree nuts & peanuts	0	>14 g/d	Agency

[a]Based on baseline age range.
[b]Except self-reported in France, Norway, and the health conscious group of the Oxford centre.
[c]Denmark, Norway, Naples (Italy), and Umea (Sweden)]; Semi-quantitative FFQ + 7-day record, validated [UK]; FFQ+7-day record on lunch and dinner, validated [Malmo (Sweden).
[d]France, Germany, Greece, Ragusa (Italy), the Netherlands, Spain, UK]; Peanuts [Norway]; Peanuts, salted [Umea (Sweden)]; Walnuts, Hazelnuts, Almonds, Peanuts [Northern Italy]; Walnuts [Naples (Italy)]; Tree nuts, peanuts, and seeds [Spain]; Peanuts as snacks + other nuts added via open-ended questions or recorded at lunch and dinner meals [Malmo (Sweden)]; Peanut butter [Germany, the Netherlands, UK.
[e]This represents the total number of obesity incidence (BMI ≥30 kg/m²) among all three cohorts, within each cohort the number of obesity cases were as follows: NHS= 8,019 cases; NHS II= 10,838 cases; HPFS= 2,465 cases.
AHS-2=Adventist Health Study 2, d=day, EPIC -PANACEA= European Prospective Investigation into Cancer and Nutrition – Physical Activity, Nutrition, Alcohol, Cessation of smoking, Eating out of home in relation to Anthropometry, FU=follow-up, HPFS = Health Professionals Follow-Up Study, M=men, N=number of participants, NHS = Nurses' Health Study, NHS II = Nurses' Health Study II, No. = number, Sun = Seguimiento Universidad de Navarra study, UK=United Kingdom, USA=United States of America, vSFFQ=validated, semi-quantitative food frequency questionnaire, W=women, yr=year.

Supplementary Table 5b. Characteristics of prospective cohort studies assessing dietary tree nut and peanut intake and body weight change (5 cohorts, N=500,150).

Study, Reference	Cohort	Country	N	Age range, yr[a]	Mean FU duration, yr	Outcome assessment method	Diet assessment method	Exposure	Lowest tile (g/d)	Highest tile (g/d)	Funding source
Bes-Rastrollo et al. 2007	SUN	Spain	8865 (3700 M, 5165 W)	18-101	2.3	Self-reported	vSFFQ	Walnuts, almonds, hazelnuts, and peanuts	<3.3	≥21.4	Agency
Freisling et al. 2018	EPIC-PANACEA	Denmark, France, Germany, Greece, Italy, the Netherlands, Norway, Spain, Sweden, UK	373,293 (103,303 M, 269990 W)	25-70	5	Centre Measured[b]	vSFFQ and/or 7-d Food Record[c]	Tree nuts and peanuts[d]	0	12.4	Agency, Agency-Industry
Smith et al. 2015	NHS	USA	46994 W	30-55	24	Self-reported	vSFFQ	Nuts, not specified	0	~28	Agency
Smith et al. 2015	NHS II	USA	47928 W	25-42	16	Self-reported	vSFFQ	Nuts, not specified	0	~28	Agency
Smith et al. 2015	HPFS	USA	25862 M	40-75	24	Self-reported	vSFFQ	Nuts, not specified	0	~28	Agency

[a]Based on baseline age range.
[b]Except self-reported in France, Norway, and the health conscious group of the Oxford centre.
[c]Semi-quantitative FFQ, validated [Denmark, Norway, Naples (Italy), and Umea (Sweden)]; Semi-quantitative FFQ + 7-day record, validated [UK]; FFQ+7-day record on lunch and dinner, validated [Malmo (Sweden)].
[d]France, Germany, Greece, Ragusa (Italy), the Netherlands, Spain, UK]; Peanuts [Norway]; Peanuts, salted [Umea (Sweden)]; Walnuts, Hazelnuts, Almonds, Peanuts [Northern Italy]; Walnuts [Naples (Italy)]; Tree nuts, peanuts, and seeds [Spain]; Peanuts as snacks + other nuts added via open-ended questions or recorded at lunch and dinner meals [Malmo (Sweden)]; Peanut butter [Germany, the Netherlands, UK.
d=day, EPIC -PANACEA= European Prospective Investigation into Cancer and Nutrition – Physical Activity, Nutrition, Alcohol, Cessation of smoking, Eating out of home in relation to Anthropometry, FU=follow-up, HPFS = Health Professionals Follow-Up Study, M=men, N=number of participants, NHS = Nurses' Health Study, NHS II = Nurses' Health Study II, Sun = Seguimiento Universidad de Navarra study, UK=United Kingdom, USA=United States of America, vSFFQ=validated, semi-quantitative food frequency questionnaire, W=women, yr=year.

.

Supplementary Table 5c. Characteristics of prospective cohort studies assessing dietary tree nut and peanut intake and incidence of ≥5 kg weight gain (3 cohorts, N=195,595).

Study, Reference	Cohort	Country	N	Age range, yr[a]	Mean FU duration, yr	No. cases	Outcome assessment method	Diet assessment method	Exposure	Lowest tile, (g/d)	Highest tile (g/d)	Funding source
Bes-Rastrollo et al. 2007	SUN	Spain	8865 (3700 M, 5165 W)	18-101	2.3	937	Self-reported	vSFFQ	Walnuts, almonds, hazelnuts, and peanuts	<3.3	≥21.4	Agency
El-Amari et al. 2016	AHS-2	USA	41845 (14437 M, 27408 W)	30-112	8	7,553	Self-reported	vSFFQ	Tree nuts and peanuts	0.2	3	Agency
Liu et al. 2019	NHS<br>NHS II<br>HPFS	USA	144885 (27521 M, 117364 W)	35-55<br>24-44<br>40-75	24<br>20<br>24	79283	Self-reported	vSFFQ	Tree nuts and peanuts	0	>14 g/d	Agency

[a]Based on baseline age range.

AHS-2=Adventist Health Study 2, d=day, FU=follow-up, HPFS = Health Professionals Follow-Up Study, N=number of participants, NHS = Nurses' Health Study, NHS II = Nurses' Health Study II, No. = number, Sun = Seguimiento Universidad de Navarra study, USA=United States of America, vSFFQ=validated, semi-quantitative food frequency questionnaire, yr=year.

Supplementary Table 5d. Characteristics of prospective cohort studies assessing dietary tree nut and peanut intake and incidence of waist circumference increasing above recommendation (2 cohorts, N=9,887).

Study, Reference	Cohort	Country	N	Age range, yr[a]	Mean FU duration, yr	No. cases	Outcome assessment method	Diet assessment method	Exposure	Lowest tile, (g/d)	Highest tile (g/d)	Funding source
Fernández-Montero et al. 2013 (Men)	SUN	Spain	3877 M	18-101	6	1940	Self-reported	vSFFQ	Walnuts, almonds, hazelnuts, peanuts	<3.3[2]	≥21.4[b]	Agency
Fernández-Montero et al. 2013 (Women)	SUN	Spain	6010 W	18-101	6	2350	Self-reported	vSFFQ	Walnuts, almonds, hazelnuts, peanuts	<3.3[2]	≥21.4[b]	Agency

[a]Based on baseline age range.
[b]Nut intake based on the SUN report published by Bes-Rastrollo et al. 2007**.**
d=day, FU=follow-up, N=number of participants, No. = number, Sun = Seguimiento Universidad de Navarra study, vSFFQ=validated, semi-quantitative food frequency questionnaire, yr=year.

Supplementary Table 6a. Analysis of confounding variables among prospective cohort studies assessing dietary tree nut and peanut intake and overweight or obesity incidence.

Cohort	SUN	AHS-2	EPIC-PANACEA	NHS, NHS II, HPFS
Reference	Bes-Rastrollo et al. 2007	El-Amari et al. 2016	Freisling et al. 2018	Liu et al. 2019
Number of variables in fully adjusted model	9	N/A[a]	11	19
Number of multivariable models presented	5		1	1
Number of pre-specified confounding variables which were evaluated (of 6)	6		6	3
**Main confounding variables of consideration**				
Energy intake	√		√	
Age	√		√	√
Sex	√		√	
Physical activity	√		√	√
Smoking	√		√	√
Baseline BMI or Body Weight	√		√	
**Other confounding variables**				
Alcohol Use				√
Fiber Intake				
Fruit Intake				√
Fruit Juice Intake				
VegTable Intake				√
Legume Intake				
Processed Meats Intake				√
Unprocessed Red Meat Intake				√
Fish Intake				
Grain Intake				
Whole Grain Intake				√
Refined Grain Intake				√
Sugar-Sweetened Beverage Intake				√
Sweets and Desserts Intake				√
Potato Intake				√
Potato Chip Intake				
French Fry Intake				√
Dairy Intake				
Whole-Fat Dairy Intake				
Low-Fat Dairy Intake				
Snacking (yes, no)	√			√
Fast Food Intake				
Plausibility of dietary energy reporting			√	
Mediterranean diet score (without fruit and nut component)			√	
Family History				
Menopausal status				√
Hormone therapy use				√
Sleep Duration				√
Sitting Duration				√
Medications				
Television Watching (h/wk)	√			
Education (illiterate and primary school/high school/university)			√	
Country/Centre			√	
Residency (urban/rural)				
Follow-up time in years			√	

AHS-2=Adventist Health Study 2, EPIC -PANACEA= European Prospective Investigation into Cancer and Nutrition – Physical Activity, Nutrition, Alcohol, Cessation of smoking, Eating out of home in relation to Anthropometry, HPFS = Health Professionals Follow-Up Study, NHS = Nurses' Health Study, NHS II = Nurses' Health Study II, Sun = Seguimiento Universidad de Navarra study.
[a]El-Amari et al did not report whether confounding variables were adjusted for as only a published abstract is available.

Supplementary Table 6b. Analysis of confounding variables among prospective cohort studies assessing dietary tree nut and peanut intake and body weight change.

Cohort	SUN	EPIC-PANACEA	NHS	NHS II	HPFS
**Reference**	**Bes-Rastrollo et al. 2007**	**Freisling et al. 2018**	**Smith et al. 2015**	**Smith et al. 2015**	**Smith et al. 2015**
Number of variables in fully adjusted model	9	11	20	20	20
Number of multivariable models presented	5	1	1	1	1
Number of pre-specified confounding variables which were evaluated (of 6)	6	6	5	5	5
**Pre-specified primary confounding variable**					
Energy intake	√	√			
Age	√	√	√	√	√
Sex	√	√	√	√	√
Physical activity	√	√	√	√	√
Smoking	√	√	√	√	√
Baseline BMI or Body Weight	√	√	√	√	√
**Other confounding variables**					
Alcohol Use			√	√	√
Fiber Intake	√				
Fruit Intake			√	√	√
Fruit Juice Intake			√	√	√
VegTable Intake			√	√	√
Legume Intake					
Processed Meats Intake			√	√	√
Unprocessed Red Meat Intake			√	√	√
Fish Intake					
Grain Intake					
Whole Grain Intake			√	√	√
Refined Grain Intake			√	√	√
Sugar-Sweetened Beverage Intake			√	√	√
Sweets and Desserts Intake			√	√	√
Potato Intake			√	√	√
Potato Chip Intake			√	√	√
French Fry Intake					
Dairy Intake					
Whole-Fat Dairy Intake			√	√	√
Low-Fat Dairy Intake			√	√	√
Snacking (yes, no)	√				
Fast Food Intake					
Plausibility of dietary energy reporting		√			
Mediterranean diet score (without fruit and nut component)		√			
Family History					
Menopausal status					
Hormone therapy use					
Sleep Duration			√	√	√
Sitting Duration					
Medications					
Television Watching (h/wk)	√		√	√	√
Education (illiterate and primary school/high school/university)		√			
Country/Centre		√			
Residency (urban/rural)					
Follow-up time in years		√			

EPIC -PANACEA= European Prospective Investigation into Cancer and Nutrition – Physical Activity, Nutrition, Alcohol, Cessation of smoking, Eating out of home in relation to Anthropometry, HPFS = Health Professionals Follow-Up Study, NHS = Nurses' Health Study, NHS II = Nurses' Health Study II, Sun = Seguimiento Universidad de Navarra study.

Supplementary Table 6c. Analysis of confounding variables among prospective cohort studies assessing dietary tree nut and peanut intake and incidence of ≥5 kg weight gain.

Cohort	SUN	AHS-2	NHS, NHS II, HPFS
**Reference**	**Bes-Rastrollo et al. 2007**	**El-Amari et al. 2016**	**Liu et al. 2019**
Number of variables in fully adjusted model	9	N/A	20
Number of multivariable models presented	5		1
Number of pre-specified confounding variables which were evaluated (of 6)	6		4
**Pre-specified primary confounding variable**			
Energy intake	√		
Age	√		√
Sex	√		
Physical activity	√		√
Smoking	√		√
Baseline BMI or Body Weight	√		√
**Other confounding variables**			
Alcohol Use			√
Fiber Intake	√		
Fruit Intake			√
Fruit Juice Intake			
VegTable Intake			√
Legume Intake			
Processed Meats Intake			√
Unprocessed Red Meat Intake			√
Fish Intake			
Grain Intake			
Whole Grain Intake			√
Refined Grain Intake			√
Sugar-Sweetened Beverage Intake			√
Sweets and Desserts Intake			√
Potato Intake			√
Potato Chip Intake			
French Fry Intake			√
Dairy Intake			
Whole-Fat Dairy Intake			
Low-Fat Dairy Intake			
Snacking (yes, no)	√		√
Fast Food Intake			
Plausibility of dietary energy reporting			
Mediterranean diet score (without fruit and nut component)			
Family History			
Menopausal status			√
Hormone therapy use			√
Sleep Duration			√
Sitting Duration			√
Medications			
Television Watching (h/wk)	√		
Education (illiterate and primary school/high school/university)			
Country/Centre			
Residency (urban/rural)			
Follow-up time in years			

AHS-2= Adventist Health Study 2, HPFS = Health Professionals Follow-Up Study, NHS = Nurses' Health Study, NHS II = Nurses' Health Study II, Sun = Seguimiento Universidad de Navarra study.

Supplementary Table 6d. Analysis of confounding variables among prospective cohort studies assessing dietary tree nut and peanut intake and incidence of waist circumference increasing above recommendation.

**Cohort**		**SUN - Men**	**SUN - Women**
**Study**		**Fernández-Montero et al. 2013**	**Fernández-Montero et al. 2013**
Number of variables in fully adjusted model		7	7
Number of multivariable models presented		1	1
Number of pre-specified confounding variables which were evaluated (of 6)		6	6
**Pre-specified primary confounding variable**			
	Energy intake	√	√
	Age	√	√
	Sex	√	√
	Physical activity	√	√
	Smoking	√	√
	Baseline BMI or Body Weight	√	√
**Other confounding variables**			
	Alcohol Use	√	√
	Fiber Intake		
	Fruit Intake		
	Fruit Juice Intake		
	VegTable Intake		
	Legume Intake		
	Processed Meats Intake		
	Unprocessed Red Meat Intake		
	Fish Intake		
	Grain Intake		
	Whole Grain Intake		
	Refined Grain Intake		
	Sugar-Sweetened Beverage Intake		
	Sweets and Desserts Intake		
	Potato Intake		
	Potato Chip Intake		
	French Fry Intake		
	Dairy Intake		
	Whole-Fat Dairy Intake		
	Low-Fat Dairy Intake		
	Snacking (yes, no)		
	Fast Food Intake		
	Plausibility of dietary energy reporting		
	Mediterranean diet score (without fruit and nut component)		
	Family History		
	Menopausal status		
	Hormone therapy use		
	Sleep Duration		
	Sitting Duration		
	Medications		
	Television Watching (h/wk)		
	Education (illiterate and primary school/high school/university)		
	Country/Centre		
	Residency (urban/rural)		
	Follow-up time in years		

Sun = Seguimiento Universidad de Navarra study

Supplementary Table 7. Characteristics of randomized controlled trials assessing dietary tree nut and peanut intake and adiposity outcomes (114 trial comparisons, N=5,873).

Trial	Health Status	Age Range (yrs)	N[a]	Design	Duration (wks)[b]	Setting	BMI (kg/m^2)	Body Weight (kg)	Nut Type, Dose	Comparator	Feeding Control	Energy Balance[c]	Diet Composition % (C:F:P)	Wt. Main[d]	Funding Source
Abazarfard et al. 2014	OW or OB	20-55	108 [100] (0 M:100 F)	P	12	Iran, OP	29.6 ± 1.5	76.0 ± 2.6			Suppl	Negative, Substitution		N	Agency
Almond			54 [50] (0 M:50 F)				29.9 ± 1.2	76.4 ± 2.7	Almonds, 50 g/d				54:30:16		
Control			54 [50] (0 M:50 F)				29.4 ± 1.7	75.6 ± 2.4		Meat and fat exchange lists			54:30:16		
Abbaspour et al. 2019	OW or OB	18-55	54 [48] (32 M:22 F)	P	8	USA, OP					Suppl	Neutral, Substitution		N	Agency
Mixed nut			24 (14 M:10 F)				30.9 ± 2.8	90.3 ± 13.8	Mixed nuts, 42.5 g/d				39:43:18		
Pretzel			24 (15 M:9 F)				31.6 ± 3.1	95.1 ± 12.2		Pretzels, unsalted			46:33:23		
Agebratt et al. 2016	H	18-76	30 [30] (18 M:12 F)	P	8	Sweden, OP	22.3 ± 1.9	70.0 ± 7.7			DA	Positive, Substitution	NR	N	Agency
Mixed Nut			15 [15] (11 M:4 F)				22.5 ± 2.3	73.6 ± 9.0	Mixed nuts, ~88 g/d (7 kcal/day/kg BW)						
Mixed Fruit			15 [15] (7 M:8 F)				22.2 ± 1.6	66.5 ± 8.7		Fruit, 7 kcal/kg weight/day					
Baer et al. 2019	H	25-75	42 [40] (20 M:20 F)	C	4	USA, OP	29.0 ± 4.4	84.2 ± 17.7			MC	Neutral, Substitution		Y	Agency-Industry
Cashew									Cashew nuts, 42 g/d				47:36:17		
Control										Mixed macronutrient			50:33:17		
Balci et al. 2012	Pre-DM/ MetS	NR	60 [NR] (27 M:33 F)	P	12	NR, NR					NR	NR, NR	NR	NR	NR
Walnut			30				NR ± NR	90.8 ± 12.0	Walnuts, 10 g/d						
Control			30				NR ± NR	88.9 ± 16.1		Mixed macronutrient					
Bamberger et al. 2017	H	>50	204 [194] (60 M:134 F)	C	8	Germany, OP	25.4 ± 4.1	71.8 ± 12.1			Suppl	Neutral, Substitution		N	Industry
Walnut									Walnuts, 43 g/d				45:40:15		
Control										Carbohydrate or Fat or Both			48:36:16		
Barbour et al. 2015	OW or OB	50-75	69 [61] (29 M:32 F)	C	12	Australia, OP	30.6 ± 4.1	87.7 ± 14.1			Suppl			N	Agency-Industry
High-oleic peanuts									Peanuts, 15-20% E (M:84 g/d for 6 d/week; W:56 g/d for 6 d/week)			Positive, Addition	37:38:19		
Control										Mixed macronutrient		Neutral, Substitution	42:32:19		

Supplementary Table 7. Characteristics *continued*.

Trial	Health Status	Age Range (yrs)	N[a]	Design	Duration (wks)[b]	Setting	BMI (kg/m^2)	Body Weight (kg)	Nut Type, Dose	Comparator	Feeding Control	Energy Balance[c]	Diet Composition % (C:F:P)	Wt. Main[d]	Funding Source
Bento et al. 2014	DL	21-57	25 [20] (8 M:12 F)	C	6	Brazil, OP	23.2 ± 2.2	63.9 ± 10.9			Suppl	Neutral, Substitution		N	Agency
Almond							23.2 ± 2.2	64.0 ± 10.7	Almonds, 20 g/d				50:33:17		
Control							23.1 ± 2.2	63.7 ± 11.6		One corn starch capsule/d			50:32:18		
Berryman et al. 2015	DL	30-65	61 [48] (22 M:26 F)	C	6	USA, OP	26.2 ± 2.8	74.7 ± 10.4			MC	Neutral, Substitution		Y	Agency-Industry
Almond									Almonds, 42.5 g/d				51:32:16		
Muffin										Isocaloric Muffin,			58:26:15		
Bitok et al. 2018	H	63-79	356 [317] (155 M:201 F)	P	104	USA & Spain, OP	27.6 ± 4.9	77.0 ± 16.8			Suppl	Neutral, Addition		N	Industry
Walnut							27.5 ± 4.8	77.1 ± 17.2	Walnuts, 43 g/d (28-56 g/d)				44:41:15		
Control							27.4 ± 4.8	75.6 ± 16.1		Mixed macronutrient			48:36:16		
Biude Silva Duarte et al. 2019[2]	OB	18-55	72 [55] (0 M:55 F)	P	8	Brazil, OP					Suppl			NR	Agency
Brazil nut			29				34.6 (30.8-37.4)	90.3 (85.3-101.6)	Brazil nuts, 5 g/d			Positive, Addition	51:31:18		
Control			26				34.8 (33.1-40.2)	88.6 (81.7-103.5)		Mixed macronutrient		Negative, Subtraction	52:31:17		
Bowen et al. 2019	OW or OB	20-70	95 [76] (45 M:31 F)	P	8	Australia, OP					Suppl	Neutral, Substitution		N	Industry
Almond							34.4 ± 6.2	102.0 ± 18.5	Almonds, 56 g/d				39:36:18		
Biscuit							33.2 ± 4.9	95.8 ± 18.4		Biscuit,			32:40:20		
Campbell et al. 2019	OW or OB	30-65	29 [17] (9 M:8 F)	P	24	USA, OP	NR ± NR				Suppl	Neutral, Substitution	NR	N	Industry
Almond								84.3 ± 8.1	Almonds, ~62 g/d (17.5 %E)						
Cereal bar								89.1 ± 12.9		Low-fat/high-carbohydrate cereal bar					

Supplementary Table 7. Characteristics *continued*.

Trial	Health Status	Age Range (yrs)	N[a]	Design	Duration (wks)[b]	Setting	BMI (kg/m²)	Body Weight (kg)	Nut Type, Dose	Comparator	Feeding Control	Energy Balance[d]	Diet Composition % (C:F:P)	Wt. Main[d]	Funding Source
Canales et al. 2007[3]	OW or OB	M:≥45 W:≥50	25 [22] (12 M:10 F)	C	5	Spain, OP	29.6 ± 3.4	81.0 ± 12.9			Suppl	Neutral, Substitution		N	Agency
Walnut									Walnuts, 19.4 g/d				30:47:18		
Control										Restructured steaks and sausages			33:40:19		
Carughi et al. 2019	H	23-49	NR [60] (0 M:60 F)	P	4	France, OP	21.6 ± 1.7				Suppl	Neutral, Substitution	NR	N	Industry
Pistachio			30					58.5 ± 6.0	Pistachios, 56 g/d						
Biscuit			30					57.3 ± 5.8		Gouda aperitif biscuits					
Casas-Agustench et al. 2011	MetS	18-65	52 [50] (28 M:22F)	P	12	Spain, OP	30.8 ± 3.1	83.2 ± 12.5			Suppl	Neutral, Addition		N	Agency-Industry
Mixed nut			27 [25]						Mixed Nuts, 30 g/d				42:36:19		
Control			25 [25]							Mixed macronutrient			46:21:31		
Chisholm et al. 2005	DL	25-70	28 [NR] (5 M:23 F)	C	6	New Zealand, OP	26.9 ± 3.2	74.2 ± 11.5			Suppl	Neutral, Substitution		N	Agency
Mixed nuts							26.8 ± 3.2	74.2 ± 11.6	Mixed Nuts, 30 g/d				46:33:16		
Canola oil based cereal							26.8 ± 3.4	74.1 ± 11.7		Cereal containing Canola oil			48:30:16		
Ciccone et al. 2014	OW or OB	18-70	47 [47] (24 M:23 F)	P	72	Italy, OP	33.9 ± 5.1	93.4 ± 14.3			DA	NR, Substitution	NR	NR	NR
Nut									Nuts, undefined, 15 nuts per week						
Fish										Non-fried fish					
Olive oil										Olive oil					
Control										Mixed macronutrient					
Cohen et al. 2011	T2DM	>50	13 [13] (7 M:6 F)	P	12	USA, OP	34.8 ± 7.6	100.9 ± 24.7			Suppl	NR, NR	NR	N	Agency
Almond							32.6 ± 8.3	96.1 ± 32.1	Almonds, 28 g/5 d/wk						
Cheese							36.7 ± 13.0	105.1 ± 40.4		Cheese sticks					
Damasceno et al. 2011	DL	25-75	26 [18] (9 M:9 F)	C	4	Spain, OP	25.7 ± 2.3	70.7 ± 17.3			Suppl	Neutral, Substitution		N	Agency-Industry
Almond									Almonds, 50-75 g/d				49:33:17		
Walnut									Walnuts, 40-65 g/d				50:32:17		
Olive oil										Virgin olive oil			49:33:16		

Supplementary Table 7. Characteristics *continued*.

Trial	Health Status	Age Range (yrs)	N[a]	Design	Duration (wks)[b]	Setting	BMI (kg/m²)	Body Weight (kg)	Nut Type, Dose	Comparator	Feeding Control	Energy Balance[c]	Diet Composition % (C:F:P)	Wt. Main[d]	Funding Source
Damavandi et al. 2012	T2DM	35-70	50 [43] (9 M:34 F)	P	8	Iran, OP	28.6 ± 4.5	72.0 ± 11.1			Suppl	Neutral, Substitution		N	Agency
Cashew							28.7 ± 5.8	72.1 ± 13.2	Cashew nuts, ~28 g/d				59:28:15		
Control							28.6 ± 3.1	71.9 ± 9.8		Mixed macronutrient			58:27:16		
Damavandi et al. 2013	T2DM	35-70	50 [48] (15 M:33 F)	P	8	Iran, OP	28.3 ± 3.5	72.1 ± 9.7			Suppl	Neutral, Substitution		Y	Agency
Hazelnut			25				28.5 ± 3.6	72.1 ± 10.3	Hazelnuts, 29 g/d				55:31:16		
Control			25				28.2 ± 3.6	72.0 ± 9.6		Mixed macronutrient			60:25:17		
de Souza et al. 2018	OW or OB	20-59	60 [46] (0 M:46 F)	P	8	Brazil, OP					Suppl	Neutral, Substitution		N	Agency
Almonds							32.5 ± 4.4	81.9 ± 14.4	Almonds, 20 g/d				48:33:19		
Control							33.3 ± 4.7	83.6 ± 13.5		Maltodextrin			56:24:21		
Dhillon et al. 2016	OW or OB	18-60	86 [79] (21 M:65 F)	P	12	USA, OP					Suppl	Negative, Substitution	NR	N	Industry
Almond			43 (11M:32 F)				29.9 ± 3.2	82.8 ± 12.9	Almonds, 15% Energy						
Control			43 (10 M:33 F)				40.0 ± 4.5	84.7 ± 14.1		Mixed macronutrient					
Dhillon et al. 2018	H	18-19	80 [73] (32 M:41 F)	P	8	USA, OP	25.5 ± 4.7	71.4 ± 16.8			Suppl	Neutral, Substitution		N	Industry
Almond							25.6 ± 5.0	71.5 ± 18.6	Almonds, 56.7 g/d (2 oz)				43:42:17		
Cracker							25.3 ± 4.5	71.3 ± 15.1		Graham crackers			54:34:14		
Foster et al. 2012	OW or OB	18-75	123 [123] (11 M:112 F)	P	72	USA, OP	34.0 ± 3.6	92.7 ± 12.5			Suppl	Negative, NR	NR	N	Industry
Almond			61 [61]				33.9 ± 3.5	94.0 ± 13.1	Almonds, 56 g/d						
Control			62 [62]				34.0 ± 3.7	91.5 ± 11.9		Mixed macronutrient					
Gebauer et al. 2008	H	31-65	29 [28] (10 M:18 F)	C	4	USA, OP	26.8 ± 3.7	76.8 ± 13.8			MC	Neutral, Substitution		Y	Agency-Industry
Pistachios (20% E)									Pistachios, 63-126 g/d				54:34:17		
Pistachios (10% E)									Pistachios, 32-63 g/d				58:30:17		
Control										Pretzels and baked potato chips			63:25:15		

Supplementary Table 7. Characteristics *continued*.

Trial	Health Status	Age Range (yrs)	N[a]	Design	Duration (wks)[b]	Setting	BMI (kg/m^2)	Body Weight (kg)	Nut Type, Dose	Comparator	Feeding Control	Energy Balance[c]	Diet Composition % (C:F:P)	Wt. Main[d]	Funding Source
Gulati et al. 2014	MetS	NR	68 [60] (37 M:31 F)	P	24	India, OP	NR ± NR	80.9 ± 11.5			Suppl/ DA	Neutral, Substitution		N	Industry
Pistachio								81.6 ± 12.9	Pistachios, 49 g/d (20% kcal/d)				51:29:20		
Control								80.3 ± 10.3		Visible fat, a portion of carbohydrates , and dairy.			60:25:15		
Hernández-Alonso et al. 2014	Pre-DM	25-65	54 [46] (29 M:25 F)	C	16	Spain, OP	28.9 ± 2.4	77.6 ± 9.5			Suppl	Neutral, Substitution	NR	N	Industry
Pistachio									Pistachios, 57 g/d						
Control										Olive oil					
Hiraoka-Yamamoto et al. 2004	H	18-24	71 [NR] (0 M:71 F)	P	3	Japan, OP					Suppl	Positive, Addition	NR	N	NR
Macadamia nut							19.9 ± 2.0	49.4 ± 5.9	Macadamia nuts, 20 g/d						
Butter							19.9 ± 2.4	49.9 ± 6.2		Butter					
Coconut							21.0 ± 12.0	51.6 ± 5.9		Coconut					
Hollis and Mattes, 2007	H	NR	24 [20] (NR)	C	10	USA, OP	25.9 ± 3.1	70.2 ± 10.1			Suppl	Neutral, Addition		N	Industry
Almond								70.4 ± 9.0	Almonds, ~58 g/d				45:40:16		
Control								69.5 ± 10.5		Mixed macronutrient			53:32:16		
Hudthagosol et al. 2012	H	23-65	27 [25] (13 M:12 F)	C	4	USA, OP	24.8 (18.7-36.6)	70.9 (51.5-115.8)			MC	Neutral, Substitution		Y	Industry
Walnut									Walnuts, 42.5 g, 6 d/wk				60:31:15		
Salmon										Salmon			58:29:15		
Control										Meats and dairy			58:30:15		
Hwang et al. 2019	MetS	30-55	119 [84] (42 M:42 F)	C	16	Korea, OP	27.1 ± 3.6	76.1 ± 14.9			Suppl	Neutral, Substitution		Y	Industry
Walnut							27.9 ± 3.8	78.6 ± 17.0	Walnuts, 45 g/d				53:30:16		
White bread							26.3 ± 3.3	73.1 ± 12.2		White bread			NR		
Jamshed et al. 2015	CAD	32-86	150 [113] (113 M:37 F)	P	12	Pakistan , OP	NR ± NR	76.0 ± 12.0	Almonds, 10 g/d		Suppl	NR, Addition		N	Agency
Almonds (Pakistani)								79.0 ± 1.4					25:12:54		
Almonds (American)								75 ± 1.4					31:16:61		
Control								73.4 ± 1.4		Mixed macronutrient			39:38:46		

Supplementary Table 7. Characteristics *continued*.

Trial	Health Status	Age Range (yrs)	N[a]	Design	Duration (wks)[b]	Setting	BMI (kg/m²)	Body Weight (kg)	Nut Type, Dose	Comparator	Feeding Control	Energy Balance[c]	Diet Composition % (C:F:P)	Wt. Main[d]	Funding Source
Jenkins et al. 2002	DL	48-86	43 [27] (15 M:12 F)	C	4	Canada, OP	25.7 ± 3.0	71.1 ± 12.6			Suppl	Neutral, Substitution		Y	Industry
Almond [High dose]								71.2 ± 13.0	Almonds, 73 g/d				45:36:17		
Almond [Low dose]								71.1 ± 12.5	Almonds, 37 g/d				48:32:18		
Muffin								71.0 ± 12.5		Muffins			55:26:18		
Jenkins et al. 2018	T2DM	>21	117 [103] (66 M:34 F)	P	12	Canada, OP	29.0 ± 4.4	81.7 ± 14.7			Suppl	Neutral, Substitution		N	Agency-Industry
Mixed nut [High dose]			40 [39]				28.8 ± 4.5	80.0 ± 14.7	Mixed nuts, 75 g/d (50-100 g/d)				39:42:18		
Mixed nut [Low dose]			38 [32]				30.3 ± 5.0	86.2 ± 15.6	Mixed nuts, 37.5 g/d (25-50 g/d)				41:39:19		
Control			39 [32]				29.4 ± 4.2	82.9 ± 14.7		Muffins			44:36:20		
Johnston et al. 2013	OW or OB	20-65	64 [44] (16 M:28 F)	P	8	USA, OP	NR ± NR	84.9 ± 12.4			Suppl	NR (Neutral or Positive), Addition		N	Industry
Peanut								87.0 ± 18.7	Peanuts, 28 g/d				54:30:16		
Grain bar								82.5 ± 17.0		Grain bar			52:33:16		
Jung et al. 2018	OW or OB	45-69	90 [84] (11 M:73 F)	C	4	Korea, OP	25.4 ± 2.0	66.3 ± 8.6			Suppl	Positive, Addition		N	Agency-Industry
Almond							25.4 ± 2.0	66.3 ± 8.7	Almonds, 56 g/d				55:32:15		
Cookie							25.4 ± 2.1	66.2 ± 8.7		Homemade cookies			61:26:14		
Katz et al. 2012	MetS	30-75	46 [40] (18 M:28 F)	C	8	USA, OP	33.2 ± 4.4	NR ± NR			Suppl	Neutral, Substitution		Y	Industry
Walnut									Walnuts, 56 g/d				41:41:17		
Control										Mixed macronutrient			45:34:20		
Kocyigit et al. 2006	H	NR	44 [44](24 M:20 F)	P	3	Turkey, OP		NR ± NR			Suppl	Neutral, Substitution		N	Agency
Pistachio							24.2 ± 6.1		Pistachios, ~70 g/d (65-75 g/d)				45:40:17		
Control							24.6 ± 5.6			Other fat sources			46:38:18		
Le et al. 2016	OW or OB	≥21	245 [213] (0 M:213 F)	P	24	USA, OP	33.5 ± 3.3	89.9 ± 11.0			Suppl	Negative, Substitution		N	Agency-Industry
Walnut			82 [71] (0 M:71 F)				33.6 ± 3.3	90.0 ± 11.8	Walnuts, 42 g/d				45:35:20		
High fat			81 [66] (0 M:66 F)				33.6 ± 3.3	90.0 ± 12.6		MUFA			45:35:20		
High carbohydrate			82 [76] (0 M:76 F)				33.2 ± 3.3	89.7 ± 10.9		VegTables, fruits and whole grains			65:20:15		

Supplementary Table 7. Characteristics *continued*.

Trial	Health Status	Age Range (yrs)	N[a]	Design	Duration (wks)[b]	Setting	BMI (kg/m^2)	Body Weight (kg)	Nut Type, Dose	Comparator	Feeding Control	Energy Balance[c]	Diet Composition % (C:F:P)	Wt. Main[d]	Funding Source
Lee et al. 2014	MetS	35-65	61 [60] (NR)	P	6	South Korea, OP	27.1 ± 2.1	73.0 ± 11.0			Suppl	Neutral, Addition		N	Agency
Mixed nut			30 [30] (NR)				27.0 ± 2.2	73.0 ± 11.1	Mixed nuts, 30 g/d				53:29:15		
Control			31 [30] (NR)				27.2 ± 2.1	73.0 ± 11.3		Mixed macronutrient			59:22:16		
Li et al. 2010	OW or OB	20-65	70 [52] (13 M:57 F)	P	12	USA, OP					Suppl	Negative, Substitution		N	NR
Pistachio			27				30.1 ± 2.1	86.0 ± 16.6	Pistachios, 53 g/d				55:30:15		
Pretzel			25				30.9 ± 2.0	85.5 ± 24.0		Salted pretzels			65:20:15		
Li et al. 2011	T2DM	NR	22 [20] (9M:11F)	C	4	Taiwan, OP	26.0 ± 3.1	NR ± NR			MC	Neutral, Substitution		Y	Agency-Industry
Almond									Almonds, 56 g/d (20% kcal/d)				47:37:17		
Control										Mixed macronutrient			57:27:17		
Liu et al. 2018	H	20-39	169 [85] (45 M:40 F)	P	20	Korea, OP					Suppl	Neutral, Substitution		N	Industry
Almond							23.0 ± 3.2	64.5 ± 13.2	Almonds, 56 g/d				49:37:15		
Control							21.7 ± 3.1	62.3 ± 11.8		Carbohydrate			54:31:14		
Ma et al. 2010	T2DM	30-75	24 [21] (10 M:14 F)	C	8	USA, OP	32.5 ± 5.0	89.0 ± 15.5			Suppl	Neutral, Substitution		Y	NR
Walnut									Walnuts, 56 g/d				39:45:17		
Control										Mixed macronutrient			43:38:19		
McKay et al. 2018	OW or OB	≥45	26 [26] (21 M:5 F)	C	4	USA, OP	29.2 ± 2.4	NR ± NR			Suppl	Neutral, Substitution		Y	Agency-Industry
Pecan									Pecans, ~42.5 g/d				48:35:16		
Control										Mixed macronutrient			48:36:16		
Mohan et al. 2018	T2DM	30-65	300 [269] (145 M:124 F)	P	12	India, OP	25.9 ± 3.2	67.4 ± 8.8			Suppl	Neutral, Subsitution		N	Industry
Cashew							25.6 ± 2.8	67.6 ± 9.1	Cashew nuts, 30 g/d				58:30:12		
Control							26.2 ± 3.9	67.3 ± 11.5		Carbohydrate			61:27:12		
Moreira Alves et al. 2014	OW or OB	18-50	76 [65] (65 M:0 F)	P	4	Brazil, OP					Suppl	Negative, Substitution		N	Agency-Industry
High-oleic peanuts			21				29.9 ± 2.7	95.1 ± 11.0	Peanuts, 56 g/d				55:30:15		
Conventional peanuts			22				29.5 ± 1.9	93.4 ± 10.3	Peanuts, 56 g/d				55:30:15		
Control			22				29.7 ± 2.8	94.5 ± 11.7		Mixed macronutrient			55:30:15		

Supplementary Table 7. Characteristics *continued*.

Trial	Health Status	Age Range (yrs)	N[a]	Design	Duration (wks)[b]	Setting	BMI (kg/m²)	Body Weight (kg)	Nut Type, Dose	Comparator	Feeding Control	Energy Balance[c]	Diet Composition % (C:F:P)	Wt. Main[d]	Funding Source
Morgan et al. 2000	H	NR (mean 45±10)	23 [19] (4 M:15 F)	P	8	USA, OP	24.0 ± 3.9	64.9 ± 8.9			Suppl			N	Agency-Industry
Pecan			9 (1 M:8 F)				24.0 ± 5.0	64.0 ± 12.0	Pecan, 68 g/d			Positive, Addition	45:43:12		
Control			10 (3 M:7 F)				24.0 ± 4.0	66.0 ± 12.0		Mixed macronutrient		Negative, Addition	47:37:18		
Morgan et al. 2002	H or DL	NR (mean 55.7±11.8)	49 [42] (17 M:25 F)	C	6	USA, OP	27.4 ± 5.8	NR ± NR			Suppl	Neutral, Addition		Y	NR
Walnut									Walnuts, 64 g/d				44:41:15		
Control										Mixed macronutrient			51:33:15		
Nagashree et al. 2017	H	18-40	58 [58] (31 M:27 F)	P	13	India, OP	21.3 ± 2.0	58.2 ± 6.1			MC	Neutral, Substitution		N	NR
Peanut									Peanut, 100 g/d				57:30:13		
Coconut										Coconut			58:30:11		
Njike et al. 2015	Pre-DM	25-75	112 (97) [31 M:81 F]	C	24	USA, OP	30.2 ± 4.1	83.6 ± 14.1			Suppl	Positive, Addition	NR	N	Industry
Walnuts + Ad libitum diet									Walnut, 56 g/d						
Ad libitum diet										Mixed macronutrient					
					24		30.0 ± 4.0	180.4 ± 31.7				Neutral, Substitution	NR		
Walnuts + Calorie adjusted diet									Walnut, 56 g/d						
Calorie adjusted diet										Mixed macronutrient					
Njike et al. 2017	OW or OB	25-75	34 [32] (13 M:21 F)	P	12	USA, OP			Mixed Nuts, ~30 to 120 g/d (1-4 servings or 200-800 kcal/d)		Suppl	Neutral, Substitution	NR	N	Agency-Industry
Nut-based snack bar			17 [16] (5 M:12 F)				34.6 ± 7.2	97.8 ± 23.1							
Conventional snack foods			17 [16] (8 M:9 F)				34.4 ± 5.7	100.4 ± 20.6		Conventional snack foods					

Supplementary Table 7. Characteristics *continued*.

Trial	Health Status	Age Range (yrs)	N[a]	Design	Duration (wks)[b]	Setting	BMI (kg/m²)	Body Weight (kg)	Nut Type, Dose	Comparator	Feeding Control	Energy Balance[c]	Diet Composition % (C:F:P)	Wt. Main[d]	Funding Source
Parham et al. 2014	T2DM	Not reported (51.6± 10.4)	48 [44] (11 M:33 F)	C	12	Iran, OP	31.2 ± 5.5	NR ± NR			Suppl	NR, Addition	NR	N	NR
Pistachio									Pistachio, 50 g/d						
Control										Mixed macronutrient					
Rajaram et al. 2001	H	25-55	23 (14 M:9 F)	C	4	USA, OP	NR ± NR	74.4 ± 16.7			MC	Neutral, Substitution		Y	Industry
Pecan									Pecan, 72 g/2400 kcal/d				47:40:13		
Control										Mixed macronutrient			57:28:15		
Robbins et al. 2012	H	21-35	117 [109] (109 M:0 F)	P	12	USA, OP					Suppl	NR, Addition	NR	N	NR
Walnut			59				25.0 ± 4.0	79.4 ± 16.0	Walnuts, 75 g/d						
Control			58				25.6 ± 3.5	79.1 ± 9.2		Mixed macronutrient					
Rock et al. 2017	OW or OB	>21	100 [97] (42 M:58 F)	P	24	USA, OP	32.4 ± 3.2	91.0 ± 14.4			Suppl	Negative, Substitution	NR	N	Agency-Industry
Walnut			49 (18 M:31 F)				32.4 ± 3.5	91.1 ± 16.1	Walnuts, 28-42 g/d						
Control			51 (24 M:27 F)				32.4 ± 2.9	90.9 ± 12.9		Mixed macronutrient					
Ros et al. 2004	DL	25-75 (after menopause in women)	21 [20] (8 M:12 F)	C	4	Spain, OP	NR ± NR	70.6 ± 10.3			Suppl	Neutral, Substitution		NR	Agency-Industry
Walnut									Walnuts, 40-65 g/d				49:33:17		
Control										Olive oil and other MUFA-rich foods			49:33:16		
Ruisinger et al. 2015	DL	18-78	50 [48] (24 M:24 F)	P	4	USA, OP	29.2 ± 4.3	83.8 ± 13.8			Suppl	Neutral, Substitution		N	Agency-Industry
Almond							29.8 ± 4.8	86.8 ± 13.3	Almonds, 100 g/d				33:49:18		
Control							28.6 ± 3.9	81.3 ± 14.3		Mixed macronutrient			53:31:17		
Sabaté et al. 2003	H	22-53	27 [25] (14 M:11 F)	C	4	USA, OP	NR ± NR	71.0 ± 2.7			MC	Neutral, Substitution		Y	Industry
Almond [High dose]									Almonds, ~83.0 g/d				46:39:14		
Almond [Low dose]									Almonds, ~42.1 g/d				51:35:13		
Control										Mixed macronutrient			56:30:14		

Supplementary Table 7. Characteristics *continued*.

Trial	Health Status	Age Range (yrs)	N[a]	Design	Duration (wks)[b]	Setting	BMI (kg/m²)	Body Weight (kg)	Nut Type, Dose	Comparator	Feeding Control	Energy Balance[c]	Diet Composition % (C:F:P)	Wt. Main[d]	Funding Source
Sabaté et al. 2005	H	30-72	94 [90] (40 M:50 F)	C	24	USA, OP	26.5 ± 3.1	75.7 ± 10.6			Suppl	Neutral, Substitution	NR	Y	Not reported
Walnut							26.1 ± 3.5	73.3 ± 13.1	Walnuts, 35 g/d (12% E)						
Control							26.9 ± 3.3	78.5 ± 15.0		Mixed macronutrient					
Sauder et al. 2015	T2DM	40-74	34 [30] (15 M:15 F)	C	4	USA, OP	31.2 ± 3.1	NR ± NR			MC	Neutral, Substitution		Y	Industry
Pistachio									Pistachios, 93.5 g/d				51:33:17		
Control										Low-fat/fat-free snacks			55:27:18		
Schutte et al. 2006[4]	MetS	21-65	68 [62] (28 M:34 F)	P	8	South Africa, OP	34.8 ± 5.2	102.6 ± 16.2			MC	Neutral, Substitution		Y	Agency-Industry
Cashew							34.4 ± 4.8	99.0 ± 14.8	Cashew nuts, 63-108 g/d (20% kcal/d)				44:37:19		
Walnut							36.0 ± 5.9	107.0 ± 17.1	Walnuts, 63-108 g/d (20% kcal/d)				42:40:18		
Control							35.1 ± 5.2	106.0 ± 15.6		Mixed macronutrient			47:33:20		
Sheridan et al. 2007	DL	36-75	20 [15] (11 M:4 F)	C	4	USA, OP	28.0 ± 3.5	79.4 ± 3.0			Suppl	Neutral, Substitution		N	Industry
Pistachio									Pistachios, 15 % kcal/d (~2-3 oz)				51:31:17		
Control										Fat			52:31:16		
Somerset et al. 2013	OW or OB	26-55	64 [NR] (10 M:54 F)	P	10	Australia, OP					DA	Neutral, Substitution		Y	Industry
Macadamia nuts							33.2 ± 4.7	95.0 ± 14.7	Macadamia nuts, ~28 g/d				36:38:21		
Control							35.8 ± 6.5	99.6 ± 15.2		Saturated fat			41:38:17		
Spaccarotella et al. 2008	H	55-75	22 [21] (21 M:0 F)	C	8	USA, OP	NR ± NR	84.8 ± 2.9			Suppl	Neutral, Substitution		Y	Industry
Walnut									Walnuts, 75 g/d				NR:45:NR		
Control										Fat			NR:36:NR		

Supplementary Table 7. Characteristics *continued*.

Trial	Health Status	Age Range (yrs)	N[a]	Design	Duration (wks)[b]	Setting	BMI (kg/m²)	Body Weight (kg)	Nut Type, Dose	Comparator	Feeding Control	Energy Balance[c]	Diet Composition % (C:F:P)	Wt. Main[d]	Funding Source
Spiller et al. 1998	DL	NR	48 [45] (12 M:33 F)	P	4	USA, OP	NR ± NR	66.0 ± 13.0			Suppl	Positive, Substitution		N	Industry
Almond									Almonds, 100 g/d				44:39:16		
Olive oil										Virgin olive oil with cottage cheese and rye crackers			47:35:17		
Cheddar cheese/butter										Cheddar cheese) with butter and rye crackers			45:35:17		
Sweazea et al. 2014	T2DM	25-75	24 [21] (9 M:12 F)	P	12	USA, OP	35.3 ± 8.1	99.1 ± 24.3			Suppl	Neutral, Addition		N	Industry
Almond							37.2 ± 7.8	106.7 ± 20.6	Almonds, 43 g/d				39:42:19		
Control							33.5 ± 8.8	92.1 ± 27.4		Mixed macronutrient			46:37:17		
Tan and Mattes. 2013	Pre-DM	18-60	150 [137] (48 M:89 F)	P	4	USA, OP			,		Suppl	Neutral, Addition		N	Industry
Almonds [Breakfast]							28.2 ± 4.8	80.5 ± 15.0	Almonds, 43 g/d				45:41:16		
Almonds [Lunch]							29.0 ± 3.9	84.8 ± 13.7	Almonds, 43 g/d				47:38:16		
Almonds [Morning snack]							28.7 ± 5.0	83.2 ± 21.1	Almonds, 43 g/d				47:39:16		
Almonds [Afternoon snack]							28.2 ± 5.2	81.8 ± 14.6	Almonds, 43 g/d				44:41:16		
Control							27.0 ± 4.4	77.2 ± 16.8		Carbohydrate			50:36:15		
Tapsell et al. 2004	T2DM	35-75	58 [55] (34M:24F)	P	24	Australia, OP	30.0 ± 3.7	84.6 ± 10.1			Suppl	Neutral, Substitution		Y	Industry
Walnut + Low Fat/Modified Fat diet							30.7 ± 3.9	87.6 ± 12.8	Walnuts, 30 g/d				44:32:22		
Low fat/Modified fat diet							30.2 ± 4.5	84.6 ± 4.3		Mixed macronutrient			41:33:23		
Low Fat Control							29.2 ± 2.6	81.9 ± 11.2		Mixed macronutrient			43:33:21		

Supplementary Table 7. Characteristics *continued*.

Trial	Health Status	Age Range (yrs)	N[a]	Design	Duration (wks)[b]	Setting	BMI (kg/m²)	Body Weight (kg)	Nut Type, Dose	Comparator	Feeding Control	Energy Balance[c]	Diet Composition % (C:F:P)	Wt. Main[d]	Funding Source
Tapsell et al. 2009	T2DM	33-70	50 [35] (NR M: NR F)	P	52	Australia, OP	33.1 ± 4.2	92.8 ± 4.5			Suppl	Neutral, Substitution		Y	Industry
Walnut			26				33.2 ± 4.4	92.3 ± 15.7	Walnuts, 30 g/d				41:34:21		
Control			24				33.0 ± 4.0	93.4 ± 3.0		Other fat containing foods			42:29:24		
Tapsell et al. 2017	OW or OB	25-54	377 [126] (99 M:278 F)	P	52	Australia, OP					Suppl	Neutral, Substitution	NR	N	Agency-Industry
Walnut + Interdisciplinary Intervention			23				32.6 ± 4.3	91.4 ± 15.5	Walnuts, 30 g/d						
Interdisciplinary Intervention			43				32.6 ± 4.3	91.9 ± 15.2		Mixed macronutrient					
Usual Care			60				32.5 ± 4.1	91.8 ± 14.7		Mixed macronutrient					
Tey et al. 2011	H	18-65	124 [118] (55 M:63 F)	P	12	New Zealand, OP	23.8 ± 3.0	69.5 ± 11.4			Suppl	Neutral, Substitution		N	Agency
Hazelnut			32 (15 M:17 F)				24.6 ± 2.8	72.0 ± 11.1	Hazelnuts, 42 g/d				46:42:15		
Chocolate			31 (17 M:16 F)				23.6 ± 3.3	69.2 ± 13.0		Dairy milk chocolate			50:38:16		
Potato crisps			26 (9 M:17 F)				23.9 ± 3.0	69.5 ± 11.6		Potato crisps			46:34:15		
Control			29 (16 M:13 F)				22.9 ± 2.8	67.3 ± 9.5		No additional food			51:35:17		
Tey et al. 2013	OW or OB	18-65	110 [107] (46 M:61 F)	P	12	New Zealand, OP	30.6 ± 5.1	89.1 ± 16.5			Suppl	Neutral, Addition		N	Agency
Hazelnut [High dose]			37 [37] (17 M:20 F)				30.9 ± 6.0	92.0 ± 19.6	Hazelnuts, 60 g/d				38:42:16		
Hazelnut [Low dose]			35 [33] (16 M:21 F)				30.7 ± 4.7	86.2 ± 11.8	Hazelnuts, 30 g/d				42:39:17		
Control			38 [37] (16 M:21 F)				30.4 ± 4.5	88.7 ± 16.7		Mixed macronutrient			47:33:17		
Tindall et al. 2019	OW or OB	30-65	45 [36] (25 M:20 F)	C	6	USA, OP	30.3 ± 4.7	95.2 ± 18.8			MC	Neutral, Substitution		Y	Agency-Industry
Walnut									Walnuts, 57-99 g/d				48:35:17		
ALA										ALA			48:35:17		
Oleic acid										Oleic acid			48:35:17		

Supplementary Table 7. Characteristics *continued*.

Trial	Health Status	Age Range (yrs)	N[a]	Design	Duration (wks)[b]	Setting	BMI (kg/m²)	Body Weight (kg)	Nut Type, Dose	Comparator	Feeding Control	Energy Balance[c]	Diet Composition % (C:F:P)	Wt. Main[d]	Funding Source
Tsaban et al. 2017	OB or DL	18-70	80 [65] (72 M:8 F)	P	72	Israel, OP	31.2 (29.1-33.9)	94.7 ± 13.5			Suppl	Negative, Substitution	NR	N	Agency
Walnut							31.3 (28.9-34.4)	94.7 ± 14.3	Walnuts, 28 g/d						
Control							31.1 (29.8-33.7)	94.7 ± 12.8		Low fat (higher carbohydrate)					
Vergani et al. 2018	MetS	20-66	38 [NR] (17 M:21 F)	P	12	Italy, OP		NR ± NR			NR	Negative, Substitution	NR	N	NR
Mixed nut			9				34.4 ± 7.2		Mixed nuts, 50 g/d						
Fruits & vegTables			12				34.9 ± 7.3			Carbohydrate					
Control			17				36.6 ± 7.8			Mixed macronutrient					
Wang et al. 2012	MetS	25-65	90 [86] (41 M:49 F)	P	12	China, OP					Suppl	Neutral, Addition	NR	N	Industry
Pistachio [High dose]			30 [29] (12 M:18 F)				28.0 ± 4.5	NR ± NR	Pistachios, 70 g/d						
Pistachio [Low dose]			30 [27] (16 M:14 F)				28.1 ± 3.2		Pistachios, 42 g/d						
Control			30 [30] (13 M:17 F)				28.0 ± 4.4			Mixed macronutrient					
Wien et al. 2003	OW or OB	27-79	65 [52] (28 M:37 F)	P	24	USA, OP					Suppl	Negative, Substitution		N	Agency-Industry
Almond							38.3 ± 1.7	111.2 ± 4.6	Almonds, 84 g/d				32:39:29		
Control							38.4 ± 1.7	111.2 ± 4.7		Self-selected complex carbohydrates			53:18:29		
Wien et al. 2010	Pre-DM	NR	65 [54] (17 M:48 F)	P	16	USA, OP					Suppl	Negative, Substitution		N	Industry
Almond							30.0 ± 5.0	82.9 ± 14.4	Almonds, 60 g/d (20% Energy)			,	42:39:19		
Control							29.0 ± 5.0	80.5 ± 14.4		Meat and fat.		,	48:30:21		

Supplementary Table 7. Characteristics *continued*.

Trial	Health Status	Age Range (yrs)	N[a]	Design	Duration (wks)[b]	Setting	BMI (kg/m²)	Body Weight (kg)	Nut Type, Dose	Comparator	Feeding Control	Energy Balance[c]	Diet Composition % (C:F:P)	Wt. Main[d]	Funding Source
Wien et al. 2014	T2DM	34-84	60 [60] (30 M:30 F)	P	24	USA, OP	32.3 ± 6.8	88.2 ± 22.0			Suppl	Neutral if BMI <25 (10% of participants), Negative if BMI >25 (90% of participants), Substitution		Y	Industry
Peanuts							31.1 ± 6.9	86.0 ± 24.8	Peanuts, 46 g/d (20% Energy)				44:41:19		
Control							33.4 ± 6.8	90.4 ± 19.3		Meat/meat substitutes and fat.			47:37:18		
Williams et al. 2019	OB	≥20	32 [24] (9 M:15 F)	C	3	USA, OP	31.3 ± 3.5	NR ± NR			CF	Neutral, Substitution		Y	Industry
Almond									Almonds, 20% Energy				50:35:15		
High carbohydrate										High carbohydrate			50:35:15		
Low carbohydrate										Lower carbohydrate			25:47:28		
Wilson et al. 2014	OB	NR	22 [22] (6 M:16 F)	P	6	USA, OP	31.1 ± 4.0	NR ± NR	Pistachios, 35.4 g/d (1.25 oz/d)		Suppl	NR, Addition	NA	N	Agency-Industry
Pistachio															
Control										Mixed macronutrient					
Wu et al. 2010	MetS	25-65	283 [277] (158 M:125 F)	P	12	China, OP	25.5 ± 2.7	71.4 ± 11.1			Suppl	Neutral, Substitution		Y	Agency-Industry
Walnut			94 (53 M:41 F)				25.7 ± 2.9	72.2 ± 11.4	Walnuts, 30 g/d				48:37:15		
Flaxseed			94 (53 M:41 F)				25.1 ± 2.3	69.7 ± 9.4		Flaxseed			47:38:16		
Control			95 (52 M:43 F)				25.4 ± 2.4	70.6 ± 10.9		Mixed macronutrient			50:34:15		
Zambon et al. 2000	DL	28-72	55 [49] (28 M:27 F)	C	6	Spain, OP	27.0 ± 3.1	70.6 ± 12.1			Suppl	Neutral, Substitution		NR	Agency-Industry, Agency
Walnut			28 [25] (NR)						Walnuts, 41-56 g/d (18% Energy)				51:33:17		
Control			27 [24] (NR)							MUFA			52:30:18		

[a]N represents the number of participants and is presented as "number randomized [number completed] (number of males: number of females)".
[b]Data was supplemented with information obtained from Biude Silva Duarte et al. 2017, which present the same trial.
[c]Data was supplemented with information obtained from Sanchez-Muniz et al. 2012 and Olmedilla-Alonso et al. 2008, which present the same trial.
[d]Data was supplemented with information obtained from Mukuddem-Petersen et al. 2007, which presents the same trial.
ALA= alpha-linolenic acid, C=crossover, CAD=coronary artery disease, CF= controlled feeding, C:F:P = carbohydrate:fat:protein, CVD=cardiovascular disease, DA= dietary advice, DL=dyslipidemia, H=Healthy, MC= metabolically controlled, MetS=metabolic syndrome, N= Number of participants, NR=not reported, OW=overweight, OB=Obese, OP=outpatient, P=parallel, Pre-DM= prediabetes, Suppl= supplemented, T2DM=type 2 diabetes mellitus, USA=United States of America, wks=weeks, Wt. Main. = trial was designed for body weight maintenance, Y= yes, yrs=years

Supplementary Table 8. Newcastle Ottawa Scale (NOS) for assessing the quality of prospective cohort studies.

Study	Selection (max 4)				Outcome (max 3)			Comparability (max 2)		Total[b]
	Representativeness of the exposed cohort	Selection of the non-exposed cohort	Ascertainment of exposure	Demonstration that outcome of interest was not present at start of study	Assessment of outcome	Was follow-up long enough for outcomes to occur	Adequacy of follow-up of cohort	Study controls for energy	Study controls for __[a]	
**Overweight/Obesity Incidence**										
Bes-Rastrollo et al. 2007	1	1	0	1	0	1	1	1	1	7
El-Amari et al. 2016[3]	0	1	0	1	0	1	1	NR	NR	4
Freisling et al. 2018 (BMI <25 kg/m^2 at baseline)	1	1	0	1	0	1	1	1	1	7
Freisling et al. 2018 (BMI ≥25 kg/m^2 at baseline)	1	1	0	1	0	1	1	1	1	7
Liu et al. 2019	0	1	0	1	0	1	1	0	0	4
**Body Weight Change**										
Bes-Rastrollo et al. 2007	1	1	0	1	0	1	1	1	1	7
Freisling et al. 2018	1	1	0	1	0	1	1	1	1	7
Smith et al. 2015 (NHS)	0	1	0	1	0	1	1	0	1	5
Smith et al. 2015 (NHS II)	0	1	0	1	0	1	1	0	1	5
Smith et al. 2015 (HPFS)	0	1	0	1	0	1	1	0	1	5
**Weight Gain (≥5 kg) Incidence**										
Bes-Rastrollo et al. 2007	1	1	0	1	0	1	1	1	1	7
El-Amari et al. 2016[c]	0	1	0	1	0	1	1	NR	NR	4
Liu et al. 2019	0	1	0	1	0	1	1	0	1	5
**Waist Circumference Incidence[d]**										
Fernández-Montero et al. 2013 (M)	1	1	0	0	0	1	1	1	1	6
Fernández-Montero et al. 2013 (W)	1	1	0	0	0	1	1	1	1	6

[a]The confounders assessed for this point are: age, sex, physical activity, smoking, baseline BMI/body weight.
[b]A maximum of 9 points may be awarded, with a score of 6 or more being considered higher quality.
[c]Data to determine comparability was not presented in the published abstract. Representativeness of the exposed cohort was assessed using the cohort profile provided by (102).
[d]Waist circumference incidence represents the incidence of increasing ≥ 94 cm for men and ≥80 cm for women.
BMI=body mass index, HPFS=Health Professionals Follow-up Study, M=men, Max= maximum, NHS=Nurses' Health Study, NHS II=Nurses' Health Study II, NR=not reported, W=women.

Supplementary Table 9. Continuous *A priori* subgroup analysis for the effect of nut consumption on measures of adiposity in randomized controlled trials (continued on next page).

a) Body weight (kg)

Subgroups	No. of Comparisons	N	β [95% CI]	P-value	Residual I^2 (%)
Dose (g/d)	105	5479	-0.012 [-0.024, -0.001]	0.038	72.54%
Duration (weeks)	105	5479	-0.009 [-0.032, 0.014]	0.433	76.80%
Age (years)	101	5279	0.014 [-0.006, 0.034]	0.164	75.89%
Baseline body weight (kg)	95	5169	-0.014 [-0.038, 0.010]	0.254	84.29%
Baseline BMI (kg/m^2)	92	5058	-0.023 [-0.073, 0.026]	0.356	44.51%
Baseline body fat (%)	22	964	0.006 [-0.052, 0.064]	0.845	52.52%
Baseline waist circumference (cm)	53	3465	-0.012 [-0.023, 0.000]	0.048	55.82%

b) Body Mass Index (kg/m^2)

Subgroups	No. of Comparisons	N	β [95% CI]	P-value	Residual I^2 (%)
Dose (g/d)	90	4783	-0.005 [-0.011, 0.001]	0.069	46.81%
Duration (weeks)	90	4783	-0.006 [-0.015, 0.003]	0.173	52.01%
Age (years)	86	4639	0.002 [-0.007, 0.012]	0.608	53.32%
Baseline body weight (kg)	76	4332	-0.010 [-0.021, 0.001]	0.070	51.14%
Baseline BMI (kg/m^2)	89	4762	-0.023 [-0.053, 0.007]	0.141	53.21%
Baseline body fat (%)	22	919	-0.001 [-0.019, 0.016]	0.876	0%
Baseline waist circumference (cm)	47	3082	-0.007 [-0.011, -0.002]	0.008	45.67%

c) Body Fat (%)

Subgroups	No. of Comparisons	N	β [95% CI]	P-value	Residual I^2 (%)
Dose (g/d)	43	2345	-0.035 [-0.058, -0.013]	0.002	76.48%
Duration (weeks)	43	2345	0.001 [-0.016, 0.019]	0.874	83.61%
Age (years)	41	2265	-0.003 [-0.034, 0.032]	0.852	85.76%
Baseline body weight (kg)	41	2265	-0.014 [-0.050, 0.022]	0.442	83.96%
Baseline BMI (kg/m^2)	42	2311	-0.014 [-0.119, 0.091]	0.799	84.47%
Baseline body fat (%)	26	1081	0.011 [-0.044, 0.066]	0.707	3.58%
Baseline waist circumference (cm)	28	1608	-0.022 [-0.069, 0.025]	0.351	87.31%

Supplementary Table 9. Continuous *A priori* subgroup analysis for the effect of nut consumption on measures of adiposity in randomized controlled trials.

d) Waist Circumference (cm)

Subgroups	No. of Comparisons	N	β [95% CI]	P-value	Residual I^2 (%)
Dose (g/d)	58	3689	0.020 [-0.008, 0.049]	0.168	98.37%
Duration (weeks)	58	3689	-0.014 [-0.045, 0.017]	0.388	99.46%
Age (years)	54	3489	-0.004 [-0.036, 0.044]	0.884	99.53%
Baseline body weight (kg)	53	3547	-0.014 [-0.024, 0.004]	0.005	24.06%
Baseline BMI (kg/m^2)	54	3415	-0.048 [-0.069, -0.026]	<0.001	11.32%
Baseline body fat (%)	17	805	-0.086 [-0.180, 0.007]	0.070	38.99%
Baseline waist circumference (cm)	54	3563	-0.008 [-0.010, -0.006]	<0.001	1.07%

e) Waist-to-Hip Ratio

Subgroups	No. of Comparisons	N	β [95% CI]	P-value	Residual I^2 (%)
Dose (g/d)	14	1020	0.0002 [-0.001, 0.001]	0.661	79.21%
Duration (weeks)	14	1020	0.0002 [-0.001, 0.001]	0.517	77.97%
Age (years)	14	1020	0.004 [-0.001, 0.002]	0.583	78.62%
Baseline body weight (kg)	11	870	-0.001 [-0.004, 0.002]	0.549	85.23%
Baseline BMI (kg/m^2)	14	1020	-0.006 [-0.015, 0.002]	0.125	75.62%
Baseline body fat (%)	3	113	-0.004 [-0.103, 0.095]	0.937	0.00%
Baseline waist circumference (cm)	7	730	-0.006 [-0.016, 0.003]	0.186	87.01%

Data is presented as the mean difference (95% CI) in each measure of adiposity for every 1-unit change in the predictor variable. ß-coefficients were estimated using continuous meta-regression analysis. Positive ß-coefficients represent an increase and a negative ß-coefficient implies a decrease in adiposity outcome for each unit increase in subgroup variable. Residual I^2 estimates the inter-study heterogeneity not-explained by the subgroup and was estimated using the Cochran Q statistic.
Subgroup analyses could not be adequately explored for visceral adipose tissue owing to too few trial comparisons (<10).
BMI=body mass index, N= number of participants.

Supplementary Table 10. Sensitivity analyses assessing the effect of the systematic removal of an individual study on altering the significance of the pooled effect estimate or the evidence for heterogeneity for the prospective cohort studies pooled analyses.[a]

	**RR [95% CI], P-value I^2, P-value**			**MD [95% CI], P-value I^2, P-value**
	**Overweight/Obesity Incidence N=5**	**Weight Gain (≥5 kg) Incidence N=3**	**Waist Circumference Incidence[b] N=2**	**Body Weight Change (kg) N=5**
Overall	0.95 [0.94, 0.96] P<0.001<br>90%, P<0.001	0.95 [0.94, 0.96] P<0.001<br>47%, P=0.15	0.72 [0.65, 0.80] P<0.001<br>62%, P=0.10	-0.46 [-0.78, -0.13] P=0.01<br>96%, P<0.001
**Removal of:**				
AHS-2 (El-Amari et al. 2016)	0.96 [0.95, 0.98] P<0.001<br>14%, P=0.32	0.95 [0.94, 0.96] P<0.001<br>71%, P=0.06	n/a	n/a
EPIC-PANACEA (Freisling et al. 2018)	n/a	n/a	n/a	-0.56 [-0.90, -0.22] P=0.001<br>92%, P<0.001
EPIC-PANACEA (Freisling et al. 2018 (BMI <25kg/m2 at baseline))	0.95 [0.94, 0.96] P<0.001<br>92%, P<0.001	n/a	n/a	n/a
EPIC-PANACEA (Freisling et al. 2018 (BMI ≥25kg/m2 at baseline))	0.95 [0.94, 0.96] P<0.001<br>92%, P<0.001	n/a	n/a	n/a
NHS/NHS II/HPFS (Liu et al. 2019)	0.92 [0.90, 0.94] P<0.001<br>86%, P<0.001	0.92 [0.87, 0.98] P=0.01<br>65%, P=0.09	n/a	n/a
HPFS (Smith et al. 2015)	n/a	n/a	n/a	-0.43 [-0.89, 0.03] P=0.068<br>97%, P<0.001
NHS (Smith et al. 2015)	n/a	n/a	n/a	-0.50 [-0.98, -0.02] P=0.041<br>98%, P<0.001
NHS II (Smith et al. 2015)	n/a	n/a	n/a	-0.49 [-0.92, -0.05] P=0.028<br>98%, P<0.001
SUN (Bes-Rastrollo et al. 2007)	0.95 [0.94, 0.96] P<0.001<br>92%, P<0.001	0.95 [0.94, 0.96] P<0.001<br>0%, P=0.56	n/a	-0.49 [-0.92, -0.05] P=0.028<br>98%, P<0.001
SUN (Fernández-Montero et al. 2013 - Men)	n/a	n/a	0.69 [0.61, 0.78] P<0.001<br>n/a	n/a
SUN (Fernández-Montero et al. 2013 - Women)	n/a	n/a	0.86 [0.68, 1.09] P=0.21<br>n/a	n/a

[a]Sensitivity analysis included the removal of each single study from the meta-analyses one at a time and the summary effect was recalculated. An influential outlier was considered a study whose removal changed the magnitude of the pooled effect by >10%.
[b]Waist circumference incidence represents the incidence of ≥ 94 cm for men and ≥80 cm for women.
BMI=body mass index, CI = confidence interval, HPFS=Health Professionals Follow-up Study, MD = mean difference, n/a = not applicable, NHS=Nurses' Health Study, RR=relative risk.

Supplementary Table 11. Sensitivity analysis of the systematic removal of each trial.[a]

	MD [95% CI], P-value<br>I^2, P-value					
	**Body Weight (kg)**<br>**N=106**	**BMI (kg/m²)**<br>**N=89**	**Body Fat (%)**<br>**N=43**	**Waist Circumference (cm)**<br>**N=59**	**Waist-to-Hip Ratio**<br>**N=14**	**Visceral Adipose Tissue[b]**<br>**N=9**
Overall	0.09 [-0.09, 0.27] P=0.340<br>63.2%, P<0.01	-0.04 [-0.12, 0.05] P=0.411<br>31.9%, P<0.01	-0.05 [-0.42, 0.31] P=0.766<br>77.0%, P<0.01	0.03 [-0.09, 0.15] P=0.637<br>68.6%, P<0.01	-0.01 [-0.04, 0.01] P=0.312<br>84.0%, P<0.01	-0.59 [-1.32, 0.14] P=0.114<br>64.7%, P=0.004
**Removal of:**						
Abazarfard et al. 2014	0.12 [-0.06, 0.31] P=0.179<br>62.0%, P<0.01	-0.01 [-0.09, 0.07] P=0.81<br>24.2%, P<0.01	n/a	0.05 [-0.04, 0.15] P=0.282<br>57.5%, P<0.01	0.00 [-0.01, 0.01] P=0.511<br>0.0%, P=0.979	n/a
Abbaspour et al. 2019	0.09 [-0.09, 0.27] P=0.334<br>63.6%, P<0.01	-0.04 [-0.12, 0.05] P=0.427<br>32.4%, P<0.01	-0.06 [-0.42, 0.31] P=0.767<br>77.6%, P<0.01	0.03 [-0.09, 0.15] P=0.603<br>69.1%, P<0.01	-0.01 [-0.04, 0.01] P=0.373<br>85.3%, P<0.01	n/a
Agebratt et al. 2016	0.09 [-0.09, 0.27] P=0.341<br>63.6%, P<0.01	-0.04 [-0.12, 0.05] P=0.403<br>32.7%, P<0.01	n/a	n/a	n/a	n/a
Baer et al. 2019	0.09 [-0.10, 0.29] P=0.360<br>63.1%, P<0.01	n/a	n/a	n/a	n/a	n/a
Balci et al. 2012	0.09 [-0.10, 0.27] P=0.344<br>63.6%, P<0.01	n/a	n/a	0.03 [-0.09, 0.15] P=0.641<br>69.2% P<0.01	n/a	n/a
Bamberger et al. 2017	0.09 [-0.10, 0.27] P=0.344<br>63.6%, P<0.01	-0.04 [-0.12, 0.05] P=0.399<br>32.7%, P<0.01	n/a	0.03 [-0.09, 0.15] P=0.633<br>69.2%, P<0.01	n/a	n/a
Barbour et al. 2015	0.07 [-0.11, 0.26] P=0.431<br>62.0%, P<0.01	-0.04 [-0.12, 0.05] P=0.382<br>32.6%, P<0.01	-007 [-0.46, 0.31] P=0.707<br>77.0%, P<0.01	0.03 [-0.09, 0.15] P=0.646<br>69.2%, P<0.01	n/a	n/a
Bento et al. 2014	0.09 [-0.09, 0.27] P=0.339<br>63.6%, P<0.01	-0.04 [-0.12, 0.05] P=0.404<br>32.7%, P<0.01	-0.05 [-0.41, 0.31] P=0.789<br>77.6%, P<0.01	n/a	n/a	n/a
Berryman et al. 2015	0.09 [-0.09, 0.27] P=0.335<br>63.6%, P<0.01	n/a	-0.05 [-0.44, 0.34] P=0.816<br>77.6%, P<0.01	0.03 [-0.09, 0.15] P=0.615<br>69.1%, P<0.01	n/a	n/a
Bitok et al. 2018	0.09 [-0.10, 0.27] P=0.343<br>63.6%, P<0.01	-0.04 [-0.12, 0.05] P=0.412<br>32.8%, P<0.01	-0.08 [-0.46, 0.30] P=0.680<br>76.4%, P<0.01	0.03 [-0.09, 0.15] P=0.646<br>69.2%, P<0.01	-0.02 [-0.04, 0.01] P=0.263<br>83.4%, P<0.01	n/a
Biude Silva Duarte et al. 2019	0.11 [-0.08, 0.29] P=0.267<br>63.4%, P<0.01	-0.04 [-0.13, 0.05] P=0.394<br>32.7%, P<0.01	-0.07 [-0.44, 0.30] P=0.712<br>77.5%, P<0.01	0.04 [-0.07, 0.15] P=0.488<br>67.2%, P<0.01	n/a	n/a
Bowen et al. 2019	0.09 [-0.10, 0.27] P=0.350<br>63.5%, P<0.01	n/a	-0.02 [-0.39, 0.35] P=0.922<br>77.3%, P<0.01	0.03 [-0.09, 0.15] P=0.581<br>69.1%, P<0.01	n/a	-0.59 [-1.34, 0.16] P=0.122<br>68.8%, P=0.002
Campbell et al. 2019	0.09 [0.09, 0.27] P=0.332<br>63.5%, P<0.01	n/a	n/a	0.03 [-0.09, 0.15] P=0.628<br>68.9%, P<0.01	n/a	-0.69 [-1.55, 0.17] P=0.114<br>68.9%, P=0.002
Canales et al. 2007	0.09 [-0.09, 0.27] P=0.341<br>63.6%, P<0.01	-0.04 [-0.12, 0.05] P=0.413<br>32.7%, P<0.01	n/a	n/a	n/a	n/a
Carughi et al. 2019	0.09 [-0.10, 0.27] P=0.344<br>63.6%, P<0.01	n/a	-0.05 [-0.41, 0.32] P=0.798<br>77.6%, P<0.01	0.03 [-0.09, 0.15] P=0.632<br>69.2%, P<0.01	-0.01 [-0.04, 0.01] P=0.315<br>85.3%, P<0.01	n/a
Casas-Agustench et al. 2011	0.10 [-0.09, 0.28] P=0.295<br>63.5%, P<0.01	-0.03 [-0.12, 0.05] P=0.457<br>32.3%, P<0.01	-0.03 [-0.40, 0.34] P=0.858<br>77.5%, P<0.01	0.03 [-0.09, 0.15] P=0.630<br>69.2%, P<0.01	n/a	n/a

Reference Removed	MD [95% CI], P-value<br>I^2, P-value					
	**Body Weight (kg)**<br>**N=105**	**BMI (kg/m²)**<br>**N=90**	**Body Fat (%)**<br>**N=43**	**Waist Circumference (cm)**<br>**N=58**	**Waist-to-Hip Ratio**<br>**N=14**	**Visceral Adipose Tissue[b]**<br>**N=9**
Chisholm et al. 2005	0.09 [-0.10, 0.28] P=0.337<br>63.5%, P<0.01	-0.04 [-0.12, 0.05] P=0.404<br>32.7%, P<0.01	n/a	n/a	-0.01 [-0.04, 0.01] P=0.318<br>84.6%, P<0.01	n/a
Ciccone et al. 2014 [Control]	0.09 [-0.09, 0.27] P=0.337<br>63.6%, P<0.01	-0.04 [-0.12, 0.05] P=0.410<br>32.6%, P<0.01	n/a	0.03 [-0.09, 0.15] P=0.637<br>69.1%, P<0.01	n/a	n/a
Ciccone et al. 2014 [Non-fried fish]	0.09 [-0.09, 0.27] P=0.340<br>63.6%, P<0.01	-0.04 [-0.12, 0.05] P=0.416<br>32.4%, P<0.01	n/a	0.03 [-0.09, 0.15] P=0.640<br>69.2%, P<0.01	n/a	n/a
Ciccone et al. 2014 [Olive Oil]	0.09 [-0.09, 0.27] P=0.336<br>63.5%, P<0.01	-0.04 [-0.12, 0.05] P=0.408<br>32.7%, P<0.01	n/a	0.03 [-0.09, 0.15] P=0.637<br>69.1%, P<0.01	n/a	n/a
Cohen et al. 2011	0.09 [-0.09, 0.27] P=0.340<br>63.6%, P<0.01	-0.04 [-0.12, 0.50] P=0.408<br>32.6%, P<0.01	-0.05 [-0.42, 0.31] P=0.767<br>77.6%, P<0.01	n/a	n/a	n/a
Damasceno et al. 2011 [Almond]	0.09 [-0.09, 0.27] P=0.341<br>63.6%, P<0.01	-0.04 [-0.12, 0.05] P=0.403<br>32.7%, P<0.01	n/a	n/a	n/a	n/a
Damasceno et al. 2011 [Walnut]	0.09 [-0.09, 0.27] P=0.341<br>63.6%, P<0.01	-0.04 [-0.12, 0.05] P=0.403<br>32.7%, P<0.01	n/a	n/a	n/a	n/a
Damavandi et al. 2012	0.09 [-0.09, 0.27] P=337<br>63.6%, P<0.01	-0.04 [-0.12, 0.05] P=0.406<br>32.7%, P<0.01	n/a	0.03 [-0.09, 0.15] P=0.637<br>69.2%, P<0.01	n/a	n/a
Damavandi et al. 2013	0.09 [-0.09, 0.27] P=0.339<br>63.6%, P<0.01	-0.04 [-0.12, 0.50] P=0.416<br>32.6%, P<0.01	n/a	n/a	n/a	n/a
de Souza et al. 2018	0.09 [-0.09, 0.27] P=0.340<br>63.6%, P<0.01	-0.04 [-0.12, 0.05] P=0.406<br>32.7%, P<0.01	-0.06 [-0.42, 0.31] P=0.765<br>77.65, P<0.01	0.03 [-0.09, 0.15] P=0.629<br>69.1%, P<0.01	n/a	n/a
Dhillon et al. 2016	0.11 [-0.09, 0.29] P=0.259<br>63.3%, P<0.01	n/a	n/a	0.03 [-0.09, 0.14] P=0.627<br>69.1%, P<0.01	n/a	-0.13 [-0.43, 0.16] P=0.382<br>0.0%, P=0.473
Dhillon et al. 2018	0.12 [-0.07, 0.30] P=0.228<br>62.2%, P<0.01	n/a	-0.06 [-0.42, 0.31] P=0.761<br>77.6%, P<0.01	-0.02 [-0.14, 0.10] P=0.725<br>52.3%, P<0.01	n/a	n/a
Foster et al. 2012	0.08 [-0.10, 0.26] P=0.386<br>63.2%, P<0.01	n/a	-0.05 [-0.44, 0.33] P=0.783<br>77.6%, P<0.01	n/a	n/a	n/a
Gebauer et al. 2008 [High Dose Pistachio]	0.09 [-0.09, 0.27] P=0.340<br>63.6%, P<0.01	-0.04 [-0.12, 0.05] P=0.408<br>32.7%, P<0.01	n/a	n/a	n/a	n/a
Gebauer et al. 2008 [Low Dose Pistachio]	0.09 [-0.09, 0.27] P=341<br>63.6%, P<0.01	-0.04 [-0.12, 0.05] P=0.408<br>32.7%, P<0.01	n/a	n/a	n/a	n/a
Gulati et al. 2014	0.09 [-0.09, 0.27] P=0.333<br>63.6%, P<0.01	n/a	n/a	0.03 [-0.09, 0.14] P=0.662<br>69.0%, P<0.01	n/a	-0.60 [-1.35, 0.14] P=0.113<br>68.9%, P=0.002
Hernández-Alonso et al. 2014	0.09 [-0.10, 0.27] P=0.369<br>63.3%, P<0.01	-0.05 [-0.14, 0.04] P=0.292<br>31.0%, P<0.01	n/a	0.01 [-0.11, 0.12] P=0.891<br>68.0%, P<0.01	n/a	n/a

Reference Removed	MD [95% CI], P-value<br>I^2, P-value					
	Body Weight (kg)<br>N=105	BMI (kg/m^2)<br>N=90	Body Fat (%)<br>N=43	Waist Circumference (cm)<br>N=58	Waist-to-Hip Ratio<br>N=14	Visceral Adipose Tissue[b]<br>N=9
Hiraoka-Yamamoto et al. 2004 [Butter]	0.09 [-0.09, 0.27] P=0.336<br>63.6%, P<0.01	-0.04 [-0.12, 0.05] P=0.418<br>32.6%, P<0.01	n/a	n/a	n/a	n/a
Hiraoka-Yamamoto et al. 2004 [Coconut]	0.09 [-0.09, 0.27] P=0.337<br>63.6%, P<0.01	-0.04 [-0.12, 0.05] P=0.410<br>32.6%, P<0.01	n/a	n/a	n/a	n/a
Hollis and Mattes. 2007	0.09 [-0.10, 0.27] P=0.348<br>63.6%, P<0.01	n/a	-0.07 [-0.43, 0.30] P=0.710<br>77.5%, P<0.01	n/a	n/a	n/a
Hudthagosol et al. 2012 [Control]	0.08 [-0.10, 0.26] P=0.387<br>63.3%, P<0.01	-0.04 [-0.13, 0.05] P=0.385<br>32.7%, P<0.01	n/a	n/a	n/a	n/a
Hudthagosol et al. 2012 [Salmon]	0.09 [-0.10, 0.27] P=0.348<br>63.6%, P<0.01	-0.04 [-0.13, 0.05] P=0.382<br>32.7%, P<0.01	n/a	n/a	n/a	n/a
Hwang et al. 2019	n/a	n/a	n/a	0.02 [-0.09, 0.14] P=0.705<br>68.9%, P<0.01	n/a	n/a
Jamshed et al. 2015 [American Almonds]	0.08 [-0.11, 0.26] P=0.405<br>63.2%, P<0.01	n/a	n/a	n/a	n/a	n/a
Jamshed et al. 2015 [Pakistani Almonds]	0.04 [-0.13, 0.20] P=0.661<br>55.3%, P<0.01	n/a	n/a	n/a	n/a	n/a
Jenkins et al. 2002 [Full Dose Almonds]	0.09 [-0.09, 0.27] P=0.339<br>63.6%, P<0.01	-0.04 [-0.12, 0.05] P=0.411<br>32.7%, P<0.01	-0.05 [-0.41, 0.32] P=0.795<br>77.6%, P<0.01	0.03 [-0.09, 0.15] P=0.635<br>69.1%, P<0.01	-0.01 [-0.04, 0.01] P=0.337<br>85.2%, P<0.01	n/a
Jenkins et al. 2002 [Half Dose Almonds]	0.09 [-0.09, 0.27] P=0.339<br>63.6%, P<0.01	-0.04 [-0.12, 0.05] P=0.419<br>32.6%, P<0.01	-0.05 [-0.42, 0.31] P=0.769<br>77.6%, P<0.01	0.03 [-0.09, 0.15] P=0.632<br>69.1%, P<0.01	-0.01 [-0.04, 0.01] P=0.303<br>85.1%, P<0.01	n/a
Jenkins et al. 2018 [Full Dose Nuts]	0.10 [-0.09, 0.29] P=0.295<br>63.6%, P<0.01	-0.03 [-0.12, 0.05] P=0.453<br>32.2%, P<0.01	n/a	0.01 [-0.19, 0.18] P=0.933<br>68.0%, P<0.01	-0.01 [-0.04, 0.02] P=0.380<br>85.0%, P<0.01	n/a
Jenkins et al. 2018 [Half Dose Nuts]	0.08 [-0.10, 0.27] P=0.388<br>63.3%, P<0.01	-0.04 [-0.13, 0.04] P=0.331<br>32.3%, P<0.01	n/a	0.01 [-0.19, 0.18] P=0.933<br>68.0%, P<0.01	-0.01 [-0.04, 0.02] P=0.380<br>85.0%, P<0.01	n/a
Johnston et al. 2013	0.07 [-0.11, 0.25] P=0.455<br>62.7%, P<0.01	n/a	-0.07 [-0.44, 0.30] P=0.713<br>77.5%, P<0.01	0.03 [-0.08, 0.15] P=0.581<br>68.6%, P<0.01	n/a	n/a
Jung et al. 2018	0.09 [-0.09, 0.28] P=0.331<br>63.6%, P<0.01	-0.04 [-0.12, 0.05] P=0.429<br>32.5%, P<0.01	-0.04 [-0.46, 0.37] P=0.845<br>77.5%, P<0.01	0.03 [-0.09, 0.15] P=8.650<br>69.1%, P<0.01	n/a	n/a
Katz et al. 2012	0.06 [-0.12, 0.24] P=0.504<br>61.6%, P<0.01	-0.05 [-0.13, 0.03] P=0.240<br>27.2%, P=0.01	n/a	0.03 [-0.09, 0.15] P=0.622<br>69.2%, P<0.01	n/a	n/a
Kocyigit et al. 2006	n/a	-0.04 [-0.12, 0.05] P=0.404<br>32.7%, P<0.01	n/a	n/a	n/a	n/a
Le et al. 2016 [Lower CHO]	0.09 [-0.09, 0.27] P=0.332<br>63.6%, P<0.01	-0.04 [-0.12, 0.05] P=0.424<br>32.5%, P<0.01	n/a	n/a	n/a	n/a
Le et al. 2016 [Lower Fat]	0.09 [-0.10, 0.27] P=347<br>63.6%, P<0.01	-0.04 [-0.12, 0.05] P=0.395<br>32.7%, P<0.01	n/a	n/a	n/a	n/a

Reference Removed	MD [95% CI], P-value I², P-value					
	Body Weight (kg) N=105	BMI (kg/m²) N=90	Body Fat (%) N=43	Waist Circumference (cm) N=58	Waist-to-Hip Ratio N=14	Visceral Adipose Tissue[b] N=9
Lee et al. 2014	0.09 [-0.10, 0.28] P=0.338 63.5%, P<0.01	-0.04 [-0.13, 0.05] P=0.386 32.7%, P<0.01	n/a	0.05 [-0.07, 0.17] P=0.429 68.5%, P<0.01	n/a	n/a
Li et al. 2010	0.09 [-0.09, 0.27] P=0.335 63.6%, P<0.01	-0.03 [-0.12, 0.05] P=0.455 32.0%, P<0.01	n/a	n/a	n/a	n/a
Li et al. 2011	n/a	-0.04 [-0.12, 0.05] P=0.400 32.7%, P<0.01	-0.05 [-0.41, 0.31] P=0.789 77.6%, P<0.01	n/a	n/a	n/a
Liu et al. 2018	0.10 [-0.09, 0.29] P=0.288 63.6%, P<0.01	-0.04 [-0.12, 0.05] P=0.437 32.5%, P<0.01	-0.03 [-0.40, 0.34] P=0.882 77.5%, P<0.01	0.03 [-0.09, 0.15] P=0.595 69.0%, P<0.01	n/a	-0.89 [-1.89, 0.12] P=0.086 68.9%, P=0.002
Ma et al. 2010	0.10 [-0.09, 0.28] P=0.291 63.5%, P<0.01	-0.03 [-0.12, 0.06] P=0.484 31.9%, P<0.01	n/a	0.03 [-0.09, 0.15] P=0.630 69.2%, P<0.01	n/a	n/a
McKay et al. 2018	0.09 [-0.09, 0.27] P=0.339 63.6%, P<0.01	-0.04 [-0.12, 0.05] P=0.409 32.7%, P<0.01	n/a	n/a	n/a	n/a
Mohan et al. 2018	0.08 [-0.11, 0.27] P=0.400 62.4%, P<0.01	-0.05 [-0.14, 0.04] P=0.306 31.3%, P<0.01	n/a	0.03 [-0.09, 0.15] P=0.585 69.1%, P<0.01	n/a	n/a
Moreira Alves et al. 2014 [Conventional Peanut]	0.08 [-0.11, 0.26] P=0.408 63.2%, P<0.01	-0.04 [-0.13, 0.04] P=0.318 32.0%, P<0.01	-0.03 [-0.40, 0.34] P=0.864 77.5%, P<0.01	0.03 [-0.09, 0.15] P=0.647 69.2%, P<0.01	-0.01 [-0.04, 0.01] P=0.315 85.3%, P<0.01	n/a
Moreira Alves et al. 2014 [High Oleic Peanut]	0.08 [-0.10, 0.27] P=0.384 63.4%, P<0.01	-0.04 [-0.13, 0.05] P=0.344 32.4%, P<0.01	-0.02 [-0.39, 0.35] P=0.925 77.3%, P<0.01	0.02 [-0.10, 0.14] P=0.704 69.1%, P<0.01	-0.01 [-0.04, 0.01] P=0.315 85.3%, P<0.01	n/a
Morgan et al. 2000	0.09 [-0.09, 0.27] P=0.341 63.6%, P<0.01	-0.04 [-0.12, 0.05] P=0.406 32.7%, P<0.01	n/a	n/a	n/a	n/a
Morgan et al. 2002	n/a	-0.04 [-0.12, 0.05] P=0.405 32.7%, P<0.01	n/a	n/a	n/a	n/a
Nagashree et al. 2017	0.09 [-0.09, 0.27] P=0.342 63.6%, P<0.01	-0.04 [-0.12, 0.05] P=0.404 32.7%, P<0.01	n/a	n/a	-0.02 [-0.04, 0.01] P=0.236 84.7%, P<0.01	n/a
Njike et al. 2015 [Ad libitum]	n/a	-0.05 [-0.13, 0.04] P=0.287 30.5%, P<0.01	-0.08 [-0.45, 0.29] P=0.665 77.4%, P<0.01	0.02 [-0.09, 0.14] P=0.698 69.0%, P<0.01	n/a	-1.06 [-2.12, 0.01] P=0.053 67.6%, P=0.003
Njike et al. 2015 [Calorie adjusted]	n/a	-0.04 [-0.13, 0.05] P=0.369 32.6%, P<0.01	-0.05 [-0.42, 0.32] P=0.794 77.6%, P<0.01	0.02 [-0.09, 0.14] P=0.698 69.0%, P<0.01	n/a	-1.03 [-2.09, 0.03] P=0.058 68.5%, P<0.002
Njike et al. 2017	0.09 [-0.09, 0.28] P=0.323 63.6%, P<0.01	-0.04 [-0.12, 0.05] P=0.429 32.5%, P<0.01	-0.03 [-0.39, 0.32] P=0.850 77.0%, P<0.01	0.03 [-0.09, 0.15] P=0.650 69.2%, P<0.01	n/a	-0.39 [-1.06, 0.28] P=0.251 60.5%, P=0.013
Parham et al. 2014	n/a	-0.02 [-0.10, 0.07] P=0.658 28.5%, P<0.01	n/a	n/a	n/a	n/a
Rajaram et al. 2001	0.12 [-0.06, 0.29] P=0.199 45.6%, P<0.01	n/a	n/a	n/a	n/a	n/a
Robbins et al. 2012	0.09 [-0.10, 0.27] P=0.367 63.4%, P<0.01	0.02 [-0.04, 0.09] P=0.501 10.9%, P=0.202	n/a	n/a	n/a	n/a

Reference Removed	MD [95% CI], P-value<br>I^2, P-value					
	**Body Weight (kg)**<br>**N=105**	**BMI (kg/m²)**<br>**N=90**	**Body Fat (%)**<br>**N=43**	**Waist Circumference (cm)**<br>**N=58**	**Waist-to-Hip Ratio**<br>**N=14**	**Visceral Adipose Tissue[b]**<br>**N=9**
Rock et al. 2017	0.09 [-0.10, 0.27] P=0.360<br>63.5%, P<0.01	-0.04 [-0.12, 0.05] P=0.397<br>32.7%, P<0.01	n/a	0.03 [-0.09, 0.14] P=0.672<br>68.9%, P<0.01	n/a	n/a
Ros et al. 2004	0.09 [-0.09, 0.27] P=0.342<br>63.6%, P<0.01	n/a	n/a	n/a	n/a	n/a
Ruisinger et al. 2015	0.09 [-0.09, 0.27] P=0.342<br>63.6%, P<0.01	-0.04 [-0.12, 0.05] P=0.397<br>32.7%, P<0.01	n/a	n/a	n/a	n/a
Sabate et al. 2003<br>[High Dose Almond]	0.09 [-0.09, 0.27] P=0.340<br>63.6%, P<0.01	n/a	n/a	n/a	n/a	n/a
Sabate et al. 2003<br>[Low Dose Almond]	0.09 [-0.09, 0.27] P=0.342<br>63.6%, P<0.01	n/a	n/a	n/a	n/a	n/a
Sabaté et al. 2005	0.08 [-0.11, 0.26] P=0.413<br>58.0%, P<0.01	-0.05 [-0.14, 0.04] P=0.290<br>30.9%, P<0.01	-0.06 [-0.46, 0.34] P=0.767<br>77.3%, P<0.01	n/a	n/a	n/a
Sauder et al. 2015	0.09 [-0.09, 0.27] P=0.340<br>63.6%, P<0.01	-0.04 [-0.12, 0.05] P=0.405<br>32.7%, P<0.01	n/a	n/a	n/a	n/a
Schutte et al. 2006<br>[Cashew]	0.09 [-0.10, 0.27] P0.369<br>63.4%, P<0.01	-0.04 [-0.12, 0.05] P=0.408<br>32.7%, P<0.01	n/a	0.03 [-0.09, 0.15] P=0.643<br>69.2%, P<0.01	n/a	n/a
Schutte et al. 2006<br>[Walnut]	0.08 [-0.10, 0.27] P=0.371<br>63.4%, P<0.01	-0.04 [-0.12, 0.05] P=0.405<br>32.7%, P<0.01	n/a	0.03 [-0.09, 0.15] P=0.640<br>69.2%, P<0.01	n/a	n/a
Sheridan et al. 2007	0.09 [-0.09, 0.27] P=0.341<br>63.6%, P<0.01	-0.04 [-0.12, 0.05] P=0.401<br>32.7%, P<0.01	n/a	n/a	n/a	n/a
Somerset et al. 2013	0.09 [-0.09, 0.27] P=0.330<br>63.5%, P<0.01	-0.04 [-0.12, 0.05] P=0.416<br>32.5%, P<0.01	-0.23 [-0.54, 0.09] P=0.162<br>68.0%, P<0.01	0.03 [-0.09, 0.15] P= 0.606<br>68.5%, P<0.01	n/a	n/a
Spaccarotella et al. 2008	0.09 [-0.10, 0.27] P=0.346<br>63.6%, P<0.01	-0.04 [-0.12, 0.05] P=0.405<br>32.7%, P<0.01	n/a	n/a	n/a	n/a
Spiller et al. 1998<br>[Cheddar<br>Cheese/Butter]	0.09 [-0.09, 0.27] P=0.343<br>63.6%, P<0.01	n/a	n/a	n/a	n/a	n/a
Spiller et al. 1998<br>[Olive oil]	0.09 [-0.10, 0.27] P=0.344<br>63.6%, P<0.01	n/a	n/a	n/a	n/a	n/a
Sweazea et al. 2014	0.08 [-0.10, 0.27] P=0.369<br>63.4%, P<0.01	-0.04 [-0.13, 0.05] P=0.367<br>32.3%, P<0.01	-0.08 [-0.44, 0.28] P=0.672<br>77.3%, P<0.01	0.03 [-0.09, 0.15] P=0.650<br>69.1%, P<0.01	n/a	n/a
Tan and Mattes. 2013<br>[Afternoon Snack<br>Almonds]	0.09 [-0.09, 0.27] P=0.341<br>63.6%, P<0.01	-0.04 [-0.12, 0.05] P=0.404<br>32.7%, P<0.01	-0.05 [-0.42, 0.31] P=0.775<br>77.6%, P<0.01	0.03 [-0.09, 0.15] P=0.640<br>69.2%, P<0.01	n/a	n/a
Tan and Mattes. 2013<br>[Breakfast Almonds]	0.09 [-0.09, 0.27] P=0.341<br>63.6%, P<0.01	-0.04 [-0.12, 0.05] P=0.404<br>32.7%, P<0.01	-0.05 [-0.42, 0.31] P=0.769<br>77.6%, P<0.01	0.03 [-0.09, 0.15] P=0.640<br>69.2%, P<0.01	n/a	n/a
Tan and Mattes. 2013<br>[Lunch Almonds]	0.09 [-0.09, 0.27] P=0.341<br>63.6%, P<0.01	-0.04 [-0.12, 0.05] P=404<br>32.7%, P<0.01	-0.05 [-0.42, 0.31] P=0.777<br>77.6%, P<0.01	0.03 [-0.09, 0.15] P=0.640<br>69.2%, P<0.01	n/a	n/a

Reference Removed	MD [95% CI], P-value<br>I², P-value					
	**Body Weight (kg)**<br>**N=105**	**BMI (kg/m²)**<br>**N=90**	**Body Fat (%)**<br>**N=43**	**Waist Circumference (cm)**<br>**N=58**	**Waist-to-Hip Ratio**<br>**N=14**	**Visceral Adipose Tissue[b]**<br>**N=9**
Tan and Mattes. 2013 [Morning Snack Almonds]	0.09 [-0.09, 0.27] P=0.341<br>63.6%, P<0.01	-0.04 [-0.12, 0.05] P=0.405<br>32.7%, P<0.01	-0.06 [-0.42, 0.31] P=0.763<br>77.6%, P<0.01	0.03 [-0.09, 0.15] P=0.640<br>69.2%, P<0.01	n/a	n/a
Tapsell et al. 2004 [Low Fat]	0.09 [-0.09, 0.27] P=0.338<br>63.6%, P<0.01	-0.04 [-0.12, 0.05] P=0.414<br>32.6%, P<0.01	-0.05 [-0.41, 0.31] P=0.783<br>77.6%, P<0.01	n/a	n/a	n/a
Tapsell et al. 2004 [Modified Fat]	0.09 [-0.09, 0.27] P=0.339<br>63.6%, P<0.01	-0.04 [-0.12, 0.05] P=0.409<br>32.7%, P<0.01	-0.05 [-0.42, 0.31] P=0.775<br>77.6%, P<0.01	n/a	n/a	n/a
Tapsell et al. 2009	0.09 [-0.09, 0.27] P=0.340<br>63.6%, P<0.01	n/a	-0.05 [-0.42, 0.31] P=0.778<br>77.6%, P<0.01	n/a	n/a	-0.61 [-1.35, 0.14] P=0.110<br>68.8%, P=0.002
Tapsell et al. 2017 [Control]	0.09 [-0.09, 0.27] P=0.335<br>63.6%, P<0.01	-0.04 [-0.12, 0.05] P=0.424<br>32.3%, P<0.01	-0.05 [-0.41, 0.31] P=0.782<br>77.6%, P<0.01	n/a	n/a	n/a
Tapsell et al. 2017 [Intervention Alone]	0.09 [-0.09, 0.27] P=0.340<br>63.6%, P<0.01	-0.04 [-0.12, 0.05] P=0.416<br>32.6%, P<0.01	-0.05 [-0.42, 0.31] P=0.772<br>77.6%, P<0.01	n/a	n/a	n/a
Tey et al. 2011 [Chocolate]	0.09 [-0.09, 0.27] P=0.359<br>63.6%, P<0.01	-0.04 [-0.13, 0.05] P=0.372<br>32.7%, P<0.01	-0.07 [-0.44, 0.30] P=0.698<br>77.5%, P<0.01	0.03 [-0.09, 0.14] P=0.653<br>69.1%, P<0.01	n/a	n/a
Tey et al. 2011 [Control]	0.10 [-0.10, 0.26] P=0.377<br>63.6%, P<0.01	-0.04 [-0.13, 0.05] P=0.340<br>32.4%, P<0.01	-0.06 [-0.43, 0.31] P=0.752<br>77.6%, P<0.01	0.03 [-0.09, 0.14] P=0.651<br>69.1%, P<0.01	n/a	n/a
Tey et al. 2011 [Potato crisp]	0.10 [-0.10, 0.26] P=0.372<br>63.6%, P<0.01	-0.04 [-0.13, 0.05] P=0.347<br>32.5%, P<0.01	-0.07 [-0.44, 0.30] P=0.716<br>77.5%, P<0.01	0.03 [-0.09, 0.14] P=0.672<br>68.7%, P<0.01	n/a	n/a
Tey et al. 2013 [High Dose Hazelnuts]	0.09 [-0.09, 0.27] P=0.341<br>63.6%, P<0.01	-0.04 [-0.12, 0.05] P=0.405<br>32.7%, P<0.01	-0.05 [-0.42, 0.31] P=0.769<br>77.6%, P<0.01	n/a	n/a	n/a
Tey et al. 2013 [Low Dose Hazelnuts]	0.09 [-0.09, 0.27] P=0.341<br>63.6%, P<0.01	-0.04 [-0.12, 0.05] P=0.405<br>32.7%, P<0.01	-0.06 [-0.42, 0.31] P=0.767<br>77.6%, P<0.01	n/a	n/a	n/a
Tindall et al. 2019 [ALA]	0.08 [-0.08, 0.28] P=0.272<br>63.6%, P<0.11	n/a	n/a	n/a	n/a	n/a
Tindall et al. 2019 [Oleic Acid]	0.09 [-0.09, 0.27] P=0.338<br>63.6%, P<0.01	n/a	n/a	n/a	n/a	n/a
Tsaban et al. 2017	0.08 [-0.08, 0.28] P=0.291<br>63.6%, P<0.01	-0.03 [-0.11, 0.06] P=0.497<br>31.2%, P<0.01	n/a	0.04 [-0.08, 0.15] P=0.521<br>67.4%, P<0.01	n/a	n/a
Vergani et al. 2018 [Control]	n/a	-0.04 [-0.12, 0.05] P=0.406<br>32.7%, P<0.01	n/a	n/a	n/a	n/a
Vergani et al. 2018 [Fruits & VegTables]	n/a	-0.04 [-0.12, 0.05] P=0.405<br>32.7%, P<0.01	n/a	n/a	n/a	n/a
Wang et al. 2012 [High Dose Pistachio]	0.09 [-0.09, 0.27] P=0.341<br>63.6%, P<0.01	-0.04 [-0.12, 0.05] P=0.406<br>32.7%, P<0.01	n/a	n/a	-0.01 [-0.04, 0.01] P=0.302<br>85.2%, P<0.01	n/a
Wang et al. 2012 [Recommended Dose Pistachio]	0.09 [-0.09, 0.27] P=0.341<br>63.6%, P<0.01	-0.04 [-0.12, 0.05] P=0.406<br>32.7%, P<0.01	n/a	n/a	-0.01 [-0.04, 0.01] P=0.317<br>85.2%, P<0.01	n/a

Reference Removed	MD [95% CI], P-value<br>I^2, P-value					
	**Body Weight (kg)**<br>**N=105**	**BMI (kg/m²)**<br>**N=90**	**Body Fat (%)**<br>**N=43**	**Waist Circumference (cm)**<br>**N=58**	**Waist-to-Hip Ratio**<br>**N=14**	**Visceral Adipose Tissue[b]**<br>**N=9**
Wien et al. 2003	0.05 [-0.05, 0.29] P=0.173<br>59.5%, P<0.11	0.01 [-0.05, 0.08] P=0.689<br>7.7%, P=0.278	0.08 [-0.19, 0.34] P=0.558<br>51.7%, P<0.01	0.04 [-0.08, 0.15] P=0.537<br>67.5%, P<0.01	n/a	n/a
Wien et al. 2010	0.09 [-0.09, 0.27] P=0.333<br>63.6%, P<0.01	-0.04 [-0.12, 0.05] P=0.422<br>32.5%, P<0.01	n/a	0.03 [-0.09, 0.15] P=0.642<br>69.2%, P<0.01	n/a	n/a
Wien et al. 2014	0.09 [-0.09, 0.27] P=0.341<br>63.6%, P<0.01	-0.04 [-0.12, 0.05] P=0.406<br>32.7%, P<0.01	n/a	0.03 [-0.09, 0.15] P=0.640<br>69.2%, P<0.01	n/a	n/a
Williams et al. 2019 [High CHO]	0.09 [-0.09, 0.27] P=0.324<br>63.6%, P<0.01	-0.03 [-0.12, 0.05] P=0.446<br>32.05, P<0.01	n/a	0.03 [-0.09, 0.14] P=0.685<br>69.1%, P<0.01	n/a	n/a
Williams et al. 2019 [Low CHO]	0.10 [-0.10, 0.26] P=0.400<br>63.6%, P<0.01	-0.04 [-0.13, 0.05] P=0.330<br>32.3%, P<0.01	n/a	0.02 [-0.10, 0.14] P=0.717<br>69.0%, P<0.01	n/a	n/a
Wilson et al. 2014	0.10 [-0.10, 0.26] P=0.407<br>63.6%, P<0.01	-0.04 [-0.12, 0.05] P=0.403<br>32.7%, P<0.01	n/a	n/a	n/a	n/a
Wu et al. 2010 [Flaxseed & Lifestyle Counseling]	0.10 [-0.10, 0.27] P=0.392<br>62.6%, P<0.01	-0.04 [-0.12, 0.05] P=0.394<br>32.7%, P<0.01	n/a	0.03 [-0.09, 0.14] P=0.661<br>69.2%, P<0.01	n/a	n/a
Wu et al. 2010 [Lifestyle Counseling Alone]	0.09 [-0.09, 0.28] P=0.321<br>63.6%, P<0.01	-0.04 [-0.12, 0.05] P=0.403<br>32.7%, P<0.01	n/a	0.03 [-0.09, 0.15] P=0.652<br>69.2%, P<0.01	n/a	n/a
Zambon et al. 2000	0.09 [-0.09, 0.27] P=0.338<br>63.6%, P<0.01	-0.04 [-0.12, 0.05] P=0.412<br>32.7%, P<0.01	n/a	n/a	n/a	n/a

[a]Sensitivity analysis included the removal of each single study from the meta-analyses one at a time and the summary effect was recalculated. An influential outlier was considered a study whose removal changed the magnitude of the pooled effect by >10%.

[b]Visceral adipose tissue was assessed as standardized mean differences as the units presented in the individual trials differed and were not able to be converted into a common unit without standardization.

ALA= alpha-linoleic acid, BMI = body mass index, I^2 = heterogeneity, MD = mean difference.

Supplementary Table 12. Sensitivity analysis of the use of correlation coefficient of 0.25 and 0.75 for crossover trials.

	MD (95% CI), P-value<br>I^2, P-value		
	Correlation Coefficient used in the Primary Analysis	Correlation Coefficient used in Sensitivity Analyses[a]	
	0.5	0.25	0.75
**Body Weight (kg)**<br>**N=105**	0.09 [-0.09, 0.27] P=0.340<br>63.2%, P<0.01	0.09 [-0.09, 0.28] P=0.332<br>62.4%, P<0.01	0.08 [-0.09, 0.26] P=0.361<br>65.3%, P<0.01
**BMI (kg/m^2)**<br>**N=90**	-0.04 [-0.12, 0.05] P=0.411<br>32.7%, P<0.01	-0.03 [-0.12, 0.05] P=0.428<br>28.9%, P=0.007	-0.04 [-0.12, 0.05] P=0.382<br>39.3%, P<0.01
**Body Fat (%)**<br>**N=43**	-0.05 [-0.42, 0.31] P=0.766<br>77.0%, P<0.01	-0.06 [-0.43, 0.31] P=0.746<br>76.9%, P<0.01	-0.04 [-0.39, 0.30] P=0.803<br>77.4%, P<0.01
**Waist Circumference (cm)**<br>**N=58**	0.03 [-0.09, 0.15] P=0.637<br>68.6%, P<0.01	0.02 [-0.10, 0.13] P=0.760<br>67.9%, P<0.01	0.05 [-0.06, 0.17] P=0.364<br>70.6%, P<0.01
**Waist-to-Hip Ratio**<br>**N=14**	-0.01 [-0.04, 0.01] P=0.312<br>84.0%, P<0.01	-0.01 [-0.04, 0.01] P=0.323<br>83.8%, P<0.01	-0.01 [-0.04, 0.01] P=0.285<br>84.6%, P<0.01
**Visceral Adipose Tissue[b]**<br>**N=9**	-0.59 [-1.32, 0.14] P=0.114<br>64.7%, P=0.004	-0.66 [-1.45, 0.13] P=0.104<br>64.2%, P=0.004	-0.49 [-1.13, 0.15] P=0.134<br>65.4%, P=0.003

[a]Sensitivity analysis was conducted using different correlation coefficient values (0.25 and 0.75) to test for the robustness of the effect size.
[b]Visceral adipose tissue was assessed as standardized mean differences as the units presented in the individual trials differed and were not able to be converted into a common unit without standardization.
ALA= alpha-linoleic acid, BMI = body mass index, I^2 = heterogeneity, MD = mean difference.
One of these crossover trials, however, did not require the use of a correlation coefficient as complete data was available
BMI, body mass index; CI, confidence interval; MD, mean difference; no., number

Supplementary Table 13. GRADE assessments for the prospective cohort studies.

Certainty assessment								Relative risk (95% CI)	Certainty*
**Adiposity outcome**	**No. cohort comparisons**	**Study design**	**Risk of bias**	**Inconsistency**	**Indirectness**	**Imprecision**	**Other considerations**		
**Overweight/ obesity incidence**	5	observational studies	not serious	not serious[a]	not serious	not serious	dose-response	**RR 0.93** (0.88, 0.98)	⊕⊕⊕◯ MODERATE
**Body weight change**	5	observational studies	serious[b]	serious[c]	not serious	not serious	none	**MD -0.46** (-0.78, -0.13)	⊕◯◯◯ VERY LOW
**Weight gain (≥ 5 kg) incidence**	3	observational studies	not serious[d]	not serious[e]	not serious	not serious	dose-response	**RR 0.95** (0.94, 0.96)	⊕⊕⊕◯ MODERATE
**Waist circumference incidence**[†]	2	observational studies	not serious	not serious[f]	not serious	not serious	dose-response	**RR 0.72** (0.65, 0.80)	⊕⊕⊕◯ MODERATE

CI=confidence interval, MD=mean difference, No.=number, RR=risk ratio

*All outcomes started with low quality evidence since all studies were observational. Risk of Bias –Risk of bias was rated down if the majority of studies were considered to be at high risk of bias (NOS<6). Inconsistency –Inconsistency was assessed using I^2 estimates where an I^2 of 50% or higher indicates substantial heterogeneity. I^2 is the percentage of variability in the treatment estimates that is attributable to heterogeneity between studies. Inconsistency was rated down if there was substantial heterogeneity that was unexplained by any *a priori* sensitivity or subgroup analyses. Indirectness –Indirectness was rated down if there were factors present relating to the population and outcomes that limited the generalizability of the results. Imprecision –Imprecision was rated down if the 95% confidence interval (95% CI) crossed the minimally important difference (MID) for harm. MIDs used for each outcome are: RR=0.1 (or 10%) for overweight/obesity risk, weight gain (≥5 kg) risk, and waist circumference risk, and 0.5 kg for body weight based on (103).

[†]Waist circumference incidence represents the incidence of increasing ≥ 94 cm for men and ≥80 cm for women.

a. No serious inconsistency for overweight/obesity incidence, as I^2 = 90% and P<0.01 was explained by sensitivity analysis and the removal of the Adventist Health Study-2 (AHS-2), which involved >50% vegetarian participants. Removal of this cohort reduced the heterogeneity from substantial to non-substantial (I^2 = 14%, P-heterogeneity=0.32) without altering the direction, significance or magnitude of the pooled risk estimate (RR 0.96 [95% CI 0.95 to 0.98], P<0.001).

b. Serious risk of bias for body weight change, as >50% of the weight (78.9%) was contributed by studies considered to be high risk of bias (NOS<6).

c. Serious inconsistency for body weight change, as I^2 = 95.9% and P<0.01 and this was unexplained by sensitivity analysis.

d. Not serious risk of bias for weight gain (≥5 kg) incidence, even though >50% of the weight (66.7%) was contributed by studies with a NOS <6, data was not available to assess comparability of one of the 3 studies. Of the seven criteria that could be evaluated, a NOS of 4 was determined which is equivalent to a NOS evaluation of 6/9 (i.e. 66.7%).

e. No serious inconsistency for weight gain (≥5 kg) incidence, while overall I^2>50% (i.e. I^2 = 53%) P=0.12, and this was explained by sensitivity analysis, specifically, the removal of the (4) SUN cohort assessment due to the large variation of the result.

f. No serious inconsistency for waist circumference increase incidence, while overall I2 = 62% and P<0.10 and this could be explained by sensitivity analysis as there were only 2 comparisons and the difference was males compared with females.

Supplementary Table 14. GRADE assessment of certainty of evidence for the outcomes of interest of randomized controlled trials.

Certainty assessment								Mean Difference (95% CI)	Certainty*
**Adiposity outcome**	**No. trial comparisons**	**Study design**	**Risk of bias**	**Inconsistency**	**Indirectness**	**Imprecision**	**Other considerations**		
**Body weight (kg)**	105	randomized trials	not serious	serious[a]	not serious	not serious	dose-response	**MD 0.09** (-0.09, 0.27)	⨁⨁⨁⨁ HIGH
**BMI (kg/m²)**	90	randomized trials	not serious	not serious	not serious	not serious	none	**MD -0.04** (-0.12, 0.05)	⨁⨁⨁⨁ HIGH
**Body fat (%)**	43	randomized trials	not serious	serious[b]	not serious	not serious	dose-response	**MD -0.05** (-0.42, 0.31)	⨁⨁⨁⨁ HIGH
**WC (cm)**	58	randomized trials	not serious	serious[c]	not serious	not serious	none	**MD 0.03** (-0.09, 0.15)	⨁⨁⨁◯ MODERATE
**Waist-to-hip ratio**	14	randomized trials	not serious	serious[d]	not serious	not serious	none	**MD -0.01** (-0.04, 0.01)	⨁⨁⨁◯ MODERATE
**VAT**	9	randomized trials	not serious	serious[e]	not serious	not serious	none	**SMD -0.59** (-1.32, 0.14)	⨁⨁⨁◯ MODERATE

CI=confidence interval, MD=mean difference, No.=number, RR=risk ratio, SMD= standardized mean difference.
*All outcomes started with high quality evidence since all studies were randomized controlled trials. Risk of Bias –We rated down for risk of bias if the majority of studies were considered to be at high risk of bias. Inconsistency – We assessed inconsistency using I^2 estimates where an I^2 of 50% or higher indicates substantial heterogeneity. I^2 is the percentage of variability in the treatment estimates that is attributable to heterogeneity between studies. We rated down for inconsistency if there was substantial heterogeneity that was unexplained by any *a priori* sensitivity or subgroup analyses. Indirectness – We rated down for indirectness if there were factors present relating to the population, interventions, and outcomes that limited the generalizability of the results. Imprecision – We rated down for imprecision if the 95% confidence interval (95% CI) crossed the minimally important difference (MID) for harm. MIDs used for each outcome are: 0.5 kg for body weight based on Johnston et al. 2014; 0.2 kg/m² for BMI; 2.0 cm for waist circumference; 2.0% for body fat; 0.02 for waist-to-hip ratio; 0.2 for visceral adipose tissue.
a. Serious inconsistency for body weight, as I^2 = 63% and P<0.01 and this was unexplained by sensitivity analysis.
b. Serious inconsistency for body fat percentage, as I^2 = 77% and P<0.01 and this was unexplained by sensitivity analysis.
c. Serious inconsistency for waist circumference, as I^2 = 69% and P<0.01 and this was unexplained by sensitivity analysis.
d. Serious inconsistency for waist-to-hip ratio, as I^2 = 84% and P<0.01 and this was unexplained by sensitivity analysis.
e. Serious inconsistency for visceral adipose tissue, as I^2 = 65% and P<0.01 and this was unexplained by sensitivity analysis.

SUPPLEMENTARY FIGURES

Supplementary Figure 1. Cochrane risk of bias summary for all included randomized controlled trials (continued on the next page).

	Random sequence generation (selection bias)	Allocation concealment (selection bias)	Blinding of participants, personnel, and/or outcome assessments	Incomplete outcome data (attrition bias)	Selective reporting (reporting bias)
Abazarfard et al. 2013	+	?	?	−	?
Abbaspour et al. 2019	−	?	?	?	?
Agebratt et al. 2016	+	?	+	+	+
Baer et al. 2019	+	?	+	?	+
Balci et al. 2015	?	?	?	?	?
Bamberger et al. 2017	?	?	+	?	+
Barbour et al. 2015	+	?	+	?	+
Bento et al. 2014	?	?	?	?	+
Berryman et al. 2015	+	?	+	?	+
Bitok et al. 2018	+	?	+	+	−
Biude Silva Duarte et al. 2019	?	+	?	?	+
Bowen et al. 2019	+	+	+	?	+
Campbell et al. 2019	+	?	−	?	+
Canales et al. 2007	?	?	−	+	+
Carughi et al. 2019	+	?	−	?	?

Supplementary Figure 1. Cochrane risk of bias summary for all included randomized controlled trials (continued on the next page).

Casas-Agustench et al. 2011	?	?	?	+	+
Chisholm et al. 2005	?	?	?	?	?
Ciccone et al. 2014 [Control]	?	?	?	?	?
Ciccone et al. 2014 [Fish]	?	?	?	?	?
Ciccone et al. 2014 [Olive oil]	?	?	?	?	?
Cohen et al. 2011	?	?	?	+	?
Damasceno et al. 2011 [Almond]	+	+	+	?	+
Damasceno et al. 2011 [Walnut]	+	+	+	?	+
Damavandi et al. 2012	?	?	?	?	?
Damavandi et al. 2013	?	?	?	+	?
de Souza et al. 2018	+	+	?	?	?
Dhillon et al. 2016	?	?	?	?	+
Dhillon et al. 2018	+	+	+	?	+
Foster et al. 2012	+	?	?	+	+
Gebauer et al. 2008 [pistachios 10%E]	?	?	+	+	?
Gebauer et al. 2008 [pistachios 20%E]	?	?	+	+	?
Gulati et al. 2014	?	?	?	+	+
Hernandez-Alonso et al. 2014	+	?	+	+	+
Hiraoka-Yamamoto et al. 2004 [Butter]	?	?	?	?	?
Hiraoka-Yamamoto et al. 2004 [Coconut]	?	?	?	?	?
Hollis and Mattes 2007	?	?	?	?	?
Hudthagosol et al. 2012 [Control]	?	?	+	?	?
Hudthagosol et al. 2012 [Salmon]	?	?	+	?	?
Hwang et al. 2019	+	?	+	−	+
Jamshed et al. 2015 [Pak]	+	?	?	?	+
Jamshed et al. 2015 [US]	+	?	?	?	+
Jenkins et al. 2002 [Full Dose Almonds]	?	?	+	?	+
Jenkins et al. 2002 [Half Dose Almonds]	?	?	+	?	+

Supplementary Figure 1. Cochrane risk of bias summary for all included randomized controlled trials (continued on the next page).

Jenkins et al. 2018 [Full-dose nut]	+	+	+	+	+
Jenkins et al. 2018 [Half-dose nut]	+	+	+	+	+
Johnston et al. 2013	?	?	?	?	?
Jung et al. 2018	?	−	?	+	?
Katz et al. 2012	?	?	+	+	+
Kocyigit et al. 2006	?	?	?	+	?
Lee et al. 2014	?	?	−	+	+
Le et al. 2016 [Lower CHO]	?	?	?	?	?
Le et al. 2016 [Lower Fat]	?	?	?	?	?
Li et al. 2010	?	?	?	?	+
Li et al. 2011	?	?	+	?	?
Liu et al. 2018	+	?	+	?	+
Ma et al. 2010	?	?	+	?	+
McKay et al. 2018	+	?	+	+	+
Mohan et al. 2018	+	?	?	+	+
Moreira Alves et al. 2014 [Conventional Peanut]	?	?	?	?	+
Moreira Alves et al. 2014 [High Oleic Peanut]	?	?	?	?	+
Morgan et al. 2000	?	?	?	?	?
Morgan et al. 2002	?	?	−	?	?
Nagashree et al. 2017	+	?	+	?	?
Njike et al. 2015 [Ad libitum]	+	?	+	+	+
Njike et al. 2015 [Calorie adjusted]	+	?	+	+	+
Njike et al. 2017	+	?	+	?	+
Parham et al. 2014	?	+	?	?	+
Rajaram et al. 2001	?	?	+	+	+
Robbins et al. 2012	+	+	+	+	+
Rock et al. 2017	?	?	?	?	+
Ros et al. 2004	?	?	?	+	+

Supplementary Figure 1. Cochrane risk of bias summary for all included randomized controlled trials (continued on the next page).

Ruisinger et al. 2015	?	?	?	+	+
Sabate et al. 2003 [High-dose nuts]	?	?	+	?	+
Sabate et al. 2003 [Low-dose nuts]	?	?	+	?	+
Sabate et al. 2005	?	?	?	?	+
Sauder et al. 2015	+	+	+	+	+
Schutte et al. 2006 [Cashew]	+	?	+	?	+
Schutte et al. 2006 [Walnut]	+	?	+	?	+
Sheridan et al. 2007	?	?	+	+	+
Somerset et al. 2013	+	+	?	?	?
Spaccarotella et al. 2008	?	?	?	+	?
Spiller et al. 1998 [Cheddar Cheese/Butter]	?	?	?	?	+
Spiller et al. 1998 [Olive Oil]	?	?	?	?	+
Sweazea et al. 2014	?	?	?	-	+
Tan & Mattes. 2013 [Afternoon Snack]	+	?	?	+	+
Tan & Mattes. 2013 [Breakfast]	+	?	?	+	+
Tan & Mattes. 2013 [Lunch]	+	?	?	+	+
Tan & Mattes. 2013 [Morning Snack]	+	?	?	+	+
Tapsell et al. 2004 [Low Fat]	?	?	?	+	+
Tapsell et al. 2004 [Modified Fat]	?	?	?	+	+
Tapsell et al. 2009	+	+	+	?	+
Tapsell et al. 2017 [Control]	+	+	+	-	+
Tapsell et al. 2017 [Intervention Control]	+	+	+	-	+
Tey et al. 2011 [Chocolate]	?	+	?	?	+
Tey et al. 2011 [Control]	?	+	?	?	+
Tey et al. 2011 [Crisps]	?	+	?	?	+
Tey et al. 2013 [30 g Hazelnuts]	+	?	+	+	?
Tey et al. 2013 [60 g Hazelnuts]	+	?	+	+	?

Supplementary Figure 1. Cochrane risk of bias summary for all included randomized controlled trials (continued on the next page).

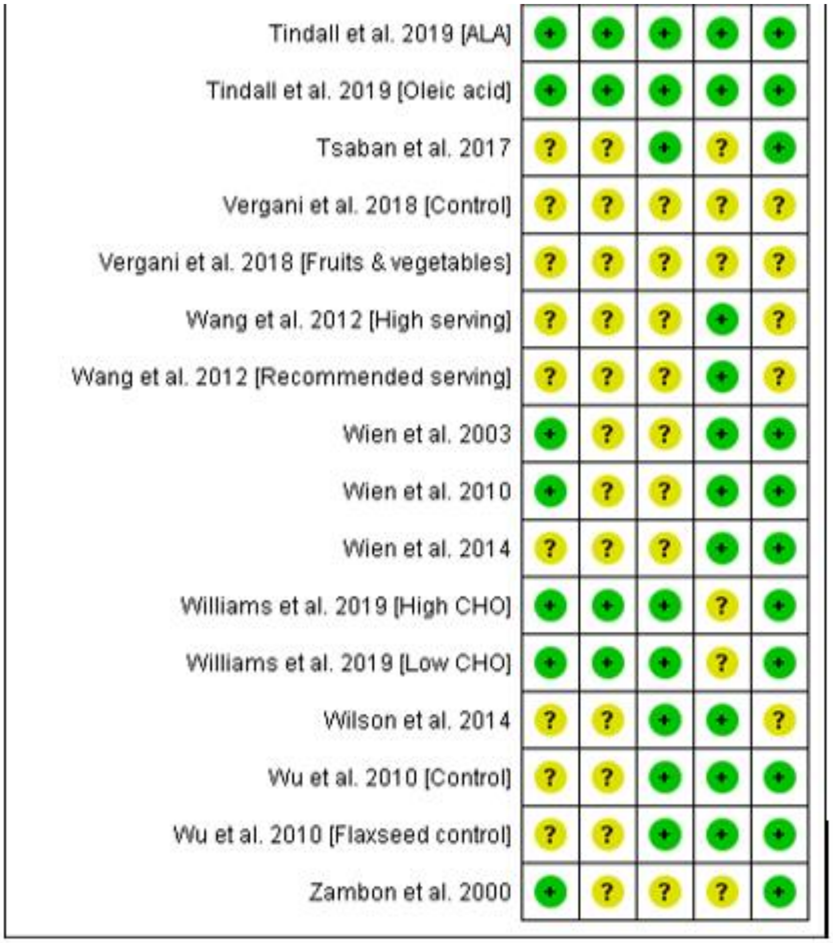

Coloured circles represent the domain for the corresponding trial assessed as low (green), unclear (yellow), or high (red) risk of bias for the 5 domains of bias noted above according to criteria set by the Cochrane Risk of Bias tool in the 114 randomized controlled trial comparisons. Where low risk of bias indicates proper methods reported being taken to reduce bias, high risk of bias indicates improper methods creating bias reported, and unclear indicates insufficient information provided to determine the bias level.
ALA, alpha linoleic acid; CHO, carbohydrate.

Supplementary Figure 2. Risk of bias proportion graph for all included randomized controlled trials

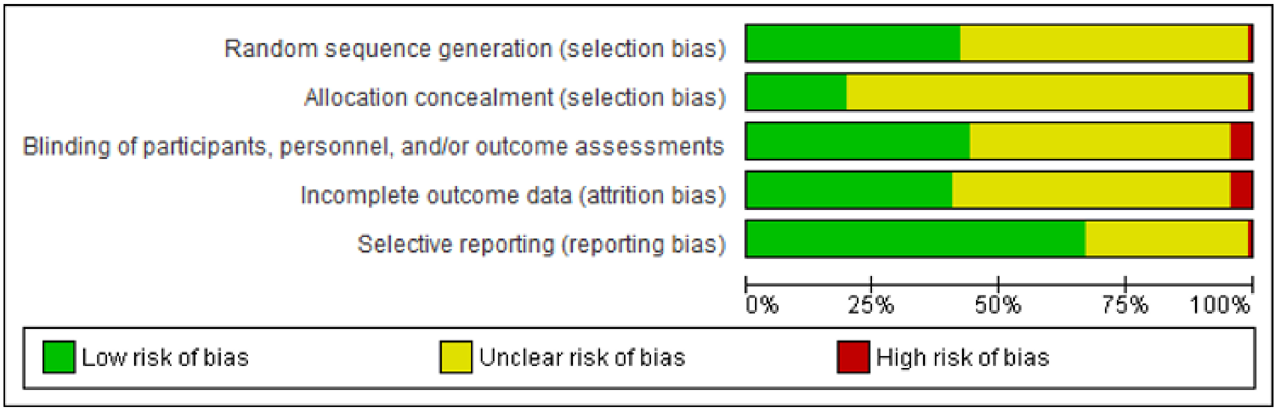

Coloured bars represent the proportion of studies assessed as low (green), unclear (yellow), or high (red) risk of bias for the 5 domains of bias noted above according to criteria set by the Cochrane Risk of Bias tool in the 115 randomized controlled trial comparisons.

Supplementary Figure 3. Forest plot of prospective cohorts investigating the association of nut consumption on overweight/obesity risk.

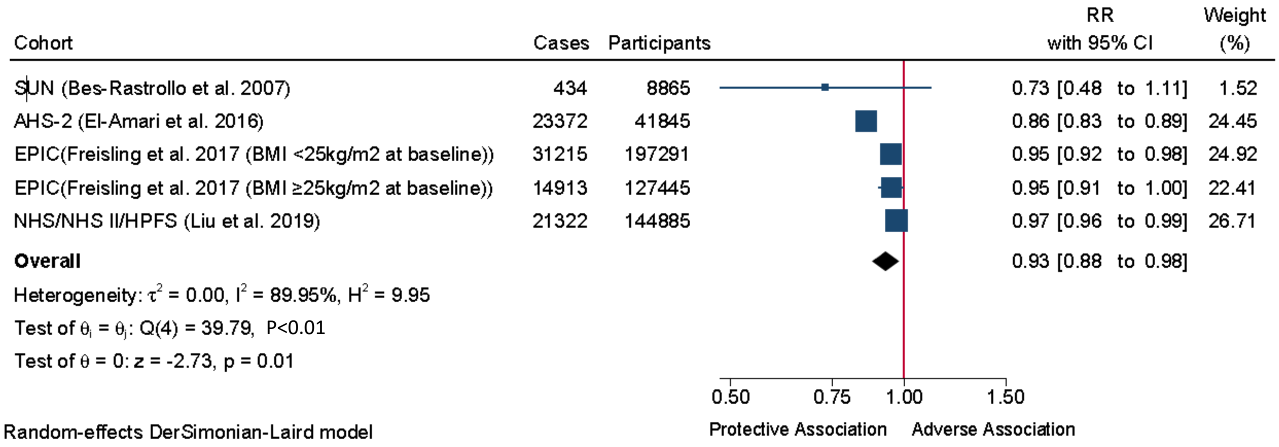

AHS-2=Adventist Health Study 2, EPIC -PANACEA= European Prospective Investigation into Cancer and Nutrition – Physical Activity, Nutrition, Alcohol, Cessation of smoking, Eating out of home in relation to Anthropometry, HPFS = Health Professionals Follow-Up Study, NHS = Nurses' Health Study, NHS II = Nurses' Health Study II, Sun = Seguimiento Universidad de Navarra study.
The black diamond represents the pooled risk estimate. Inter-study heterogeneity was tested using the Cochran Q statistic at a significance level of p<0.10, and quantified by the I^2 statistic. An I^2 value ≥50% is considered as indicative of substantial heterogeneity. All results are presented as Relative Risks (RR) with 95% Confidence Intervals (CI).

Supplementary Figure 4a. Forest plot of prospective cohorts investigating the association of nut consumption on body weight change (kg).

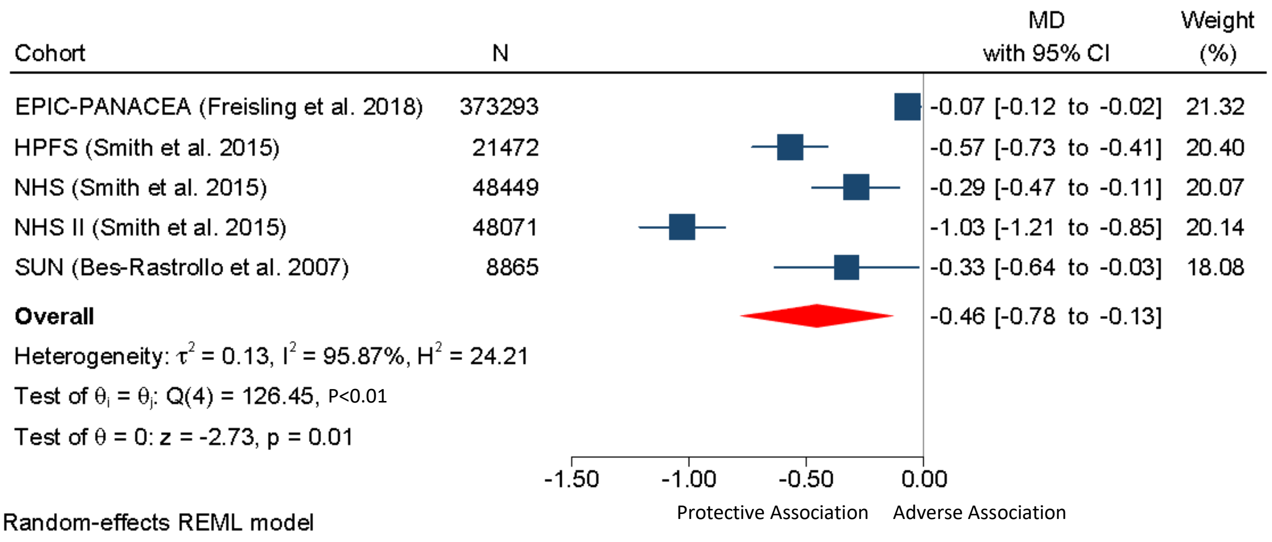

Supplementary Figure 4b. Forest plot of prospective cohorts investigating the association of nut consumption on body weight change (kg), using data from the least adjusted model.

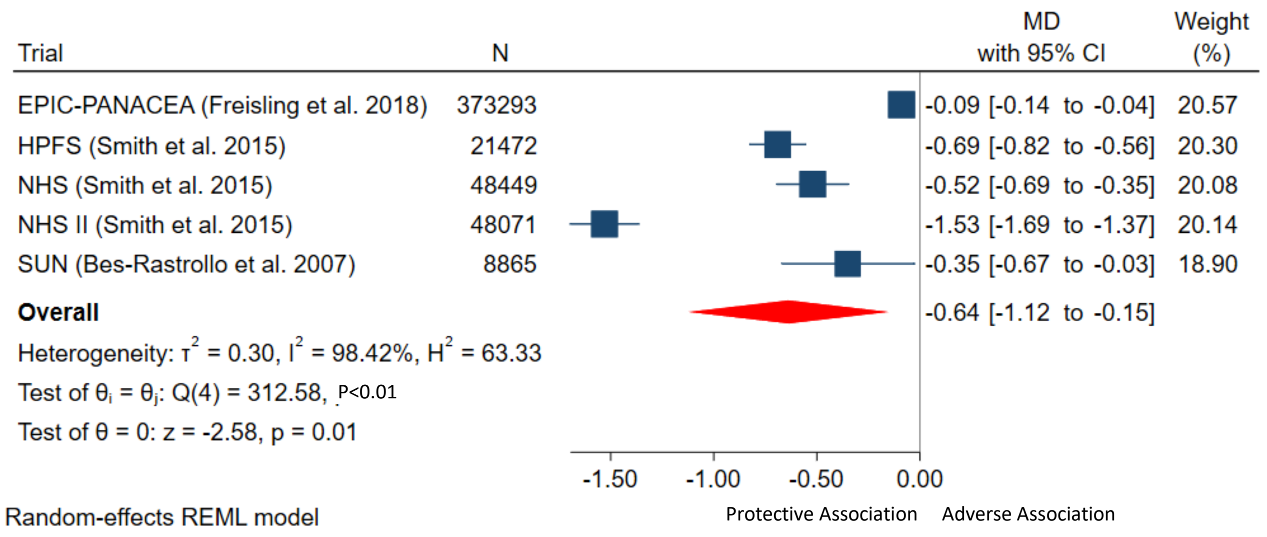

EPIC -PANACEA= European Prospective Investigation into Cancer and Nutrition – Physical Activity, Nutrition, Alcohol, Cessation of smoking, Eating out of home in relation to Anthropometry, HPFS = Health Professionals Follow-Up Study, NHS = Nurses' Health Study, NHS II = Nurses' Health Study II, Sun = Seguimiento Universidad de Navarra study.

The black diamond represents the pooled risk estimate. Inter-study heterogeneity was tested using the Cochran Q statistic at a significance level of $p<0.10$, and quantified by the I^2 statistic. An I^2 value ≥50% is considered as indicative of substantial heterogeneity. All results are presented as Mean Differences (MD) with 95% Confidence Intervals (CI).

Supplementary Figure 5. Forest plot of prospective cohorts investigating the association of nut consumption on weight gain (≥5 kg) incidence.

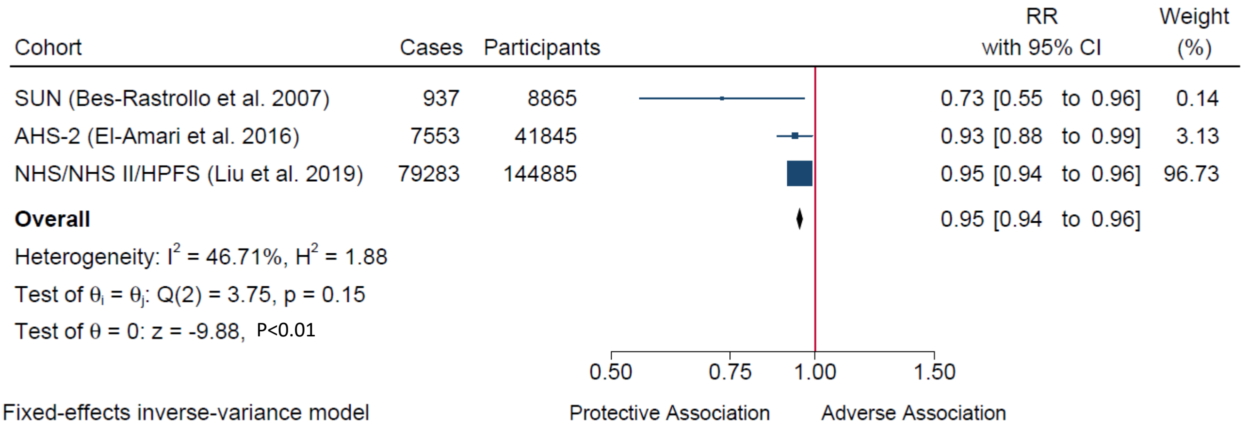

AHS-2=Adventist Health Study 2, HPFS = Health Professionals Follow-Up Study, NHS = Nurses' Health Study, NHS II = Nurses' Health Study II, Sun = Seguimiento Universidad de Navarra study.
The black diamond represents the pooled risk estimate. Inter-study heterogeneity was tested using the Cochran Q statistic at a significance level of p<0.10, and quantified by the I^2 statistic. An I^2 value ≥50% is considered as indicative of substantial heterogeneity. All results are presented as Relative Risks (RR) with 95% Confidence Intervals (CI).

Supplementary Figure 6. Forest plot of prospective cohorts investigating the association of nut consumption on the incidence of waist circumference increasing ≥94 cm in men and ≥80 cm in women.

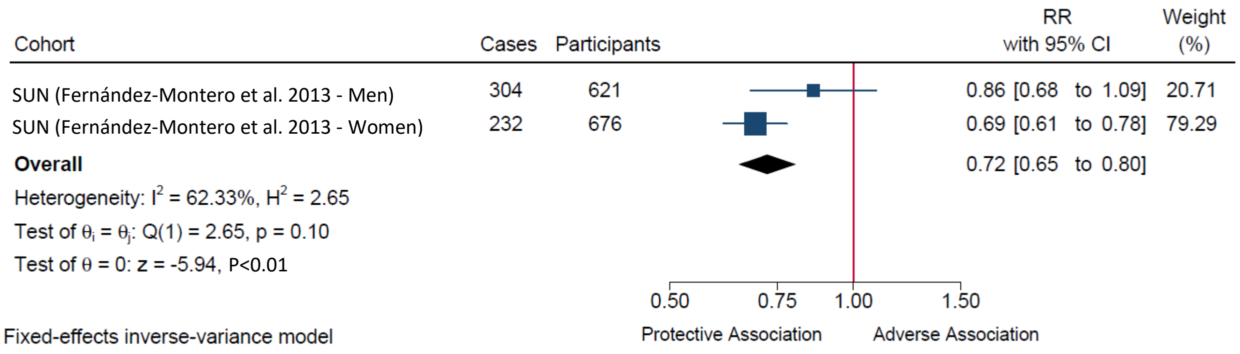

Sun = Seguimiento Universidad de Navarra study.
The black diamond represents the pooled risk estimate. Inter-study heterogeneity was tested using the Cochran Q statistic at a significance level of $p<0.10$, and quantified by the I^2 statistic. An I^2 value ≥50% is considered as indicative of substantial heterogeneity. All results are presented as Relative Risks (RR) with 95% Confidence Intervals (CI).

Supplementary Figure 7. Forest plot of randomized controlled trials investigating the effects of nut consumption on body weight (kg) (continued on the next page).

Trial	N	MD with 95% CI	Weight (%)
Abazarfard et al. 2014	100	-2.41 [-3.68 to -1.14]	1.38
Abbaspour et al. 2019	48	-2.00 [-9.41 to 5.41]	0.06
Agebratt et al. 2016	30	-0.03 [-6.43 to 6.37]	0.08
Baer et al. 2019	40	0.00 [-0.19 to 0.19]	3.98
Balci et al. 2012	60	0.90 [-5.81 to 7.61]	0.07
Bamberger et al. 2017	204	0.10 [-1.61 to 1.81]	0.90
Barbour et al. 2015	61	0.50 [0.11 to 0.89]	3.50
Bento et al. 2014	20	-0.30 [-5.26 to 4.66]	0.13
Berryman et al. 2015	48	-0.30 [-3.24 to 2.64]	0.35
Bitok et al. 2018	317	0.20 [-3.44 to 3.84]	0.24
Biude Silva Duarte et al. 2019	55	-0.70 [-1.65 to 0.25]	1.96
Bowen et al. 2019	76	0.10 [-0.61 to 0.81]	2.55
Campbell et al. 2019	12	-6.10 [-17.82 to 5.62]	0.02
Canales et al. 2007	22	-0.20 [-19.62 to 19.22]	0.01
Carughi et al. 2019	60	0.20 [-2.84 to 3.24]	0.33
Casas-Agustench et al. 2011	50	-0.70 [-2.11 to 0.71]	1.19
Chisholm et al. 2005	28	0.00 [-0.52 to 0.52]	3.11
Ciccone et al. 2014 [Control]	31	-3.70 [-17.90 to 10.50]	0.02
Ciccone et al. 2014 [Non-fried fish]	15	-2.00 [-17.87 to 13.87]	0.01
Ciccone et al. 2014 [Olive Oil]	15	-6.40 [-23.19 to 10.39]	0.01
Cohen et al. 2011	13	-2.80 [-30.31 to 24.71]	0.00
Damasceno et al. 2011 [Almond]	18	0.11 [-6.82 to 7.04]	0.07
Damasceno et al. 2011 [Walnut]	18	0.06 [-6.82 to 6.94]	0.07
Damavandi et al. 2012	43	-0.90 [-7.48 to 5.68]	0.08
Damavandi et al. 2013	48	-0.32 [-5.89 to 5.25]	0.10
DeSouza et al. 2018	46	-0.34 [-8.29 to 7.61]	0.05
Dhillon et al. 2016	86	-1.13 [-2.41 to 0.15]	1.38
Dhillon et al. 2018	73	-0.60 [-0.85 to -0.35]	3.86
Foster et al. 2012	123	2.20 [-0.57 to 4.97]	0.39
Gebauer et al. 2008 [High Dose Pistachio]	28	-0.30 [-8.83 to 8.23]	0.05
Gebauer et al. 2008 [Low Dose Pistachio]	28	-0.10 [-8.63 to 8.43]	0.05
Gulati et al. 2014	68	-1.10 [-6.25 to 4.05]	0.12
Hernández-Alonso et al. 2014	49	0.19 [-0.30 to 0.68]	3.19
Hiraoka-Yamamoto et al. 2004 [Butter]	35	-0.40 [-4.43 to 3.63]	0.20
Hiraoka-Yamamoto et al. 2004 [Coconut]	36	-0.30 [-4.27 to 3.67]	0.20
Hollis and Mattes. 2007	20	0.80 [-3.59 to 5.19]	0.17
Hudthagosol et al. 2012 [Control]	25	1.70 [-0.69 to 4.09]	0.51
Hudthagosol et al. 2012 [Salmon]	25	0.30 [-2.10 to 2.70]	0.51

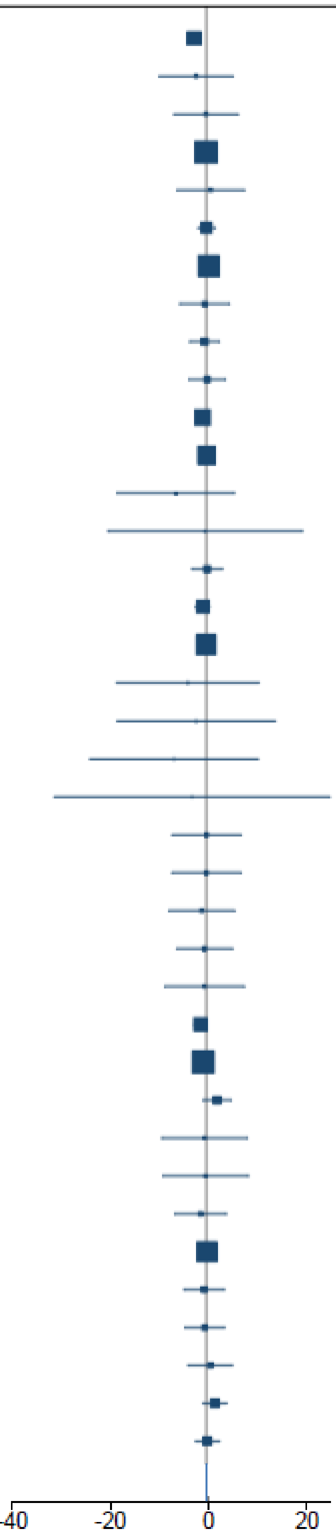

Favours Nuts Favours Control

Supplementary Figure 7. Forest plot of randomized controlled trials investigating the effects of nut consumption on body weight (kg) (continued on the next page).

Trial	N	MD with 95% CI	Weight (%)
Jamshed et al. 2015 [American Almonds]	75	1.00 [-0.41 to 2.41]	1.20
Jamshed et al. 2015 [Pakistani Almonds]	72	5.00 [3.59 to 6.41]	1.20
Jenkins et al. 2002 [Full Dose Almonds]	27	-0.40 [-7.26 to 6.46]	0.07
Jenkins et al. 2002 [Half Dose Almonds]	27	-0.40 [-7.12 to 6.32]	0.07
Jenkins et al. 2018 [Full Dose Nuts]	100	-0.33 [-1.09 to 0.43]	2.41
Jenkins et al. 2018 [Half Dose Nuts]	100	0.40 [-0.39 to 1.19]	2.34
Johnston et al. 2013	44	1.10 [0.12 to 2.08]	1.90
Jung et al. 2018	84	-0.20 [-2.08 to 1.68]	0.77
Katz et al. 2012	46	1.05 [0.42 to 1.67]	2.79
Le et al. 2016 [Lower CHO]	137	-1.10 [-5.94 to 3.74]	0.14
Le et al. 2016 [Lower Fat]	147	0.70 [-3.64 to 5.04]	0.17
Lee et al. 2014	60	0.01 [-0.56 to 0.58]	2.98
Li et al. 2010	52	-1.00 [-6.50 to 4.50]	0.11
Liu et al. 2018	85	-0.54 [-1.59 to 0.51]	1.76
Ma et al. 2010	22	-0.60 [-1.78 to 0.58]	1.51
McKay et al. 2018	26	-0.24 [-5.25 to 4.77]	0.13
Mohan et al. 2018	269	0.32 [-0.02 to 0.66]	3.65
Moreira Alves et al. 2014 [Conventional Peanut]	44	0.78 [-0.34 to 1.90]	1.62
Moreira Alves et al. 2014 [High Oleic Peanut]	43	0.54 [-0.63 to 1.71]	1.55
Morgan et al. 2000	19	0.00 [-10.59 to 10.59]	0.03
Nagashree et al. 2017	58	0.12 [-4.48 to 4.72]	0.15
Njike et al. 2017	34	-0.20 [-1.44 to 1.04]	1.42
Rajaram et al. 2001	23	-0.43 [-0.50 to -0.36]	4.14
Robbins et al. 2012	109	0.25 [-0.59 to 1.09]	2.22
Rock et al. 2017	97	0.60 [-1.52 to 2.72]	0.63
Ros et al. 2004	20	0.20 [-4.30 to 4.70]	0.16
Ruisinger et al. 2015	48	0.45 [-7.39 to 8.30]	0.05
Sabate et al. 2003 [High Dose Almond]	25	-0.30 [-7.78 to 7.18]	0.06
Sabate et al. 2003 [Low Dose Almond]	25	0.20 [-7.28 to 7.68]	0.06
Sabaté et al. 2005	90	0.40 [0.20 to 0.60]	3.98
Sauder et al. 2015	30	-0.10 [-6.76 to 6.56]	0.07
Schutte et al. 2006 [Cashew]	42	0.34 [-0.74 to 1.42]	1.69
Schutte et al. 2006 [Walnut]	41	0.29 [-0.57 to 1.15]	2.17
Sheridan et al. 2007	15	0.00 [-5.77 to 5.77]	0.10
Somerset et al. 2013	64	-4.27 [-13.01 to 4.47]	0.04
Spaccarotella et al. 2008	21	0.90 [-4.74 to 6.54]	0.10

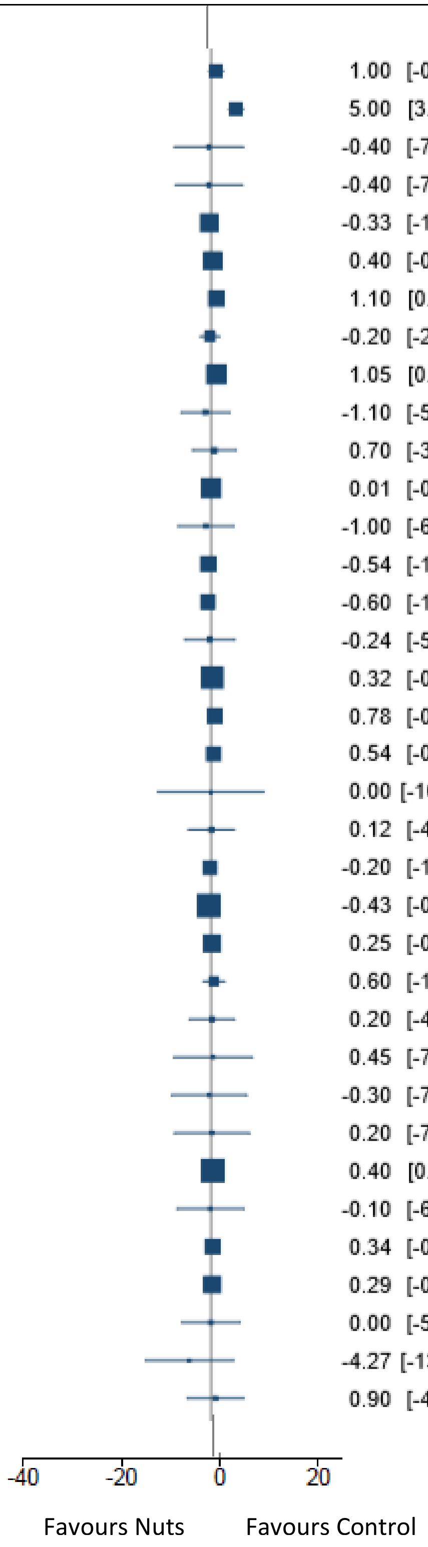

Supplementary Figure 7. Forest plot of randomized controlled trials investigating the effects of nut consumption on body weight (kg) (continued on next page).

Trial	N	MD with 95% CI	Weight (%)
Spiller et al. 1998 [Cheddar Cheese/Butter]	30	1.00 [-9.53 to 11.53]	0.03
Spiller et al. 1998 [Olive oil]	33	2.00 [-9.23 to 13.23]	0.03
Sweazea et al. 2014	21	1.18 [-1.35 to 3.71]	0.46
Tan and Mattes. 2013 [Afternoon Snack Almonds]	55	0.10 [-13.91 to 14.11]	0.02
Tan and Mattes. 2013 [Breakfast Almonds]	55	0.20 [-13.76 to 14.16]	0.02
Tan and Mattes. 2013 [Lunch Almonds]	53	0.30 [-13.54 to 14.14]	0.02
Tan and Mattes. 2013 [Morning Snack Almonds]	55	0.00 [-15.04 to 15.04]	0.01
Tapsell et al. 2004 [Low Fat]	28	-1.68 [-11.47 to 8.11]	0.03
Tapsell et al. 2004 [Modified Fat]	27	-1.09 [-11.37 to 9.19]	0.03
Tapsell et al. 2009	35	-0.70 [-11.30 to 9.90]	0.03
Tapsell et al. 2017 [Control]	96	-1.96 [-10.11 to 6.20]	0.05
Tapsell et al. 2017 [Intervention Alone]	80	-0.33 [-9.72 to 9.07]	0.04
Tey et al. 2011 [Chocolate]	63	0.24 [-0.90 to 1.38]	1.59
Tey et al. 2011 [Control]	61	0.37 [-0.58 to 1.32]	1.95
Tey et al. 2011 [Potato crisp]	58	0.33 [-0.65 to 1.31]	1.90
Tey et al. 2013 [High Dose Hazelnuts]	74	0.20 [-9.74 to 10.14]	0.03
Tey et al. 2013 [Low Dose Hazelnuts]	70	0.00 [-8.53 to 8.53]	0.05
Tindall et al. 2019 [ALA]	45	-0.68 [-1.68 to 0.32]	1.86
Tindall et al. 2019 [Oleic Acid]	45	0.00 [-1.27 to 1.27]	1.38
Tsaban et al. 2017	71	-2.60 [-5.57 to 0.37]	0.35
Wang et al. 2012 [High Dose Pistachio]	45	0.55 [-22.84 to 23.93]	0.01
Wang et al. 2012 [Recommended Dose Pistachio]	45	0.00 [-23.64 to 23.64]	0.01
Wien et al. 2003	65	-7.45 [-10.05 to -4.86]	0.44
Wien et al. 2010	65	-1.60 [-7.79 to 4.59]	0.09
Wien et al. 2014	60	-0.10 [-11.00 to 10.80]	0.03
Williams et al. 2019 [High CHO]	24	-0.10 [-0.88 to 0.68]	2.36
Williams et al. 2019 [Low CHO]	24	0.40 [-0.19 to 0.99]	2.91
Wilson et al. 2014	22	0.54 [-0.25 to 1.33]	2.35
Wu et al. 2010 [Flaxseed & Lifestyle Counseling]	188	0.26 [-0.01 to 0.53]	3.82
Wu et al. 2010 [Lifestyle Counseling Alone]	189	-0.10 [-0.50 to 0.30]	3.48
Zambon et al. 2000	49	-0.20 [-3.63 to 3.23]	0.27
**Overall**		0.09 [-0.09 to 0.27]	

Heterogeneity: $\tau^2 = 0.21$, $I^2 = 63.23\%$, $H^2 = 2.72$

Test of $\theta_i = \theta_j$: $Q(104) = 282.88$, P<0.01

Test of $\theta = 0$: $z = 0.95$, $p = 0.34$

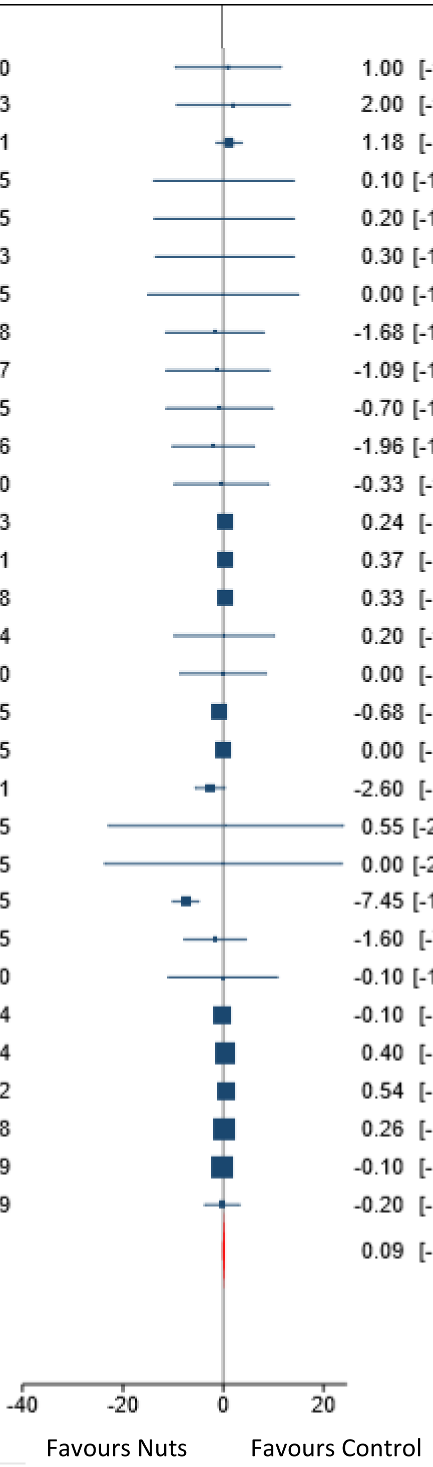

Random-effects DerSimonian-Laird model

Pooled effect estimate is represented by the diamond and was estimated with the use of a random-effects DerSimonian-Laird model.

Supplementary Figure 7. Forest plot of randomized controlled trials investigating the effects of nut consumption on body weight (kg).

To avoid unit of analysis error, standard error, used for determining the 95% confidence interval, was calculated by splitting the N for studies with multiple comparisons as per the Cochrane Handbook, 2019.
CI, confidence interval; MD, mean difference; N, number of participants.

Supplementary Figure 8. Forest plot of randomized controlled trials investigating the effects of nut consumption on BMI (kg/m^2) (continued on the next page).

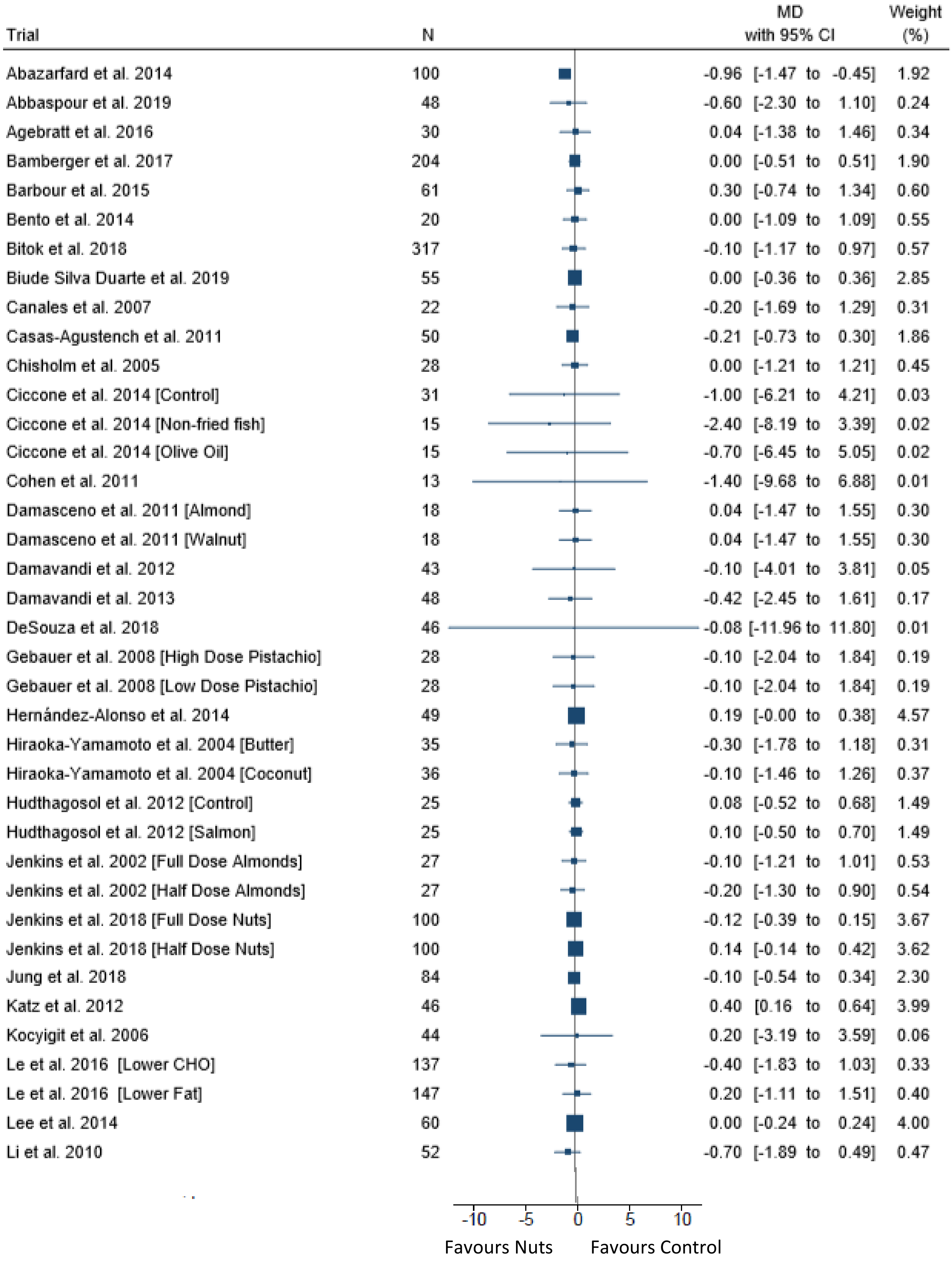

Supplementary Figure 8. Forest plot of randomized controlled trials investigating the effects of nut consumption on BMI (kg/m^2) (continued on next page).

Trial	N	MD with 95% CI	Weight (%)
Li et al. 2011	20	0.10 [-1.33 to 1.53]	0.34
Liu et al. 2018	85	-0.11 [-0.49 to 0.27]	2.74
Ma et al. 2010	22	-0.30 [-0.81 to 0.21]	1.88
McKay et al. 2018	26	-0.07 [-1.29 to 1.15]	0.45
Mohan et al. 2018	269	0.12 [-0.01 to 0.25]	5.20
Moreira Alves et al. 2014 [Conventional Peanut]	44	0.23 [-0.12 to 0.58]	2.94
Moreira Alves et al. 2014 [High Oleic Peanut]	43	0.15 [-0.21 to 0.51]	2.86
Morgan et al. 2000	19	0.00 [-4.05 to 4.05]	0.04
Morgan et al. 2002	67	0.00 [-1.96 to 1.96]	0.18
Nagashree et al. 2017	58	0.00 [-1.06 to 1.06]	0.58
Njike et al. 2015 [Ad libitum]	49	0.47 [0.03 to 0.91]	2.32
Njike et al. 2015 [Calorie adjusted]	48	0.19 [-0.44 to 0.82]	1.40
Njike et al. 2017	34	-0.10 [-0.54 to 0.34]	2.31
Parham et al. 2014	44	-0.50 [-0.87 to -0.13]	2.78
Robbins et al. 2012	109	-0.78 [-1.06 to -0.50]	3.59
Rock et al. 2017	97	0.20 [-1.33 to 1.73]	0.29
Ruisinger et al. 2015	48	0.30 [-1.51 to 2.11]	0.21
Sabaté et al. 2005	90	0.20 [0.00 to 0.40]	4.51
Sauder et al. 2015	30	0.00 [-2.16 to 2.16]	0.15
Schutte et al. 2006 [Cashew]	42	-0.20 [-3.13 to 2.73]	0.08
Schutte et al. 2006 [Walnut]	41	0.10 [-3.22 to 3.42]	0.07
Sheridan et al. 2007	15	0.14 [-1.55 to 1.83]	0.24
Somerset et al. 2013	64	-0.76 [-3.57 to 2.05]	0.09
Spaccarotella et al. 2008	21	0.30 [-6.76 to 7.36]	0.01
Sweazea et al. 2014	21	0.40 [-0.50 to 1.30]	0.78
Tan and Mattes. 2013 [Afternoon Snack Almonds]	55	0.20 [-3.65 to 4.05]	0.05
Tan and Mattes. 2013 [Breakfast Almonds]	55	0.20 [-3.56 to 3.96]	0.05
Tan and Mattes. 2013 [Lunch Almonds]	53	0.20 [-3.44 to 3.84]	0.05
Tan and Mattes. 2013 [Morning Snack Almonds]	55	0.10 [-3.71 to 3.91]	0.05
Tapsell et al. 2004 [Low Fat]	28	-0.66 [-3.49 to 2.17]	0.09
Tapsell et al. 2004 [Modified Fat]	27	-0.35 [-3.57 to 2.87]	0.07
Tapsell et al. 2017 [Control]	96	-1.20 [-3.97 to 1.57]	0.09
Tapsell et al. 2017 [Intervention Alone]	80	-0.70 [-3.41 to 2.01]	0.10
Tey et al. 2011 [Chocolate]	63	0.07 [-0.31 to 0.45]	2.70
Tey et al. 2011 [Control]	61	0.14 [-0.18 to 0.46]	3.22
Tey et al. 2011 [Potato crisp]	58	0.13 [-0.21 to 0.47]	3.02
Tey et al. 2013 [High Dose Hazelnuts]	74	0.00 [-2.66 to 2.66]	0.10
Tey et al. 2013 [Low Dose Hazelnuts]	70	0.00 [-2.62 to 2.62]	0.10

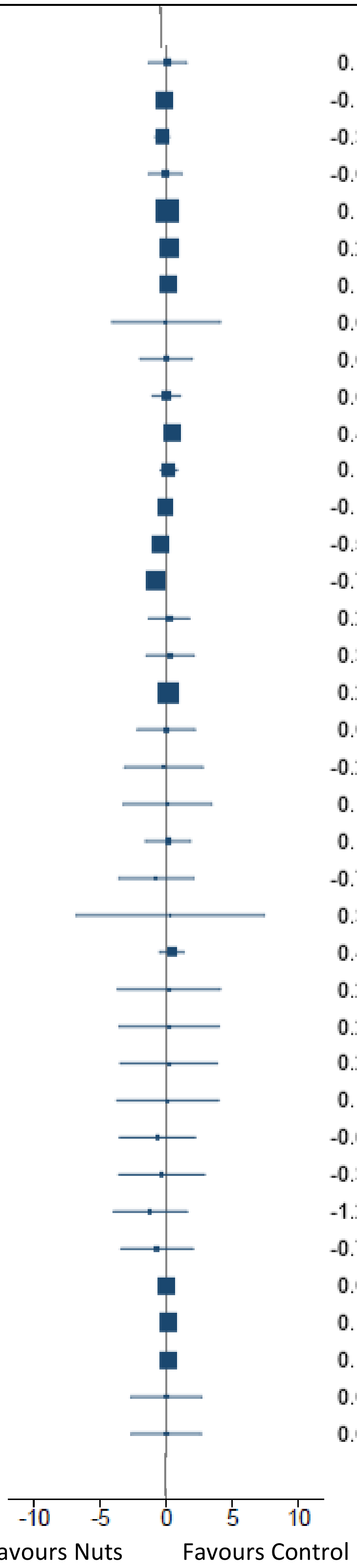

Supplementary Figure 8. Forest plot of randomized controlled trials investigating the effects of nut consumption on BMI (kg/m^2).

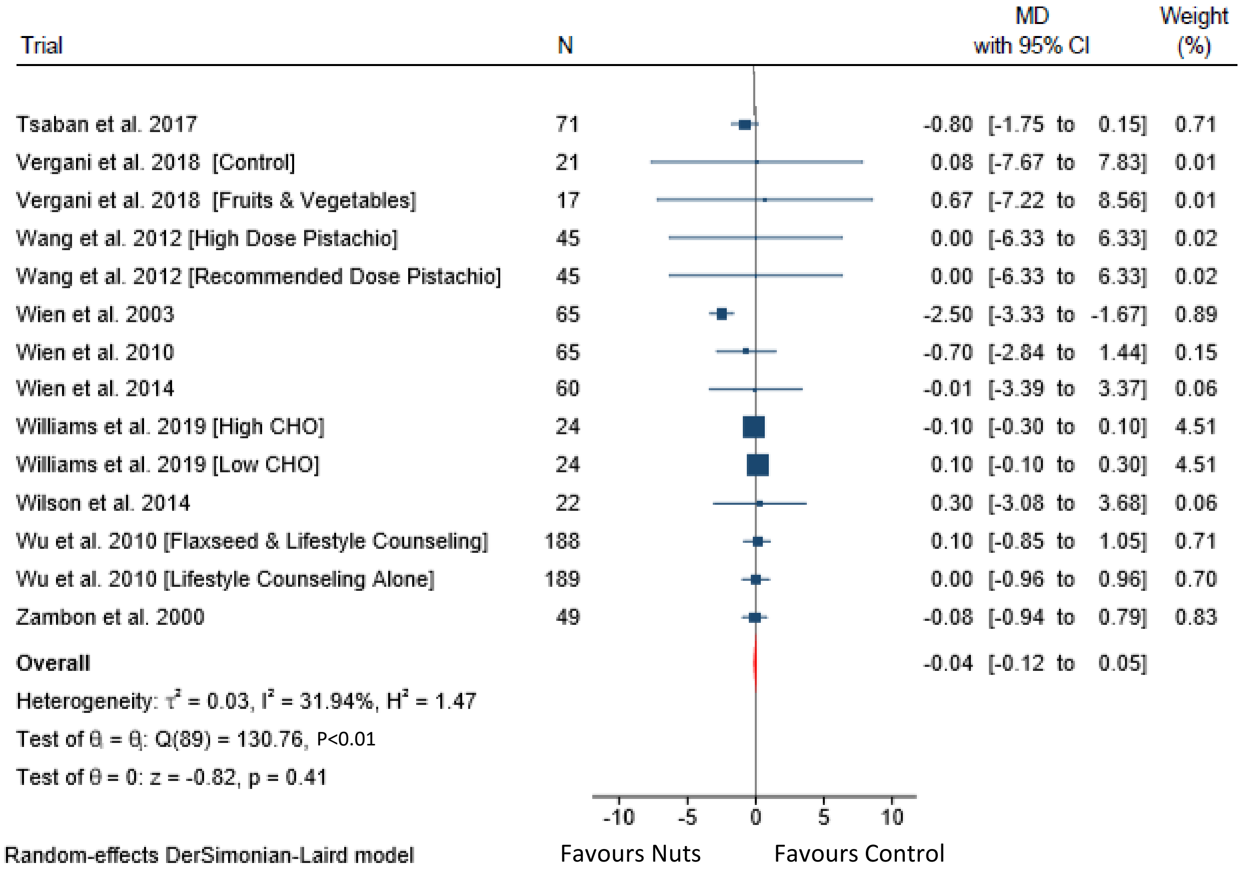

Pooled effect estimate is represented by the diamond and was estimated with the use of a random-effects DerSimonian-Laird model.
To avoid unit of analysis error, standard error, used for determining the 95% confidence interval, was calculated by splitting the N for studies with multiple comparisons as per the Cochrane Handbook, 2019.
BMI, body mass index; CI, confidence interval; MD, mean difference; N, number of participants.

Supplementary Figure 9. Forest plot of randomized controlled trials investigating the effects of nut consumption on body fat (%) (continued on the next page).

Trial	N	MD with 95% CI	Weight (%)
Abbaspour et al. 2019	48	0.00 [-4.95 to 4.95]	0.49
Barbour et al. 2015	61	0.30 [-0.09 to 0.69]	5.57
Bento et al. 2014	20	-0.90 [-5.37 to 3.57]	0.59
Berryman et al. 2015	48	-0.15 [-0.52 to 0.21]	5.62
Bitok et al. 2018	317	0.41 [0.05 to 0.77]	5.63
Biude Silva Duarte et al. 2019	55	0.40 [-1.00 to 1.80]	3.15
Bowen et al. 2019	76	-0.87 [-1.81 to 0.07]	4.25
Carughi et al. 2019	60	-0.50 [-3.02 to 2.02]	1.53
Casas-Agustench et al. 2011	50	-0.60 [-1.77 to 0.57]	3.67
Cohen et al. 2011	13	0.10 [-10.32 to 10.52]	0.12
Dhillon et al. 2018	73	0.20 [-4.08 to 4.48]	0.64
Foster et al. 2012	123	-0.04 [-0.51 to 0.43]	5.42
Hollis and Mattes. 2007	20	1.30 [-1.92 to 4.52]	1.04
Jenkins et al. 2002 [Full Dose Almonds]	27	-1.10 [-5.44 to 3.24]	0.62
Jenkins et al. 2002 [Half Dose Almonds]	27	-0.10 [-4.42 to 4.22]	0.63
Johnston et al. 2013	44	0.30 [-0.68 to 1.28]	4.15
Jung et al. 2018	84	-0.20 [-0.42 to 0.02]	5.83
Li et al. 2011	20	-0.80 [-4.99 to 3.39]	0.67
Liu et al. 2018	85	-0.74 [-1.89 to 0.41]	3.72
Moreira Alves et al. 2014 [Conventional Peanut]	44	-0.58 [-1.60 to 0.44]	4.04
Moreira Alves et al. 2014 [High Oleic Peanut]	43	-0.91 [-1.90 to 0.08]	4.13
Njike et al. 2015 [Ad libitum]	49	1.14 [-0.85 to 3.13]	2.13
Njike et al. 2015 [Calorie adjusted]	48	-0.19 [-1.38 to 1.00]	3.62
Njike et al. 2017	34	-7.90 [-14.71 to -1.09]	0.27
Sabaté et al. 2005	90	0.10 [-0.19 to 0.39]	5.74
Somerset et al. 2013	64	3.98 [2.89 to 5.07]	3.87
Sweazea et al. 2014	21	2.80 [-0.95 to 6.55]	0.81
Tan and Mattes. 2013 [Afternoon Snack Almonds]	55	-0.28 [-4.29 to 3.74]	0.72
Tan and Mattes. 2013 [Breakfast Almonds]	55	-0.08 [-4.10 to 3.94]	0.71
Tan and Mattes. 2013 [Lunch Almonds]	53	-0.34 [-4.33 to 3.65]	0.72
Tan and Mattes. 2013 [Morning Snack Almonds]	55	0.13 [-3.88 to 4.14]	0.72
Tapsell et al. 2004 [Low Fat]	28	-1.64 [-8.85 to 5.57]	0.24
Tapsell et al. 2004 [Modified Fat]	27	-0.86 [-8.59 to 6.87]	0.21
Tapsell et al. 2009	35	-0.60 [-5.73 to 4.53]	0.46
Tapsell et al. 2017 [Control]	96	-1.76 [-9.56 to 6.04]	0.21
Tapsell et al. 2017 [Intervention Alone]	80	-0.60 [-9.07 to 7.87]	0.18

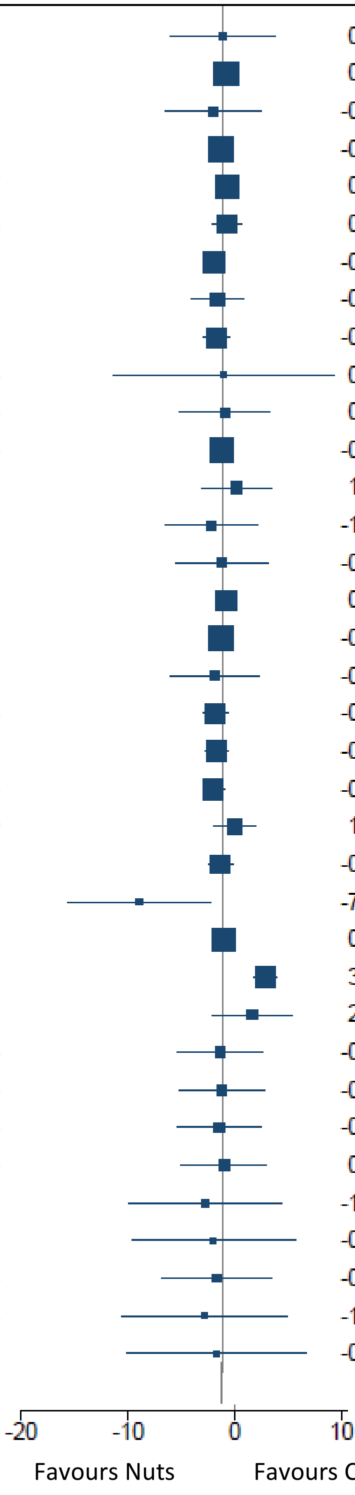

Supplementary Figure 9. Forest plot of randomized controlled trials investigating the effects of nut consumption on body fat (%).

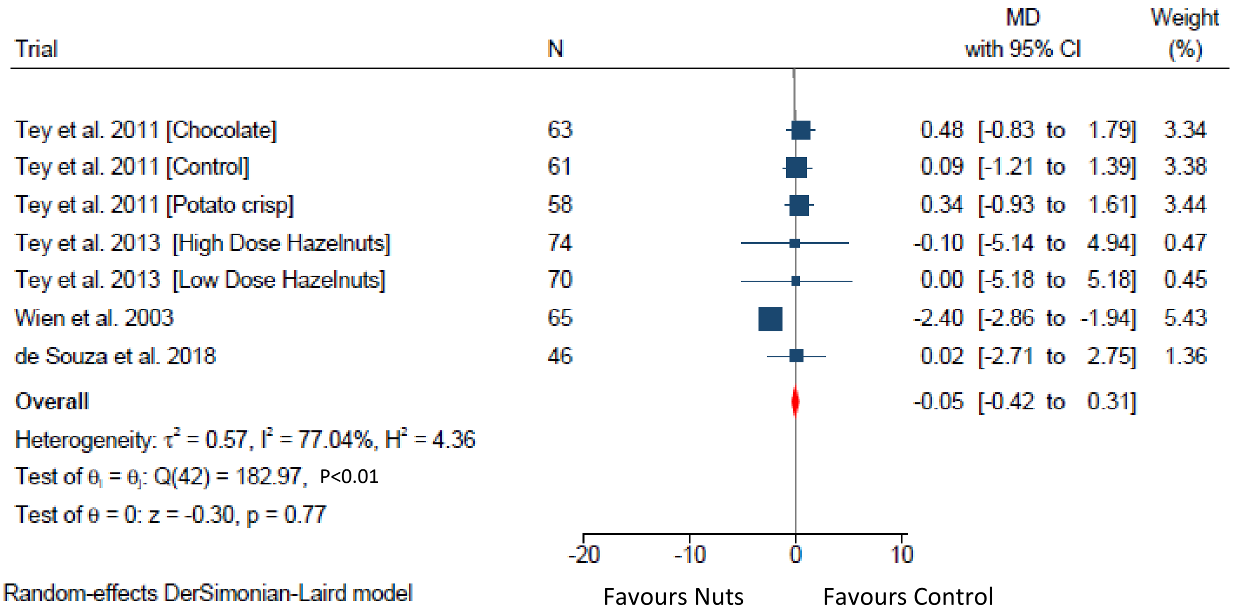

Pooled effect estimate is represented by the diamond and was estimated with the use of a random-effects DerSimonian-Laird model.
To avoid unit of analysis error, standard error, used for determining the 95% confidence interval, was calculated by splitting the N for studies with multiple comparisons as per the Cochrane Handbook, 2019.
CI, confidence interval; MD, mean difference; N, number of participants.

Supplementary Figure 10. Forest plot of randomized controlled trials investigating the effects of nut consumption on waist circumference (cm) (continued on next page).

Trial	N	MD with 95% CI	Weight (%)
Abazarfard et al. 2014	100	-8.72 [-11.15 to -6.29]	0.23
Abbaspour et al. 2019	48	-0.30 [-1.46 to 0.86]	0.98
Balci et al. 2012	60	0.10 [-5.83 to 6.03]	0.04
Bamberger et al. 2017	204	-0.10 [-1.69 to 1.49]	0.53
Barbour et al. 2015	61	0.30 [-2.55 to 3.15]	0.17
Berryman et al. 2015	48	-0.80 [-3.15 to 1.55]	0.25
Bitok et al. 2018	317	0.30 [-2.76 to 3.36]	0.15
Biude Silva Duarte et al. 2019	55	-3.60 [-5.75 to -1.45]	0.29
Bowen et al. 2019	76	-0.47 [-1.62 to 0.68]	1.00
Campbell et al. 2019	12	-7.20 [-19.13 to 4.73]	0.01
Carughi et al. 2019	60	-0.50 [-3.58 to 2.58]	0.14
Casas-Agustench et al. 2011	50	-0.40 [-2.99 to 2.19]	0.20
Ciccone et al. 2014 [Control]	31	-4.10 [-18.69 to 10.49]	0.01
Ciccone et al. 2014 [Non-fried fish]	15	-1.40 [-17.38 to 14.58]	0.01
Ciccone et al. 2014 [Olive Oil]	15	-5.80 [-24.75 to 13.15]	0.00
Damavandi et al. 2012	43	-0.80 [-6.90 to 5.30]	0.04
DeSouza et al. 2018	46	-2.45 [-8.93 to 4.03]	0.03
Dhillon et al. 2016	86	-1.79 [-6.71 to 3.13]	0.06
Dhillon et al. 2018	73	0.20 [0.15 to 0.25]	24.53
Gulati et al. 2014	68	1.80 [-1.66 to 5.26]	0.11
Hernández-Alonso et al. 2014	49	1.07 [0.29 to 1.85]	2.06
Hwang et al. 2019	84	1.66 [-0.24 to 3.56]	0.37
Jenkins et al. 2002 [Full Dose Almonds]	27	-1.30 [-7.75 to 5.15]	0.03
Jenkins et al. 2002 [Half Dose Almonds]	27	-2.00 [-8.45 to 4.45]	0.03
Jenkins et al. 2018 [Full Dose Nuts]	100	0.00 [-0.02 to 0.02]	25.21
Jenkins et al. 2018 [Half Dose Nuts]	100	0.00 [-0.02 to 0.02]	25.21
Johnston et al. 2013	44	-2.60 [-5.51 to 0.31]	0.16
Jung et al. 2018	84	0.10 [-1.16 to 1.36]	0.83
Katz et al. 2012	46	-0.20 [-1.56 to 1.16]	0.72
Lee et al. 2014	60	-0.62 [-1.27 to 0.03]	2.85
Liu et al. 2018	85	-0.86 [-2.66 to 0.94]	0.42
Ma et al. 2010	22	-0.30 [-2.55 to 1.95]	0.27
Mohan et al. 2018	269	-0.12 [-0.70 to 0.46]	3.46
Moreira Alves et al. 2014 [Conventional Peanut]	44	0.06 [-1.08 to 1.20]	1.01
Moreira Alves et al. 2014 [High Oleic Peanut]	43	0.48 [-0.59 to 1.55]	1.15

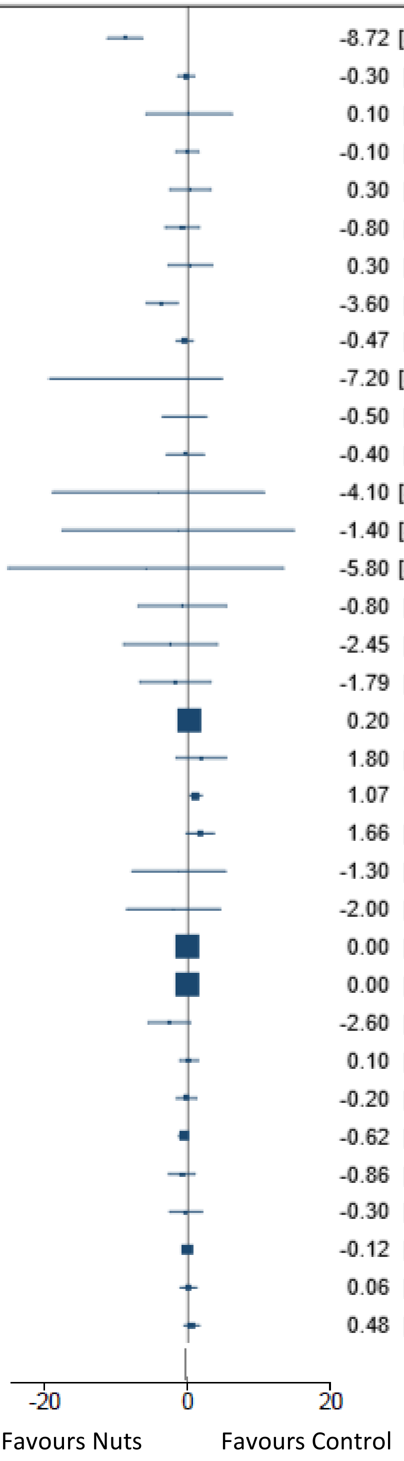

Supplementary Figure 10. Forest plot of randomized controlled trials investigating the effects of nut consumption on waist circumference (cm).

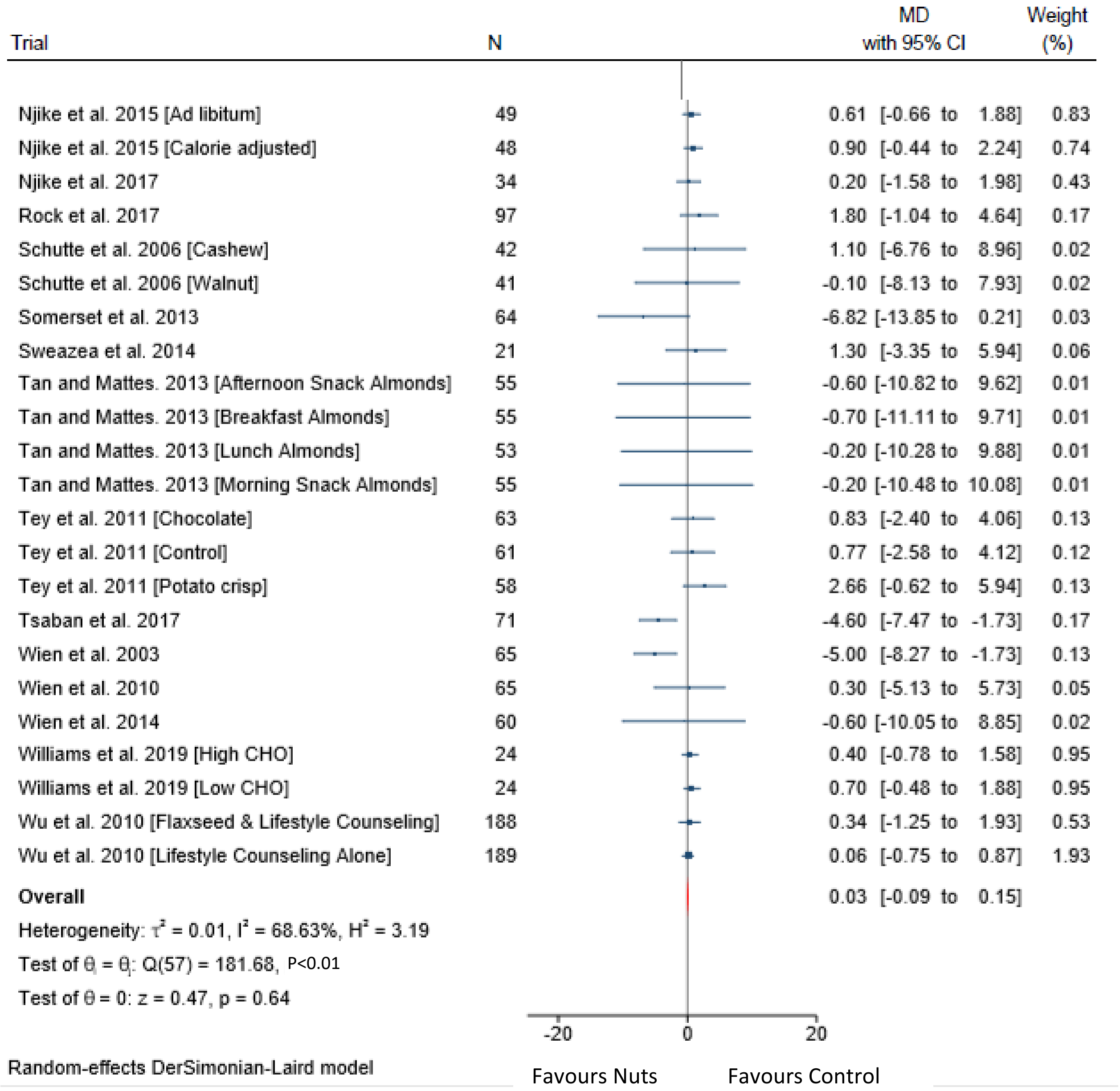

Pooled effect estimate is represented by the diamond and was estimated with the use of a random-effects DerSimonian-Laird model.

To avoid unit of analysis error, standard error, used for determining the 95% confidence interval, was calculated by splitting the N for studies with multiple comparisons as per the Cochrane Handbook, 2019.

CI, confidence interval; MD, mean difference; N, number of participants.

Supplementary Figure 11. Forest plot of randomized controlled trials investigating the effects of nut consumption on waist-to-hip ratio.

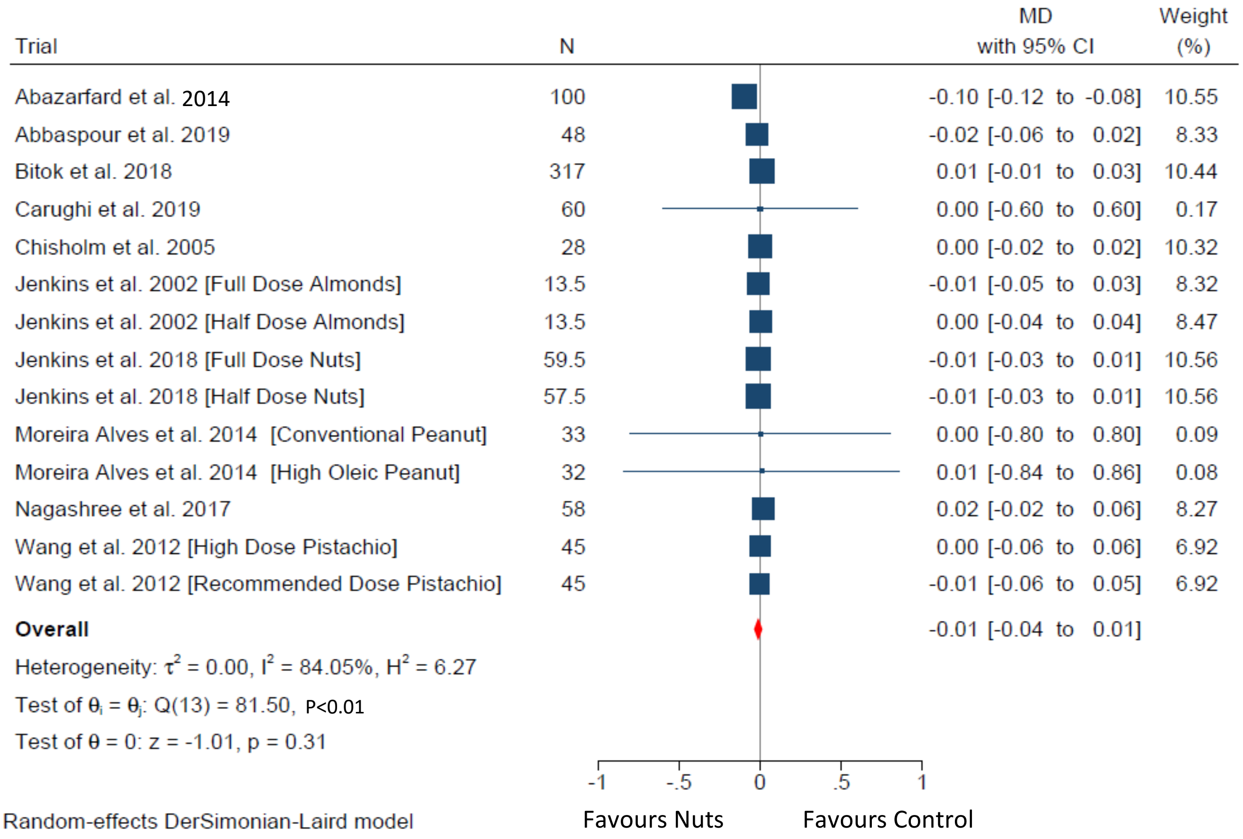

Pooled effect estimate is represented by the diamond and was estimated with the use of a random-effects DerSimonian-Laird model.
To avoid unit of analysis error, standard error, used for determining the 95% confidence interval, was calculated by splitting the N for studies with multiple comparisons as per the Cochrane Handbook, 2019.
CI, confidence interval; MD, mean difference; N, number of participants.

Supplementary Figure 12. Forest plot of randomized controlled trials investigating the effects of nut consumption on visceral adipose tissue.

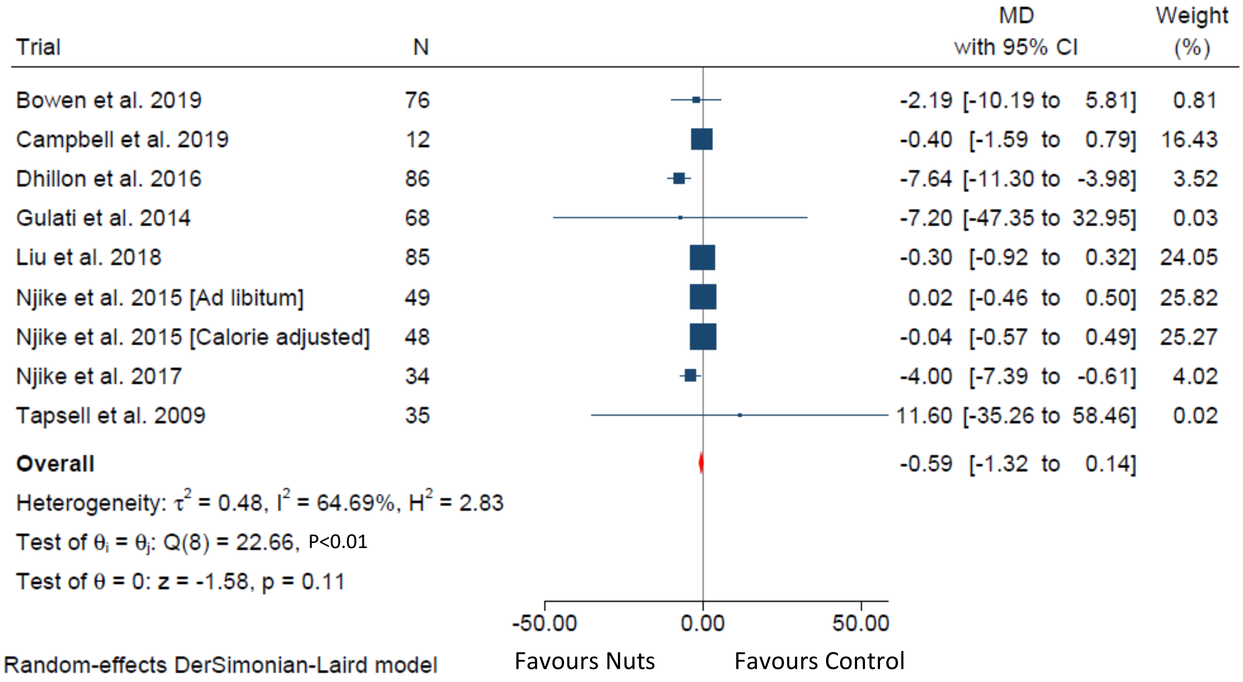

Pooled effect estimate is represented by the diamond and was estimated with the use of a random-effects DerSimonian-Laird model.

To avoid unit of analysis error, standard error, used for determining the 95% confidence interval, was calculated by splitting the N for studies with multiple comparisons as per the Cochrane Handbook, 2019.

CI, confidence interval; MD, mean difference; N, number of participants.

Supplementary Figure 13. Linear and non-linear meta-regression analyses for the effect of nut consumption on measures of adiposity from prospective cohorts.

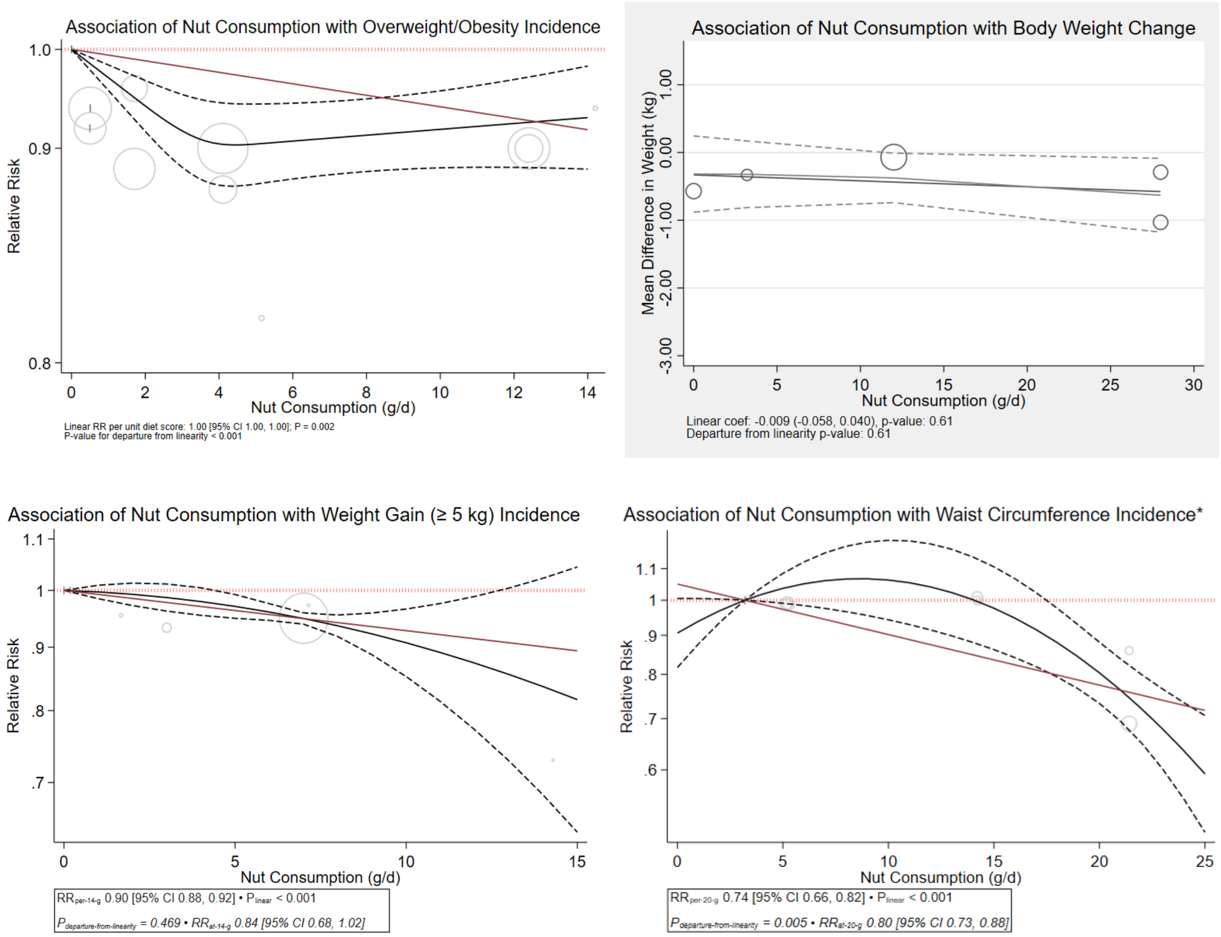

*Waist circumference incidence refers to the incidence of waist circumference increasing ≥94 cm for men or ≥ for women. Individual cohorts are represented by the circles with their weight in the overall analysis represented by the size of the circles. The straight solid line represents the linear estimate dose-response and the solid curved line represents the non-linear dose response for nut consumption (g/d) dotted lines represent the upper and lower 95% confidence intervals for the non-linear estimates.

Supplementary Figure 14. Linear and non-linear meta-regression analyses for the effect of nut consumption on measures of adiposity from randomized controlled trials.

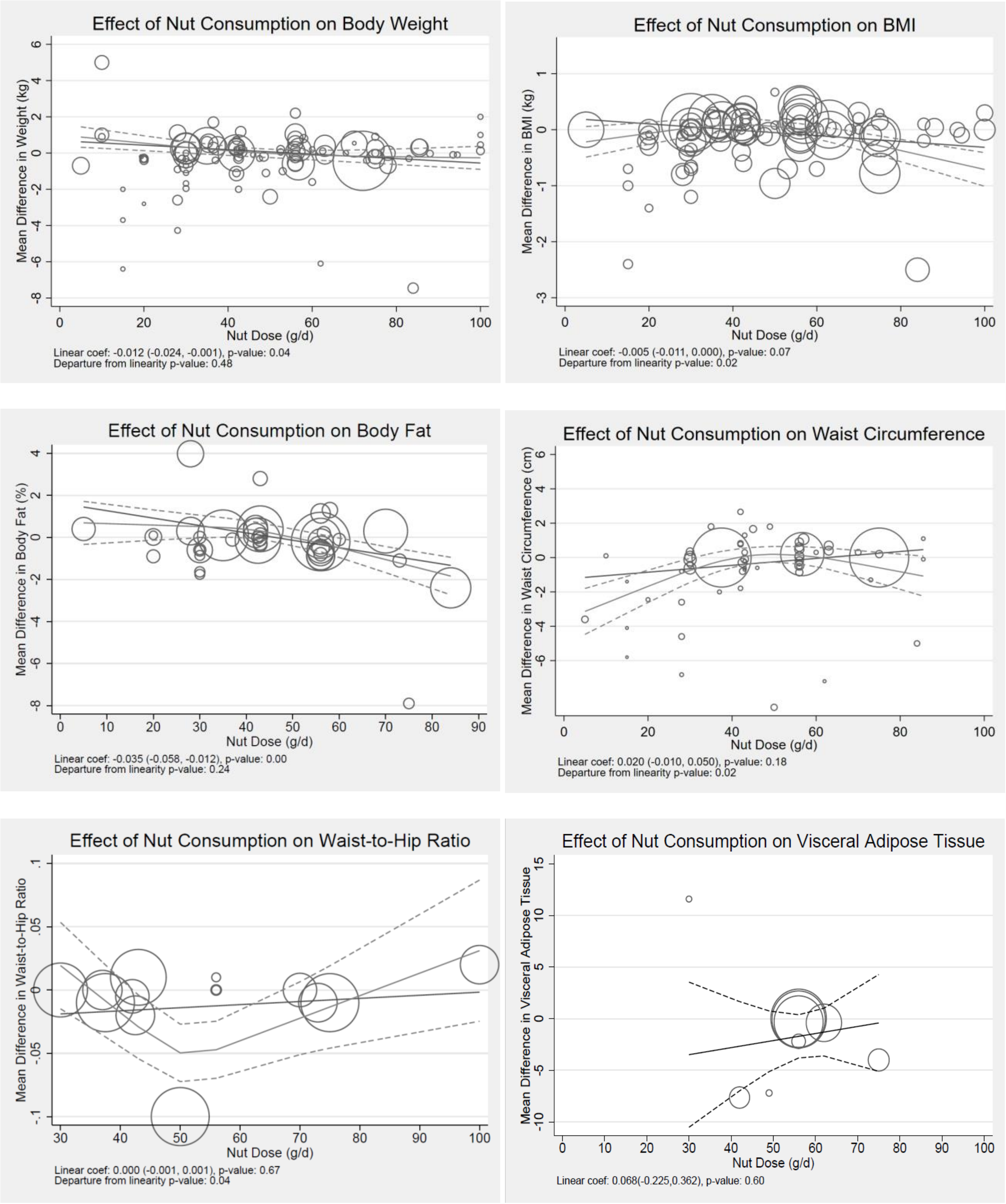

Individual trials are represented by the circles with their weight in the overall analysis represented by the size of the circles. The straight solid line represents the linear estimate dose-response and the solid curved line represents the non-linear dose response for nut consumption (g/d) dotted lines represent the upper and lower 95% confidence intervals for the non-linear estimates, except for visceral adipose tissue where they represent the upper and lower 95% confidence intervals for the linear estimate dose-response.

Supplementary Figure 15. *A priori* subgroup analysis for mean differences (95% CIs) of the effects of nut consumption in on body weight (kg) (continued on the next page).

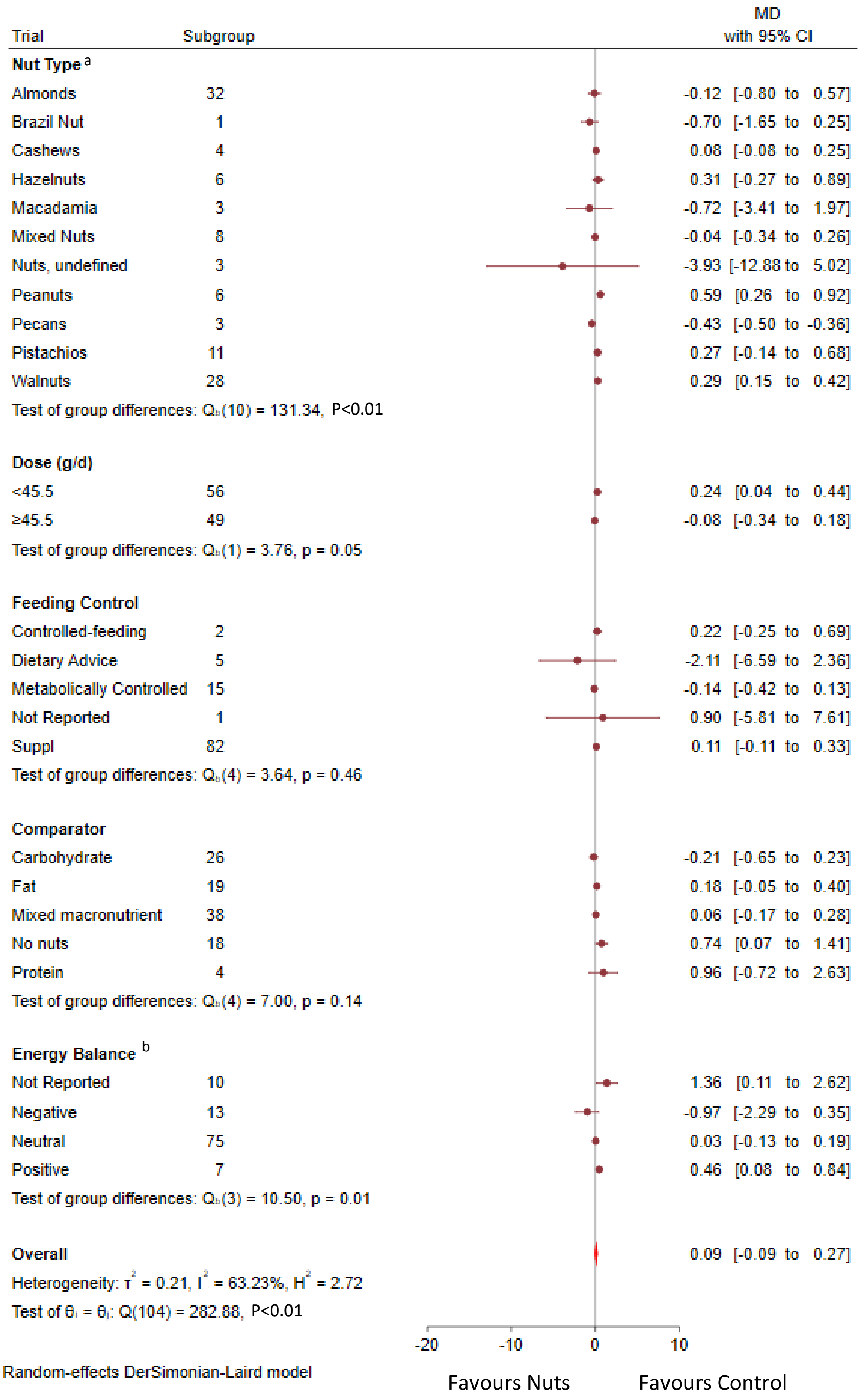

Supplementary Figure 15. *A priori* subgroup analysis for mean differences (95% CIs) of the effects of nut consumption in on body weight (kg) (continued on the next page).

Trial	Subgroup	MD with 95% CI
**Design**		
Crossover	34	0.12 [-0.15 to 0.39]
Parallel	71	0.06 [-0.22 to 0.33]
Test of group differences: $Q_b(1) = 0.10$, p = 0.75		
**Duration (weeks)**		
≤8	56	-0.00 [-0.18 to 0.18]
>8	49	0.09 [-0.21 to 0.39]
Test of group differences: $Q_b(1) = 0.24$, p = 0.62		
**Health Status**		
Coronary Artery Disease	2	3.00 [-0.92 to 6.92]
Dyslipidemia	13	-0.01 [-0.50 to 0.49]
Healthy	25	-0.02 [-0.31 to 0.27]
Metabolic Syndrome	10	0.21 [-0.05 to 0.47]
Multiple	2	-2.03 [-4.74 to 0.69]
Overweight/Obese	34	-0.18 [-0.61 to 0.25]
Prediabetes	6	0.18 [-0.31 to 0.67]
Type 2 Diabetes	13	0.20 [-0.08 to 0.47]
Test of group differences: $Q_b(7) = 8.17$, p = 0.32		
**Intended Weight Maintenance**		
No	67	0.10 [-0.20 to 0.39]
Not Reported	7	-0.64 [-1.52 to 0.25]
Yes	31	0.10 [-0.17 to 0.37]
Test of group differences: $Q_b(2) = 2.52$, p = 0.28		
**Funding**		
Agency	20	0.16 [-0.60 to 0.92]
Agency-Industry	32	0.07 [-0.17 to 0.31]
Industry	42	0.07 [-0.19 to 0.34]
None reported	11	0.36 [0.17 to 0.55]
Test of group differences: $Q_b(3) = 4.81$, p = 0.19		
**Overall**		0.09 [-0.09 to 0.27]
Heterogeneity: $\tau^2 = 0.21$, $I^2 = 63.23\%$, $H^2 = 2.72$		
Test of $\theta_i = \theta_j$: $Q(104) = 282.88$, P<0.01		

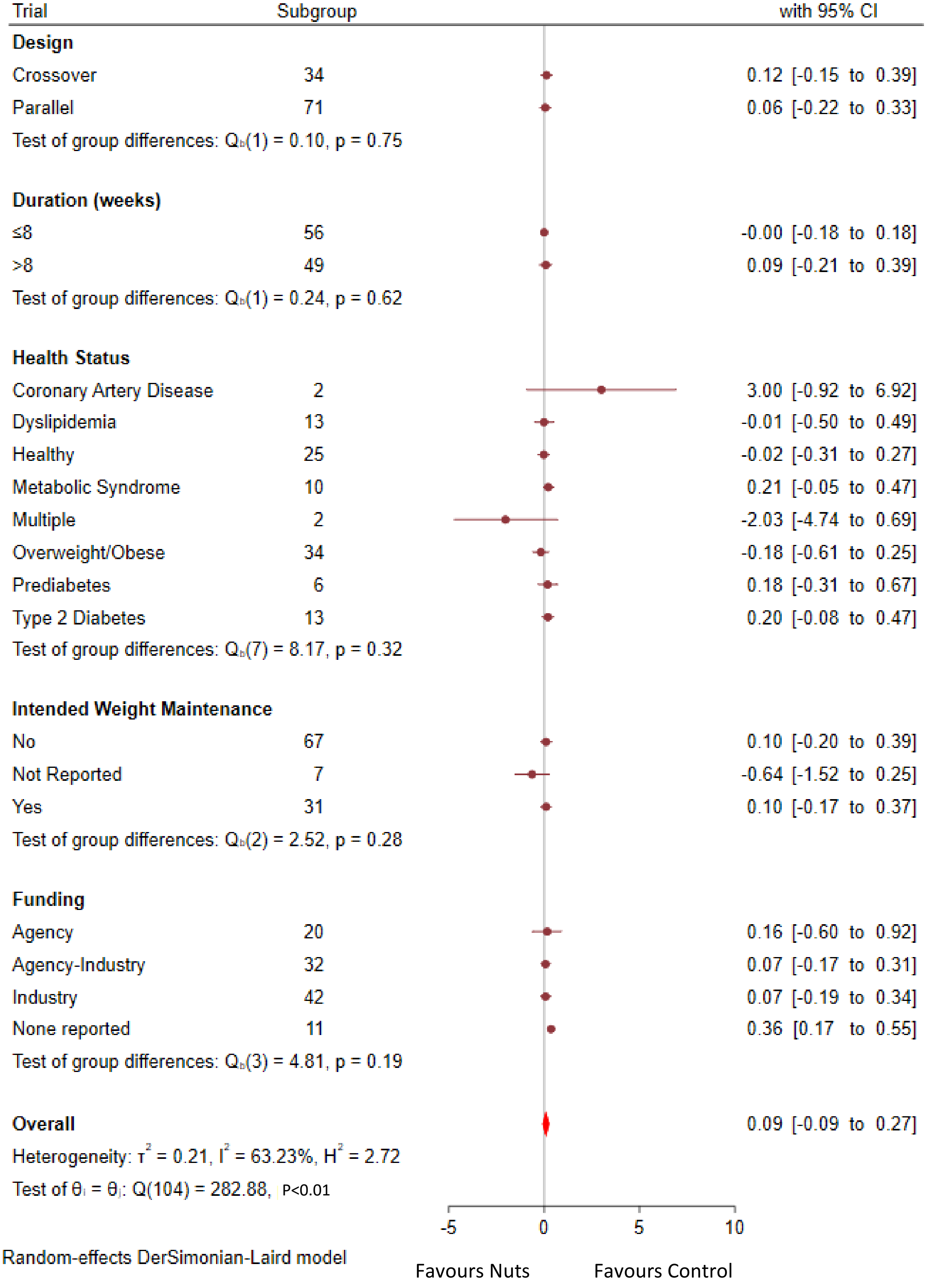

Random-effects DerSimonian-Laird model

Supplementary Figure 15. *A priori* subgroup analysis for mean differences (95% CIs) of the effects of nut consumption in on body weight (kg) (continued on next page).

Pooled effect estimates for each subgroup and overall effect are represented by the diamonds. Data are expressed as weighted mean differences with 95% CIs using the random-effects DerSimonian-Laird model. Paired analyses were applied to all crossover trials. Inter-trial heterogeneity was assessed using the Cochran Q statistic and quantified using the I^2 statistic, with significance set at P<0.10 and I^2>50% considered to be evidence of substantial heterogeneity.
CI, confidence interval; DA, dietary advice; Feeding-control is the provision of some meals and foods consumed during the trial; MC, metabolically controlled: is the provision of all meals and foods consumed during the trial under controlled conditions; MD, mean difference; N, no; NR, not reported; SE, standard error; Suppl, supplemented: is the provision of the intervention and control foods during the trial; Y, yes. Negative energy balance refers to a deficit in normal energy intake and/or intake below energy requirements. Neutral energy balance refers to the maintenance of usual energy intake and/or meeting energy requirements. Position energy balance refers to an excess in normal energy intake and/or intake above energy requirements. Weight maintenance intended refers to the trial being designed to maintain participants' body weight during the course of the trial.
Agency funding is that from government, university, or not-for-profit sources. Industry funding is that from trade organizations that obtain revenue from the sale of products.
[a]Pairwise between-subgroup mean differences (95% CIs) for Nut Type were as follows: -0.60 kg (-1.90, 0.69 kg) (Brazil nut vs. Almonds) to 0.27 kg (-0.38, 0.93 kg) (Cashews vs. Almonds) to 0.40 kg (-0.42, 1.23 kg) (Hazelnuts vs. Almonds) to -0.63 kg (-3.40, 2.13 kg) (Macadamia vs. Almonds) to 0.03 kg (-0.57, 0.62 kg) (Mixed nuts vs. Almonds) to -3.83 kg (-12.8, 5.14 kg) (Undefined nuts vs. Almonds) to 0.77 kg (0.09 to 1.46 kg) (Peanuts vs. Almonds) to -0.32 kg (-1.19, 0.55 kg) (Pecans vs. Almonds) to 0.37 kg (-0.40, 1.14 kg) (Pistachios vs. Almond) to 0.28 kg (-0.21, 0.77 kg) (Walnuts vs. Almonds) to 0.88 kg (-0.48, 2.23 kg) (Cashews vs. Brazil nuts) to 1.00 kg (-0.44, 2.45 kg) (Hazelnuts vs. Brazil nuts) to -0.32 kg (-3.04, 2.98 kg) (Macadamia vs. Brazil nuts) to 0.63 kg (-0.70, 1.95 kg) (Mixed nuts vs. Brazil nuts) to -3.23 kg (-12.30, 5.82 kg) (Undefined nuts vs. Brazil nuts) to 1.38 kg (0.01, 2.74 kg) (Peanuts vs. Brazil nuts) to 0.28 kg (-1.19, 1.75 kg) (Pecans vs. Brazil nuts) to 0.97 kg (-0.44, 2.38 kg) (Pistachios vs. Brazil nuts) to 0.88 kg (-0.40, 2.17 kg) (Walnuts vs. Brazil nuts) to 0.13 kg (-0.76, 1.04 kg) (Hazelnuts vs. Cashews) to -0.91 kg (-3.70, 1.89 kg) (Macadamia vs. Cashews) to -0.25 kg (-0.96, 0.47 kg) (Mixed nuts vs. Cashews) to -4.10 kg (-13.1, 4.88 kg) (Undefined nuts vs. Cashews) to 0.50 kg (-0.29,1.29 kg) (Peanuts vs. Cashews) to -0.60 kg (-1.56, 0.36 kg) (Pecans vs. Cashews) to 0.94 kg (-0.77, 0.96 kg) (Pistachios vs. Cashews) to 0.004 kg (-0.63, 0.64 kg) (Walnuts vs. Cashews) to -1.04 kg (-3.87, 1.80 kg) (Macadamia vs. Hazelnuts) to -0.38 kg (-1.25, 0.49 kg) (Mixed nuts vs. Hazelnuts) to -4.23 kg (-13.2, 4.76 kg) (Undefined nuts vs. Hazelnuts) to 0.37 kg (-0.57, 1.31 kg) (Peanuts vs. Hazelnuts) to -0.73 kg (-1.81, 0.35 kg) (Pecans vs. Hazelnuts) to -0.04 kg (-1.04, 0.96 kg) (Pistachios vs. Hazelnuts) to -0.13 kg (-0.93, 0.68 kg) (Walnuts vs. Hazelnuts) to 0.66 kg (-2.12, 3.44 kg) (Mixed nuts vs. Macadamia) to -3.20 kg (-12.6, 6.18 kg) (Undefined nuts vs. Macadamia) to 1.41 kg (-1.39, 4.21 kg) (Peanuts vs. Macadamia) to 0.31 kg (-2.54, 3.16 kg) (Pecans vs. Macadamia) to 1.00 kg (-1.82, 3.82 kg) (Pistachios vs. Macadamia) to 0.91 kg (-1.82, 3.67 kg) (Walnuts vs. Macadamia) to -3.85 kg (-12.8, 5.12 kg) (Undefined nuts vs. Mixed nuts) to 0.75 kg (-0.004, 1.49 kg) (Peanuts vs. Mixed nuts) to -0.35 kg (-1.27, 0.57 kg) (Pecans vs. Mixed nuts) to 0.34 kg (-0.48, 1.16 kg) (Pistachios vs. Mixed nuts) to 0.25 kg (-0.32, 0.82 kg) (Walnuts vs. Mixed nuts) to 4.60 kg (-4.38, 13.6 kg) (Peanuts vs. Undefined nuts) to 3.50 kg (-5.49, 12.50 kg) (Pecans vs. Undefined nuts) to 4.20 kg (-4.79, 13.20 kg) (Pistachios vs. Undefined nuts) to 4.11 kg (-4.86, 13.10 kg) (Walnuts vs. Undefined nuts) to -1.10 kg (-2.08, -0.12 kg) (Pecans vs. Peanuts) to -0.41 kg (-1.30, 0.49 kg) (Pistachios vs. Peanuts) to -0.49 kg (-1.16, 0.17 kg) (Walnuts vs. Peanuts) to 0.69 kg (-0.35, 1.73 kg)

Supplementary Figure 15. *A priori* subgroup analysis for mean differences (95% CIs) of the effects of nut consumption in on body weight (kg).

(Pistachios vs. Pecans) to 0.60 kg (-0.25, 1.46 kg) (Walnuts vs. Pecans) to -0.09 kg (-0.84, 0.66 kg) (Walnuts vs. Pistachios).

[b]Pairwise between subgroup mean differences (95% CIs) for Energy balance were as follows: -1.92 kg (-2.77,-1.07 kg) (Negative vs. NR) to -1.17 kg (-1.79, -0.56 kg) (Neutral vs. NR) to -0.87 kg (-1.84, 0.11 kg) (Positive vs. NR) to 0.75 kg (0.10, 1.39 kg) (Neutral vs. Negative) to 1.05 kg (-0.06, 2.05 kg) (Positive vs. Negative) to 0.31 kg (-0.50, 1.11 kg) (Positive vs. Neutral).

Supplementary Figure 16. *A priori* subgroup analysis for mean differences (95% CIs) of the effects of nut consumption in on BMI (kg/m^2) (continued on the next page).

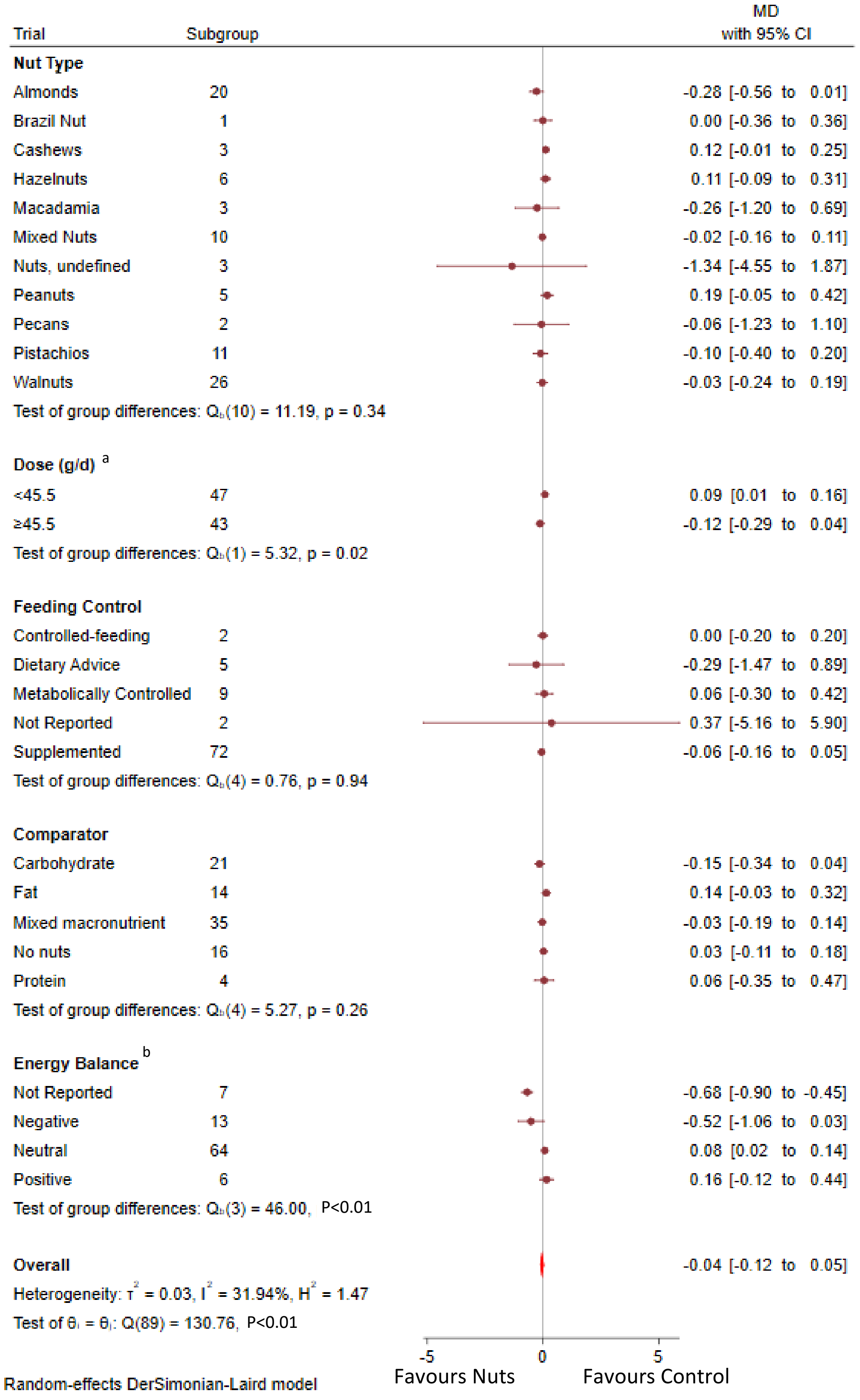

Supplementary Figure 16. *A priori* subgroup analysis for mean differences (95% CIs) of the effects of nut consumption in on BMI (kg/m^2) (continued on the next page).

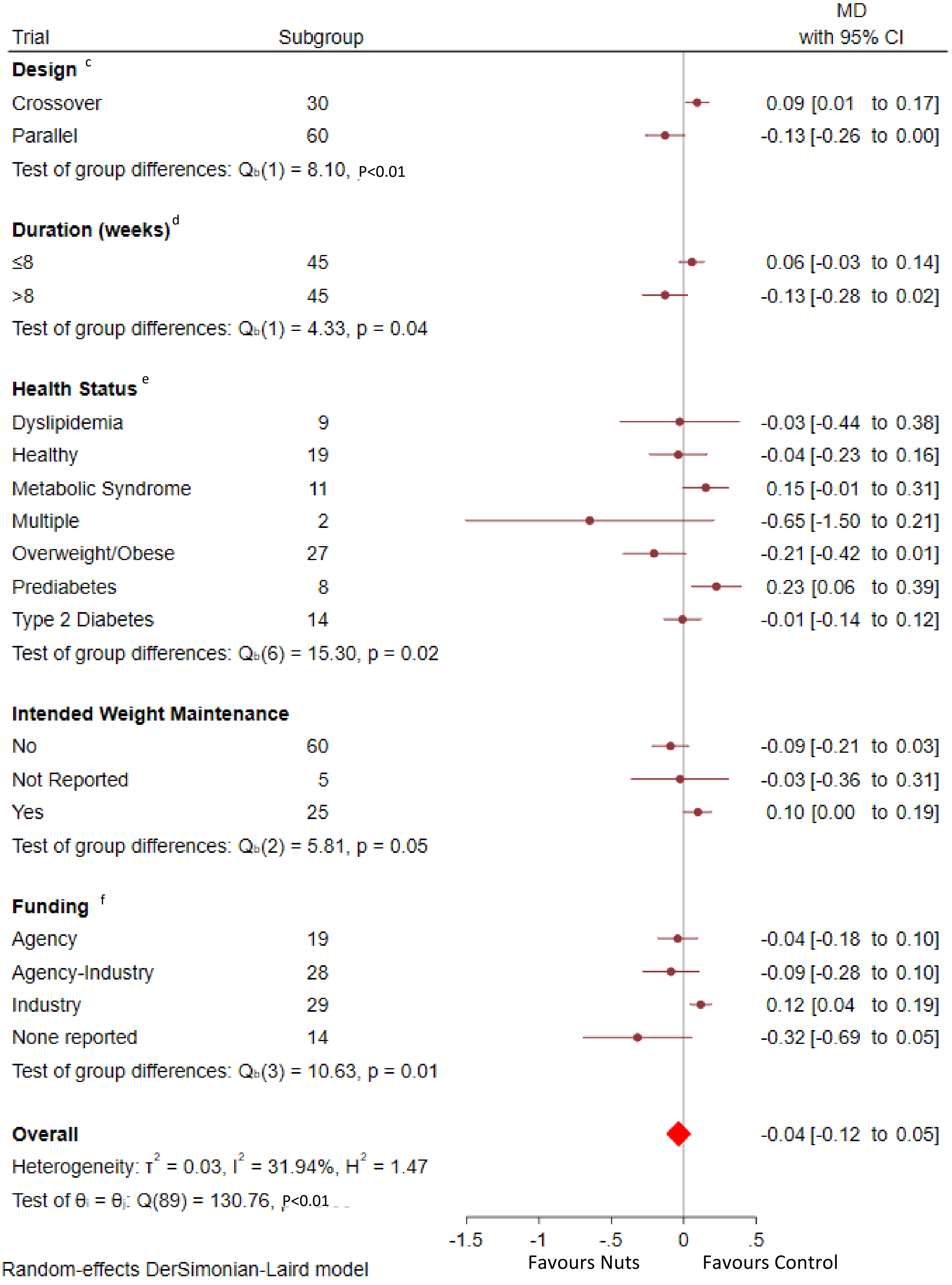

Supplementary Figure 16. *A priori* subgroup analysis for mean differences (95% CIs) of the effects of nut consumption in on BMI (kg/m^2).

Pooled effect estimates for each subgroup and overall effect are represented by the diamonds. Data are expressed as weighted mean differences with 95% CIs using the random-effects DerSimonian-Laird model. Paired analyses were applied to all crossover trials. Inter-trial heterogeneity was assessed using the Cochran Q statistic and quantified using the I^2 statistic, with significance set at P<0.10 and I^2>50% considered to be evidence of substantial heterogeneity.

CI, confidence interval; DA, dietary advice; Feeding-control is the provision of some meals and foods consumed during the trial; MC, metabolically controlled: is the provision of all meals and foods consumed during the trial under controlled conditions; MD, mean difference; N, no; NR, not reported; SE, standard error; Suppl, supplemented: is the provision of the intervention and control foods during the trial; Y, yes. Negative energy balance refers to a deficit in normal energy intake and/or intake below energy requirements. Neutral energy balance refers to the maintenance of usual energy intake and/or meeting energy requirements. Position energy balance refers to an excess in normal energy intake and/or intake above energy requirements. Weight maintenance intended refers to the trial being designed to maintain participants' body weight during the course of the trial.

Agency funding is that from government, university, or not-for-profit sources. Industry funding is that from trade organizations that obtain revenue from the sale of products.

[a]Pairwise between-subgroup mean differences (95% CIs) for Dose were as follows: -0.16 kg/m^2 (-0.31, 0.03 kg/m^2) (1 vs. 2).

[b]Pairwise between-subgroup mean differences (95% CIs) for Energy balance were as follows: 0.44 kg/m^2 (0.14, 0.74 kg/m^2) (Negative vs. Not Reported) to 0.76 kg/m^2 (0.53, 0.99 kg/m^2) (Neutral vs. Not Reported) to 0.84 kg/m^2 (0.48, 1.19 kg/m^2) (Positive vs. Not Reported) to 0.32 kg/m^2 (0.11, 0.53 kg/m^2) (Neutral vs. Negative) to 0.40 kg/m^2 (0.05, 0.74 kg/m^2) (Positive vs. Negative) to 0.08 kg/m^2 (-0.21, 0.36 kg/m^2) (Neutral vs. Positive).

[c]Pairwise between-subgroup mean differences (95% CIs) for Design were as follows: -0.17 kg/m^2 (-0.34, -0.004) (Parallel vs. Crossover).

[d]Pairwise between-subgroup mean differences (95% CIs) for Duration were as follows: -0.13 kg/m^2 (-0.31, 0.04 kg/m^2) (≥8 vs. <8).

[e]Pairwise between-subgroup mean differences (95% CIs) for Health status were as follows: -0.01 kg/m^2 (-0.47, 0.45 kg/m^2) (Healthy vs. Dyslipidemia) to -0.14 kg/m^2 (-0.37, 0.63 kg/m^2) (Metabolic Syndrome vs. Dyslipidemia) to -0.61 kg/m^2 (-1.61, 0.39 kg/m^2) (Multiple vs. Dyslipidemia) to -0.12 kg/m^2 (-0.58, 0.34 kg/m^2) (Overweight/Obese vs. Dyslipidemia) to -0.28 kg/m^2 (-0.25, 0.80 kg/m^2) (Prediabetes vs. Dyslipidemia) to -0.03 kg/m^2 (-0.51, 0.44 kg/m^2) (Type 2 diabetes vs. Dyslipidemia) to 0.15 kg/m^2 (-0.16, 0.45 kg/m^2) (Metabolic Syndrome vs. Healthy) to -0.60 kg/m^2 (-1.52 vs. 0.33 kg/m^2) (Multiple vs. Healthy) to -0.11 kg/m^2 (-0.34, 0.12 kg/m^2) (Overweight/Obese vs. Healthy) to 0.29 kg/m^2 (-0.05, 0.63 kg/m^2) (Prediabetes vs. Healthy) to -0.02 kg/m^2 (-0.28, 0.24 kg/m^2) (Type 2 Diabetes vs. Healthy) to -0.75 kg/m^2 (-1.69, 0.20 kg/m^2) (Multiple vs. Metabolic Syndrome) to -0.26 kg/m^2 (-0.56, 0.05 kg/m^2) (Overweight/Obese vs. Metabolic Syndrome) to 0.14 kg/m^2 (-0.25, 0.53 kg/m^2) (Prediabetes vs. Metabolic Syndrome) to -0.17 kg/m^2 (-0.49, 0.16 kg/m^2) (Type 2 Diabetes vs. Metabolic Syndrome) to 0.49 kg/m^2 (-0.43, 1.41 kg/m^2) (Overweight/Obese vs. Multiple) to 0.89 kg/m^2 (-0.07, 1.84 kg/m^2) (Prediabetes vs. Multiple) to 0.58 kg/m^2 (-0.35, 1.50 kg/m^2) (Type 2 Diabetes vs. Multiple) to 0.40 kg/m^2 (0.06, 0.73 kg/m^2) (Prediabetes vs. Overweight/Obese) to 0.09 kg/m^2 (-0.17, 0.34 kg/m^2) (Type 2 Diabetes vs. Overweight/Obese) to -0.31 kg/m^2 (-0.67, 0.05 kg/m^2) (Type 2 Diabetes vs. Prediabetes).

[f]Pairwise between-subgroup mean differences (95% CIs) for Funding were as follows: 0.01 kg/m^2 (-0.23, 0.25 kg/m^2) (AI vs. A) to 0.19 kg/m^2 (-0.03, 0.41 kg/m^2) (I vs. A) to -0.22 kg/m^2 (-0.49, 0.05 kg/m^2) (NR vs. A)

to 0.18 kg/m^2 (-0.02, 0.38 kg/m^2) (I vs. AI) to -0.23 kg/m^2 (-0.49, 0.03 kg/m^2) (NR vs. AI) to -0.41 kg/m^2 (-0.65, -0.16 kg/m^2) (NR vs. I) where A=agency, AI=agency-industry, I=industry, NR=none reported.

Supplementary Figure 17. *A priori* subgroup analysis for mean differences (95% CIs) of the effects of nut consumption on body fat (%) (continued on the next page).

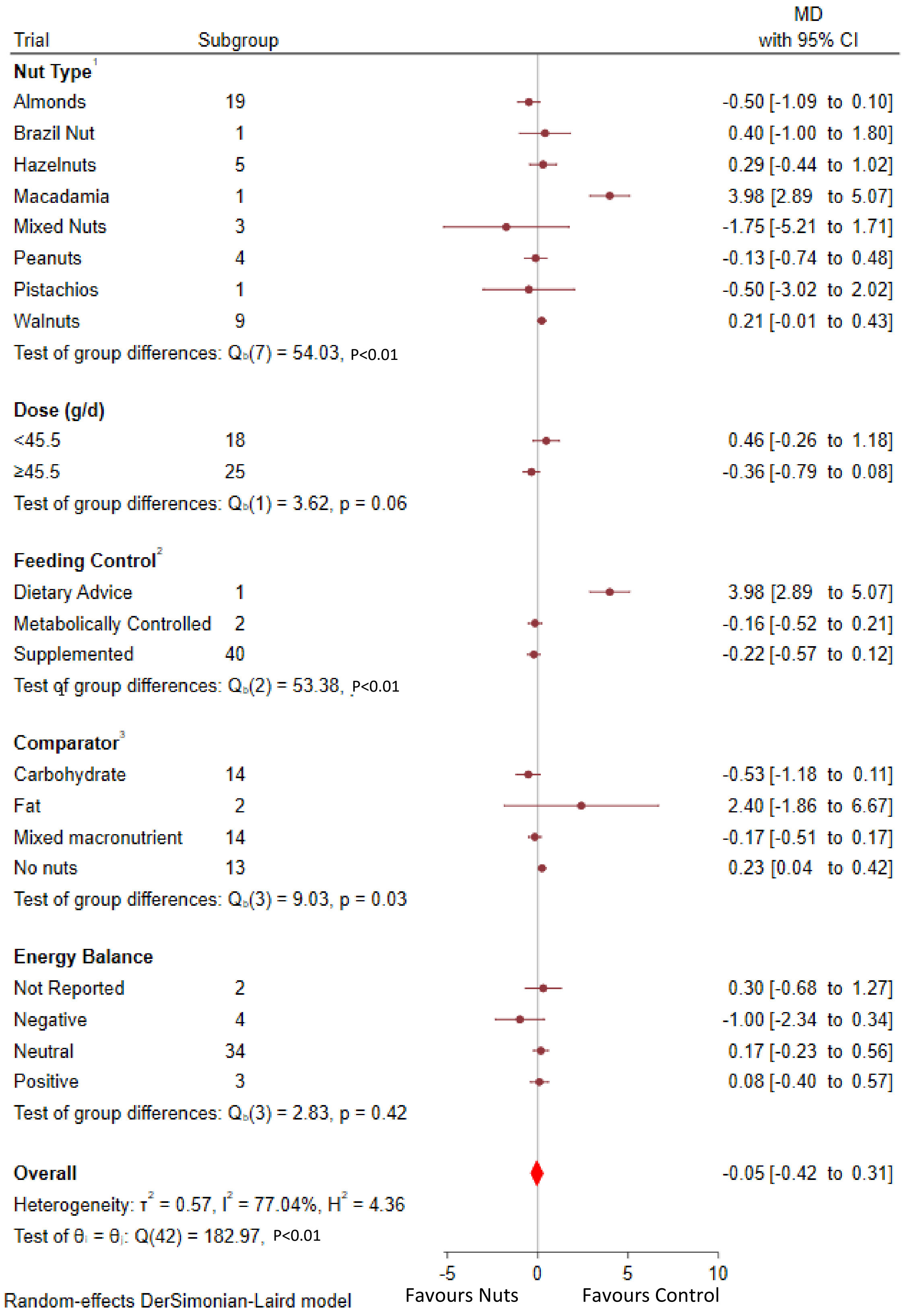

Supplementary Figure 17. *A priori* subgroup analysis for mean differences (95% CIs) of the effects of nut consumption on body fat (%) (continued on the next page).

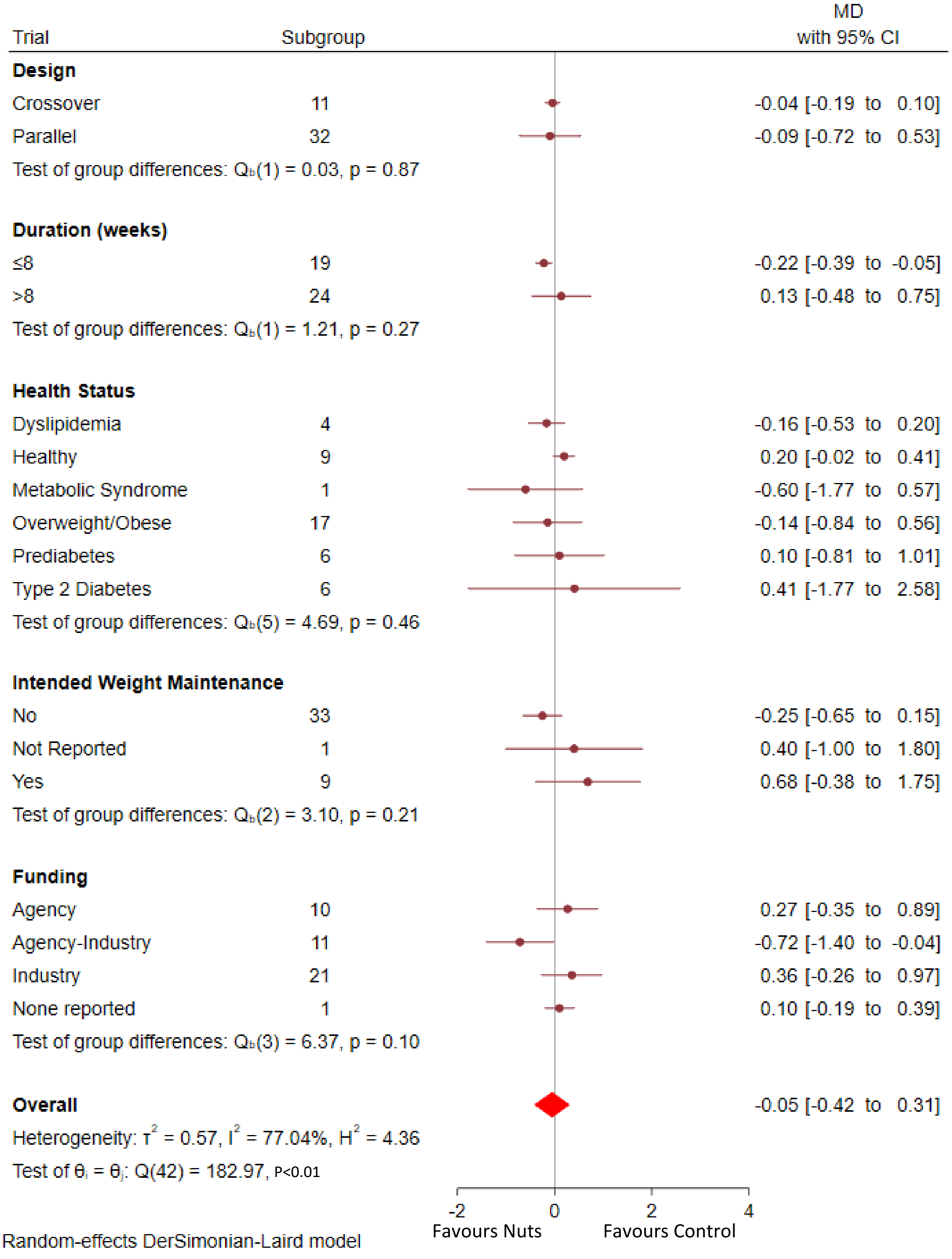

Supplementary Figure 17. *A priori* subgroup analysis for mean differences (95% CIs) of the effects of nut consumption on body fat (%).
Pooled effect estimates for each subgroup and overall effect are represented by the diamonds. Data are expressed as weighted mean differences with 95% CIs using the random-effects DerSimonian-laird model. Paired analyses were applied to all crossover trials. Inter-trial heterogeneity was assessed using the Cochran Q statistic and quantified using the I^2 statistic, with significance set at P<0.10 and I^2>50% considered to be evidence of substantial heterogeneity.

CI, confidence interval; DA, dietary advice; Feeding-control is the provision of some meals and foods consumed during the trial; MC, metabolically controlled: is the provision of all meals and foods consumed during the trial under controlled conditions; MD, mean difference; N, no; NR, not reported; SE, standard error; Suppl, supplemented: is the provision of the intervention and control foods during the trial; Y, yes. Negative energy balance refers to a deficit in normal energy intake and/or intake below energy requirements. Neutral energy balance refers to the maintenance of usual energy intake and/or meeting energy requirements. Position energy balance refers to an excess in normal energy intake and/or intake above energy requirements. Weight maintenance intended refers to the trial being designed to maintain participants' body weight during the course of the trial.
Agency funding is that from government, university, or not-for-profit sources. Industry funding is that from trade organizations that obtain revenue from the sale of products.
[1]Pairwise between-subgroup mean differences (95% CIs) for Nut type were as follows: 0.97% (-0.97, 2.86%) (Brazil nuts vs. Almonds) to 0.82% (-0.28, 1.93%) (Hazelnuts vs. Almonds) to 4.53% (2.82, 6.23%) (Macadamia vs. Almonds) to -0.37% (-2.00 vs. 1.27%) (Mixed nuts vs. Almonds) to 0.39% (-0.50 vs. 1.28%) (Peanuts vs. Almonds) to 0.05% (-2.79, 2.88%) (Pistachios vs. Almonds) to 0.74% (-0.14, 1.62%) (Walnuts vs. Almonds) to -0.12% (-2.22, 1.97%) (Hazelnuts vs. Brazil nuts) to 3.58% (1.11, 6.04%) (Macadamia vs. Brazil nuts) to -1.31% (-3.73, 1.10%) (Mixed nuts vs. Brazil nuts) to -0.56% (-2.55, 1.43%) (Peanuts vs. Brazil nuts) to -0.90% (-4.25, 2.45%) (Pistachios vs. Brazil nuts) to -0.21% (-2.20, 1.78%) (Walnuts vs. Brazil nuts) to 3.70% (1.80, 5.60%) (Macadamia vs. Hazelnuts) to -1.19 % (-3.03, 0.65%) (Mixed nuts vs. Hazelnuts) to -0.44% (-1.67, 0.79%) (Peanuts vs. Hazelnuts) to -0.78% (-3.74, 2.19%) (Pistachios vs. Hazelnuts) to -0.09% (-1.31, 1.14%) (Walnuts vs. Hazelnuts) to -4.89% (-7.14, -2.64%) (Mixed nuts vs. Macadamia) to -4.14% (-5.93, -2.35%) (Peanuts vs. Macadamia) to -4.48% (-7.71, -1.24%) (Pistachios vs. Macadamia) to -3.79% (-5.57, -2.01%) (Walnuts vs. Macadamia) to 0.75% (-0.97, 2.47%) (Peanuts vs. Mixed nuts) to 0.41% (-2.78, 3.61%) (Pistachios vs. Mixed nuts) to 1.10% (-0.61, 2.82%) (Walnuts vs. Mixed nuts) to -0.34% (-3.23, 2.55%) (Pistachios vs. Peanuts) to 0.35% (-0.68, 1.38%) (Walnuts vs. Peanuts) to 0.69% (-2.20, 3.58%) (Walnuts vs. Pistachios).
[2]Pairwise between-subgroup mean differences (95% CIs) for Feeding control were as follows:-4.21% (-6.63, -1.78%) (MC vs. DA) to -4.19% (-6.14, -2.25%) (Suppl vs. DA) to 0.01% (-1.54, 1.57%) (Suppl vs. MC).
[3]Pairwise between-subgroup mean differences (95% CIs) for Comparator were as follows: 4.18% (2.60, 5.76%) (Fat vs Carbohydrate) to 0.36% (-0.40, 1.12%) (Mixed macronutrient vs Carbohydrate) to 0.80% (0.65, 1.53%) (No nuts vs Carbohydrate) to -3.82% (-5.43, -2.21%) (Mixed macronutrient vs Fat) to -3.38% (-4.98, -1.78%) (No nuts vs Fat) to 0.44% (-0.37, 1.24%) (No nuts vs Mixed macronutrients).

Supplementary Figure 18. *A priori* subgroup analysis for mean differences (95% CIs) of the effects of nut consumption on waist circumference (cm) (continued on the next page).

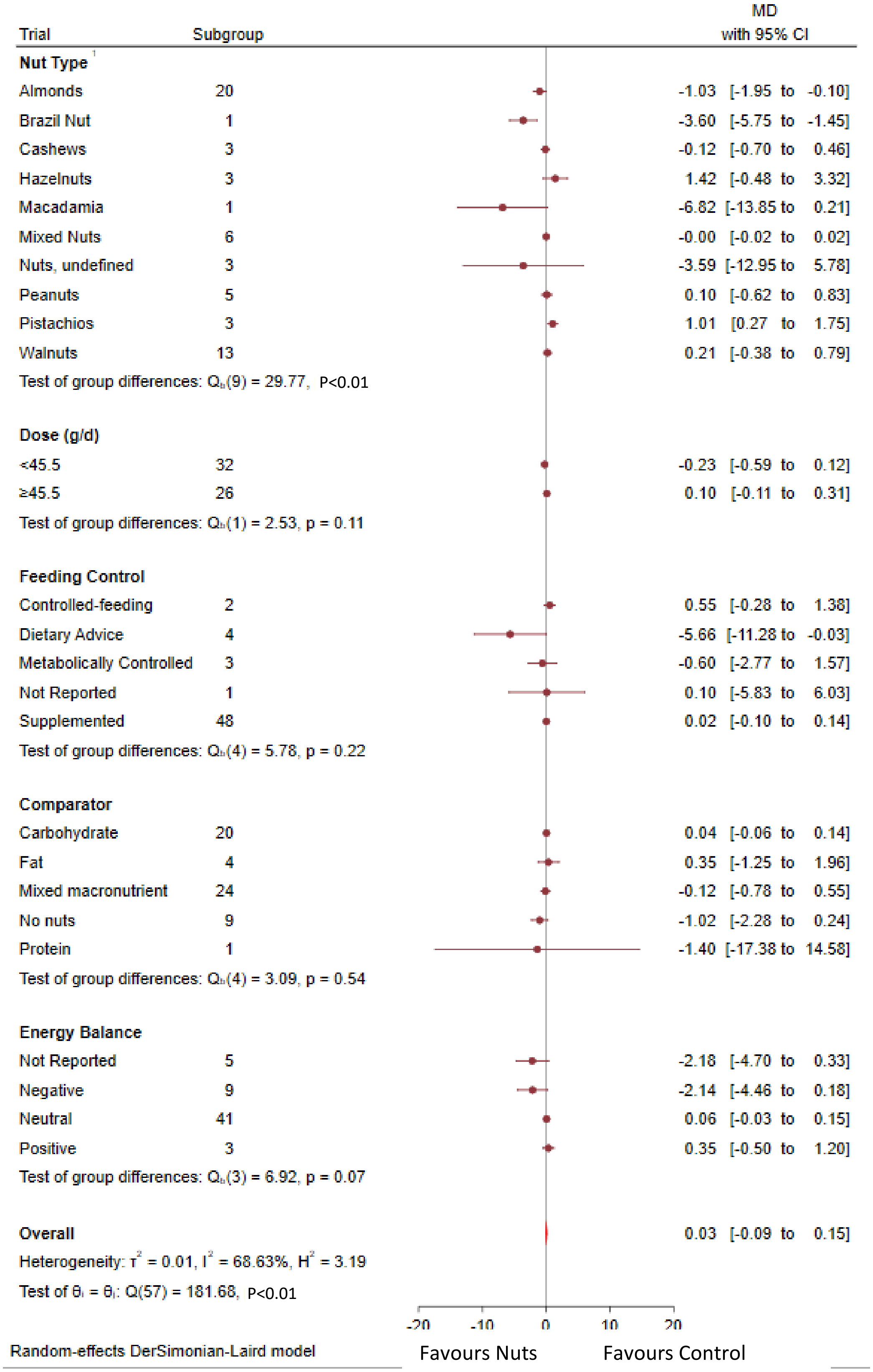

Supplementary Figure 18. *A priori* subgroup analysis for mean differences (95% CIs) of the effects of nut consumption on waist circumference (cm) (continued on the next page).

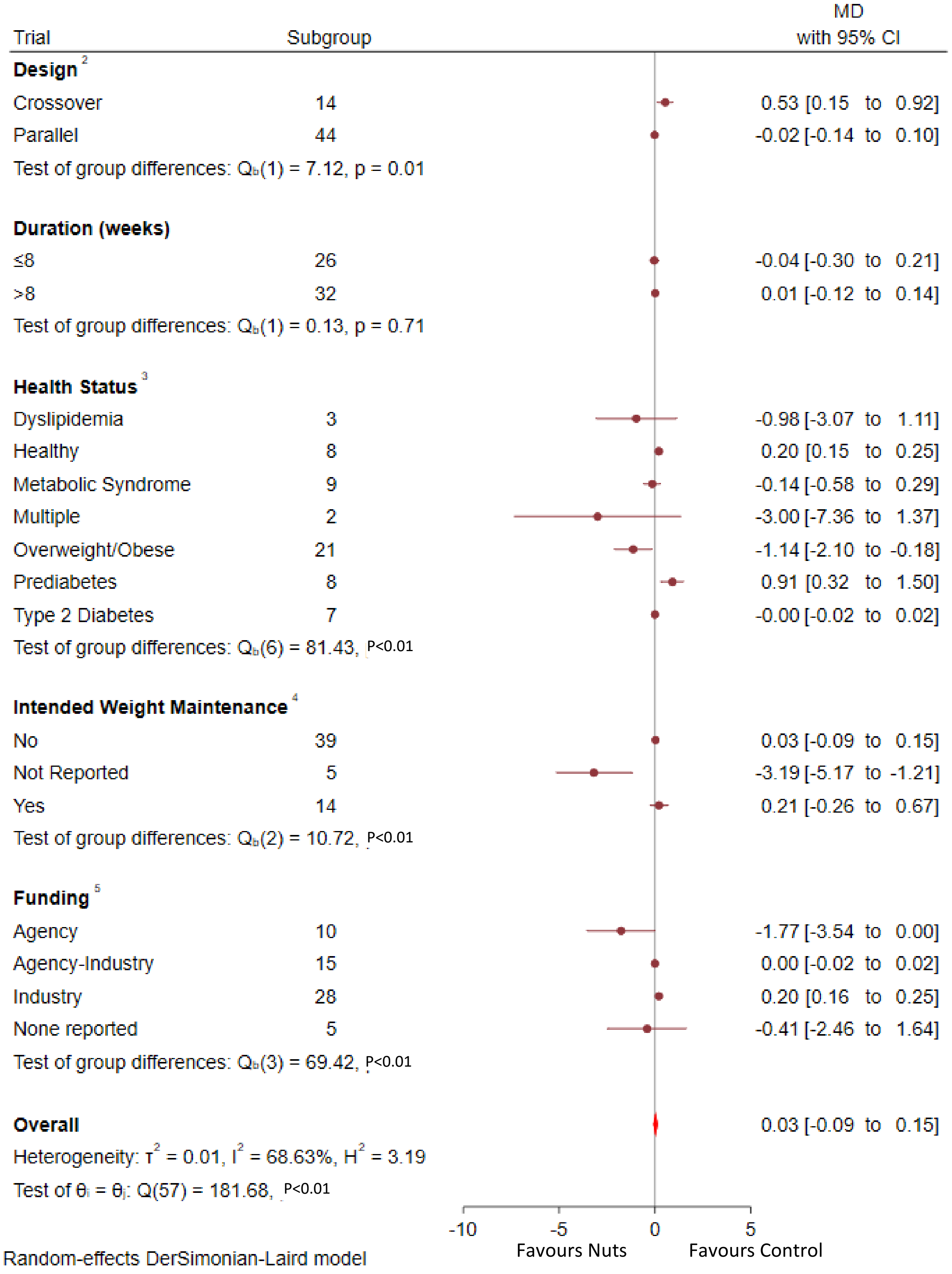

Supplementary Figure 18. *A priori* subgroup analysis for mean differences (95% CIs) of the effects of nut consumption on waist circumference (cm) (continued on next page).
Pooled effect estimates for each subgroup and overall effect are represented by the diamonds. Data are expressed as weighted mean differences with 95% CIs using the random-effects DerSimonian-Laird model. Paired analyses were applied to all crossover trials. Inter-trial heterogeneity was assessed using the Cochran Q statistic and quantified using the I^2 statistic, with significance set at P<0.10 and I^2>50% considered to be evidence of substantial heterogeneity.

CI, confidence interval; DA, dietary advice; Feeding-control is the provision of some meals and foods consumed during the trial; MC, metabolically controlled: is the provision of all meals and foods consumed during the trial under controlled conditions; MD, mean difference; N, no; NR, not reported; SE, standard error; Suppl, supplemented: is the provision of the intervention and control foods during the trial; Y, yes. Negative energy balance refers to a deficit in normal energy intake and/or intake below energy requirements. Neutral energy balance refers to the maintenance of usual energy intake and/or meeting energy requirements. Position energy balance refers to an excess in normal energy intake and/or intake above energy requirements. Weight maintenance intended refers to the trial being designed to maintain participants' body weight during the course of the trial.
Agency funding is that from government, university, or not-for-profit sources. Industry funding is that from trade organizations that obtain revenue from the sale of products.
[1]Pairwise between-subgroup mean differences (95% CIs) for Nut type were as follows: -3.72 cm (-5.89, -1.56 cm) (Brazil nuts vs. Almonds) to -0.24 cm (-0.87, 0.38 cm) (Cashews vs. Almonds) to 1.30 cm (-0.61, 3.21 cm) (Hazelnuts vs. Almonds) to -6.94 cm (-14.00, 0.09 cm) (Macadamia vs. Almonds) to -0.15 cm (-0.35, 0.06 cm) (Mixed Nuts vs. Almonds) to -3.71 cm (-13.10, 5.66 cm) (Undefined nuts vs. Almonds) to -0.03 cm (-0.78, 0.73 cm) (Peanuts vs. Almonds) to 0.89 cm (0.15, 1.66 cm) (Pistachios vs. Almonds) to 0.09 cm (-0.39, 0.58 cm) (Walnuts vs. Almonds) to 3.48 cm (1.24, 5.72 cm) (Cashews vs. Brazil nuts) to 5.02 cm (2.14, 7.90 cm) (Hazelnuts vs. Brazil nuts) to -3.22 cm (-10.60, 4.13 cm) (Macadamia vs. Brazil nuts) to 3.58 cm (1.42, 5.74 cm) (Mixed nuts vs. Brazil nuts) to 0.01 cm (-9.60, 9.62 cm) (Undefined nuts vs. Brazil nuts) to 3.70 cm (1.41, 5.98 cm) (Peanuts vs. Brazil nuts) to 4.61 cm (2.32, 6.90 cm) (Pistachios vs. Brazil nuts) to 3.84 cm (1.42, 5.98 cm) (Walnuts vs. Brazil nuts) to 1.54 cm (-0.45, 3.53 cm) (Hazelnuts vs. Cashews) to -6.70 cm (-13.80, 0.36 cm) (Macadamia vs. Cashews) to 0.10 cm (-0.52, 0.71 cm) (Mixed nuts vs. Cashews) to -3.47 cm (-12.90, 5.92 cm) (Undefined nuts vs. Cashews) to 0.22 cm (-0.73, 1.17 cm) (Peanuts vs. Cashews) to 1.13 cm (0.16, 2.10 cm) (Pistachios vs. Cashews) to 0.34 cm (-0.42, 1.09 cm) (Walnuts vs. Cashews) to -8.24 cm (-15.50, -0.96 cm) (Macadamia vs. Hazelnuts) to -1.44 cm (-3.35, 0.46 cm) (Mixed nuts vs. Hazelnuts) to -5.01 cm (-14.60, 4.55 cm) (Undefined nuts vs. Hazelnuts) to -1.32 cm (-3.36, 0.71 cm) (Peanuts vs. Hazelnuts) to -0.41 cm (-2.46, 1.63 cm) (Pistachios vs. Hazelnuts) to -1.21 cm (-3.16, 0.75 cm) (Walnuts vs. Hazelnuts) to 6.80 cm (-0.23, 13.80 cm) (Mixed nuts vs. Macadamia) to 3.23 cm (-8.48, 14.90 cm) (Undefined nuts vs. Macadamia) to 6.92 cm (-0.15, 14.00 cm) (Peanuts vs. Macadamia) to 7.04 cm (-0.76, 14.90 cm) (Pistachios vs. Macadamia) to 7.04 cm (-0.01, 14.10 cm) (Walnuts vs. Macadamia) to -3.57 cm (-12.90, 5.80 cm) (Undefined nuts vs. Mixed nuts) to 0.12 cm (-0.62, 0.86 cm) (Peanuts vs. Mixed nuts) to 1.03 cm (0.27, 1.80 cm) (Pistachios vs. Mixed nuts) to 0.24 cm (-0.23, 0.71 cm) (Walnuts vs. Mixed nuts) to 3.69 cm (-5.71, 13.10 cm) (Peanuts vs. Undefined nuts) to 4.60 cm (-4.80, 14.00 cm) (Pistachios vs. Undefined nuts) to 3.80 cm (-5.57, 13.20 cm) (Walnuts vs. Undefined nuts) to 0.91 cm (-0.14, 1.97 cm) (Pistachios vs. Peanuts) to 0.12 cm (-0.75, 0.98 cm) (Walnuts vs. Peanuts) to -0.80 cm (-1.68, 0.09 cm) (Walnuts vs. Pistachios).
[2]Pairwise between-subgroup mean differences (95% CIs) for Design were as follows:-0.54 cm (-0.96, -0.13 cm) (Parallel vs. Crossover).
[3]Pairwise between-subgroup mean differences (95% CIs) for Health Status were as follows: 1.17 cm (-0.93, 3.28 cm) (Healthy vs. Dyslipidemia) to 0.84 cm (-1.30 vs. 2.98 cm) (Metabolic Syndrome vs. Dyslipidemia) to

Supplementary Figure 18. *A priori* subgroup analysis for mean differences (95% CIs) of the effects of nut consumption on waist circumference (cm).

-2.73 cm (-6.06, 0.60 cm) (Multiple vs. Dyslipidemia) to 0.61 cm (-1.52, 2.75 cm) (Overweight/Obese vs. Dyslipidemia) to 1.89 cm (-0.29, 4.06 cm) (Prediabetes vs. Dyslipidemia) to 0.97 cm (-1.12, 3.07 cm) (Type 2 Diabetes vs. Dyslipidemia) to -0.33 cm (-0.80, 0.14 cm) (Metabolic Syndrome vs. Healthy) to -3.90 cm (-6.49, -1.31 cm) (Multiple vs. Healthy) to -0.56 cm (-0.99, -0.13 cm) (Overweight/Obese vs. Healthy) to 0.71 cm (0.09, 1.33 cm) (Prediabetes vs. Healthy) to -0.20 cm (-0.40, 0.002 cm) (Type 2 Diabetes vs. Healthy) to –3.57 cm (-6.20, -0.94 cm) (Multiple vs. Metabolic Syndrome) to -0.23 cm (-0.83, 0.36 cm) (Overweight/Obese vs. Metabolic Syndrome) to 1.04 cm (0.30, 1.78 cm) (Prediabetes vs. Metabolic Syndrome) to 0.13 cm (-0.33, 0.59 cm) (Type 2 Diabetes vs. Metabolic Syndrome) to 3.34 cm (0.72, 5.96 cm) (Overweight/Obese vs. Multiple) to 4.61 cm (1.96, 7.27 cm) (Prediabetes vs. Multiple) to 3.70 cm (1.11, 6.29 cm) (Type 2 Diabetes vs. Multiple) to 1.27 cm (0.55, 1.99 cm) (Prediabetes vs. Overweight/Obese) to 0.36 cm (-0.05, 0.77 cm) (Type 2 Diabetes vs. Overweight/Obese) to -0.91 cm (-1.52, -0.30 cm).
[4]Pairwise between-subgroup mean differences (95% CIs) for Weight Maintenance Intended were as follows: -3.22 cm(-5.21, -1.22 cm) (NR vs. N) to 0.18 cm(-0.31, 0.66 cm) (Y vs. N) to 3.39 cm(1.35, 5.43 cm) (Y vs. NR).
[5]Pairwise between-subgroup mean differences (95% CIs) for Funding Source were as follows: 1.09 cm(0.56, 1.61 cm) (AI vs. A) to 1.29 cm(0.75, 1.82 cm) (I vs. A) to 0.67 cm(-1.45, 2.79 cm) (NR vs. A) to 0.20 cm(0.02, 0.39 cm) (I vs. AI) to -0.42 cm(-2.48, 1.64 cm) (NR vs. AI) to -0.62 cm(-2.68, 1.45 cm) (NR vs. I), where A=agency, AI = agency-industry, I=industry, NR=not reported.

Supplementary Figure 19. *A priori* subgroup analysis for mean differences (95% CIs) of the effects of nut consumption on waist-to-hip ratio (continued on the next page).

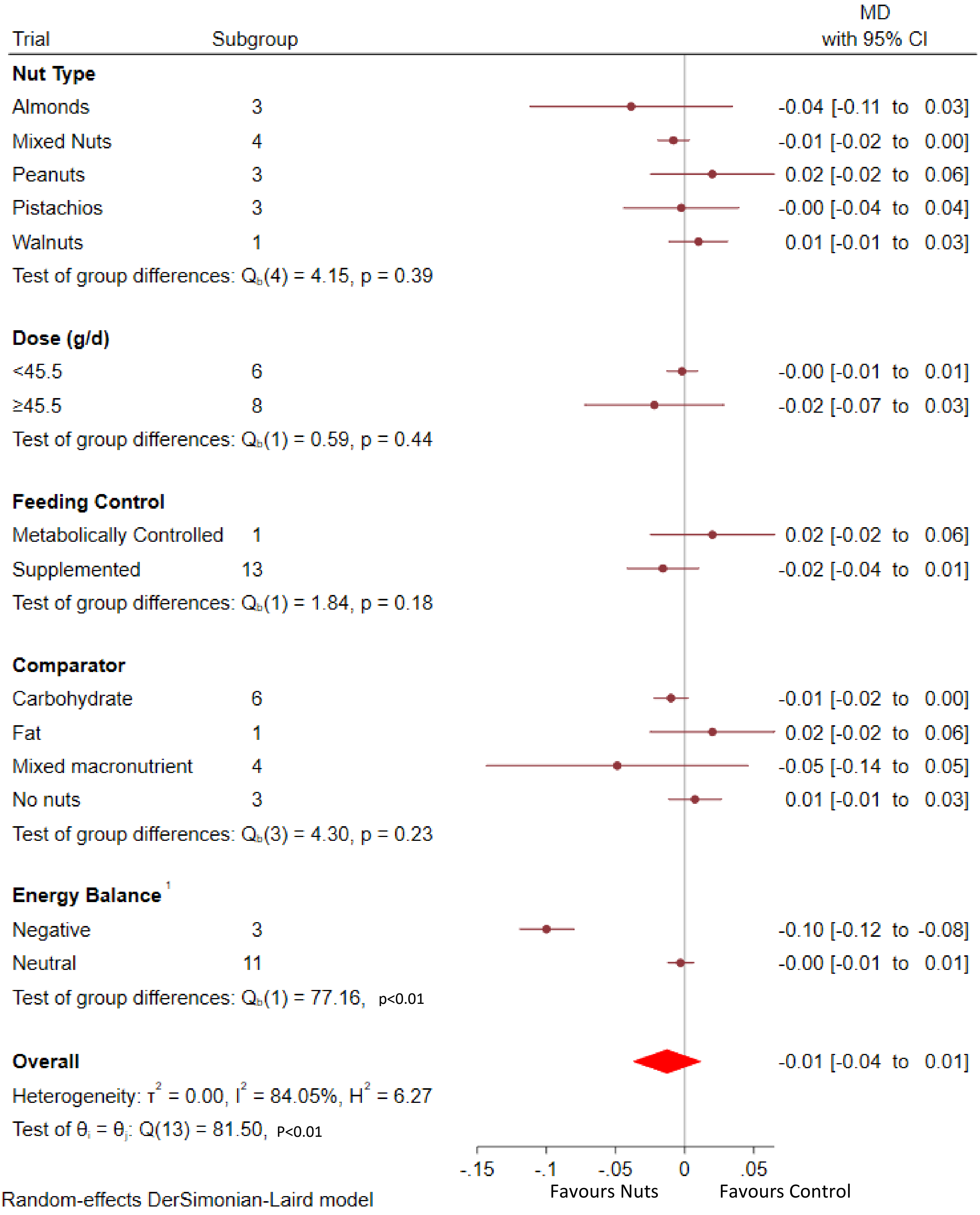

Supplementary Figure 19. *A priori* subgroup analysis for mean differences (95% CIs) of the effects of nut consumption on waist-to-hip ratio (continued on the next page).

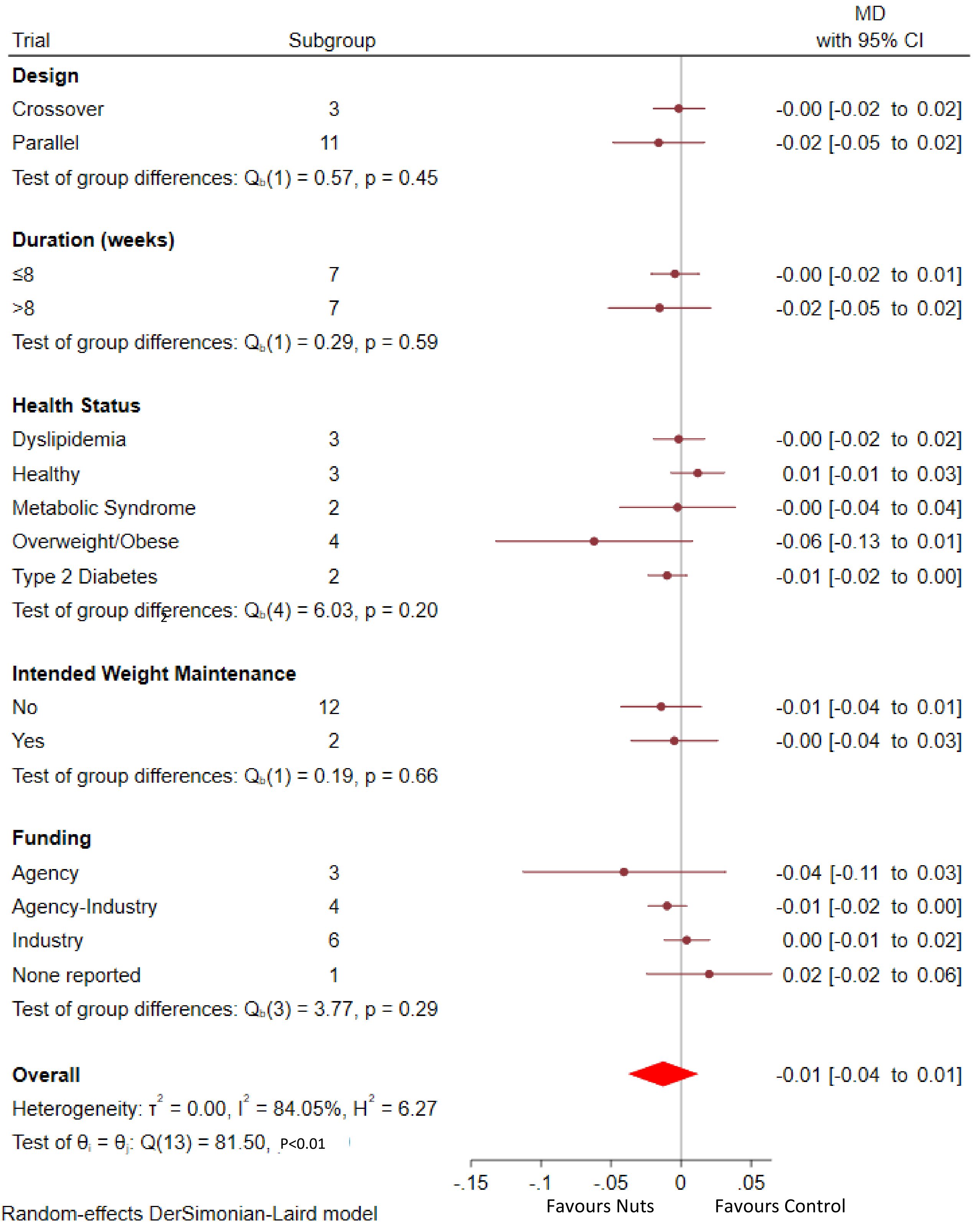

Supplementary Figure 19. *A priori* subgroup analysis for mean differences (95% CIs) of the effects of nut consumption on waist-to-hip ratio.

Pooled effect estimates for each subgroup and overall effect are represented by the diamonds. Data are expressed as weighted mean differences with 95% CIs using the random-effects DerSimonian-Laird model. Paired analyses were applied to all crossover trials. Inter-trial heterogeneity was assessed using the Cochran Q statistic and quantified using the I^2 statistic, with significance set at P<0.10 and I^2>50% considered to be evidence of substantial heterogeneity.

CI, confidence interval; DA, dietary advice; Feeding-control is the provision of some meals and foods consumed during the trial; MC, metabolically controlled: is the provision of all meals and foods consumed during the trial under controlled conditions; MD, mean difference; N, no; NR, not reported; SE, standard error; Suppl, supplemented: is the provision of the intervention and control foods during the trial; Y, yes. Negative energy balance refers to a deficit in normal energy intake and/or intake below energy requirements. Neutral energy balance refers to the maintenance of usual energy intake and/or meeting energy requirements. Position energy balance refers to an excess in normal energy intake and/or intake above energy requirements. Weight maintenance intended refers to the trial being designed to maintain participants' body weight during the course of the trial.
Agency funding is that from government, university, or not-for-profit sources. Industry funding is that from trade organizations that obtain revenue from the sale of products.
[1]Pairwise between-subgroup mean differences (95% CIs) for Energy Balance were as follows: 0.10 (0.08, 0.12) (Neutral vs. Negative).

Supplementary Figure 20. Forest plot of prospective cohorts investigating the association of nut consumption on overweight/obesity risk using a fixed-effects model.

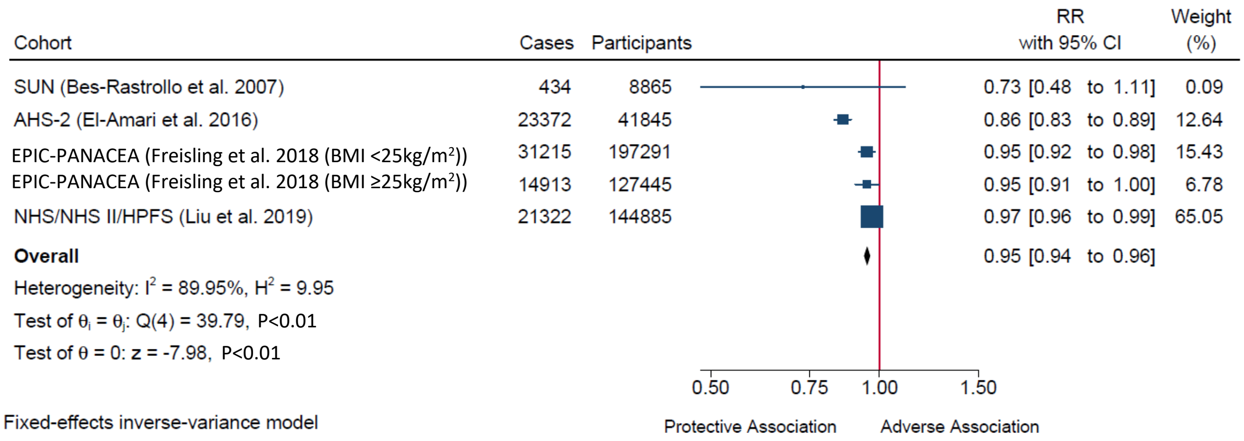

AHS-2=Adventist Health Study 2, EPIC -PANACEA= European Prospective Investigation into Cancer and Nutrition – Physical Activity, Nutrition, Alcohol, Cessation of smoking, Eating out of home in relation to Anthropometry, HPFS = Health Professionals Follow-Up Study, NHS = Nurses' Health Study, NHS II = Nurses' Health Study II, Sun = Seguimiento Universidad de Navarra study.

The black diamond represents the pooled risk estimate. Inter-study heterogeneity was tested using the Cochran Q statistic at a significance level of p<0.10, and quantified by the I^2 statistic. An I^2 value ≥50% is considered as indicative of substantial heterogeneity. All results are presented as Relative Risks (RR) with 95% Confidence Intervals (CI).

Supplementary Figure 21a. Forest plot of prospective cohorts investigating the association of nut consumption on body weight change (kg) with the use of a fixed-effects model.

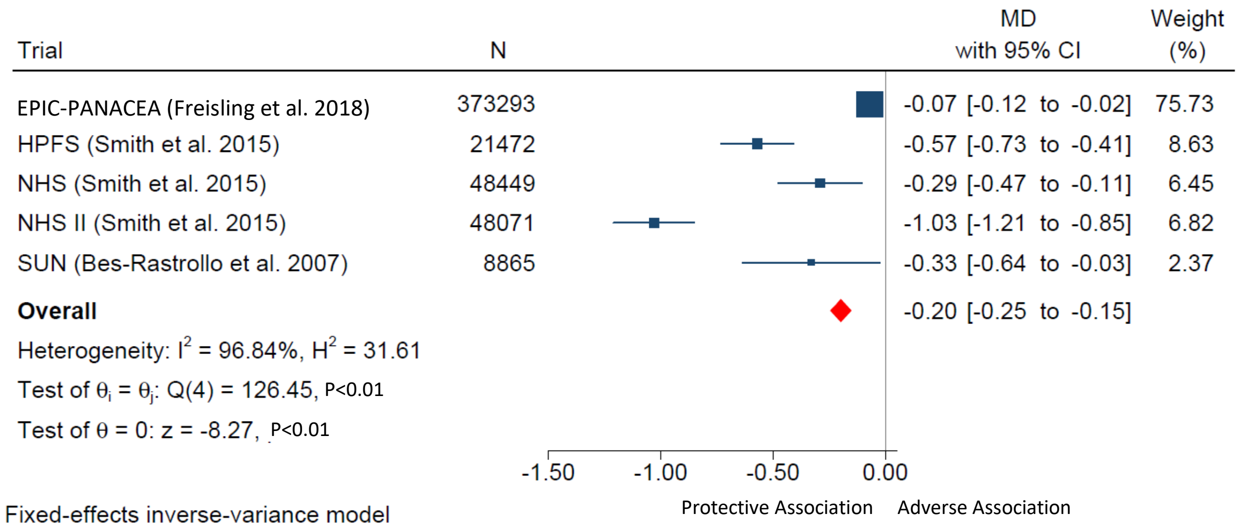

Supplementary Figure 21b. Forest plot of prospective cohorts investigating the association of nut consumption on body weight change (kg) with the use of a fixed-effects model, using the least adjusted data.

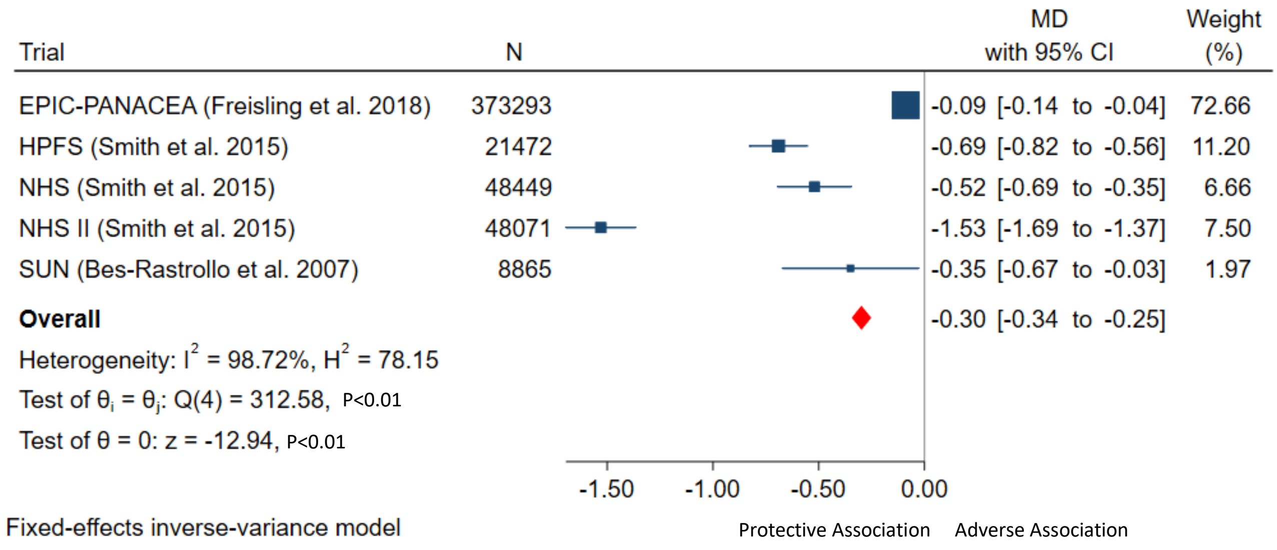

EPIC -PANACEA= European Prospective Investigation into Cancer and Nutrition – Physical Activity, Nutrition, Alcohol, Cessation of smoking, Eating out of home in relation to Anthropometry, HPFS = Health Professionals Follow-Up Study, NHS = Nurses' Health Study, NHS II = Nurses' Health Study II, Sun = Seguimiento Universidad de Navarra study.

The black diamond represents the pooled risk estimate. Inter-study heterogeneity was tested using the Cochran Q statistic at a significance level of $p<0.10$, and quantified by the I^2 statistic. An I^2 value ≥50% is considered as indicative of substantial heterogeneity. All results are presented as Mean Differences (MD) with 95% Confidence Intervals (CI).

Supplementary Figure 22. Forest plot of prospective cohorts investigating the association of nut consumption on weight gain (≥5 kg) incidence with the use of a random-effects model.

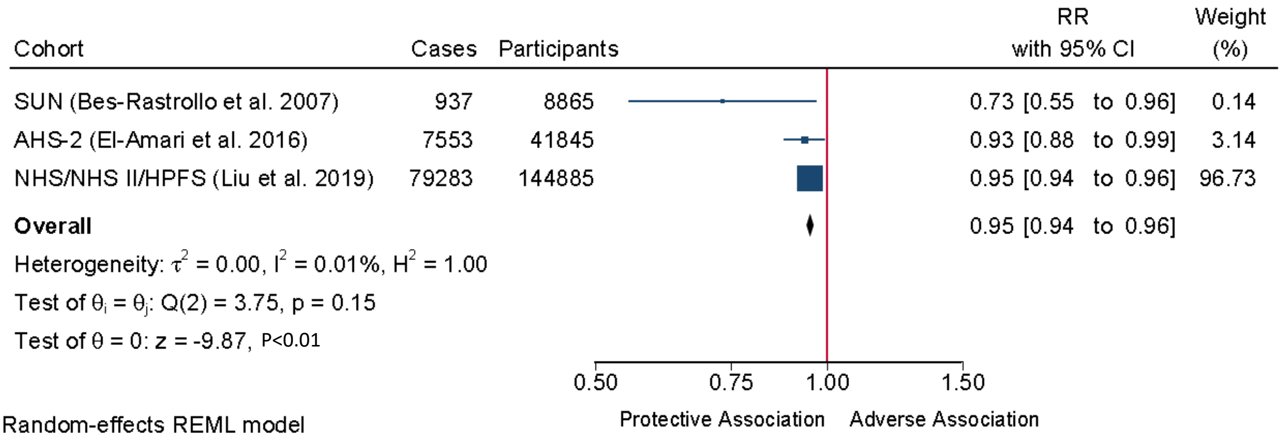

AHS-2=Adventist Health Study 2, HPFS = Health Professionals Follow-Up Study, NHS = Nurses' Health Study, NHS II = Nurses' Health Study II, Sun = Seguimiento Universidad de Navarra study.

The black diamond represents the pooled risk estimate. Inter-study heterogeneity was tested using the Cochran Q statistic at a significance level of $p<0.10$, and quantified by the I^2 statistic. An I^2 value ≥50% is considered as indicative of substantial heterogeneity. All results are presented as Relative Risks (RR) with 95% Confidence Intervals (CI).

Supplementary Figure 23. Forest plot of prospective cohorts investigating the association of nut consumption on the incidence of waist circumference increasing ≥94 cm in men and ≥80 cm in women with the use of a random-effects model.

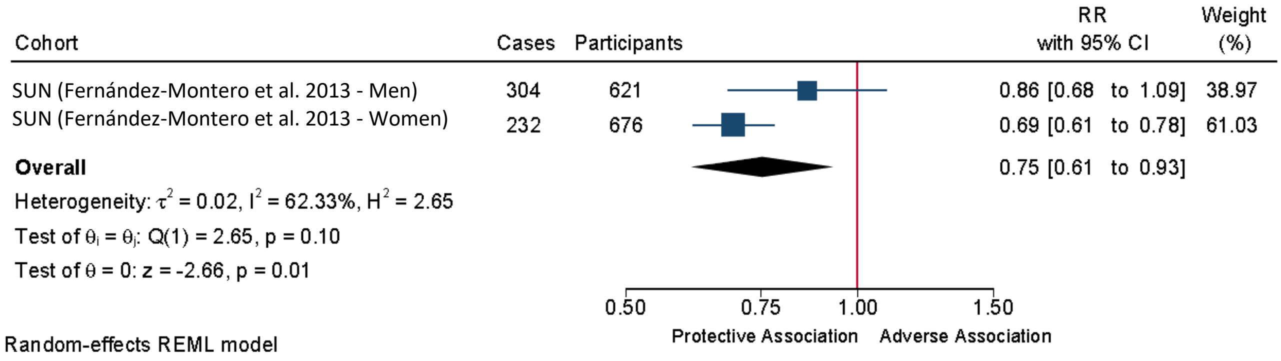

Sun = Seguimiento Universidad de Navarra study.

The black diamond represents the pooled risk estimate. Inter-study heterogeneity was tested using the Cochran Q statistic at a significance level of $p<0.10$, and quantified by the I^2 statistic. An I^2 value ≥50% is considered as indicative of substantial heterogeneity. All results are presented as Relative Risks (RR) with 95% Confidence Intervals (CI).

Supplementary Figure 24. Forest plot of randomized controlled trials investigating the effects of nut consumption on body weight (kg) with the use of a fixed-effects model (continued on the next page).

Trial	N	MD with 95% CI	Weight (%)
Abazarfard et al 2014	100	-2.41 [-3.68 to -1.14]	0.19
Abbaspour et al. 2019	48	-2.00 [-9.41 to 5.41]	0.01
Agebratt et al. 2016	30	-0.03 [-6.43 to 6.37]	0.01
Baer et al. 2019	40	0.00 [-0.19 to 0.19]	7.93
Balci et al. 2012	60	0.90 [-5.81 to 7.61]	0.01
Bamberger et al. 2017	204	0.10 [-1.61 to 1.81]	0.10
Barbour et al. 2015	61	0.50 [0.11 to 0.89]	1.96
Bento et al. 2014	20	-0.30 [-5.26 to 4.66]	0.01
Berryman et al. 2015	48	-0.30 [-3.24 to 2.64]	0.03
Bitok et al. 2018	317	0.20 [-3.44 to 3.84]	0.02
Biude Silva Duarte et al. 2019	55	-0.70 [-1.65 to 0.25]	0.33
Bowen et al. 2019	76	0.10 [-0.61 to 0.81]	0.59
Campbell et al. 2019	12	-6.10 [-17.82 to 5.62]	0.00
Canales et al. 2007	22	-0.20 [-19.62 to 19.22]	0.00
Carughi et al. 2019	60	0.20 [-2.84 to 3.24]	0.03
Casas-Agustench et al. 2011	50	-0.70 [-2.11 to 0.71]	0.15
Chisholm et al. 2005	28	0.00 [-0.52 to 0.52]	1.09
Ciccone et al. 2014 [Control]	31	-3.70 [-17.90 to 10.50]	0.00
Ciccone et al. 2014 [Non-fried fish]	15	-2.00 [-17.87 to 13.87]	0.00
Ciccone et al. 2014 [Olive Oil]	15	-6.40 [-23.19 to 10.39]	0.00
Cohen et al. 2011	13	-2.80 [-30.31 to 24.71]	0.00
Damasceno et al. 2011 [Almond]	18	0.11 [-6.82 to 7.04]	0.01
Damasceno et al. 2011 [Walnut]	18	0.06 [-6.82 to 6.94]	0.01
Damavandi et al. 2012	43	-0.90 [-7.48 to 5.68]	0.01
Damavandi et al. 2013	48	-0.32 [-5.89 to 5.25]	0.01
DeSouza et al. 2018	46	-0.34 [-8.29 to 7.61]	0.00
Dhillon et al. 2016	86	-1.13 [-2.41 to 0.15]	0.19
Dhillon et al. 2018	73	-0.60 [-0.85 to -0.35]	4.64
Foster et al. 2012	123	2.20 [-0.57 to 4.97]	0.04
Gebauer et al. 2008 [High Dose Pistachio]	28	-0.30 [-8.83 to 8.23]	0.00
Gebauer et al. 2008 [Low Dose Pistachio]	28	-0.10 [-8.63 to 8.43]	0.00
Gulati et al. 2014	68	-1.10 [-6.25 to 4.05]	0.01
Hernández-Alonso et al. 2014	49	0.19 [-0.30 to 0.68]	1.23
Hiraoka-Yamamoto et al. 2004 [Butter]	35	-0.40 [-4.43 to 3.63]	0.02
Hiraoka-Yamamoto et al. 2004 [Coconut]	36	-0.30 [-4.27 to 3.67]	0.02
Hollis and Mattes. 2007	20	0.80 [-3.59 to 5.19]	0.02
Hudthagosol et al. 2012 [Control]	25	1.70 [-0.69 to 4.09]	0.05
Hudthagosol et al. 2012 [Salmon]	25	0.30 [-2.10 to 2.70]	0.05

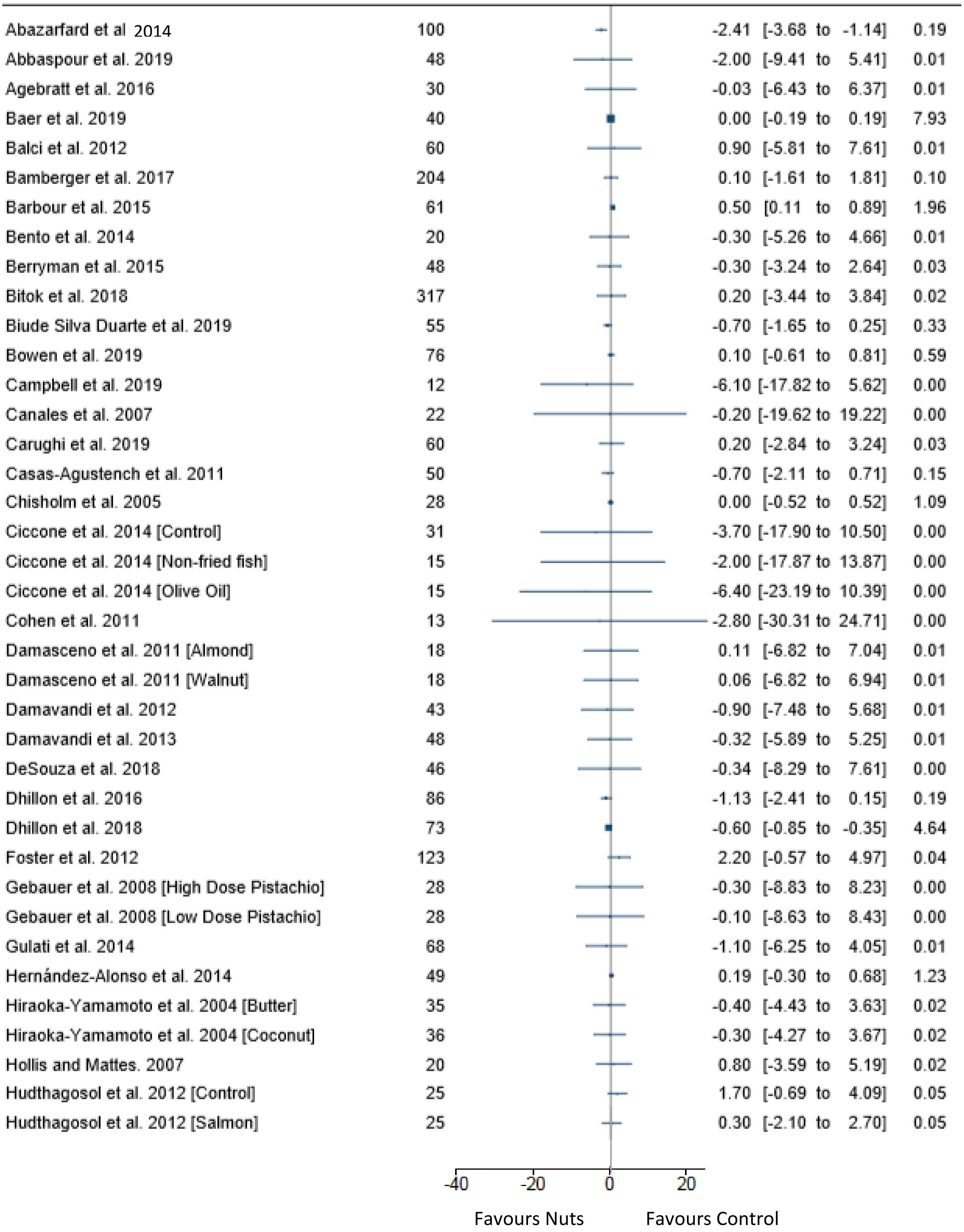

Supplementary Figure 24. Forest plot of randomized controlled trials investigating the effects of nut consumption on body weight (kg) with the use of a fixed-effects model (continued on the next page).

Trial	N	MD with 95% CI	Weight (%)
Jamshed et al. 2015 [American Almonds]	75	1.00 [-0.41 to 2.41]	0.15
Jamshed et al. 2015 [Pakistani Almonds]	72	5.00 [3.59 to 6.41]	0.15
Jenkins et al. 2002 [Full Dose Almonds]	27	-0.40 [-7.26 to 6.46]	0.01
Jenkins et al. 2002 [Half Dose Almonds]	27	-0.40 [-7.12 to 6.32]	0.01
Jenkins et al. 2018 [Full Dose Nuts]	100	-0.33 [-1.09 to 0.43]	0.52
Jenkins et al. 2018 [Half Dose Nuts]	100	0.40 [-0.39 to 1.19]	0.48
Johnston et al. 2013	44	1.10 [0.12 to 2.08]	0.31
Jung et al. 2018	84	-0.20 [-2.08 to 1.68]	0.09
Katz et al. 2012	46	1.05 [0.42 to 1.67]	0.76
Le et al. 2016 [Lower CHO]	137	-1.10 [-5.94 to 3.74]	0.01
Le et al. 2016 [Lower Fat]	147	0.70 [-3.64 to 5.04]	0.02
Lee et al. 2014	60	0.01 [-0.56 to 0.58]	0.94
Li et al. 2010	52	-1.00 [-6.50 to 4.50]	0.01
Liu et al. 2018	85	-0.54 [-1.59 to 0.51]	0.27
Ma et al. 2010	22	-0.60 [-1.78 to 0.58]	0.21
McKay et al. 2018	26	-0.24 [-5.25 to 4.77]	0.01
Mohan et al. 2018	269	0.32 [-0.02 to 0.66]	2.61
Moreira Alves et al. 2014 [Conventional Peanut]	44	0.78 [-0.34 to 1.90]	0.24
Moreira Alves et al. 2014 [High Oleic Peanut]	43	0.54 [-0.63 to 1.71]	0.22
Morgan et al. 2000	19	0.00 [-10.59 to 10.59]	0.00
Nagashree et al. 2017	58	0.12 [-4.48 to 4.72]	0.01
Njike et al. 2017	34	-0.20 [-1.44 to 1.04]	0.19
Rajaram et al. 2001	23	-0.43 [-0.50 to -0.36]	55.59
Robbins et al. 2012	109	0.25 [-0.59 to 1.09]	0.43
Rock et al. 2017	97	0.60 [-1.52 to 2.72]	0.07
Ros et al. 2004	20	0.20 [-4.30 to 4.70]	0.01
Ruisinger et al. 2015	48	0.45 [-7.39 to 8.30]	0.00
Sabate et al. 2003 [High Dose Almond]	25	-0.30 [-7.78 to 7.18]	0.01
Sabate et al. 2003 [Low Dose Almond]	25	0.20 [-7.28 to 7.68]	0.01
Sabaté et al. 2005	90	0.40 [0.20 to 0.60]	7.83
Sauder et al. 2015	30	-0.10 [-6.76 to 6.56]	0.01
Schutte et al. 2006 [Cashew]	42	0.34 [-0.74 to 1.42]	0.26
Schutte et al. 2006 [Walnut]	41	0.29 [-0.57 to 1.15]	0.41
Sheridan et al. 2007	15	0.00 [-5.77 to 5.77]	0.01
Somerset et al. 2013	64	-4.27 [-13.01 to 4.47]	0.00
Spaccarotella et al. 2008	21	0.90 [-4.74 to 6.54]	0.01
Spiller et al. 1998 [Cheddar Cheese/Butter]	30	1.00 [-9.53 to 11.53]	0.00
Spiller et al. 1998 [Olive oil]	33	2.00 [-9.23 to 13.23]	0.00
Sweazea et al. 2014	21	1.18 [-1.35 to 3.71]	0.05

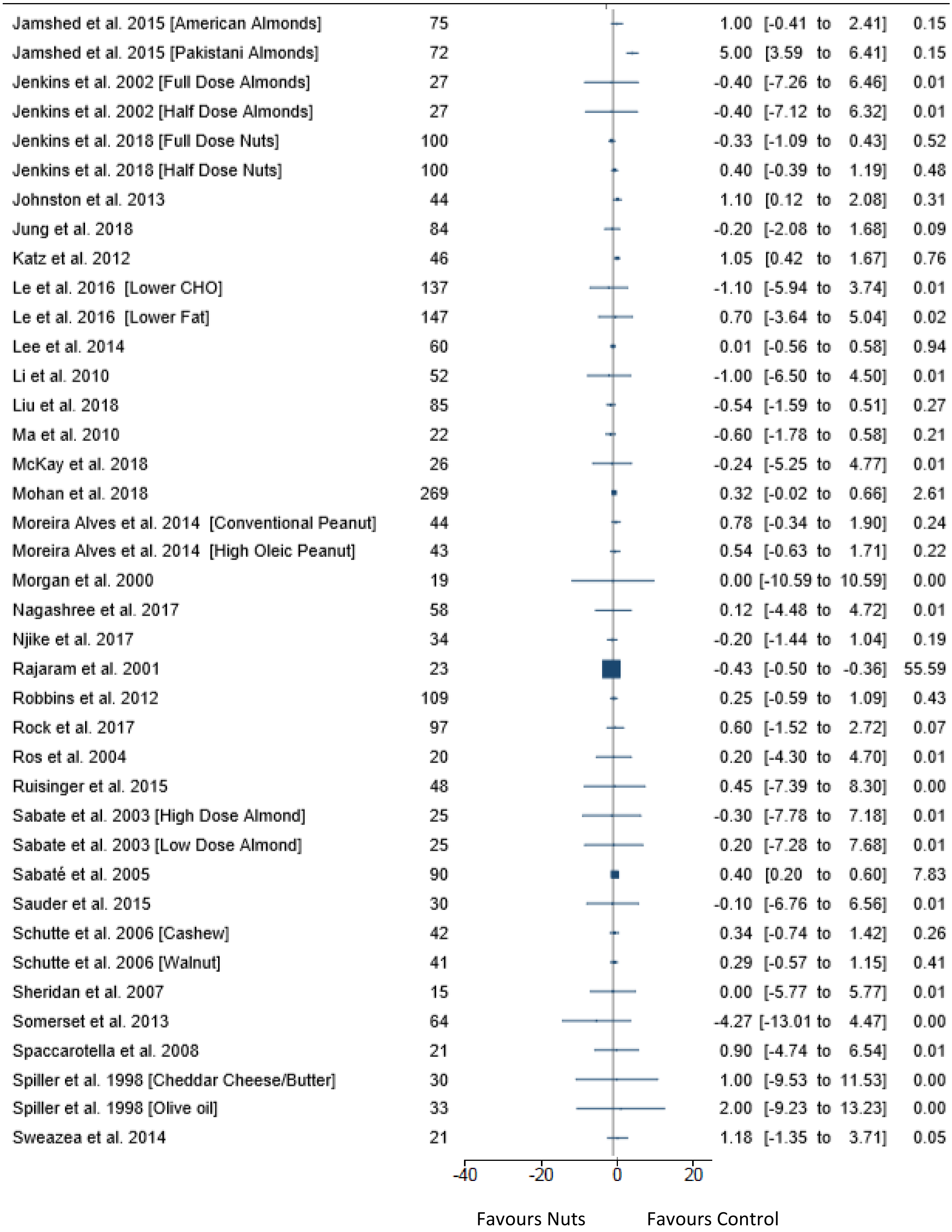

Supplementary Figure 24. Forest plot of randomized controlled trials investigating the effects of nut consumption on body weight (kg) with the use of a fixed-effects model (continued on the next page).

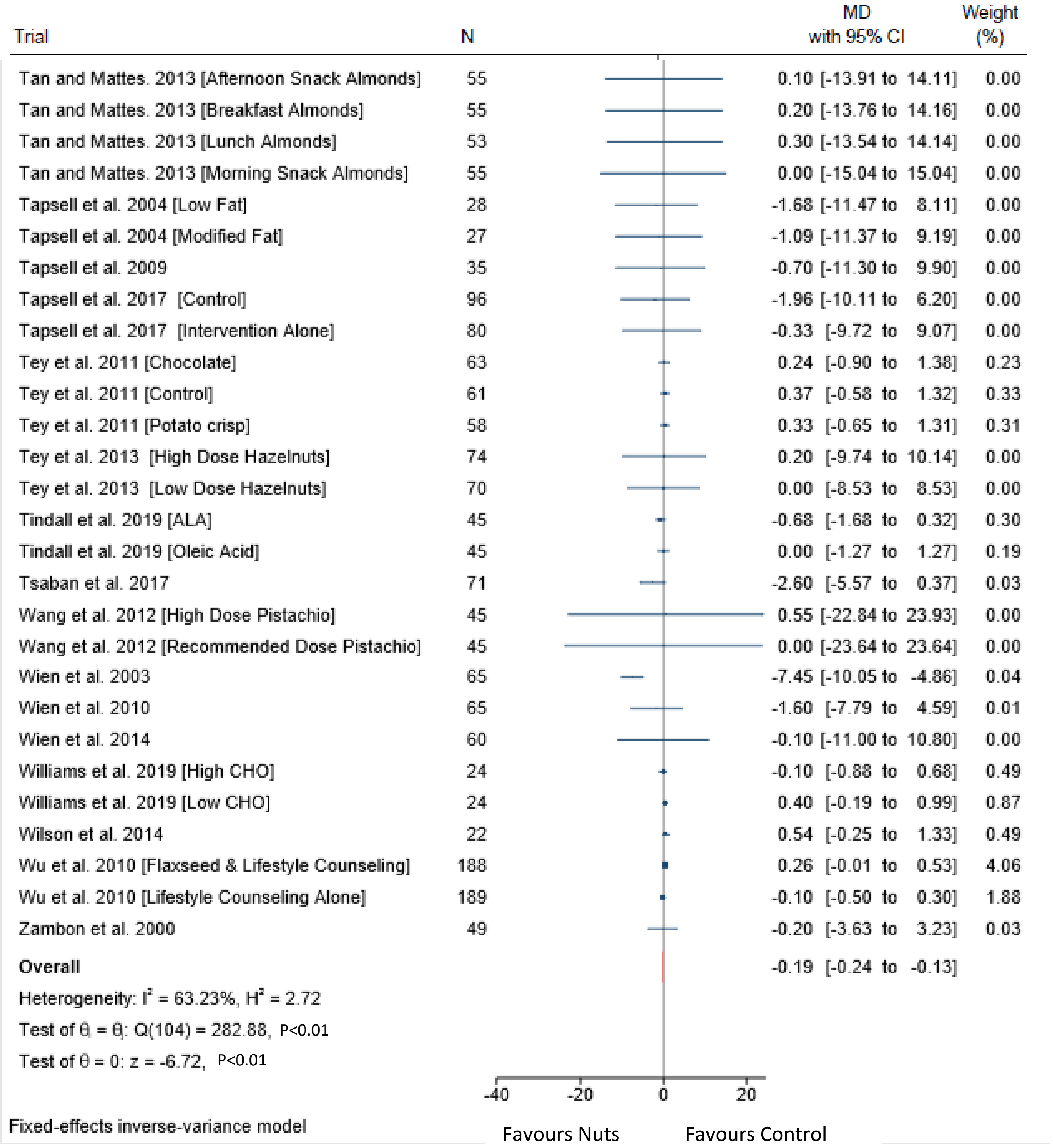

Pooled effect estimate is represented by the diamond and was estimated with the use of fixed effects inverse-variance model.
To avoid unit of analysis error, standard error, used for determining the 95% confidence interval, was calculated by splitting the N for studies with multiple comparisons as per the Cochrane Handbook, 2019.
CI, confidence interval; MD, mean difference, N, number of participants.

Supplementary Figure 25. Forest plot of randomized controlled trials investigating the effects of nut consumption on body mass index (BMI) (kg/m^2) with the use of a fixed-effects model (continued on the next page).

Trial	N	MD with 95% CI	Weight (%)
Abazarfard et al. 2014	100	-0.96 [-1.47 to -0.45]	1.08
Abbaspour et al. 2019	48	-0.60 [-2.30 to 1.10]	0.10
Agebratt et al. 2016	30	0.04 [-1.38 to 1.46]	0.14
Bamberger et al. 2017	204	0.00 [-0.51 to 0.51]	1.06
Barbour et al. 2015	61	0.30 [-0.74 to 1.34]	0.25
Bento et al. 2014	20	0.00 [-1.09 to 1.09]	0.23
Bitok et al. 2018	317	-0.10 [-1.17 to 0.97]	0.24
Biude Silva Duarte et al. 2019	55	0.00 [-0.36 to 0.36]	2.09
Canales et al. 2007	22	-0.20 [-1.69 to 1.29]	0.12
Casas-Agustench et al. 2011	50	-0.21 [-0.73 to 0.30]	1.03
Chisholm et al. 2005	28	0.00 [-1.21 to 1.21]	0.19
Ciccone et al. 2014 [Control]	31	-1.00 [-6.21 to 4.21]	0.01
Ciccone et al. 2014 [Non-fried fish]	15	-2.40 [-8.19 to 3.39]	0.01
Ciccone et al. 2014 [Olive Oil]	15	-0.70 [-6.45 to 5.05]	0.01
Cohen et al. 2011	13	-1.40 [-9.68 to 6.88]	0.00
Damasceno et al. 2011 [Almond]	18	0.04 [-1.47 to 1.55]	0.12
Damasceno et al. 2011 [Walnut]	18	0.04 [-1.47 to 1.55]	0.12
Damavandi et al. 2012	43	-0.10 [-4.01 to 3.81]	0.02
Damavandi et al. 2013	48	-0.42 [-2.45 to 1.61]	0.07
DeSouza et al. 2018	46	-0.08 [-11.96 to 11.80]	0.00
Gebauer et al. 2008 [High Dose Pistachio]	28	-0.10 [-2.04 to 1.84]	0.07
Gebauer et al. 2008 [Low Dose Pistachio]	28	-0.10 [-2.04 to 1.84]	0.07
Hernández-Alonso et al. 2014	49	0.19 [-0.00 to 0.38]	7.58
Hiraoka-Yamamoto et al. 2004 [Butter]	35	-0.30 [-1.78 to 1.18]	0.13
Hiraoka-Yamamoto et al. 2004 [Coconut]	36	-0.10 [-1.46 to 1.26]	0.15
Hudthagosol et al. 2012 [Control]	25	0.08 [-0.52 to 0.68]	0.76
Hudthagosol et al. 2012 [Salmon]	25	0.10 [-0.50 to 0.70]	0.76
Jenkins et al. 2002 [Full Dose Almonds]	27	-0.10 [-1.21 to 1.01]	0.22
Jenkins et al. 2002 [Half Dose Almonds]	27	-0.20 [-1.30 to 0.90]	0.23
Jenkins et al. 2018 [Full Dose Nuts]	100	-0.12 [-0.39 to 0.15]	3.67
Jenkins et al. 2018 [Half Dose Nuts]	100	0.14 [-0.14 to 0.42]	3.55
Jung et al. 2018	84	-0.10 [-0.54 to 0.34]	1.43
Katz et al. 2012	46	0.40 [0.16 to 0.64]	4.66
Kocyigit et al. 2006	44	0.20 [-3.19 to 3.59]	0.02
Le et al. 2016 [Lower CHO]	137	-0.40 [-1.83 to 1.03]	0.13
Le et al. 2016 [Lower Fat]	147	0.20 [-1.11 to 1.51]	0.16
Lee et al. 2014	60	0.00 [-0.24 to 0.24]	4.67
Li et al. 2010	52	-0.70 [-1.89 to 0.49]	0.19

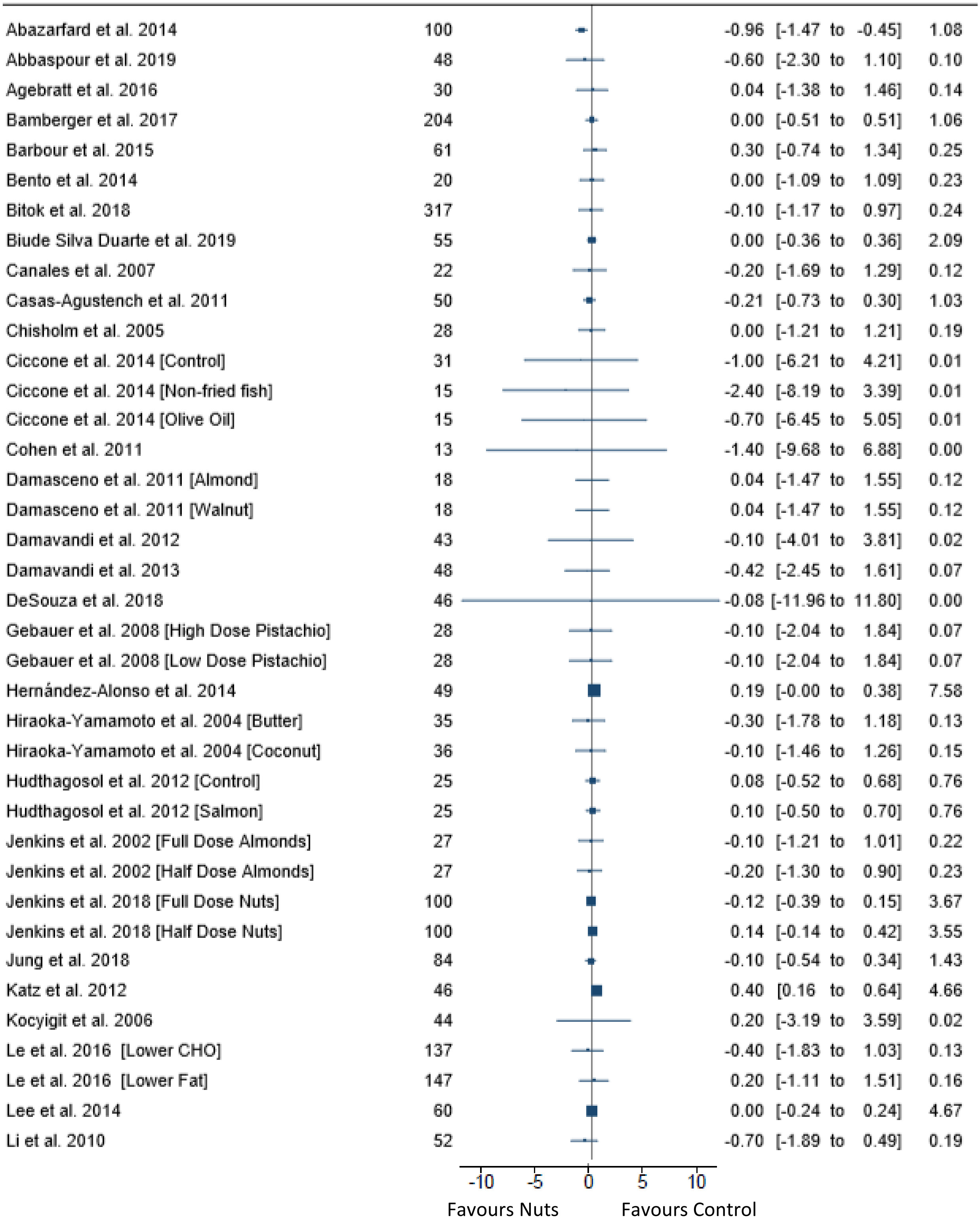

Supplementary Figure 25. Forest plot of randomized controlled trials investigating the effects of nut consumption on body mass index (BMI) (kg/m^2) with the use of a fixed-effects model (continued on the next page).

Trial	N	MD with 95% CI	Weight (%)
Li et al. 2011	20	0.10 [-1.33 to 1.53]	0.14
Liu et al. 2018	85	-0.11 [-0.49 to 0.27]	1.94
Ma et al. 2010	22	-0.30 [-0.81 to 0.21]	1.05
McKay et al. 2018	26	-0.07 [-1.29 to 1.15]	0.19
Mohan et al. 2018	269	0.12 [-0.01 to 0.25]	16.33
Moreira Alves et al. 2014 [Conventional Peanut]	44	0.23 [-0.12 to 0.58]	2.22
Moreira Alves et al. 2014 [High Oleic Peanut]	43	0.15 [-0.21 to 0.51]	2.10
Morgan et al. 2000	19	0.00 [-4.05 to 4.05]	0.02
Morgan et al. 2002	67	0.00 [-1.96 to 1.96]	0.07
Nagashree et al. 2017	58	0.00 [-1.06 to 1.06]	0.24
Njike et al. 2015 [Ad libitum]	49	0.47 [0.03 to 0.91]	1.44
Njike et al. 2015 [Calorie adjusted]	48	0.19 [-0.44 to 0.82]	0.70
Njike et al. 2017	34	-0.10 [-0.54 to 0.34]	1.44
Parham et al. 2014	44	-0.50 [-0.87 to -0.13]	1.99
Robbins et al. 2012	109	-0.78 [-1.06 to -0.50]	3.46
Rock et al. 2017	97	0.20 [-1.33 to 1.73]	0.12
Ruisinger et al. 2015	48	0.30 [-1.51 to 2.11]	0.08
Sabaté et al. 2005	90	0.20 [0.00 to 0.40]	7.18
Sauder et al. 2015	30	0.00 [-2.16 to 2.16]	0.06
Schutte et al. 2006 [Cashew]	42	-0.20 [-3.13 to 2.73]	0.03
Schutte et al. 2006 [Walnut]	41	0.10 [-3.22 to 3.42]	0.03
Sheridan et al. 2007	15	0.14 [-1.55 to 1.83]	0.10
Somerset et al. 2013	64	-0.76 [-3.57 to 2.05]	0.03
Spaccarotella et al. 2008	21	0.30 [-6.76 to 7.36]	0.01
Sweazea et al. 2014	21	0.40 [-0.50 to 1.30]	0.34
Tan and Mattes. 2013 [Afternoon Snack Almonds]	55	0.20 [-3.65 to 4.05]	0.02
Tan and Mattes. 2013 [Breakfast Almonds]	55	0.20 [-3.56 to 3.96]	0.02
Tan and Mattes. 2013 [Lunch Almonds]	53	0.20 [-3.44 to 3.84]	0.02
Tan and Mattes. 2013 [Morning Snack Almonds]	55	0.10 [-3.71 to 3.91]	0.02
Tapsell et al. 2004 [Low Fat]	28	-0.66 [-3.49 to 2.17]	0.03
Tapsell et al. 2004 [Modified Fat]	27	-0.35 [-3.57 to 2.87]	0.03
Tapsell et al. 2017 [Control]	96	-1.20 [-3.97 to 1.57]	0.04
Tapsell et al. 2017 [Intervention Alone]	80	-0.70 [-3.41 to 2.01]	0.04
Tey et al. 2011 [Chocolate]	63	0.07 [-0.31 to 0.45]	1.88
Tey et al. 2011 [Control]	61	0.14 [-0.18 to 0.46]	2.67
Tey et al. 2011 [Potato crisp]	58	0.13 [-0.21 to 0.47]	2.34

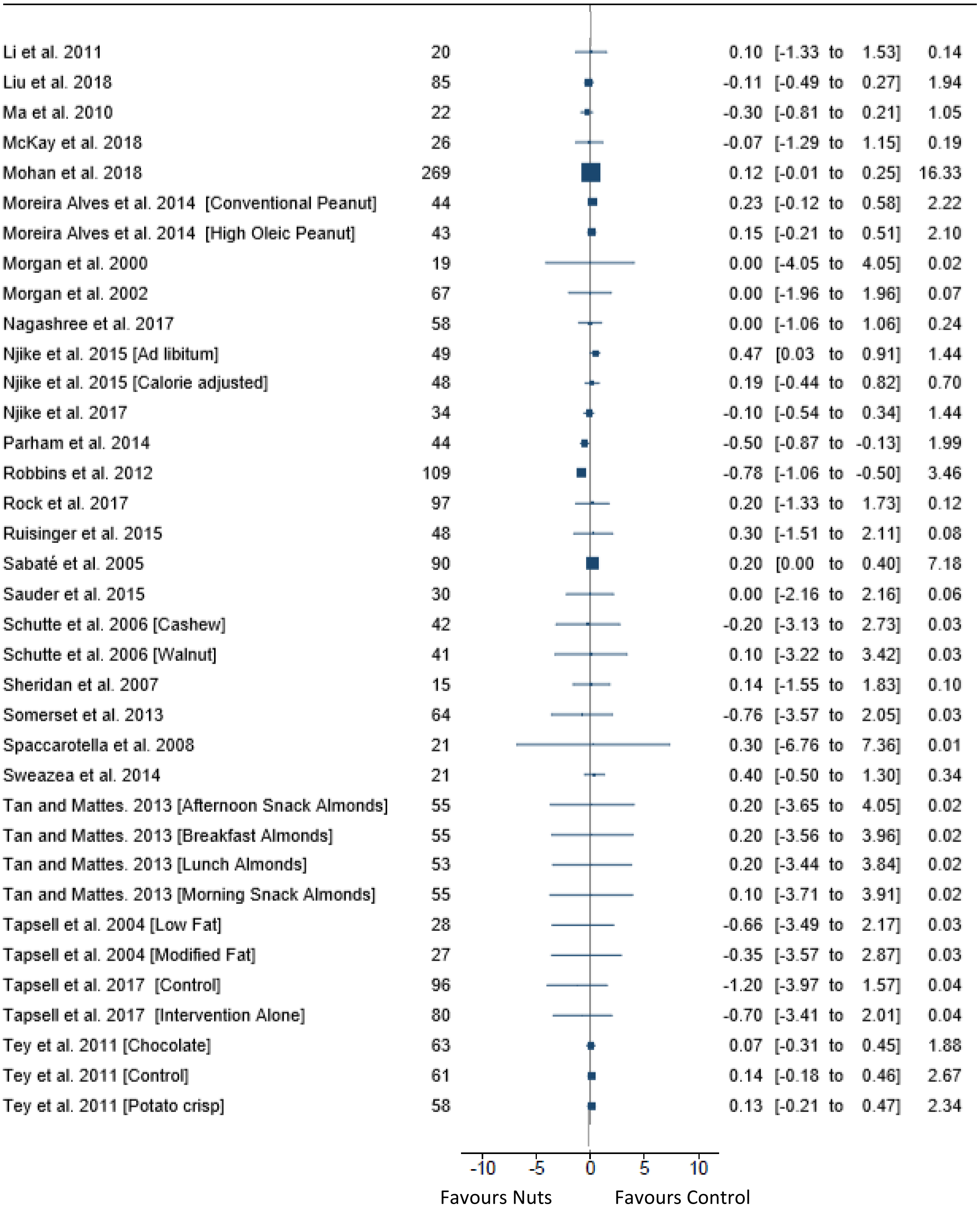

Supplementary Figure 25. Forest plot of randomized controlled trials investigating the effects of nut consumption on body mass index (BMI) (kg/m^2) with the use of a fixed-effects model.

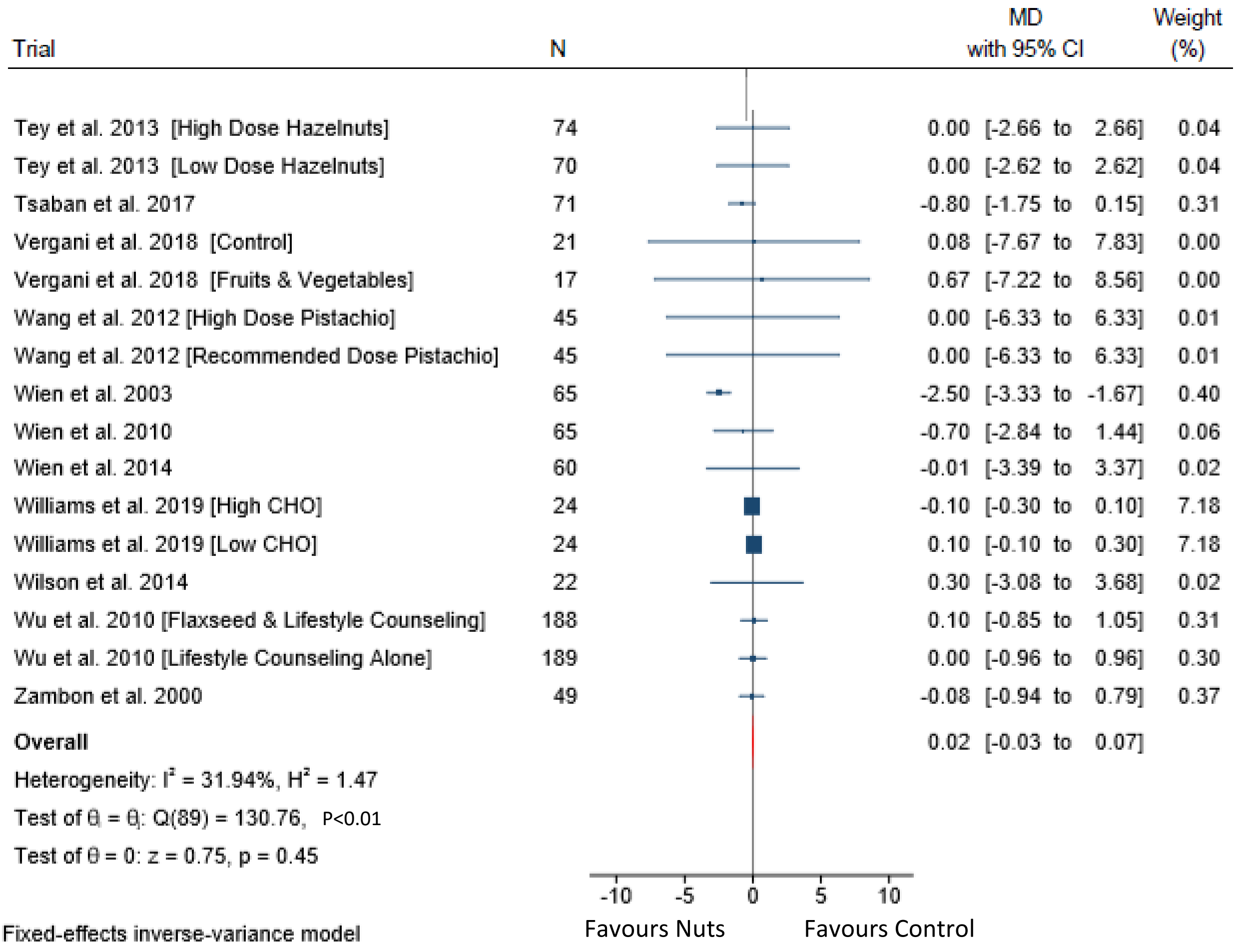

Pooled effect estimate is represented by the diamond and was estimated with the use of fixed effects inverse-variance model. To avoid unit of analysis error, standard error, used for determining the 95% confidence interval, was calculated by splitting the N for studies with multiple comparisons as per the Cochrane Handbook, 2019.
CI, confidence interval; MD, mean difference; N, number of participants.

Supplementary Figure 26. Forest plot of randomized controlled trials investigating the effects of nut consumption on body fat (%) with the use of a fixed-effects model (continued on the next page).

Trial	N	MD with 95% CI	Weight (%)
Abbaspour et al. 2019	48	0.00 [-4.95 to 4.95]	0.05
Barbour et al. 2015	61	0.30 [-0.09 to 0.69]	8.73
Bento et al. 2014	20	-0.90 [-5.37 to 3.57]	0.07
Berryman et al. 2015	48	-0.15 [-0.52 to 0.21]	10.03
Bitok et al. 2018	317	0.41 [0.05 to 0.77]	10.47
Biude Silva Duarte et al. 2019	55	0.40 [-1.00 to 1.80]	0.68
Bowen et al. 2019	76	-0.87 [-1.81 to 0.07]	1.52
Carughi et al. 2019	60	-0.50 [-3.02 to 2.02]	0.21
Casas-Agustench et al. 2011	50	-0.60 [-1.77 to 0.57]	0.98
Cohen et al. 2011	13	0.10 [-10.32 to 10.52]	0.01
Dhillon et al. 2018	73	0.20 [-4.08 to 4.48]	0.07
Foster et al. 2012	123	-0.04 [-0.51 to 0.43]	6.11
Hollis and Mattes. 2007	20	1.30 [-1.92 to 4.52]	0.13
Jenkins et al. 2002 [Full Dose Almonds]	27	-1.10 [-5.44 to 3.24]	0.07
Jenkins et al. 2002 [Half Dose Almonds]	27	-0.10 [-4.42 to 4.22]	0.07
Johnston et al. 2013	44	0.30 [-0.68 to 1.28]	1.40
Jung et al. 2018	84	-0.20 [-0.42 to 0.02]	27.92
Li et al. 2011	20	-0.80 [-4.99 to 3.39]	0.08
Liu et al. 2018	85	-0.74 [-1.89 to 0.41]	1.02
Moreira Alves et al. 2014 [Conventional Peanut]	44	-0.58 [-1.60 to 0.44]	1.28
Moreira Alves et al. 2014 [High Oleic Peanut]	43	-0.91 [-1.90 to 0.08]	1.38
Njike et al. 2015 [Ad libitum]	49	1.14 [-0.85 to 3.13]	0.34
Njike et al. 2015 [Calorie adjusted]	48	-0.19 [-1.38 to 1.00]	0.95
Njike et al. 2017	34	-7.90 [-14.71 to -1.09]	0.03
Sabaté et al. 2005	90	0.10 [-0.19 to 0.39]	15.80
Somerset et al. 2013	64	3.98 [2.89 to 5.07]	1.13
Sweazea et al. 2014	21	2.80 [-0.95 to 6.55]	0.10
Tan and Mattes. 2013 [Afternoon Snack Almonds]	55	-0.28 [-4.29 to 3.74]	0.08
Tan and Mattes. 2013 [Breakfast Almonds]	55	-0.08 [-4.10 to 3.94]	0.08
Tan and Mattes. 2013 [Lunch Almonds]	53	-0.34 [-4.33 to 3.65]	0.08
Tan and Mattes. 2013 [Morning Snack Almonds]	55	0.13 [-3.88 to 4.14]	0.08
Tapsell et al. 2004 [Low Fat]	28	-1.64 [-8.85 to 5.57]	0.03
Tapsell et al. 2004 [Modified Fat]	27	-0.86 [-8.59 to 6.87]	0.02
Tapsell et al. 2009	35	-0.60 [-5.73 to 4.53]	0.05

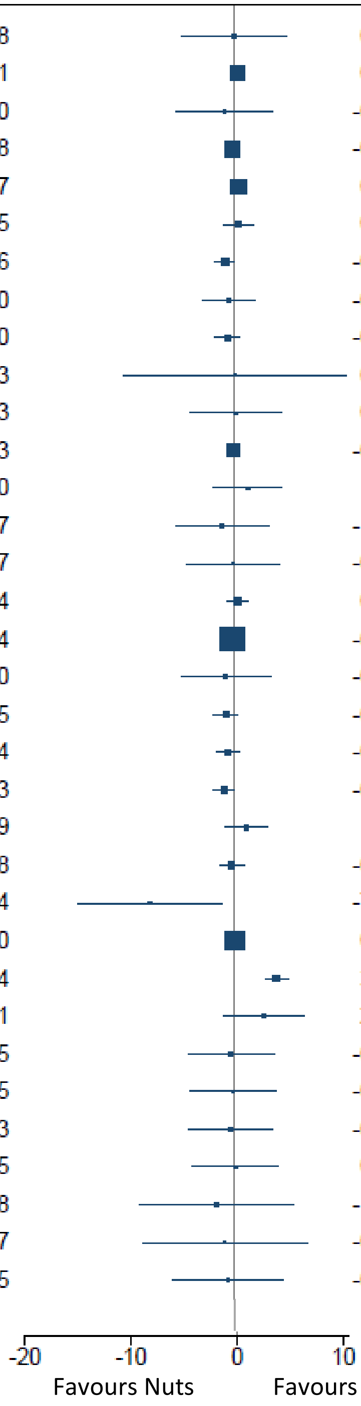

Supplementary Figure 26. Forest plot of randomized controlled trials investigating the effects of nut consumption on body fat (%) with the use of a fixed-effects model.

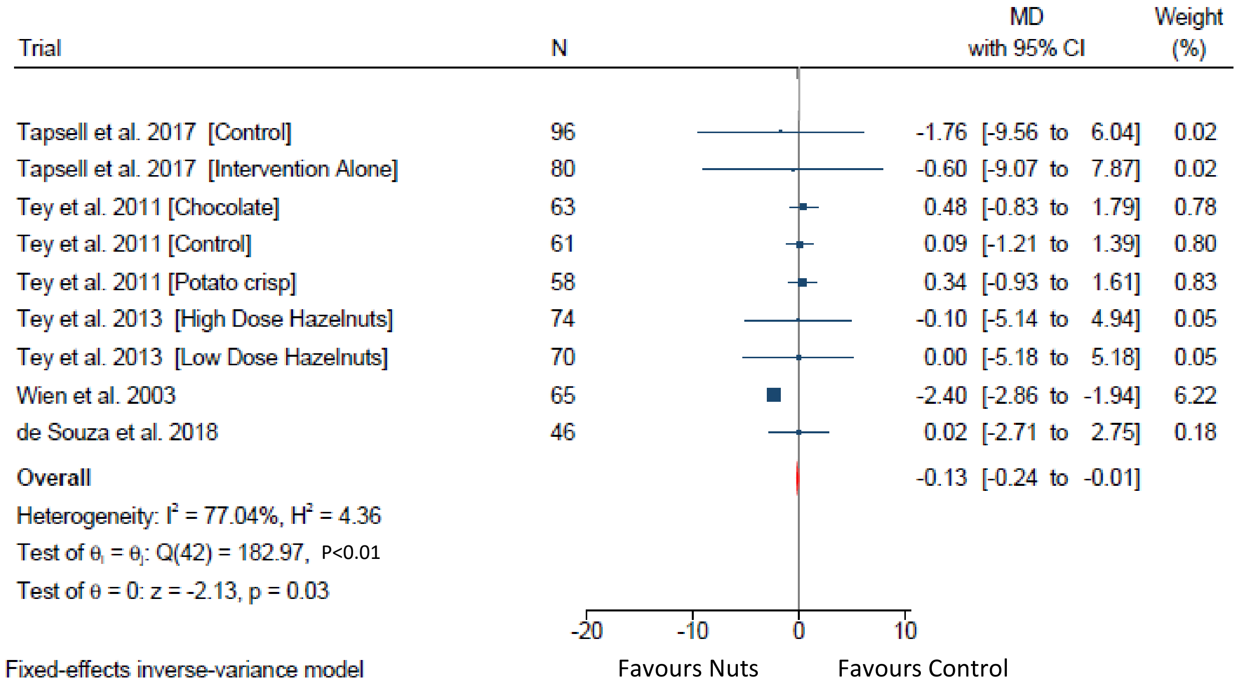

Pooled effect estimate is represented by the diamond and was estimated with the use of fixed effects inverse-variance model. To avoid unit of analysis error, standard error, used for determining the 95% confidence interval, was calculated by splitting the N for studies with multiple comparisons as per the Cochrane Handbook, 2019.

CI, confidence interval; MD, mean difference; N, number of participants.

Supplementary Figure 27. Forest plot of randomized controlled trials investigating the effects of nut consumption on waist circumference (cm) with the use of a fixed-effects model (continued on the next page).

Trial	N	MD with 95% CI	Weight (%)
Abazarfard et al. 2014	100	-8.72 [-11.15 to -6.29]	0.00
Abbaspour et al. 2019	48	-0.30 [-1.46 to 0.86]	0.02
Balci et al. 2012	60	0.10 [-5.83 to 6.03]	0.00
Bamberger et al. 2017	204	-0.10 [-1.69 to 1.49]	0.01
Barbour et al. 2015	61	0.30 [-2.55 to 3.15]	0.00
Berryman et al. 2015	48	-0.80 [-3.15 to 1.55]	0.00
Bitok et al. 2018	317	0.30 [-2.76 to 3.36]	0.00
Biude Silva Duarte et al. 2019	55	-3.60 [-5.75 to -1.45]	0.01
Bowen et al. 2019	76	-0.47 [-1.62 to 0.68]	0.02
Campbell et al. 2019	12	-7.20 [-19.13 to 4.73]	0.00
Carughi et al. 2019	60	-0.50 [-3.58 to 2.58]	0.00
Casas-Agustench et al. 2011	50	-0.40 [-2.99 to 2.19]	0.00
Ciccone et al. 2014 [Control]	31	-4.10 [-18.69 to 10.49]	0.00
Ciccone et al. 2014 [Non-fried fish]	15	-1.40 [-17.38 to 14.58]	0.00
Ciccone et al. 2014 [Olive Oil]	15	-5.80 [-24.75 to 13.15]	0.00
Damavandi et al. 2012	43	-0.80 [-6.90 to 5.30]	0.00
DeSouza et al. 2018	46	-2.45 [-8.93 to 4.03]	0.00
Dhillon et al. 2016	86	-1.79 [-6.71 to 3.13]	0.00
Dhillon et al. 2018	73	0.20 [0.15 to 0.25]	12.37
Gulati et al. 2014	68	1.80 [-1.66 to 5.26]	0.00
Hernández-Alonso et al. 2014	49	1.07 [0.29 to 1.85]	0.04
Hwang et al. 2019	84	1.66 [-0.24 to 3.56]	0.01
Jenkins et al. 2002 [Full Dose Almonds]	27	-1.30 [-7.75 to 5.15]	0.00
Jenkins et al. 2002 [Half Dose Almonds]	27	-2.00 [-8.45 to 4.45]	0.00
Jenkins et al. 2018 [Full Dose Nuts]	100	0.00 [-0.02 to 0.02]	43.53
Jenkins et al. 2018 [Half Dose Nuts]	100	0.00 [-0.02 to 0.02]	43.59
Johnston et al. 2013	44	-2.60 [-5.51 to 0.31]	0.00
Jung et al. 2018	84	0.10 [-1.16 to 1.36]	0.02
Katz et al. 2012	46	-0.20 [-1.56 to 1.16]	0.01
Lee et al. 2014	60	-0.62 [-1.27 to 0.03]	0.06
Liu et al. 2018	85	-0.86 [-2.66 to 0.94]	0.01
Ma et al. 2010	22	-0.30 [-2.55 to 1.95]	0.01
Mohan et al. 2018	269	-0.12 [-0.70 to 0.46]	0.08
Moreira Alves et al. 2014 [Conventional Peanut]	44	0.06 [-1.08 to 1.20]	0.02
Moreira Alves et al. 2014 [High Oleic Peanut]	43	0.48 [-0.59 to 1.55]	0.02

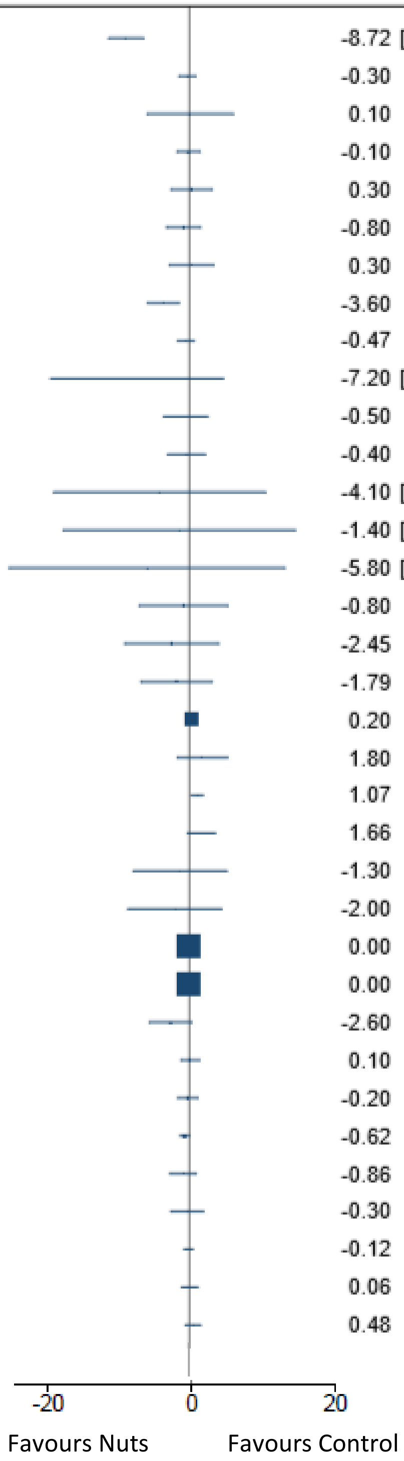

Supplementary Figure 27. Forest plot of randomized controlled trials investigating the effects of nut consumption on waist circumference (cm) with the use of a fixed-effects model.

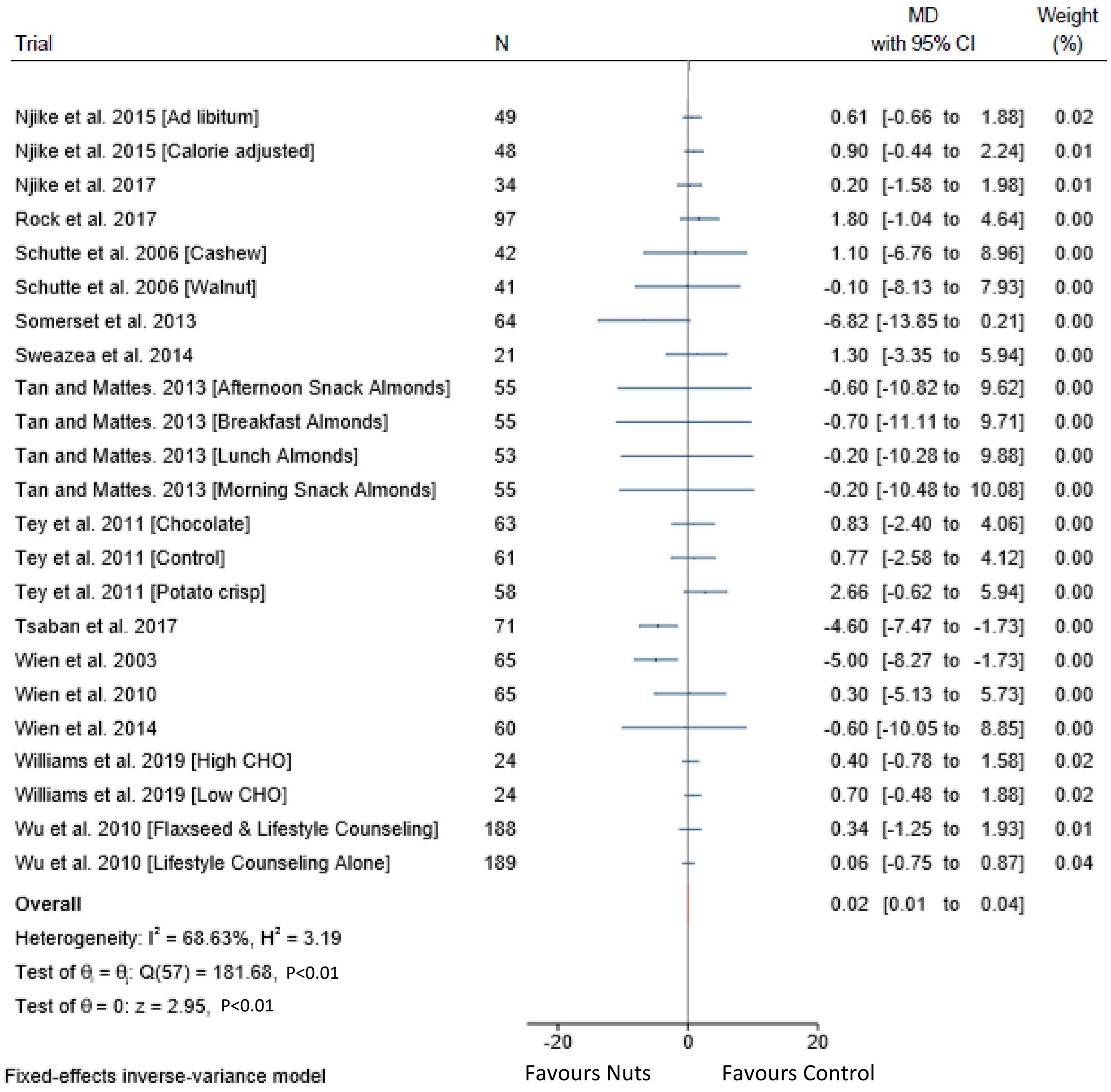

Pooled effect estimate is represented by the diamond and was estimated with the use of fixed effects inverse-variance model. To avoid unit of analysis error, standard error, used for determining the 95% confidence interval, was calculated by splitting the N for studies with multiple comparisons as per the Cochrane Handbook, 2019.
CI, confidence interval; MD, mean difference; N, number of participants.

Supplementary Figure 28. Forest plot of randomized controlled trials investigating the effects of nut consumption on waist-to-up ratio with the use of a fixed-effects model.

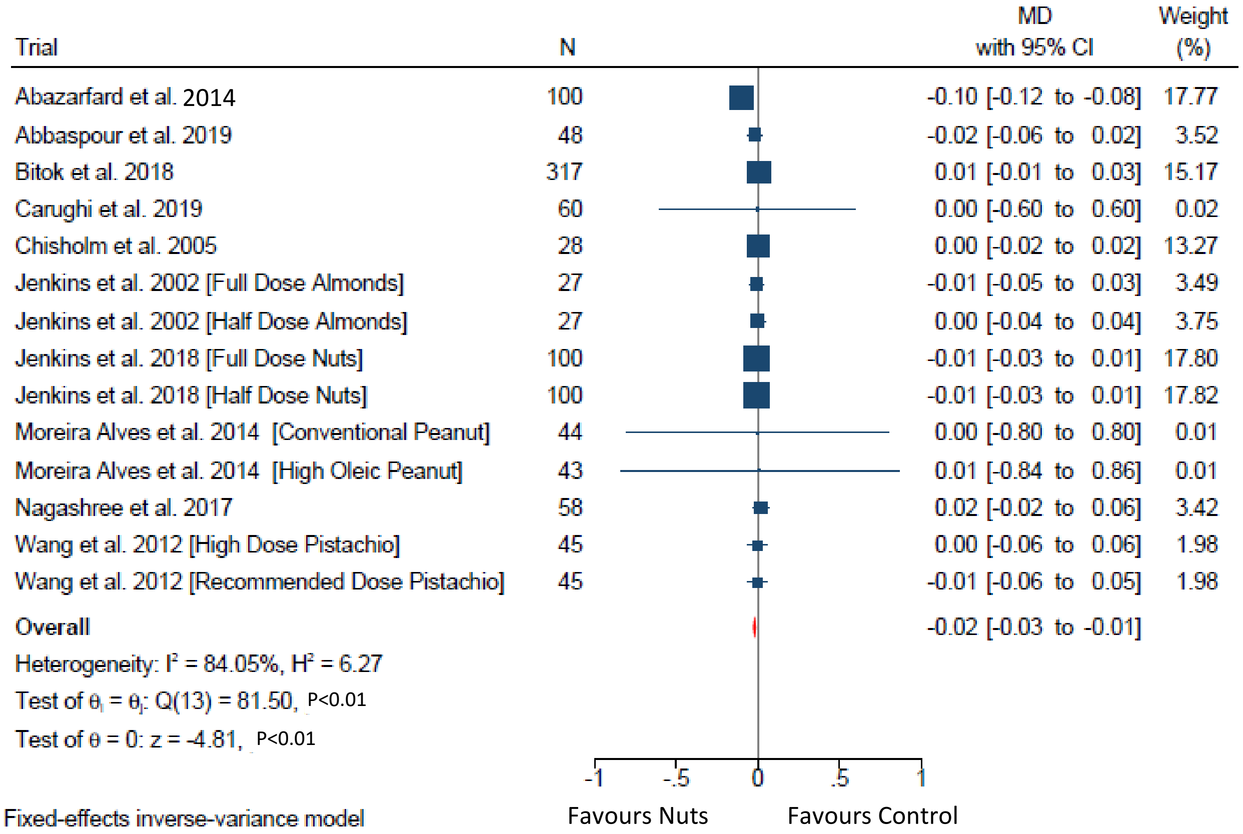

Pooled effect estimate is represented by the diamond and was estimated with the use of fixed effects inverse-variance model.
To avoid unit of analysis error, standard error, used for determining the 95% confidence interval, was calculated by splitting the N for studies with multiple comparisons as per the Cochrane Handbook, 2019.
CI, confidence interval; MD, mean difference; N, number of participants.

Supplementary Figure 29. Forest plot of randomized controlled trials investigating the effects of nut consumption on visceral adipose tissue with the use of a fixed-effects model.

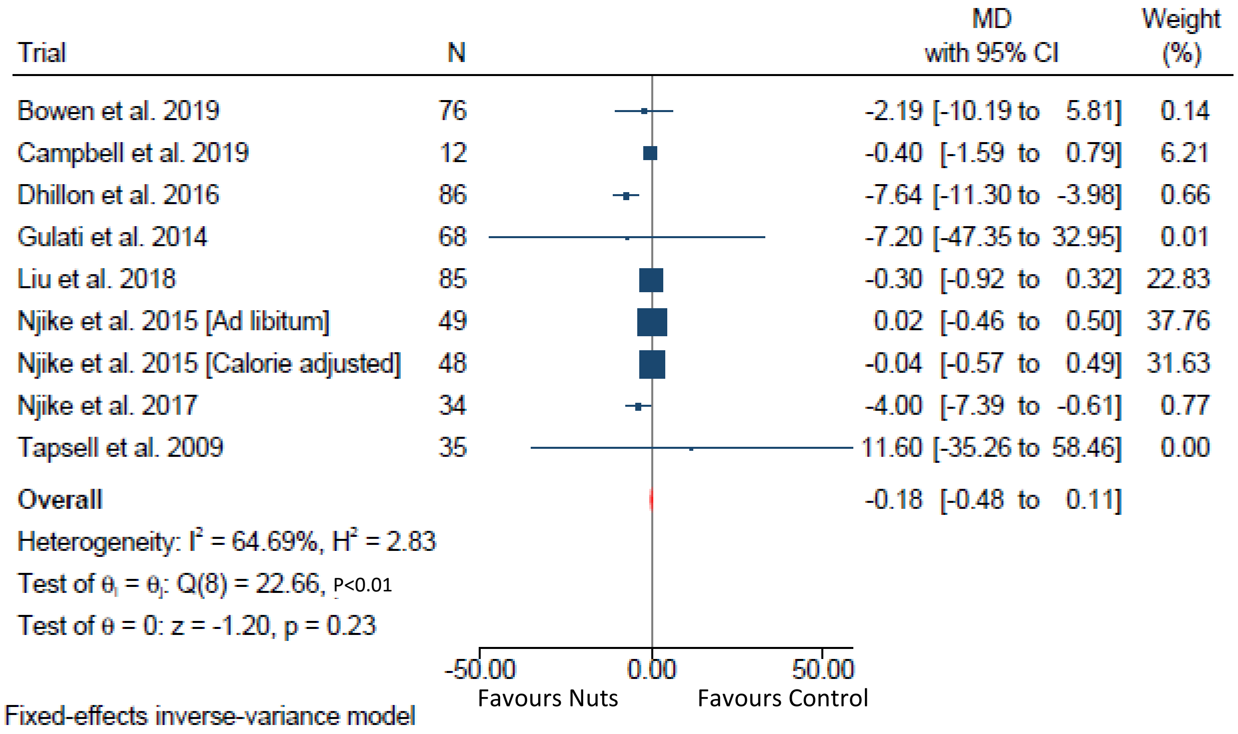

Pooled effect estimate is represented by the diamond and was estimated with the use of fixed effects inverse-variance model. To avoid unit of analysis error, standard error, used for determining the 95% confidence interval, was calculated by splitting the N for studies with multiple comparisons as per the Cochrane Handbook, 2019.

CI, confidence interval; MD, mean difference; N, number of participants.

Supplementary Figure 30. Risk of bias (using The Cochrane Collaboration Tool) subgroup analysis for the effect of nut consumption on body weight (kg).

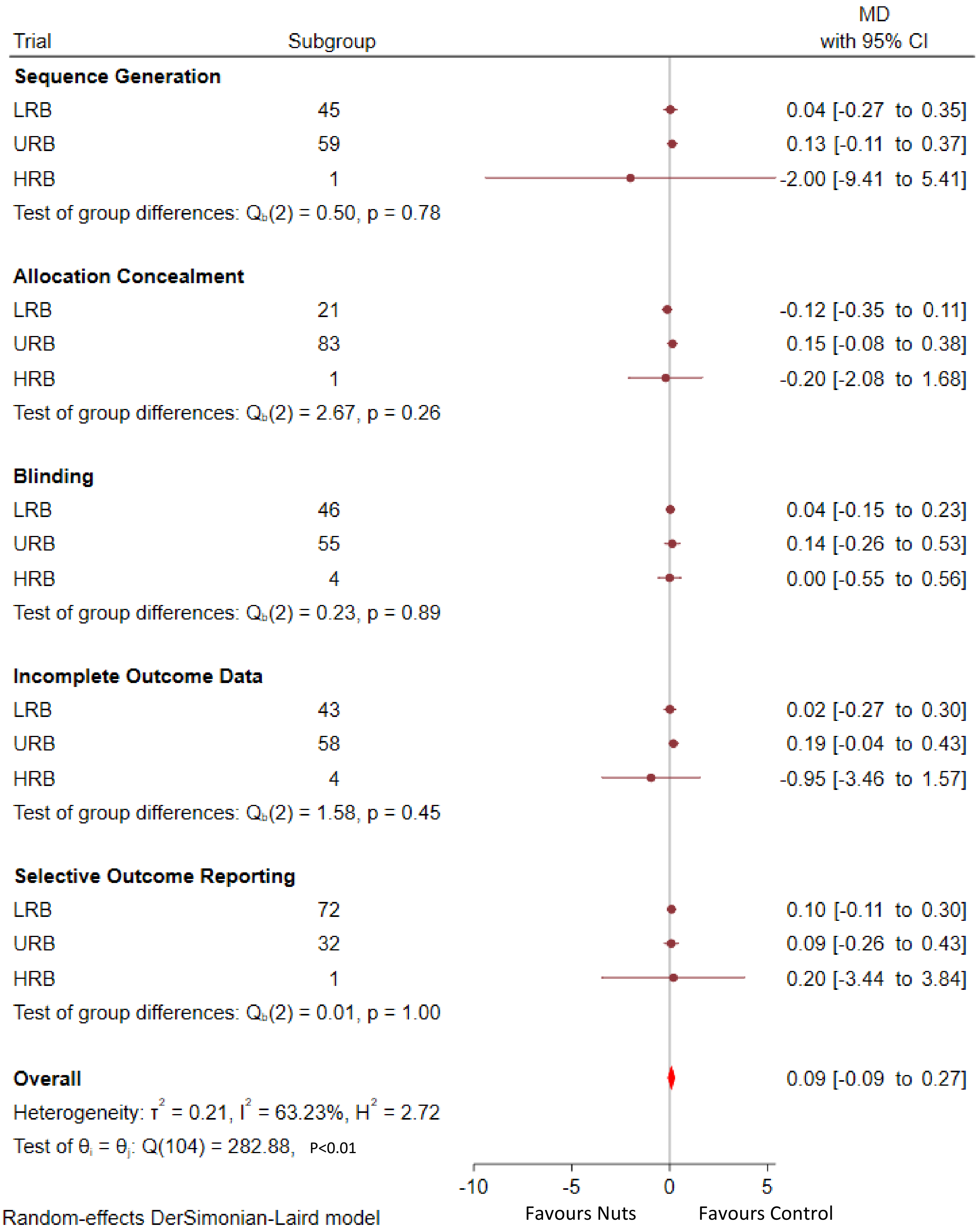

LRB, low risk of bias; URB, unclear risk of bias; HRB, high risk of bias.

Supplementary Figure 31. Risk of bias (using The Cochrane Collaboration Tool) subgroup analysis for the effect of nut consumption on BMI (kg/m^2).

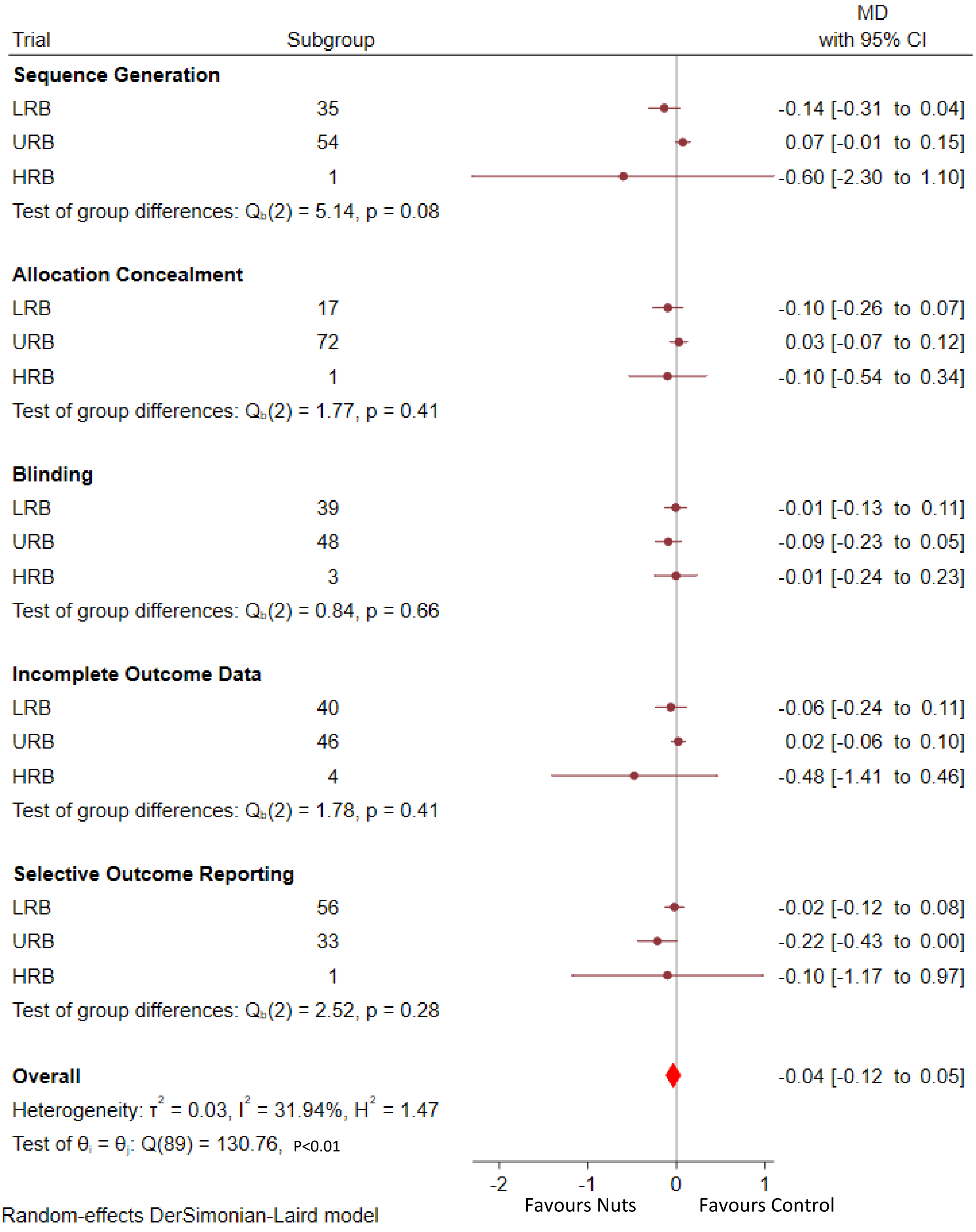

LRB, low risk of bias; URB, unclear risk of bias; HRB, high risk of bias.

Supplementary Figure 32. Risk of bias (using The Cochrane Collaboration Tool) subgroup analysis for the effect of nut consumption on body fat (%) (continued on next page).

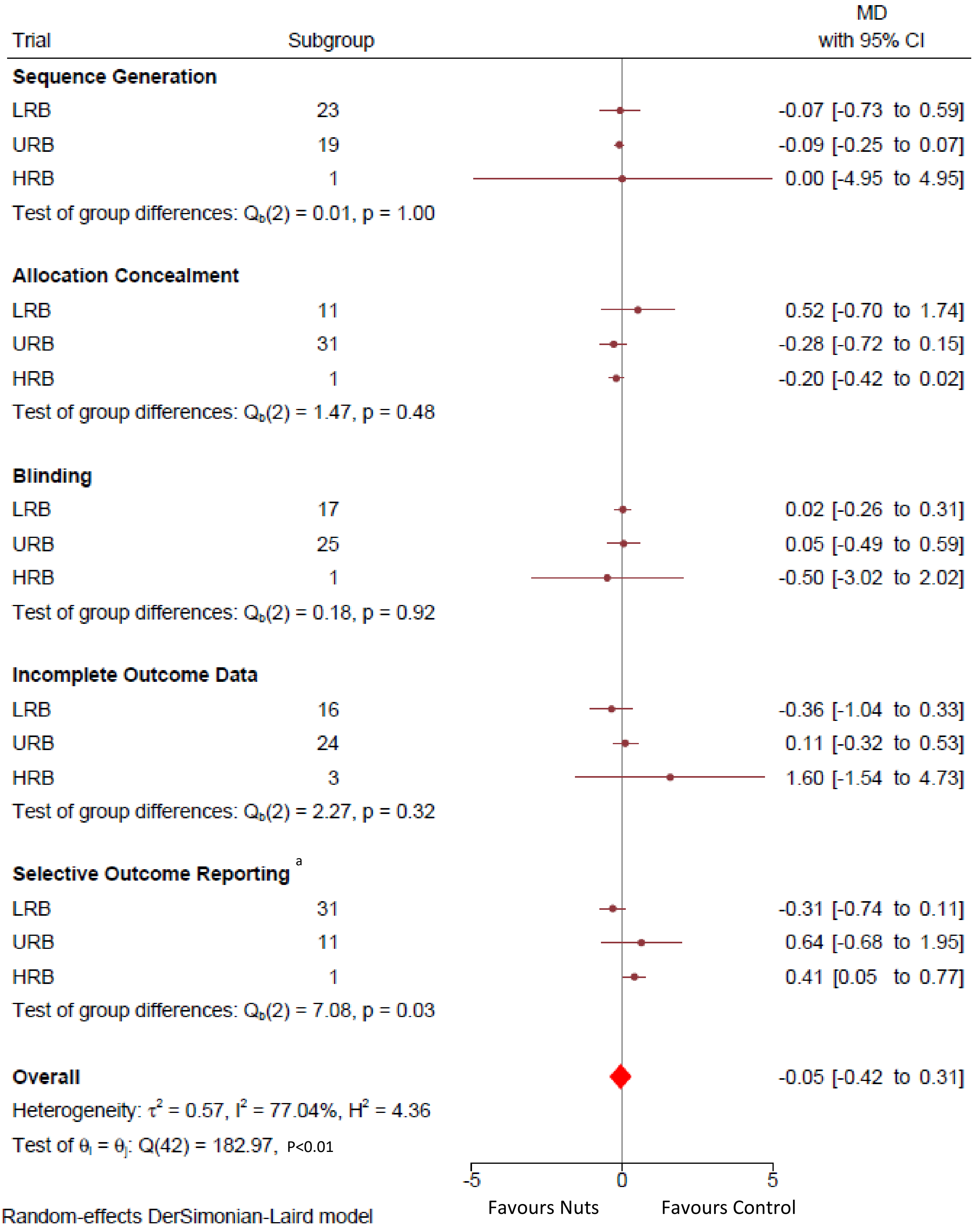

Supplementary Figure 32. Risk of bias (using The Cochrane Collaboration Tool) subgroup analysis for the effect of nut consumption on body fat (%).

LRB, low risk of bias; URB, unclear risk of bias; HRB, high risk of bias.

[a]Pairwise between-subgroup mean differences (95% CIs) for Selective Outcome Reporting were as follows:-0.72% (-2.51, 1.08%0 (LRB vs. HRB) to 0.36% (-1.59 to 2.30%) (URB vs. HRB) to 1.07% (0.08, 2.07%) (URB vs. LRB).

Supplementary Figure 33. Risk of bias (using The Cochrane Collaboration Tool) subgroup analysis for the effect of nut consumption on waist circumference (cm) (continued on next page).

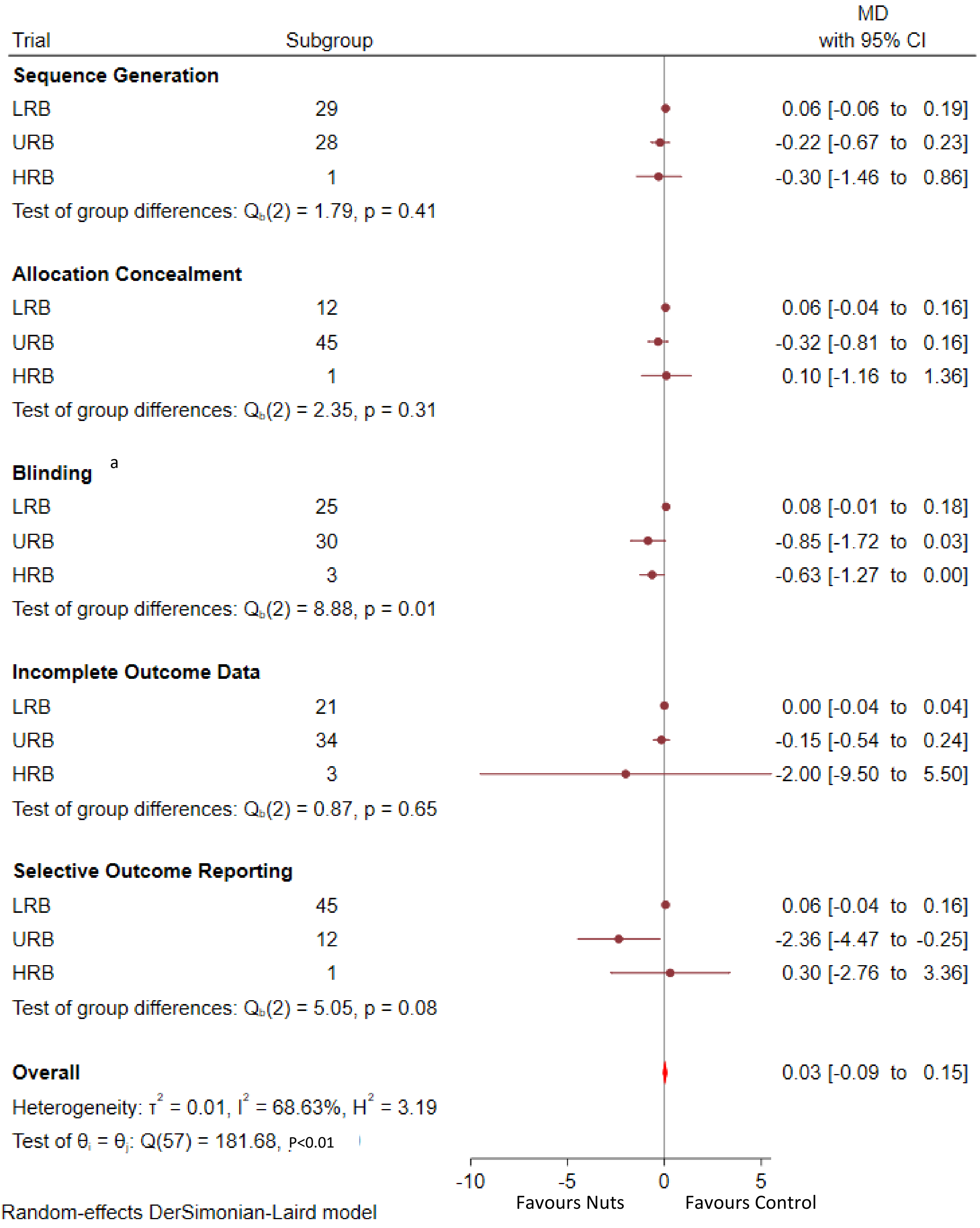

Supplementary Figure 33. Risk of bias (using The Cochrane Collaboration Tool) subgroup analysis for the effect of nut consumption on waist circumference (cm).

LRB, low risk of bias; URB, unclear risk of bias; HRB, high risk of bias.
[a]Pairwise between-subgroup mean differences (95% CIs) for Blinding were as follows: 0.73 cm (0.04, 1.41 cm) (LRB vs. HRB) to 0.26 cm (-0.51, 1.03 cm) (URB vs. HRB) to -0.47 cm (-0.87, -0.07 cm) (URB vs. LRB).

Supplementary Figure 34. Risk of bias (using The Cochrane Collaboration Tool) subgroup analysis for the effect of nut consumption on waist-to-hip ratio.

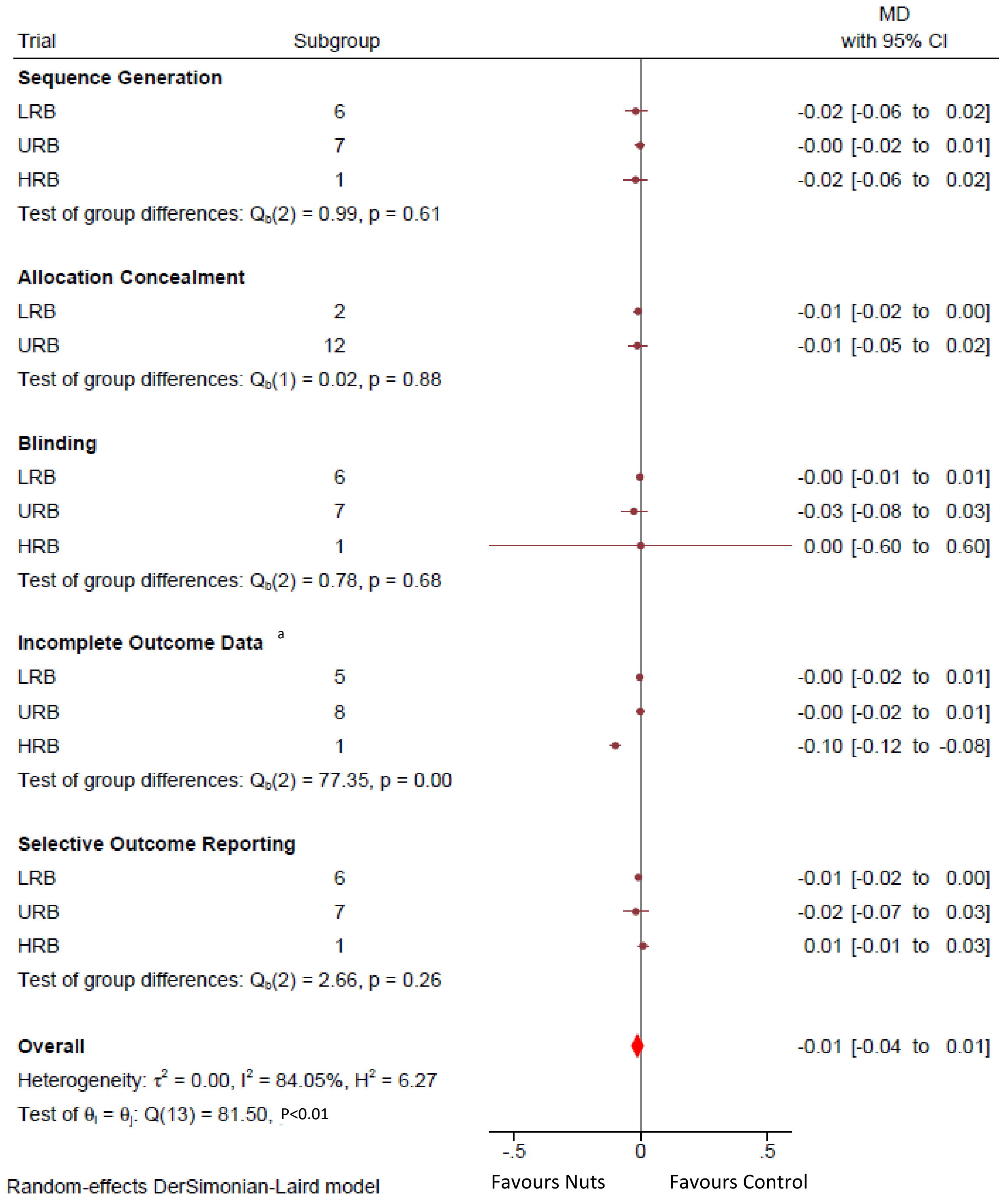

LRB, low risk of bias; URB, unclear risk of bias; HRB, high risk of bias.

[a]Pairwise between-subgroup mean differences (95% CIs) for Incomplete Outcome Data were as follows: 0.10 (0.01, 0.18) (LRB vs. HRB) to 0.10 (0.01, 0.19) (URB vs. HRB) to 0.001 (-0.05, 0.05) (URB vs. LRB).

Supplementary Figure 35. Funnel plot for the effect of nut consumption on adiposity measures.

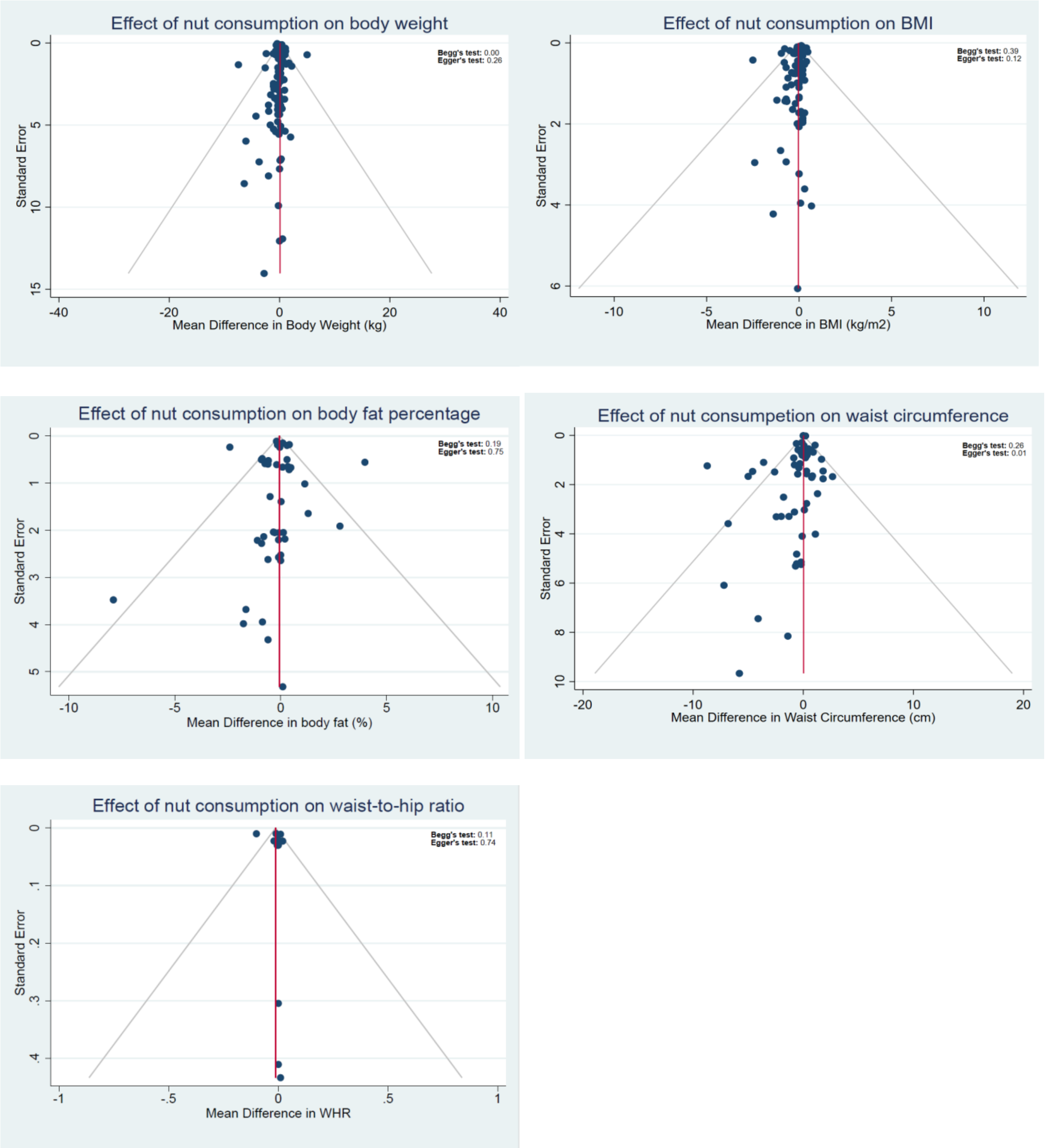

The vertical line represents the pooled effect estimate expressed as mean difference. The diagonal lines represent the pseudo 95% confidence limits, and the circles represent effect estimates for each included trial. P-values were derived from quantitative assessment of publication bias by Egger's and Begg's tests set at a significance level of $p<0.05$. Note publication bias could not be performed for the outcome visceral adipose tissue as there were too few trial comparisons.

Supplementary Figure 36. Trim-and-Fill analysis for the effect of nut consumption on adiposity measures.

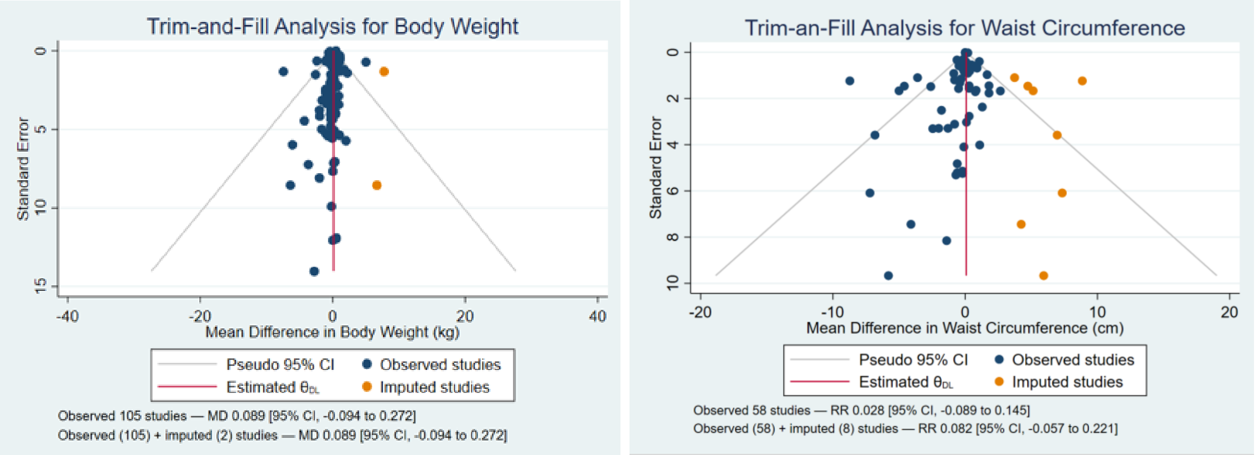

The vertical line represents the pooled effect estimate expressed as mean difference. The diagonal lines represent the pseudo 95% confidence limits, the dark coloured circles represent the effect estimate for each included trial, and the light coloured circles represent the effect estimate for each imputed "missed" trial. Imputed random mean difference is provided, $p<0.05$ is considered evidence of small-trial effects.

REFERENCES

1. Stroup DF, Berlin JA, Morton SC, et al. Meta-analysis of observational studies in epidemiology: a proposal for reporting. Meta-analysis Of Observational Studies in Epidemiology (MOOSE) group. Jama. 2000;283(15):2008-12.
2. Moher D, Liberati A, Tetzlaff J, Altman DG. Preferred reporting items for systematic reviews and meta-analyses: the PRISMA statement. PLoS medicine. 2009;6(7):e1000097.
3. Moher D, Shamseer L, Clarke M, et al. Preferred reporting items for systematic review and meta-analysis protocols (PRISMA-P) 2015 statement. Systematic reviews. 2015;4:1.
4. Bes-Rastrollo M, Sabate J, Gomez-Gracia E, Alonso A, Martinez JA, Martinez-Gonzalez MA. Nut consumption and weight gain in a Mediterranean cohort: The SUN study. Obesity (Silver Spring, Md). 2007;15(1):107-16.
5. El-Amari SS, Lloren JI, Sabate J. Nut intake, prospective weight change, and obesity risk: The adventist health study-2. FASEB Journal Conference: Experimental Biology. 2016;30(Meeting Abstracts).
6. Freisling H, Noh H, Slimani N, et al. Nut intake and 5-year changes in body weight and obesity risk in adults: results from the EPIC-PANACEA study. European journal of nutrition. 2018;57(7):2399-408.
7. Liu X, Li Y, Guasch-Ferré M, Willett WC, et al. Changes in nut consumption influence long-term weight change in US men and women. BMJ Nutrition, Prevention & Health. 2019:bmjnph-2019-000034.
8. Smith JD, Hou T, Hu FB, et al. A Comparison of Different Methods for Evaluating Diet, Physical Activity, and Long-Term Weight Gain in 3 Prospective Cohort Studies. The Journal of nutrition. 2015;145(11):2527-34.
9. Fernandez-Montero A, Bes-Rastrollo M, Beunza JJ, et al. Nut consumption and incidence of metabolic syndrome after 6-year follow-up: the SUN (Seguimiento Universidad de Navarra, University of Navarra Follow-up) cohort. Public health nutrition. 2013;16(11):2064-72.
10. Abazarfard Z, Salehi M, Keshavarzi S. The effect of almonds on anthropometric measurements and lipid profile in overweight and obese females in a weight reduction program: A randomized controlled clinical trial. Journal of research in medical sciences : the official journal of Isfahan University of Medical Sciences. 2014;19(5):457-64.
11. Abbaspour N, Roberts T, Hooshmand S, Kern M, Hong MY. Mixed Nut Consumption May Improve Cardiovascular Disease Risk Factors in Overweight and Obese Adults. Nutrients. 2019;11(7).
12. Agebratt C, Strom E, Romu T, et al. A Randomized Study of the Effects of Additional Fruit and Nuts Consumption on Hepatic Fat Content, Cardiovascular Risk Factors and Basal Metabolic Rate. PloS one. 2016;11(1):e0147149.
13. Baer DJ, Novotny JA. Consumption of cashew nuts does not influence blood lipids or other markers of cardiovascular disease in humans: a randomized controlled trial. The American journal of clinical nutrition. 2019;109(2):269-75.
14. Balci MK, Balci B, Hoda P. Metabolic effects of walnuts in patients with prediabetic metabolic syndrome. Endocrine Reviews Conference: 94th Annual Meeting and Expo of the Endocrine Society, ENDO. 2012;33(3 MeetingAbstracts).

15. Barbour JA, Howe PR, Buckley JD, Bryan J, Coates AM. Effect of 12 Weeks High Oleic Peanut Consumption on Cardio-Metabolic Risk Factors and Body Composition. Nutrients. 2015;7(9):7381-98.
16. Bento AP, Cominetti C, Simoes Filho A, Naves MM. Baru almond improves lipid profile in mildly hypercholesterolemic subjects: a randomized, controlled, crossover study. Nutrition, metabolism, and cardiovascular diseases : NMCD. 2014;24(12):1330-6.
17. Berryman CE, West SG, Fleming JA, Bordi PL, Kris-Etherton PM. Effects of daily almond consumption on cardiometabolic risk and abdominal adiposity in healthy adults with elevated LDL-cholesterol: a randomized controlled trial. Journal of the American Heart Association. 2015;4(1):e000993.
18. Bitok E, Rajaram S, Jaceldo-Siegl K, et al. Effects of Long-Term Walnut Supplementation on Body Weight in Free-Living Elderly: Results of a Randomized Controlled Trial. Nutrients. 2018;10(9).
19. Biude Silva Duarte G, Zavarize Reis B, Cercato C, Macedo Rogero M, Maria Franciscato Cozzolino S. Brazilian nut intake has no influence on body composition in obese women. Annals of Nutrition and Metabolism. 2017;71 (Supplement 2):1142.
20. Bowen J, Luscombe-Marsh ND, Stonehouse W, et al. Effects of almond consumption on metabolic function and liver fat in overweight and obese adults with elevated fasting blood glucose: A randomised controlled trial. Clinical nutrition ESPEN. 2019;30:10-8.
21. Campbell A, Roychoudhury A, St-Onge MP. Almond consumption increases satiety hormones relative to a high-carbohydrate food but has minimal impact on body composition: A pilot study in black and hispanic adults. Circulation Conference: American Heart Association's Epidemiology and Prevention/Lifestyle and Cardiometabolic Health. 2019;139(Supplement 1).
22. Canales A, Benedi J, Nus M, Librelotto J, Sanchez-Montero JM, Sanchez-Muniz FJ. Effect of walnut-enriched restructured meat in the antioxidant status of overweight/obese senior subjects with at least one extra CHD-risk factor. Journal of the American College of Nutrition. 2007;26(3):225-32.
23. Carughi A, Bellisle F, Dougkas A, Giboreau A, Feeney MJ, Higgs J. A Randomized Controlled Pilot Study to Assess Effects of a Daily Pistachio (Pistacia Vera) Afternoon Snack on Next-Meal Energy Intake, Satiety, and Anthropometry in French Women. Nutrients. 2019;11(4).
24. Casas-Agustench P, Lopez-Uriarte P, Bullo M, Ros E, Cabre-Vila JJ, Salas-Salvado J. Effects of one serving of mixed nuts on serum lipids, insulin resistance and inflammatory markers in patients with the metabolic syndrome. Nutrition, metabolism, and cardiovascular diseases : NMCD. 2011;21(2):126-35.
25. Chisholm A, Mc Auley K, Mann J, Williams S, Skeaff M. Cholesterol lowering effects of nuts compared with a Canola oil enriched cereal of similar fat composition. Nutrition, metabolism, and cardiovascular diseases : NMCD. 2005;15(4):284-92.
26. Ciccone MM SP, Cortese F, Gesualdo M, Fornarelli F, Sassara M, De Santis L, Zito A, Riccardi R, Lovero MC, Ghiadoni L, Lagioia R, Scrutinio D, De Pergola. Endothelial function in ovese and overweight patients: The role of olive oil, fish and nuts. International Journal of Diabetes and Clinical Research. 2014;1(1):1-5.
27. Cohen AE, Johnston CS. Almond ingestion at mealtime reduces postprandial glycemia and chronic ingestion reduces hemoglobin A(1c) in individuals with well-controlled type 2 diabetes mellitus. Metabolism: clinical and experimental. 2011;60(9):1312-7.

28. Damasceno NR, Perez-Heras A, Serra M, et al. Crossover study of diets enriched with virgin olive oil, walnuts or almonds. Effects on lipids and other cardiovascular risk markers. Nutrition, metabolism, and cardiovascular diseases : NMCD. 2011;21 Suppl 1:S14-20.
29. Damavandi RD, Shidfar F, Rajab A, Mohammadi V, Hosseini S. The effects of cashew consumption on serum glucose, insulin and lipoprotein in type 2 diabetic patients. Iranian Journal of Endocrinology and Metabolism. 2012;14(4):Pe325-Pe34, En413.
30. Damavandi RD, Eghtesadi S, Shidfar F, Heydari I, Foroushani AR. Effects of hazelnuts consumption on fasting blood sugar and lipoproteins in patients with type 2 diabetes. Journal of research in medical sciences : the official journal of Isfahan University of Medical Sciences. 2013;18(4):314-21.
31. de Souza RGM, Gomes AC, de Castro IA, Mota JF. A baru almond-enriched diet reduces abdominal adiposity and improves high-density lipoprotein concentrations: a randomized, placebo-controlled trial. Nutrition (Burbank, Los Angeles County, Calif). 2018;55-56:154-60.
32. Dhillon J, Tan SY, Mattes RD. Almond Consumption during Energy Restriction Lowers Truncal Fat and Blood Pressure in Compliant Overweight or Obese Adults. The Journal of nutrition. 2016;146(12):2513-9.
33. Dhillon J, Thorwald M, De La Cruz N, et al. Glucoregulatory and Cardiometabolic Profiles of Almond vs. Cracker Snacking for 8 Weeks in Young Adults: A Randomized Controlled Trial. Nutrients. 2018;10(8).
34. Estruch R, Martinez-Gonzalez MA, Corella D, et al. Effect of a high-fat Mediterranean diet on bodyweight and waist circumference: a prespecified secondary outcomes analysis of the PREDIMED randomised controlled trial. The lancet Diabetes & endocrinology. 2019;7(5):e6-e17.
35. Foster GD, Shantz KL, Vander Veur SS, et al. A randomized trial of the effects of an almond-enriched, hypocaloric diet in the treatment of obesity. The American journal of clinical nutrition. 2012;96(2):249-54.
36. Gebauer SK, West SG, Kay CD, Alaupovic P, Bagshaw D, Kris-Etherton PM. Effects of pistachios on cardiovascular disease risk factors and potential mechanisms of action: a dose-response study. The American journal of clinical nutrition. 2008;88(3):651-9.
37. Gulati S, Misra A, Pandey RM, Bhatt SP, Saluja S. Effects of pistachio nuts on body composition, metabolic, inflammatory and oxidative stress parameters in Asian Indians with metabolic syndrome: a 24-wk, randomized control trial. Nutrition (Burbank, Los Angeles County, Calif). 2014;30(2):192-7.
38. Hernandez-Alonso P, Salas-Salvado J, Baldrich-Mora M, Juanola-Falgarona M, Bullo M. Beneficial effect of pistachio consumption on glucose metabolism, insulin resistance, inflammation, and related metabolic risk markers: a randomized clinical trial. Diabetes care. 2014;37(11):3098-105.
39. Hiraoka-Yamamoto J, Ikeda K, Negishi H, et al. Serum lipid effects of a monounsaturated (palmitoleic) fatty acid-rich diet based on macadamia nuts in healthy, young Japanese women. Clinical and experimental pharmacology & physiology. 2004;31 Suppl 2:S37-8.
40. Hollis J, Mattes R. Effect of chronic consumption of almonds on body weight in healthy humans. The British journal of nutrition. 2007;98(3):651-6.
41. Hudthagosol C, Haddad E, Jongsuwat R. Antioxidant activity comparison of walnuts and fatty fish. J Med Assoc Thai. 2012;95 Suppl 6:S179-88.

42. Hwang HJ, Liu Y, Kim HS, Lee H, Lim Y, Park H. Daily walnut intake improves metabolic syndrome status and increases circulating adiponectin levels: randomized controlled crossover trial. Nutrition research and practice. 2019;13(2):105-14.
43. Jamshed H, Sultan FA, Iqbal R, Gilani AH. Dietary Almonds Increase Serum HDL Cholesterol in Coronary Artery Disease Patients in a Randomized Controlled Trial. The Journal of nutrition. 2015;145(10):2287-92.
44. Jenkins DJ, Kendall CW, Marchie A, et al. Dose response of almonds on coronary heart disease risk factors: blood lipids, oxidized low-density lipoproteins, lipoprotein(a), homocysteine, and pulmonary nitric oxide: a randomized, controlled, crossover trial. Circulation. 2002;106(11):1327-32.
45. Jenkins DJA, Kendall CWC, Lamarche B, et al. Nuts as a replacement for carbohydrates in the diabetic diet: a reanalysis of a randomised controlled trial. Diabetologia. 2018;61(8):1734-47.
46. Jenkins DJA, Kendall CWC, Lamarche B, et al. Correction to: Nuts as a replacement for carbohydrates in the diabetic diet: a reanalysis of a randomised controlled trial. Diabetologia. 2019;62(3):549-52.
47. Johnston CS, Trier CM, Fleming KR. The effect of peanut and grain bar preloads on postmeal satiety, glycemia, and weight loss in healthy individuals: an acute and a chronic randomized intervention trial. Nutrition journal. 2013;12:35.
48. Jung H, Chen CO, Blumberg JB, Kwak HK. The effect of almonds on vitamin E status and cardiovascular risk factors in Korean adults: a randomized clinical trial. European journal of nutrition. 2018;57(6):2069-79.
49. Katz DL, Davidhi A, Ma Y, Kavak Y, Bifulco L, Njike VY. Effects of walnuts on endothelial function in overweight adults with visceral obesity: a randomized, controlled, crossover trial. Journal of the American College of Nutrition. 2012;31(6):415-23.
50. Kocyigit A, Koylu AA, Keles H. Effects of pistachio nuts consumption on plasma lipid profile and oxidative status in healthy volunteers. Nutrition, metabolism, and cardiovascular diseases : NMCD. 2006;16(3):202-9.
51. Le T, Flatt SW, Natarajan L, et al. Effects of Diet Composition and Insulin Resistance Status on Plasma Lipid Levels in a Weight Loss Intervention in Women. Journal of the American Heart Association. 2016;5(1).
52. Lee YJ, Nam GE, Seo JA, et al. Nut consumption has favorable effects on lipid profiles of Korean women with metabolic syndrome. Nutrition research (New York, NY). 2014;34(9):814-20.
53. Li Z, Song R, Nguyen C, et al. Pistachio nuts reduce triglycerides and body weight by comparison to refined carbohydrate snack in obese subjects on a 12-week weight loss program. Journal of the American College of Nutrition. 2010;29(3):198-203.
54. Li SC, Liu YH, Liu JF, Chang WH, Chen CM, Chen CY. Almond consumption improved glycemic control and lipid profiles in patients with type 2 diabetes mellitus. Metabolism: clinical and experimental. 2011;60(4):474-9.
55. Liu Y, Hwang HJ, Kim HS, Park H. Time and Intervention Effects of Daily Almond Intake on the Changes of Lipid Profile and Body Composition Among Free-Living Healthy Adults. Journal of medicinal food. 2018;21(4):340-7.
56. Ma Y, Njike VY, Millet J, et al. Effects of walnut consumption on endothelial function in type 2 diabetic subjects: a randomized controlled crossover trial. Diabetes care. 2010;33(2):227-32.

57. McKay DL, Eliasziw M, Chen CYO, Blumberg JB. A Pecan-Rich Diet Improves Cardiometabolic Risk Factors in Overweight and Obese Adults: A Randomized Controlled Trial. Nutrients. 2018;10(3).
58. Mohan V, Gayathri R, Jaacks LM, et al. Cashew Nut Consumption Increases HDL Cholesterol and Reduces Systolic Blood Pressure in Asian Indians with Type 2 Diabetes: A 12-Week Randomized Controlled Trial. The Journal of nutrition. 2018;148(1):63-9.
59. Moreira Alves RD, Boroni Moreira AP, Macedo VS, et al. High-oleic peanuts: new perspective to attenuate glucose homeostasis disruption and inflammation related obesity. Obesity (Silver Spring, Md). 2014;22(9):1981-8.
60. Morgan JM, Horton K, Reese D, Carey C, Walker K, Capuzzi DM. Effects of walnut consumption as part of a low-fat, low-cholesterol diet on serum cardiovascular risk factors. International journal for vitamin and nutrition research Internationale Zeitschrift fur Vitamin- und Ernahrungsforschung Journal international de vitaminologie et de nutrition. 2002;72(5):341-7.
61. Morgan WA, Clayshulte BJ. Pecans lower low-density lipoprotein cholesterol in people with normal lipid levels. Journal of the American Dietetic Association. 2000;100(3):312-8.
62. Nagashree RS, Manjunath NK, Indu M, et al. Effect of a Diet Enriched with Fresh Coconut Saturated Fats on Plasma Lipids and Erythrocyte Fatty Acid Composition in Normal Adults. Journal of the American College of Nutrition. 2017;36(5):330-4.
63. Njike VY, Ayettey R, Petraro P, Treu JA, Katz DL. Walnut ingestion in adults at risk for diabetes: effects on body composition, diet quality, and cardiac risk measures. BMJ open diabetes research & care. 2015;3(1):e000115.
64. Njike VY, Kavak Y, Treu JA, Doughty K, Katz DL. Snacking, Satiety, and Weight: A Randomized, Controlled Trial. American journal of health promotion : AJHP. 2017;31(4):296-301.
65. Parham M, Heidari S, Khorramirad A, et al. Effects of pistachio nut supplementation on blood glucose in patients with type 2 diabetes: a randomized crossover trial. The review of diabetic studies : RDS. 2014;11(2):190-6.
66. Rajaram S, Burke K, Connell B, Myint T, Sabate J. A monounsaturated fatty acid-rich pecan-enriched diet favorably alters the serum lipid profile of healthy men and women. The Journal of nutrition. 2001;131(9):2275-9.
67. Robbins WA, Xun L, FitzGerald LZ, Esguerra S, Henning SM, Carpenter CL. Walnuts improve semen quality in men consuming a Western-style diet: randomized control dietary intervention trial. Biology of reproduction. 2012;87(4):101.
68. Rock CL, Flatt SW, Barkai HS, Pakiz B, Heath DD. Walnut consumption in a weight reduction intervention: effects on body weight, biological measures, blood pressure and satiety. Nutrition journal. 2017;16(1):76.
69. Ros E, Nunez I, Perez-Heras A, et al. A walnut diet improves endothelial function in hypercholesterolemic subjects: a randomized crossover trial. Circulation. 2004;109(13):1609-14.
70. Ruisinger JF, Gibson CA, Backes JM, et al. Statins and almonds to lower lipoproteins (the STALL Study). Journal of clinical lipidology. 2015;9(1):58-64.
71. Sabate J, Cordero-Macintyre Z, Siapco G, Torabian S, Haddad E. Does regular walnut consumption lead to weight gain? The British journal of nutrition. 2005;94(5):859-64.
72. Sabate J, Haddad E, Tanzman JS, Jambazian P, Rajaram S. Serum lipid response to the graduated enrichment of a Step I diet with almonds: a randomized feeding trial. The American journal of clinical nutrition. 2003;77(6):1379-84.

73. Sauder KA, McCrea CE, Ulbrecht JS, Kris-Etherton PM, West SG. Effects of pistachios on the lipid/lipoprotein profile, glycemic control, inflammation, and endothelial function in type 2 diabetes: A randomized trial. Metabolism: clinical and experimental. 2015;64(11):1521-9.
74. Schutte AE, Van Rooyen JM, Huisman HW, et al. Modulation of baroreflex sensitivity by walnuts versus cashew nuts in subjects with metabolic syndrome. American journal of hypertension. 2006;19(6):629-36.
75. Sheridan MJ, Cooper JN, Erario M, Cheifetz CE. Pistachio nut consumption and serum lipid levels. Journal of the American College of Nutrition. 2007;26(2):141-8.
76. Somerset SM, Graham L, Markwell K. Isoenergetic replacement of dietary saturated with monounsaturated fat via macadamia nuts enhances endothelial function in overweight subjects. e-SPEN Journal. 2013;8(3):e113-e9.
77. Spaccarotella KJ, Kris-Etherton PM, Stone WL, et al. The effect of walnut intake on factors related to prostate and vascular health in older men. Nutrition journal. 2008;7:13.
78. Spiller GA, Jenkins DA, Bosello O, Gates JE, Cragen LN, Bruce B. Nuts and plasma lipids: an almond-based diet lowers LDL-C while preserving HDL-C. Journal of the American College of Nutrition. 1998;17(3):285-90.
79. Sweazea KL, Johnston CS, Ricklefs KD, Petersen KN. Almond supplementation in the absence of dietary advice significantly reduces C-reactive protein in subjects with type 2 diabetes. Journal of Functional Foods. 2014;10:252-9.
80. Tan SY, Mattes RD. Appetitive, dietary and health effects of almonds consumed with meals or as snacks: a randomized, controlled trial. European journal of clinical nutrition. 2013;67(11):1205-14.
81. Tapsell L, Batterham M, Tan SY, Warensjo E. The effect of a calorie controlled diet containing walnuts on substrate oxidation during 8-hours in a room calorimeter. Journal of the American College of Nutrition. 2009;28(5):611-7.
82. Tapsell LC, Gillen LJ, Patch CS, et al. Including walnuts in a low-fat/modified-fat diet improves HDL cholesterol-to-total cholesterol ratios in patients with type 2 diabetes. Diabetes care. 2004;27(12):2777-83.
83. Tapsell LC, Lonergan M, Batterham MJ, et al. Effect of interdisciplinary care on weight loss: a randomised controlled trial. BMJ open. 2017;7(7):e014533.
84. Tey SL, Brown R, Gray A, Chisholm A, Delahunty C. Nuts improve diet quality compared to other energy-dense snacks while maintaining body weight. Journal of nutrition and metabolism. 2011;2011:357350.
85. Tey SL, Gray AR, Chisholm AW, Delahunty CM, Brown RC. The dose of hazelnuts influences acceptance and diet quality but not inflammatory markers and body composition in overweight and obese individuals. The Journal of nutrition. 2013;143(8):1254-62.
86. Tindall AM, Petersen KS, Skulas-Ray AC, Richter CK, Proctor DN, Kris-Etherton PM. Replacing Saturated Fat With Walnuts or VegTable Oils Improves Central Blood Pressure and Serum Lipids in Adults at Risk for Cardiovascular Disease: A Randomized Controlled-Feeding Trial. Journal of the American Heart Association. 2019;8(9):e011512.
87. Tsaban G, Wolak A, Avni-Hassid H, et al. Dynamics of intrapericardial and extrapericardial fat tissues during long-term, dietary-induced, moderate weight loss. The American journal of clinical nutrition. 2017;106(4):984-95.

88. Vergani E, Bruno C, Olivieri G, et al. Differential metabolic response to various regimens of natural antioxidant enriched-diets in patients with insulin resistance. Endocrine Reviews Conference: 100th Annual Meeting of the Endocrine Society, ENDO. 2018;39(2 Supplement 1).
89. Wang X, Li Z, Liu Y, Lv X, Yang W. Effects of pistachios on body weight in Chinese subjects with metabolic syndrome. Nutrition journal. 2012;11:20.
90. Wien M, Bleich D, Raghuwanshi M, et al. Almond consumption and cardiovascular risk factors in adults with prediabetes. Journal of the American College of Nutrition. 2010;29(3):189-97.
91. Wien M, Oda K, Sabate J. A randomized controlled trial to evaluate the effect of incorporating peanuts into an American Diabetes Association meal plan on the nutrient profile of the total diet and cardiometabolic parameters of adults with type 2 diabetes. Nutrition journal. 2014;13:10.
92. Wien MA, Sabate JM, Ikle DN, Cole SE, Kandeel FR. Almonds vs complex carbohydrates in a weight reduction program. International journal of obesity and related metabolic disorders : journal of the International Association for the Study of Obesity. 2003;27(11):1365-72.
93. Williams PT, Bergeron N, Chiu S, Krauss RM. A randomized, controlled trial on the effects of almonds on lipoprotein response to a higher carbohydrate, lower fat diet in men and women with abdominal adiposity. Lipids in health and disease. 2019;18(1):83.
94. Wilson T YJ, Anderson AD, Anderson MM, Jacobson JL, Popko MR, Wang Y, Singh AP, Vorsa N, Lomburg PJ, Carughi A. Effect of bedtime pistachio consumption for 6 weeks on weight, lipid profile and glycemic status in overweight persons. International Journal of Food and Nutritional Science. 2014;1(1):1-4.
95. Wu H, Pan A, Yu Z, et al. Lifestyle counseling and supplementation with flaxseed or walnuts influence the management of metabolic syndrome. The Journal of nutrition. 2010;140(11):1937-42.
96. Zambon D, Sabate J, Munoz S, et al. Substituting walnuts for monounsaturated fat improves the serum lipid profile of hypercholesterolemic men and women. A randomized crossover trial. Annals of internal medicine. 2000;132(7):538-46.
97. Duarte GBS, Reis BZ, Rogero MM, et al. Consumption of Brazil nuts with high selenium levels increased inflammation biomarkers in obese women: A randomized controlled trial. Nutrition (Burbank, Los Angeles County, Calif). 2019;63-64:162-8.
98. Bamberger C, Rossmeier A, Lechner K, et al. A Walnut-Enriched Diet Reduces Lipids in Healthy Caucasian Subjects, Independent of Recommended Macronutrient Replacement and Time Point of Consumption: a Prospective, Randomized, Controlled Trial. Nutrients. 2017;9(10).
99. Sanchez-Muniz FJ, Canales A, Nus M, et al. The antioxidant status response to low-fat and walnut paste-enriched meat differs in volunteers at high cardiovascular Risk carrying different PON-1 polymorphisms. Journal of the American College of Nutrition. 2012;31(3):194-205.
100. Olmedilla-Alonso B, Granado-Lorencio F, Herrero-Barbudo C, Blanco-Navarro I, Blazquez-Garcia S, Perez-Sacristan B. Consumption of restructured meat products with added walnuts has a cholesterol-lowering effect in subjects at high cardiovascular risk: a randomised, crossover, placebo-controlled study. Journal of the American College of Nutrition. 2008;27(2):342-8.
101. Mukuddem-Petersen J, Stonehouse Oosthuizen W, Jerling JC, Hanekom SM, White Z. Effects of a high walnut and high cashew nut diet on selected markers of the metabolic syndrome: a controlled feeding trial. The British journal of nutrition. 2007;97(6):1144-53.

102. Butler TL, Fraser GE, Beeson WL, et al. Cohort profile: The Adventist Health Study-2 (AHS-2). International journal of epidemiology. 2008;37(2):260-5.
103. Johnston BC, Kanters S, Bandayrel K, et al. Comparison of weight loss among named diet programs in overweight and obese adults: a meta-analysis. Jama. 2014;312(9):923-33.
104. Higgins JPT TJ, Chandler J, Cumpston M, Li T, Page MJ, Welch VA (editors). Cochrane Handbook for Systematic Reviews of Interventions version 6.0 (updated July 2019). Cochrane, 2019. Available from www.training.cochrane.org/handbook. 2019.
